# Secondary Phosphines Bearing N‑Heterocyclic Imine Groups: Polarity Umpolung of Highly Electron-Rich P–H Bonds

**DOI:** 10.1021/acs.inorgchem.5c04504

**Published:** 2025-11-26

**Authors:** Maike B. Röthel, Tobias Eder, Franka Brylak, Michael Seidl, Pawel Löwe, Fabian Dielmann

**Affiliations:** a Institute of General, Inorganic and Theoretical Chemistry, 27255 Universität Innsbruck, Innsbruck 6020, Austria; b Institute of Inorganic and Analytical Chemistry, Universität Münster, Münster 48149, Germany

## Abstract

The synthesis and reactivity of secondary phosphines carrying two N-heterocyclic imine substituents are reported. These highly electron-rich phosphines feature a polarized P–H bond, with computational studies revealing their dual nature as excellent hydride donors and superbasic phosphorus nucleophiles. Experimental investigations demonstrated their ability to transfer a hydride, proton, or hydrogen atom, depending on the reaction partner, as well as their reactivity in hydrophosphination reactions. The hydrophosphination of nonactivated alkynes proceeded under mild, metal-free conditions, yielding alkenylphosphine intermediates that partly rearranged into azaphosphole heterocycles.

## Graphical abstract

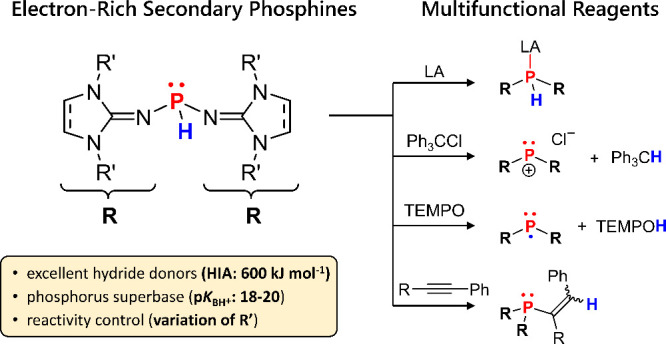

## Introduction

The P–H bond is generally characterized by low polarity due to the nearly identical electronegativity values of phosphorus and hydrogen (Pauling: χ^P^ = 2.19, χ^H^ = 2.20).^1^ However, the polarity of this bond is significantly influenced by the substituents attached to the phosphorus atom, resulting in a wide range of possible reaction pathways for secondary phosphines (Figure 1a), with the predominant reactivity being tunable through electronic and steric characteristics.

**Figure 1:**
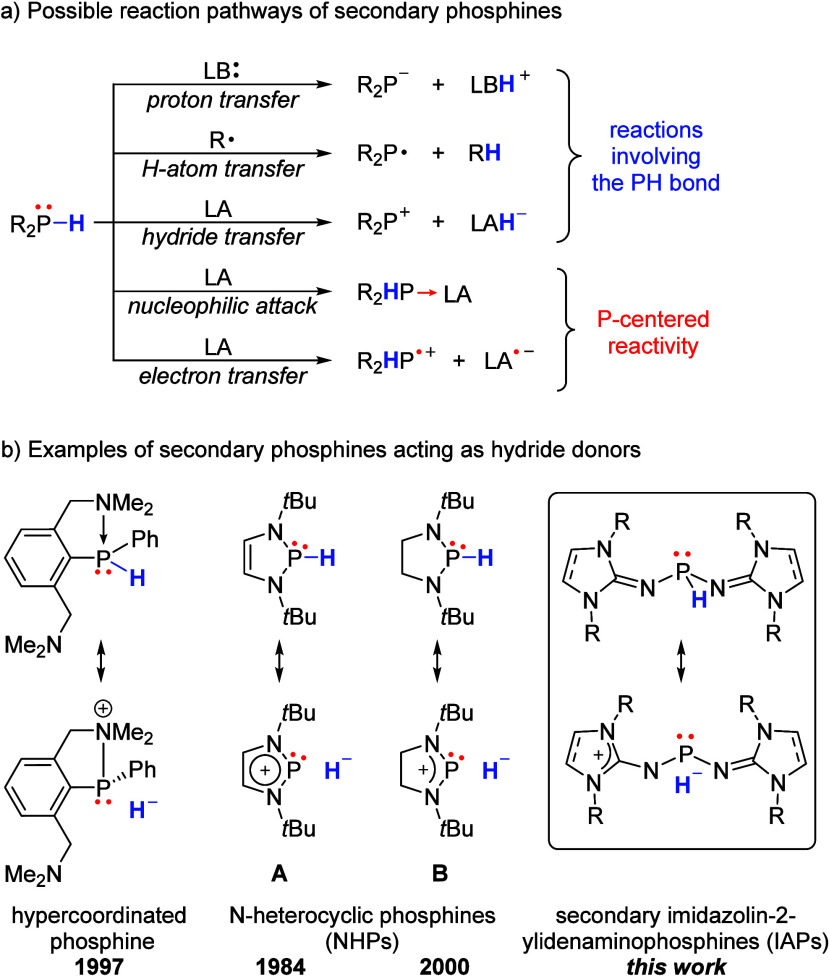
(a) Schematic overview of reaction pathways of secondary phosphines; LA = Lewis acid, LB = Lewis base. (b) Resonance structures of selected secondary phosphines reflecting the negative polarization of the P–H bond toward the hydrogen atom.

Secondary organophosphines bearing two alkyl- or aryl substituents typically act as proton donors, and the resulting organophosphides serve as valuable building blocks.^2,3^ These compounds are also prone to homolysis of the P–H bond by radicals which is a common pathway in hydrophosphination reactions.^4,5^ In contrast, heterolytic cleavage of the P–H bond to deliver a hydride is uncommon as these substituents are inefficient at stabilizing the remaining phosphenium cation.^6–9^

The first successful strategy to alter this reactivity and facilitate hydride transfer involved the introduction of an adjacent amino group, which interacts with the phosphorus center and stabilizes the phosphenium cation (Figure 1b, left).^10^ Another approach to enhance the hydridic reactivity of secondary phosphines involves the use of π-donating substituents at phosphorus, which polarize the P–H bond via negative hyperconjugation into the σ*­(P–H) orbital. This π-conjugation is particularly pronounced in N-heterocyclic phosphines (NHPs), as reflected by their resonance structures (Figure 1b, center), resulting in elongation of the P–H bond and a “polarity umpolung.”^11^

Over the past decade, the structural diversity of NHPs has expanded significantly, and they have emerged as powerful stoichiometric and catalytic reductants in organic synthesis, enabling transformations such as hydrogenations, hydrodefluorinations, and *N*-formylations of amines using carbon dioxide.^12–17^

More recently, the ability of NHPs to undergo homolytic P–H bond cleavage, initiated by radical starters or UV irradiation, has been utilized in applications such as the dehalogenation of aromatics^18,19^ and the chemoselective deoxygenation of aryl esters.^20^

Beyond reactions involving the P–H bond, secondary phosphines generally also exhibit phosphorus-centered reactivity, including the formation of complexes with Lewis acids and electron transfer reactions (Figure 1a).^11,21^

Herein, we report the synthesis and reactivity of a new class of secondary phosphines featuring N-heterocyclic imine (NHI) substituents (Figure 1b, right). NHIs are strong π-donating substituents with tunable steric and electronic properties,^22,23^ making them well-suited to impart both thermodynamic and kinetic stability to electron-deficient phosphorus centers. This has enabled the isolation of a variety of phosphorus species, including three-coordinate phosphonium cations, [(NHI)_2_P=E]^+^ (E = O, S, Se, NR, CR_2_),^24–28^ two-coordinate phosphenium cations, (NHI)_2_P^+^,^29,30^ and phosphinyl radicals (NHI)_2_P·.^31,32^ In turn, phosphines carrying NHIs exhibit exceptional nucleophilicity and electron-donating ability.^33–36^ Collectively, these findings suggest that NHI-bearing secondary phosphines, (NHI)_2_PH, possess multiple, competing reaction pathways. In this study, we investigate these pathways and explore the reactivity profile of this new class of electron-rich secondary phosphines.

## Results and Discussion

### Synthesis and Characterization

We prepared secondary phosphines with three distinct NHI substituents to investigate how variations in steric (*R* ^1^ versus *R* ^2^) and electronic (*R* ^1^ versus *R* ^3^) properties of the NHI substituents influence the PH-centered reactivity (Figure 2). Notably, NHIs with a double bond in the backbone are known to exhibit stronger π-donating properties compared to those with a saturated backbone, while the substituents on the nitrogen atoms have minimal impact on this characteristic.^37^

**Figure 2:**
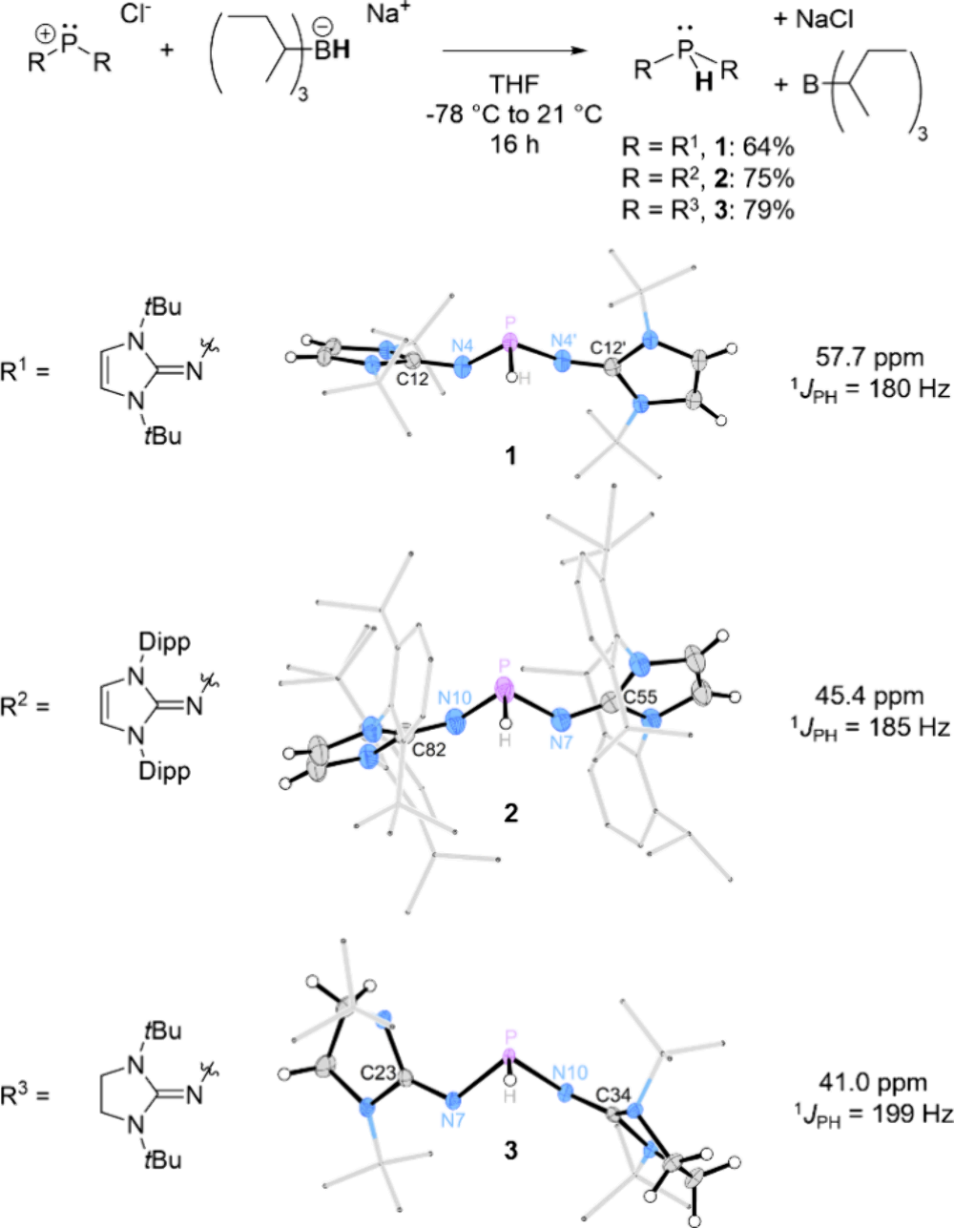
Top: Synthesis of secondary phosphines **1–3** starting from phosphenium chlorides; bottom: solid-state structures of **1–3** (positional disorders and most hydrogen atoms are omitted for clarity; thermal ellipsoids are set at 50% probability; selected bond lengths (Å) and angles (°): **1**: P–H 1.45(9), P–N4 1.630(6), N4–C12 1.283(5), P–N4′ 1.656(6), N4′–C12′ 1.283(5), N4–P–H 103.1(33), N4′–P–H 102.(3), N4–P–N4′ 101.5(4); **2**: P–H 1.50(3), P–N7 1.713(3), P–N10 1.645(2), N7–C55 1.2751(16), N10–C82 1.273(2), N10–P–H 101.7(11), N7–P–H 98.1(10), N10–P–N7 97.58(12); **3**: P–H 1.48(3), P–N7 1.726(4), P–N10 1.681(4), N7–C23 1.277(2), N10–C34 1.278(2), N7–P–H 97.7(15), N10–P–H 101.4(15), N7–P–N10 97.2(2)); ^31^P NMR chemical shifts in C_6_D_6_ with ^1^ *J*_PH_ coupling constants.

The secondary imidazolin-2-ylidenaminophosphines (IAPs) **1** **–** **3** (Figure 2) were synthesized by reacting the corresponding phosphenium salts with sodium tri*sec*-butylborohydride (*N*-Selectride). The use of bulky alkyl groups on boron was necessary to prevent formation of phosphine borane adducts between the reaction products. After workup, the phosphines were isolated as white (**3**) to yellow (**1**, **2**) solids in moderate to good yields (64%–79%). The secondary phosphines **1** **–** **3** are soluble in THF and in nonpolar solvents such as *n*-hexane and toluene. In line with their high reactivity (vide infra), they decompose rapidly in dichloromethane, chloroform, or acetonitrile and are highly air sensitive. In the ^31^P NMR spectra of the secondary phosphines, a doublet was observed for each compound (**1**: 57.7 ppm, **2**: 45.4 ppm, **3**: 41.0 ppm), with the P–H coupling constants (^1^ *J*_PH_) ranging from 180 to 199 Hz. The corresponding ^1^H NMR resonances for the P–H bond were found at 5.72 ppm (**2**), 8.07 ppm (**3**) and 8.42 ppm (**1**). The lower chemical shift of the P–H resonance in **2** is attributed to shielding effects from the flanking diisopropylphenyl substituents. The observed coupling constants are slightly higher than those reported for NHPs (^1^ *J*_PH_ = 132–181 Hz)^11^ and are comparable to those of secondary alkylphosphines (HP*i*Pr_2_: ^1^ *J*_PH_ = 192 Hz,^38^ HPCy_2_: ^1^ *J*_PH_ = 194 Hz^39^). In contrast, secondary arylphosphines mostly exhibit higher coupling constants ranging from ^1^ *J*_PH_ = 198 to 225 Hz.^39^

Single crystals suitable for X-ray diffraction (SCXRD) analysis were obtained for all three phosphines. A representative molecule for each phosphine is depicted in Figure 2. In all three structures, the phosphorus centers exhibit the expected pyramidal coordination geometry. Notable structural differences include the increased steric shielding of the P–H unit by the bulky diisopropylphenyl groups in **2** and the contrasting geometries of the N atoms within the heterocycles of **1** (planar) and **3** (pyramidalized). This difference aligns with the lower energy penalty for nitrogen pyramidalization in saturated NHIs. The positional disorder of the central phosphorus unit in all three solid-state structures precludes a detailed discussion of geometrical parameters (see the Supporting Information for details).

The Kohn–Sham orbitals and natural bond orbitals (NBOs) of **1** and **3** were calculated using density functional theory (DFT) at the B3LYP/6-311+G­(d,p) level (Figure 3). The respective highest occupied molecular orbital (HOMO) is localized primarily at the phosphorus center, representing the lone pair and σ­(P–H) bond, with smaller orbital contributions from the π system of the NHI substituents. The lower ionization energy of **1** (5.43 eV) compared to **3** (6.29 eV) is consistent with the stronger π-donor ability of NHIs with unsaturated backbone, leading to a more electron-rich P–H unit. The lowest unoccupied molecular orbitals (LUMOs) are well-separated in energy, indicating minimal electrophilic reactivity. The NBO analysis indicates the σ-type nature of the P–H bond with a significant contribution from the s orbital of the H atom (**1**: 55.0%, **3**: 53.8%) and a polarization toward the hydrogen atom, which is the only hydrogen in the molecule that exhibits a negative natural atomic charge (*q*(H) = −0.114 (**1**), −0.084 (**3**), Table S16). To further assess the reactivity of the P–H bond, the p*K*_a_ and p*K*_BH_^+^ values in DMSO, and the bond dissociation free energies (BDFEs) in toluene for compounds **1** and **3**, as well as the hydride ion affinities (HIAs) of their corresponding phosphenium cations, were calculated (Table 1). For comparison, the NHPs **A** and **B** (Figure 1), which feature *tert*-butyl groups on the endocyclic nitrogen atoms and either a saturated or unsaturated backbone, were also included in the analysis. The calculated p*K*_BH_^+^ values of the secondary phosphines reveal that **1** and **3** are significantly more basic than the NHPs **A** and **B** (Table 1). Notably, the basicity of **1** and **3** falls within the range of phosphazene superbases, whereas **A** and **B** are weak bases, comparable to pyridine.^40^ Despite this pronounced difference in phosphorus basicity, the acidity of the P–H bond is in a similar range for all compounds (p*K*_a_ = 35–42), indicating that deprotonation requires extreme conditions, such as the use of alkali metal organyls. The calculated HIA values suggest that secondary IAPs **1** and **3** more readily donate a hydride than NHPs. Remarkably, the HIA values for all these secondary phosphines are even lower than those of diisobutylaluminum hydride (HIA of DIBAL_2_^+^: 787 kJ mol^–1^) and tributyltin hydride (HIA of *n*Bu_3_Sn^+^: 886 kJ mol^–1^),^41^ highlighting the exceptional potential of phosphines **1** **–** **3** to transfer hydrides. Furthermore, the BDFE for the P–H bond in **3** (64.9 kcal mol^–1^) is slightly lower than that in **1** (68.4 kcal mol^–1^). However, both values are within a similar range to those reported for NHPs.^42^

**Figure 3:**
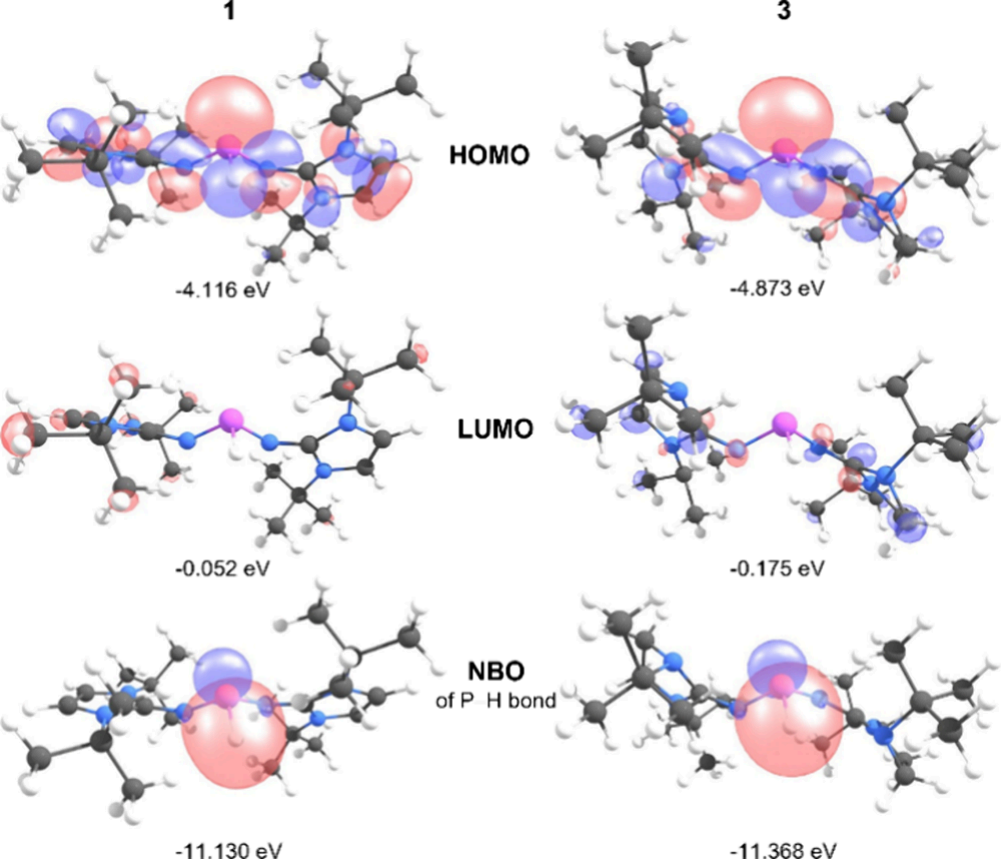
Views of the Kohn–Sham orbitals (HOMO and LUMO) and the natural bond orbital (NBO) representing the P–H bond of **1** (left) and **3** (right), along with their corresponding energies, as determined by DFT calculations (B3LYP/6-311+G­(d,p)).

**Table 1:** Calculated p*K*_a_ and p*K*_BH_^+^ Values in DMSO of the Secondary NHI-Substituted Phosphines (**1** and **3**) and Secondary N-Heterocyclic Phosphines (NHPs, **A**: 1,3-di-*tert*-butyl-2-hydrido-1,3,2-diazaphosphole, **B**: 1,3-di-*tert*-butyl-2-hydrido-1,3,2-diazaphospholidine) and Hydride Ion Affinities (HIA) of the Corresponding Phosphenium Cations and Bond Dissociation Free Energies (BDFEs) of the Phosphines

	1	3	A	B
**p** ***K***_**BH**_ **+** ^a^	20.1	17.5	–0.672	4.26
**p** ***K***_**a**_ ^b^	38.4	35.1	38.3	41.5
**HIA** ^c^/kJ mol^–1^	607	596	671	731
**BDFE**/kcal mol^–1^	64.9^d^	68.4^d^	(62.3 ^42^)	(70.7 ^42^)

a Calculated using DFT (B3LYP/6-311++(2df,2p)/SMD­(DMSO)) considering R_2_PH_2_^+^ + C_6_H_5_NH^–^ → R_2_PH + C_6_H_5_NH_2_.

b Calculated using DFT (B3LYP/6-311++(2df,2p)/SMD­(DMSO)) considering R_2_PH + C_6_H_5_NH^–^ → R_2_P^–^ + C_6_H_5_NH_2_.

c Calculated using DFT (B3LYP/def2-TZVPP) considering R_2_P^+^ + Me_3_SiH → R_2_PH + Me_3_Si ^+^.

d Calculated using DFT (B3LYP/6-311+G­(d,p)/SCRF­(toluene)) considering R_2_PH + TEMPO· → R_2_P· + TEMPOH.

### Nucleophilic Reactivity and Phosphine Donor Properties

To gauge the donor properties of the secondary phosphines **1** **–** **3**, two commonly used descriptors were employed: the Tolman electronic parameter (TEP)^43^ and the ^1^ *J*_PSe_ coupling constant^44–46^ (Figure 4). Both parameters decrease with increasing donor strength of the corresponding phosphine. IR spectroscopic analysis of the phosphine complexes [Ni­(CO)_3_(L)] (L = **1** **–** **3**) in dichloromethane revealed TEP values of 2043.1 cm^–1^ (**1**), 2048.7 cm^–1^ (**2**) and 2051.5 cm^–1^ (**3**). The ^1^ *J*_PSe_ coupling constants of the phosphine selenides, recorded in deuterated benzene, also follow the same qualitative trend as the TEP values (**1**: 673 Hz, **2**: 687 Hz, **3**: 695 Hz). In the more CH-acidic solvent dichloromethane, the coupling constants for the sterically more accessible *tert*-butyl derivatives **1** and **3** are significantly smaller (**1**: 638 Hz, **2**: 673 Hz, **3**: 659 Hz), likely due to solvent effects, which are a known issue.^44,47–49^ Overall, both methods rank secondary phosphines with unsaturated NHI backbones (**1** and **2**) as stronger donors compared to **3**, consistent with previous observations.^37^ The donor properties of these phosphines fall within the range of classical NHCs^50^ and other phosphines with strongly donating substituents,^33,34,51–55^ but they are significantly stronger donors than secondary arylphosphines (e.g., HPPh_2_ TEP: 2073.3 cm^–1^).^56^

**Figure 4:**
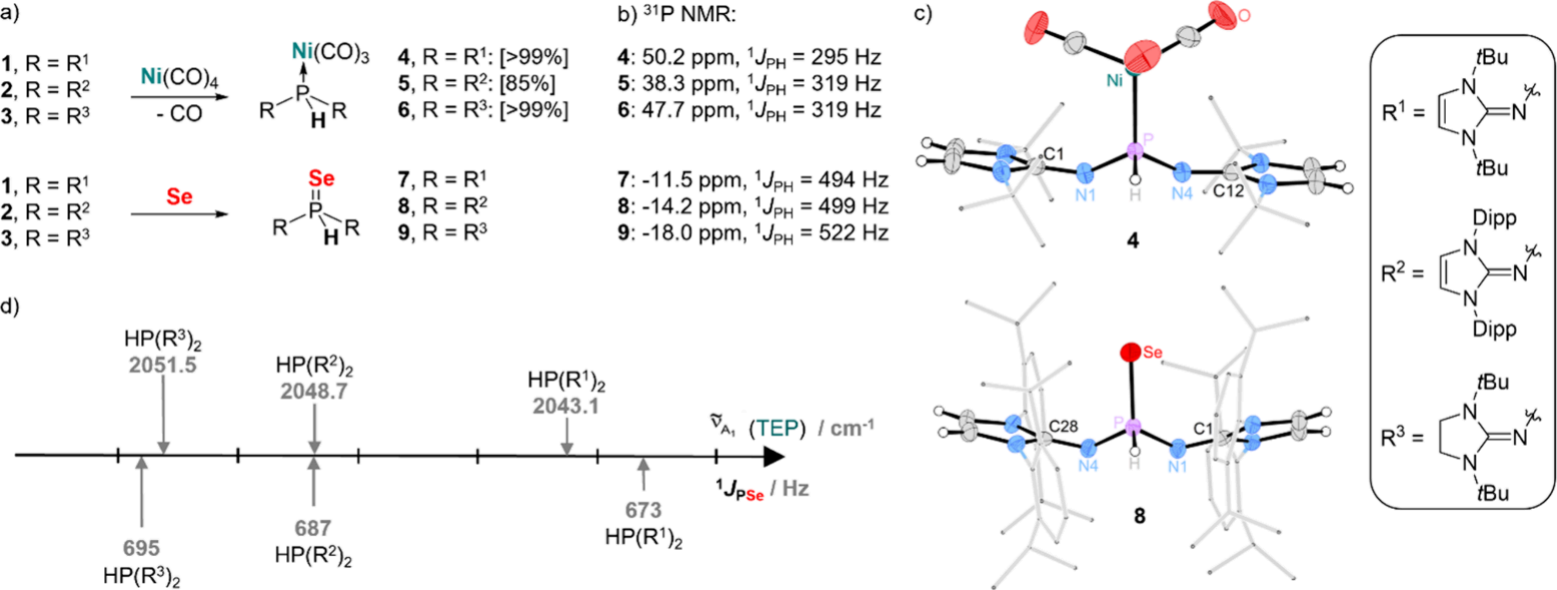
Gauging the donor properties of the secondary phosphines **1** **–3**. (a) Syntheses of [Ni­(CO)_3_(PHR_2_)] and SePHR_2_, [yields according to ^31^P NMR in brackets]. (b) ^31^P NMR chemical shifts and ^1^ *J*_PH_ coupling constants obtained in C_6_D_6_. (c) Solid-state structures of **8** and **4** (positional disorders, solvent molecule, and most hydrogen atoms are omitted for clarity; thermal ellipsoids are set at 50% probability; selected bond lengths (Å) and angles (°): **8**: P–Se 2.1132(7), P–H 1.33(3), P–N1 1.607(2), P–N4 1.606(2), N4–C28 1.296(3), N1–C1 1.292(3), N4–P–H 106.4(12), H–P–N1 101.8(12), N4–P–N1 102.89(10); **4**: P–Ni 2.2608(7), P–H 1.34(3), P–N1 1.636(2), P–N4 1.635(2), N1–C1 1.295(3), N4–C12 1–294(3), N4–P–H 106.7(12), H–P–N1 102.2(12), N1–P–N4 101.77(9)). (d) TEP values (in DCM) and ^1^ *J*_PSe_ coupling constants (in C_6_D_6_).

The ability of the secondary phosphines to act as phosphorus nucleophiles is demonstrated by the successful preparation of nickel complexes **4**-**6** and the phosphine selenides **7**-**9**. The reactivity with nitrous oxide, borane and a gold­(I) NHC complex further supports this as the preferred reaction pathway (Figure 5). All three phosphines react selectively with nitrous oxide at ambient temperature to afford the corresponding phosphine oxides in quantitative yield. Notably, the reaction with **2** takes significantly longer (2 weeks) than with **1** and **3** (maximum 15 min). The phosphine oxides exhibit larger ^1^ *J*_PH_ coupling constants in the ^31^P NMR spectrum (**10**: 539, **11**: 535, **12**: 580 Hz) and more upfield chemical shifts (**10**: −26.0, **11**: −20.8, **12**: −36.3 ppm) compared to the free phosphines.

**Figure 5:**
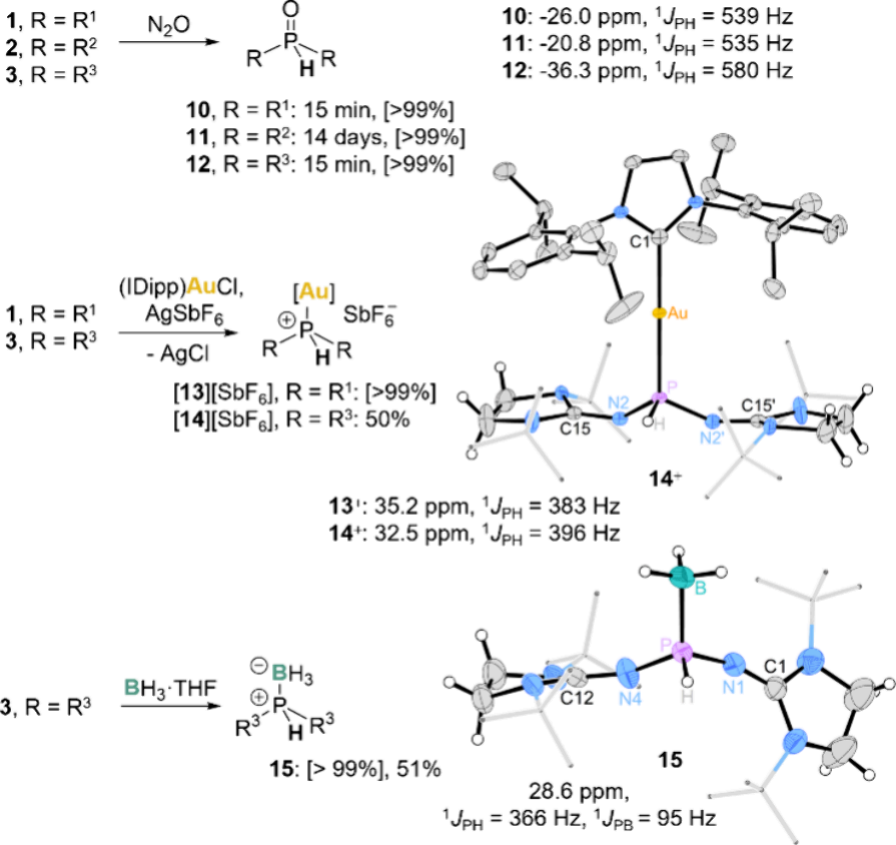
Reactions of **1–3** with nitrous oxide, borane, and in situ-generated gold­(I) complex [(IDipp)­Au]­[SbF_6_], in which the secondary phosphines act as nucleophiles [yields according to ^31^P NMR in brackets]. ^31^P NMR data measured in C_6_D_6_ (**13** and **14** in CD_2_Cl_2_). Solid-state structures of **14** and **15**; positional disorders, solvent molecules, and most hydrogen atoms are omitted for clarity; thermal ellipsoids are set at 50% probability; selected bond lengths (Å) and angles (°): **14**: P–Au 2.2832(15), P–H 1.33(10), P–N2 1.571(3), P–N2′ 1.607(3), N2–C15 1.296(4), N2′–C15′ 1.296(4), Au–C1 2.052(4), P–Au–C1 178.67(4), N2–P–H 104.(4), N2–P–N2 107.4(2); **15**: P–B 1.907(3), P–H 1.29(3), P–N4 1.597(2), P–N1 1.608(2), N4–C12 1.270(3), N1–C1 1.265(3), N4–P–H 106.4(12), N4–P–N1 108.02(12), H–P–N1 108.1(12).

The reaction of **1** and **3** with the in situ generated complex [(IDipp)­Au]­[SbF_6_] (IDipp = 1,3-bis­(2,6-diisopropylphenyl)-imidazol-2-ylidene) led to coordination of the phosphine to the gold­(I) atom. The ^31^P NMR spectra of the resulting complexes show resonances at 35.2 ppm (**13**) and 32.5 ppm (**14**), with ^1^ *J*_PH_ coupling constants of 383 and 396 Hz, respectively. Complex **14** was further characterized by SCXRD revealing a linear coordination geometry (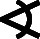
C–Au–P = 178.67(4)°) (Figure 5).

Phosphine **3** also acts as phosphorus nucleophile when treated with borane tetrahydrofuran complex (BH_3_·THF). ^31^P NMR analysis indicated complete consumption of the phosphine **3**, and quantitative conversion into the phosphine borane adduct **15**, showing that this reaction pathway is preferred over an adduct formation with the exocyclic nitrogen atoms at the NHI substituents or the hydride transfer. The ^31^P NMR spectrum of **15** shows a signal at 28.6 ppm with coupling constants of ^1^ *J*_PH_ = 366 Hz and ^1^ *J*_PB_ = 95 Hz, and the ^11^B NMR spectrum exhibits a resonance at −32.8 ppm with coupling constants of ^1^ *J*_BH_ = 95 Hz and ^1^ *J*_PB_ = 95 Hz. The structure of **15** was also confirmed by SCXRD analysis (Figure 5).

### Hydrogen Atom Transfer

To assess whether secondary phosphines **1** **–** **3** act as hydrogen atom donors, their reactions with substoichiometric amounts of 2,2,6,6-tetramethylpiperidinyloxyl (TEMPO) were analyzed by electron paramagnetic resonance (EPR) spectroscopy. The sterically accessible phosphines **1** and **3** readily undergo hydrogen atom abstraction, as evidenced by the decrease in the TEMPO resonance and the appearance of phosphinyl radical signals (Figure 6). The calculated BDFEs of these phosphines (**1**: 64.9 kcal mol^–1^, **3**: 68.4 kcal mol^–1^) are very similar to that of TEMPO (65.2 kcal mol^–1^)^57^ suggesting the feasibility of the hydrogen atom transfer process. For **1**, a doublet at *g* = 2.003 with a coupling constant of *a*(^31^P) = 66 G was observed immediately after mixing the reactants. In the case of **3**, the TEMPO resonance disappeared within 3 min. Upon heating to 100 °C, a similar doublet (*g* = 2.003, *a*(^31^P) = 71 G), as observed in the case of **1**, appeared. These signals are consistent with phosphinyl radicals, showing hyperfine coupling to the phosphorus nucleus but no coupling to ^14^N, and align with previously reported values for a similar NHI-substituted phosphinyl radical (*g* = 2.005, *a*(^31^P) = 78 G)^31^ and other phosphinyl radicals (*a*(^31^P) = 63–100 G).^58,59^ In contrast, phosphine **2** showed no significant decrease in the TEMPO signal, even upon heating, likely due to steric shielding of the P–H bond by the Dipp groups.

**Figure 6:**
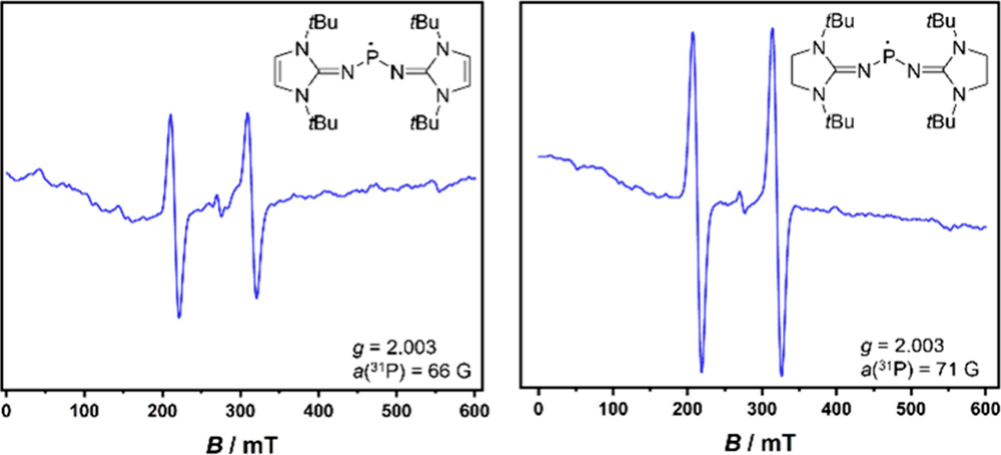
EPR spectra of phosphinyl radicals generated after the addition of **1** (left) and **3** (right) to TEMPO in toluene; left: ∼1 min after addition, right: after heating the mixture to 100 °C.

NMR experiments corroborate these findings: **1** and **3** react quantitatively with two equivalents of TEMPO to form phosphine oxides **16** and **17**, respectively, which exhibit singlets in the ^31^P NMR spectrum at −25.0 ppm (**16**) and −28.1 ppm (**17**), with no observable phosphorus–hydrogen coupling. A SCXRD study confirms the structure of phosphine oxide **16**, whose formation can be rationalized by coupling of the phosphinyl and TEMPO radicals followed by reaction with the generated TEMPOH (Figure 7, also see Figure S84). Notably, the reaction of **3** is slower compared to **1**, while **2** shows no reaction with TEMPO in the NMR experiment.

**Figure 7:**
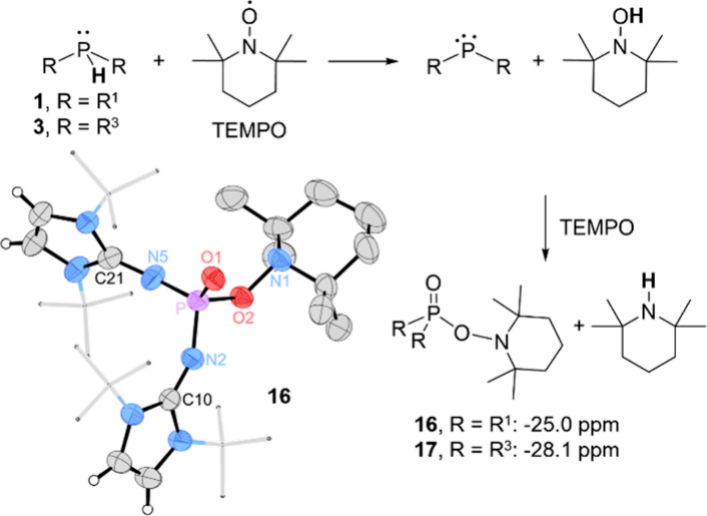
Reaction of secondary phosphines **1** and **3** with TEMPO. ^31^P NMR data measured in C_6_D_6_. Solid-state structure of **16**; solvent molecules and most hydrogen atoms are omitted for clarity; thermal ellipsoids are set at 50% probability; selected bond lengths (Å) and angles (°): P–O1 1.4741(16), P–O2 1.6453(15), P–N2 1.591(2), P–N5 1.585(2), O2–N1 1.470(2), N2–C10 1.285(3), N5–C21 1.293(3), N2–P–N5 110.20(11), N2–P–O2 98.77(9), O2–P–N5 102.54(10), O2–P–O1 110.96(9).

### Hydride Transfer

Although the secondary phosphines readily act as phosphorus nucleophiles (vide supra), the reaction of **1** and **3** with chlorotriphenylmethane highlights their hydride transfer capability (Figure 8). The successful hydride transfer is evidenced by the detection of the strongly deshielded ^31^P NMR resonances corresponding to the phosphenium ions and the characteristic resonance of the tertiary CH group of triphenylmethane in the ^1^H NMR spectrum. The hydride ion affinity of the trityl cation ([Ph_3_C]^+^, 860 kJ mol^–1^)^60^ is significantly higher than those of the phosphenium ions corresponding to the secondary phosphines (Table 1), making the transfer reaction energetically favorable. The fact that this reaction occurs under ambient conditions and involves the cleavage of both the P–H and C–Cl bonds while only one covalent bond (C–H) is formed, underlines the exceptional hydride donor ability of the secondary phosphines. In contrast, for the NHPs and the hypercoordinated phosphine shown in Figure 1, as well as a 2,6-bis­(*o*-carborano)­pyridine-stabilized secondary phosphine,^61^ similar reactivity is only reported when activated ionic tritylium salts ([Ph_3_C]^+^[X]^−^, where X is a weakly coordinating anion) are used.^10,62^

**Figure 8:**
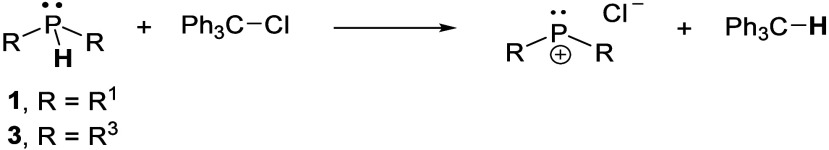
Reaction of secondary IAPs **1** and **3** with chlorotriphenylmethane undergoing hydride transfer.

### Proton Transfer

To further explore the reaction pathways of secondary IAPs, their reactivity toward proton acceptors was investigated. The resulting phosphides (PR_2_^–^) could serve as valuable intermediates for the synthesis of polydentate phosphines. Treatment with sodium borohydride (NaBH_4_), which typically produces hydrogen gas when reacting with acids, showed no reaction. Similarly, potassium *tert*-butoxide (KO*t*Bu) proved to be insufficiently basic to deprotonate the secondary phosphines. In line with the p*K*_a_ values of **1** and **3** (Table 1), the stronger bases *n*-butyllithium (*n*-BuLi) and lithium diisopropylamide (LDA) reacted with both **1** and **3**, albeit in an unselective manner, yielding species that lacked P–H coupling in their ^31^P NMR spectra. Interestingly, phosphine **2** also reacted with *n*-BuLi and LDA, but the resulting products retained P–H coupling, suggesting a different reaction pathway. This behavior highlights the efficient steric shielding of the P–H unit by the Dipp substituents, thereby governing the reactivity of **2.**

### Hydrophosphinations

The addition of P–H bonds to unsaturated substrates, known as hydrophosphination, has been studied for nearly 70 years^63^ and is now widely used in industrial phosphine synthesis.^4,64–68^ These reactions are typically metal-catalyzed, but can also proceed thermally, photochemically, or via radical initiation.^4,69^ Unlike secondary phosphines with aryl or alkyl groups, which require activation of the nonpolar P–H bond, NHPs undergo hydrophosphination with polar double bonds (e.g., aldehydes and ketones) without a catalyst due to their pronounced hydricity. However, hydrophosphination of nonpolar substrates, such as alkenes or alkynes, by NHPs has not been reported.^14,15^

We investigated the hydrophosphination of nonpolarized alkynes using secondary phosphines **1** **–3** (Figures 9 and 10). Indeed, compounds **1** and **3** formed hydrophosphination products, while **2** showed no reactivity, even at elevated temperatures, and is therefore excluded from further discussion. Reaction of **1** with phenylacetylene at room temperature resulted in full conversion after 18 h, yielding the (Z)-alkenylphosphine **18** as the major product (85% yield by ^31^P NMR analysis), which was isolated by crystallization. The ^31^P NMR spectrum showed a resonance at 63.5 ppm with characteristic coupling constants of the (Z)-isomer (^3^ *J*_PH_ = 23 Hz, ^2^ *J*_PH_ = 1.5 Hz, the ^2^ *J*_PH_ coupling constant is only distinguishable in the ^1^H NMR spectrum, see Table S2 for a comparison of the NMR data of alkenylphosphines). A SCXRD study confirmed the structure with the phenyl group and phosphorus center in *cis* confirmation. The C=C bond length (1.336(6) Å) and the pyramidal geometry at phosphorus (Σ­(N–P–X) = 295°) are consistent with a classic alkenylphosphine structure. Notably, prolonged reaction times led to decomposition of **18** into unidentified species.

**Figure 9:**
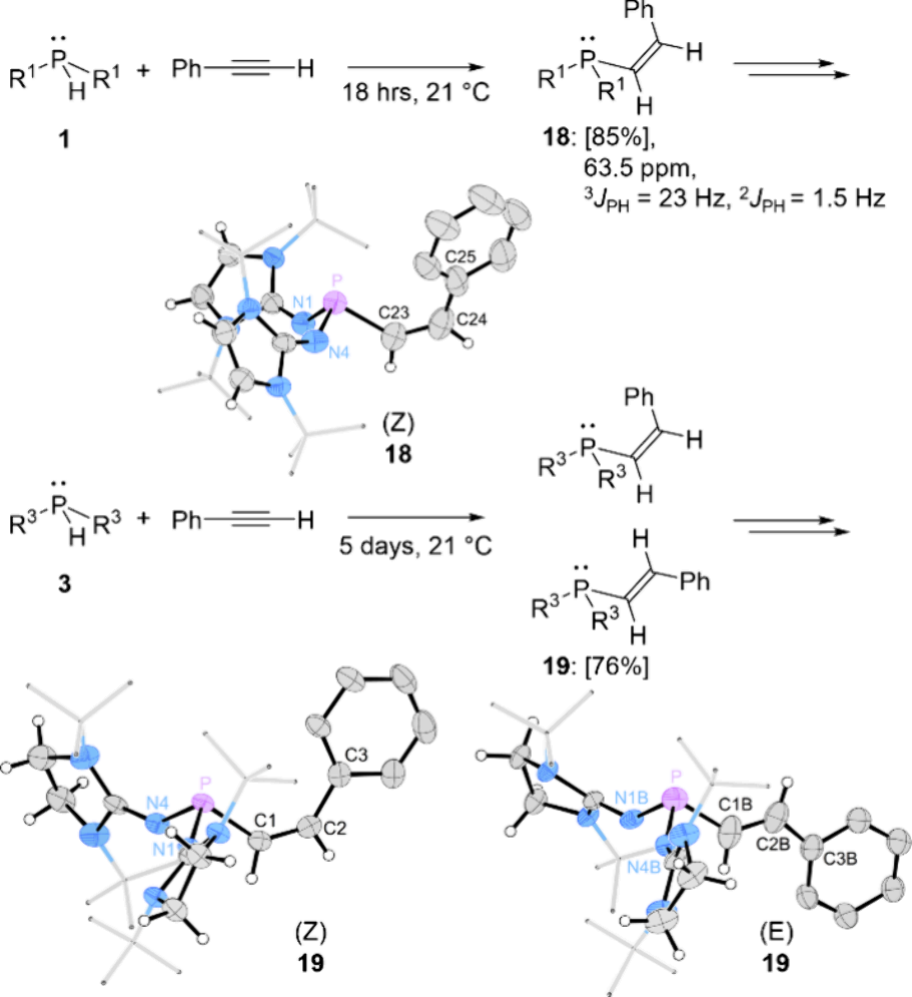
Reaction of secondary IAPs **1** (top) and **3** (bottom) with phenylacetylene yielding the alkenylphosphines **18** and **19** [yields according to ^31^P NMR in brackets]. ^31^P NMR data measured in C_6_D_6_. Solid-state structures of **18** and **19** (most hydrogen atoms are omitted for clarity; thermal ellipsoids are set at 50% probability; selected bond lengths (Å) and angles (°): **18**: P–N1 1.670(3), P–N4 1.673(3), P–C23 1.831(4), C23–C24 1.336(6), C24–C25 1.476(7), N1–P–N4 103.1(2), N4–P–C23 96.6(2), N1–P–C23 95.6(2); (Z)-**19**: P–N1 1.673(2), P–N4 1.684(2), P–C1 1.836(2), C1–C2 1.340(3), C2–C3 1.474(3), N1–P–N4 99.74(1), N4–P–C1 93.19(10), N1–P–C1 96.80(12); (E)-**19**: P–N1B 1.636(13), P–N4B 1.664(12), P–C1B 1.82(2), C1B–C2B 1.32(2), C2B–C3B 1.45(2), N1B–P–N4B 99.2(12), N4B–P–C1B 94.9(12), N1B–P–C1B 93.3(11)).

In contrast, the reaction of **3** with phenylacetylene proceeded more slowly, reaching >95% conversion only after 5 days at ambient temperature. A ^31^P NMR analysis revealed overlapping multiplets at 60 ppm, including a doublet with a coupling constant of *J*_PH_ = 20 Hz. By the means of SCXRD analysis we identified a mixture of (Z)- and (E)-isomers of alkenylphosphine **19** in a 9:1 ratio which matches the ratio estimated by quantitative ^31^P NMR analysis of the reaction mixture. The C=C bond lengths ((Z)-**19**: 1.340(3) Å, (E)-**19**: 1.319(18) Å) and pyramidal phosphorus geometries ((Z)-**19**: Σ­(N–P–X) = 289.73°, (E)-**19**: Σ­(N–P–X) = 287.4°) align with alkenylphosphine structures.^70^

Reactions with diphenylacetylene (tolane) showed markedly different rates depending on the phosphine. For compound **1** full conversion was achieved at room temperature within 72 h or at 70 °C within 2 h, while **3** required 48 h at 70 °C. ^31^P NMR spectra revealed doublet resonances for the alkenylphosphines (**20**: δ = 74.6 ppm, ^3^ *J*_PH_ = 24.2 Hz; **21**: δ = 81.5 ppm, ^3^ *J*_PH_ = 33.9 Hz), which diminished over time while singlet resonances for azaphosphole heterocycles (**22**: δ = 93.4 ppm, **23**: δ = 80.1 ppm) increased (Figure 10a). This indicates the subsequent transformation of the alkenylphoshines into the azaphospholes. The azaphospholes **22** and **23** were isolated in yields of 40% and 70%, respectively. The attempted recrystallization of **22** from dichloromethane solution resulted in the formation of the phospholium compound **24** via nucleophilic substitution at dichloromethane, as confirmed by SCXRD analysis, which retained the planar five-membered azaphosphole ring structure (Figure 10c).

**Figure 10:**
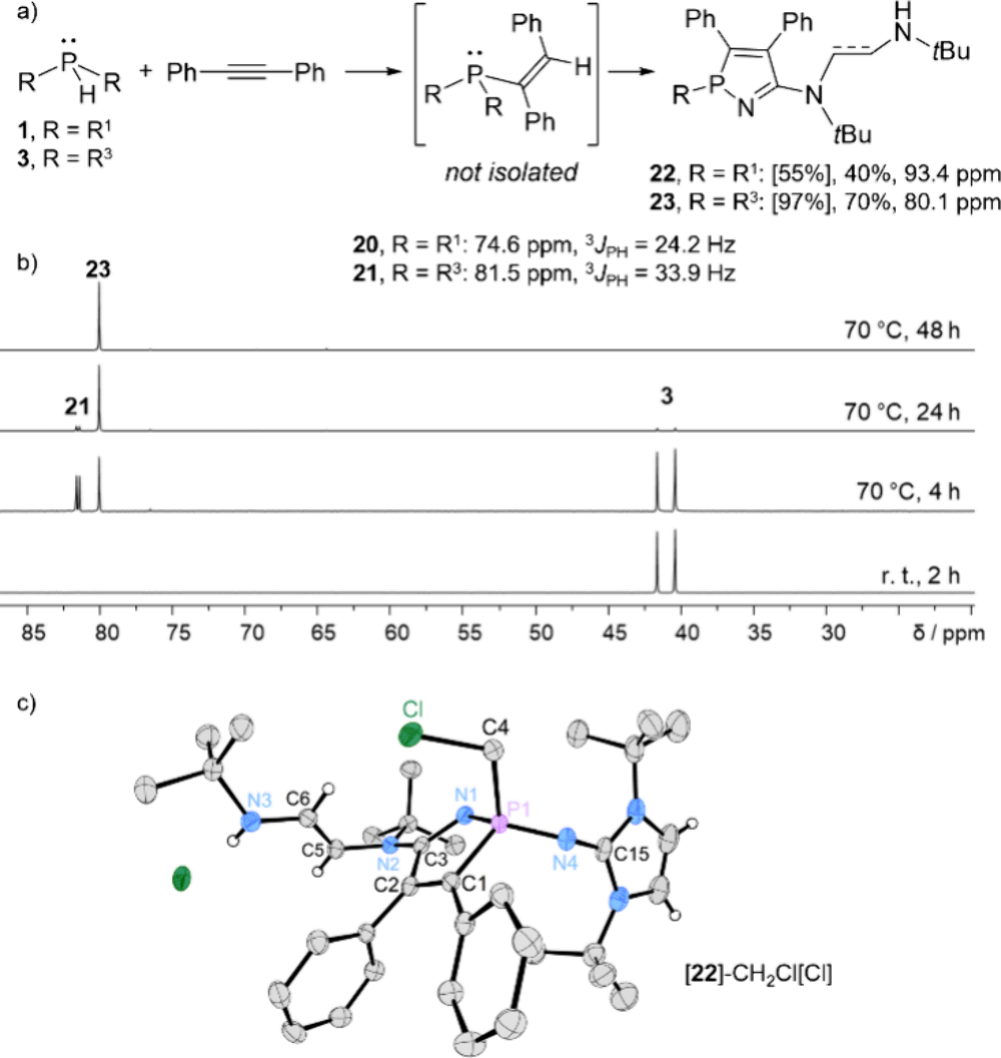
(a) Reaction of secondary IAPs **1** and **3** with diphenylacetylene yielding azaphospholes **22** and **23** via alkenylphosphines **20** and **21** [yields according to ^31^P NMR in brackets]. ^31^P NMR data measured in C_6_D_6_. (b) ^31^P NMR spectra (C_6_D_6_, 162 MHz) of the reaction mixture of **3** and diphenylacetylene. (c) Solid-state structure of [**20**-CH_2_Cl]­[Cl] (most hydrogen atoms are omitted for clarity; thermal ellipsoids are set at 50% probability; selected bond lengths (Å) and angles (°): P–N1 1.642(3), P–N4 1.558(3), P–C1 1.799(3), P–C4 1.806(3), N1–C3 1.311(4), C1–C2 1.346(4), C2–C3 1.506(4), C3–N2 1.343(4), N2–C5 1.423(4), C5–C6 1.341(5), C6–N3 1.352(4), N1–P–N4 118.9(2), N4–P–C1 115.6(2), N1–P–C1 97.54(12), C3–N1–P1 108.5(2), C1–C2–C3 111.5(3), C2–C3–N1 123.1(3)).

Overall, the intermolecular hydrophosphination of secondary phosphines **1** and **3** with nonactivated alkynes leads to the regioselective formation of anti-Markovnikov products and the preferential formation of the Z-isomer. This selectivity suggests a stepwise rather than concerted mechanism, involving nucleophilic addition of the phosphorus atom to the terminal carbon of the alkyne, forming a zwitterionic intermediate, followed by proton transfer. The resulting electron-rich alkenylphosphines undergo further reactions at ambient temperature, including an intramolecular cyclization to give azaphospholes. Deuteration experiments using (ethynyl-*d*)­benzene (Ph–≡–D) revealed the formation of all possible isotopomers in alkenyl positions of (Z)-**18** (with **1**) and a mixture of stereoisomers (Z) and (E) including isotopomers of **19** (with **3**). The introduction of deuterium atoms in all olefinic positions can be explained by participation of further phenylacetylene molecules in the proton shuttling processes.

## Conclusions

In this study, we report the synthesis and unique reactivity of secondary NHI-substituted phosphines (**1** **–** **3**), highlighting their potential and limitations as multifunctional reagents. These highly electron-rich secondary phosphines feature a polarized P–H bond, with electron density shifted toward the hydrogen atom. Computational studies reveal that they are excellent hydride donors while also exhibiting superbasic properties at the phosphorus atom. By selecting appropriate reaction partners, we demonstrated the versatility of the reactive P–H unit to transfer a hydride, proton, or hydrogen atom, as well as to act as a phosphorus nucleophile. Furthermore, the hydrophosphination of nonpolarized alkynes showcase their exceptional reactivity, resulting in the regioselective formation of anti-Markovnikov products and subsequent rearrangement into azaphosphole heterocycles. Notably, the steric bulk of the Dipp groups in phosphine **2** significantly limits the accessibility and reactivity of the P–H bond.

Overall, these findings emphasize the superior ability of NHI substituents to increase the electron abundance of phosphines and to control their reactivity by steric effects. However, the reactivity of the NHI groups themselves can introduce additional, and potentially undesired, reaction pathways, which must be carefully considered in potential applications.

## Experimental Section

### Synthesis of Secondary Imidazolidin2-yliden-aminophosphines **1–3**

The respective phosphenium chloride (1.0 equiv) was suspended in THF, and the mixture was cooled to −78 °C. Sodium tri*sec*-butyl­(hydrido)­borate (N-Selectride, 1.0 M in THF, 1.0 equiv) was added dropwise to the stirred suspension over 5 min using a syringe. The reaction mixture was then allowed to warm to ambient temperature over the course of three hours and stirred continuously for an additional 10 h. The solvent and all volatile components were removed *in vacuo* at 60 °C. The resulting residue was extracted, for the specific solvent and procedures of each compound see the Supporting Information.

**1:** Yield 64% (542 mg, 1.29 mmol). ^1^H NMR (400 MHz, C_6_D_6_): δ (ppm) = 8.42 (d, ^1^ *J*_PH_ = 179.6 Hz, 1H, PH), 6.15 (s, 4H, CH), 1.62 (s, 36H, CH_3_). ^13^C­{^1^H} NMR (101 MHz, C_6_D_6_): δ (ppm) = 143.0 (d, ^2^ *J*_PC_ = 22.4 Hz, C=N), 108.0 (C=C), 54.9 (*C*(CH_3_)_3_), 29.8 (d, ^5^ *J*_PC_ = 7.6 Hz, CH_3_). ^31^P NMR (162 MHz, C_6_D_6_): δ (ppm) = 57.7 (d, ^1^ *J*_PH_ = 179.6 Hz). ^31^P­{^1^H} NMR (162 MHz, C_6_D_6_): δ (ppm) = 57.7 (s). HRMS (ESI, positive): *m*/*z* calculated for [C_22_H_42_N_6_P]^+^ (1+H)^+^ 421.3203, found: 421.3205.

**2:** Yield 75% (720 mg, 0.86 mmol). ^1^H NMR (400 MHz, C_6_D_6_): δ (ppm) = 7.26 (t, ^3^ *J*_HH_ = 7.7 Hz, 4H, CH, H6), 7.05 (dm, ^3^ *J*_HH_ = 7.7 Hz, 8H, CH, H5), 5.78 (s, 4H, CH, H2), 5.72 (d, ^1^ *J*_PH_ = 184.3 Hz, 1H, PH), 3.11 (sept, ^3^ *J*_HH_ = 6.9 Hz, 4H, C*H*(CH_3_)_2_, H7), 3.00 (sept, ^3^ *J*_HH_ = 6.9 Hz, 4H, C*H*(CH_3_)_2_, H7), 1.21 (d, ^3^ *J*_HH_ = 6.9 Hz, 12H, CH_3_, H8), 1.19 (d, ^3^ *J*_HH_ = 6.9 Hz, 12H, CH_3_, H8), 1.17 (d, ^3^ *J*_HH_ = 6.9 Hz, 12H, CH_3_, H8), 1.12 (d, ^3^ *J*_HH_ = 6.9 Hz, 12H, CH_3_, H8). ^13^C­{^1^H} NMR (101 MHz, C_6_D_6_): δ (ppm) = 148.7 (d, ^5^ *J*_PC_ = 2.4 Hz, C_q_, C4), 148.1 (C_q_, C4), 141.3 (d, ^2^ *J*_PC_ = 17 Hz, C=N, C1), 134.7 (C_q_, C3), 129.0 (CH, C6), 123.5 (CH, C5), 123.2 (CH, C5), 114.0 (CH, C2), 29.0 (d, ^6^ *J*_PC_ = 1.6 Hz, *C*H­(CH_3_)_2_, C7), 28.9 (*C*H­(CH_3_)_2_, C7), 24.5 (*C*H_3_, C8), 24.2 (*C*H_3_, C8), 23.7 (*C*H_3_, C8), 23.6 (d, ^7^ *J*_PC_ = 5 Hz, *C*H_3_, C8). ^31^P NMR (162 MHz, C_6_D_6_): δ (ppm) = 45.4 (d, ^1^ *J*_PH_ = 184 Hz). ^31^P­{^1^H} NMR (162 MHz, C_6_D_6_): δ (ppm) = 45.4 (s). HRMS (ESI, positive): *m*/*z* calculated for [C_54_H_74_N_6_P]^+^ (2+H)^+^ 837.5707, found: 837.5695; *m*/*z* calculated for [C_54_H_75_N_6_OP]^+^ (2+OH)^+^ 853.5656, found: 853.5638. CHN analysis calculated (found) for [C_54_H_73_N_6_P]: C 77.47 (77.80) H 8.79 (8.91) N 10.04 (9.86).

**3:** Yield 79%. ^1^H NMR (400 MHz, C_6_D_6_): δ (ppm) = 8.07 (d, ^1^ *J*_PH_ = 199.0 Hz, 1H, PH), 2.84–2.80 (m, 8H, CH_2_), 1.45 (s, 36H, CH_3_). ^13^C­{^1^H} NMR (101 MHz, C_6_D_6_): δ (ppm) = 148.9 (d, ^2^ *J*_PC_ = 16.0 Hz, C=N), 53.3 (H_2_C–CH_2_), 42.1 (*C*(CH_3_)_3_), 28.6 (d, ^5^ *J*_PC_ = 6.9 Hz, CH_3_). ^31^P NMR (162 MHz, C_6_D_6_): δ (ppm) = 41.0 (d, ^1^ *J*_PH_ = 199.0 Hz). ^31^P­{^1^H} NMR (162 MHz, C_6_D_6_): δ (ppm) = 41.0 (s). HRMS (ESI, positive): *m*/*z* calculated for [C_22_H_46_N_6_P]^+^ (**3**+H)^+^ 425.3516, found: 425.3509.

## Supplementary Material



## Notes

Synthetic procedures, NMR spectra, mass spectrometry data, other characterization data, crystallographic data, and computational details (PDF)
