# Supplementary material for: Secondary Phosphines Bearing N‑Heterocyclic Imine Groups: Polarity Umpolung of Highly Electron-Rich P–H Bonds
Source: Inorg Chem. 2025 Nov 26;64(49):24134–42. doi: 10.1021/acs.inorgchem.5c04504 (PMC12709569; doi:10.1021/acs.inorgchem.5c04504)
Supplement: Supplementary file 1 [file ic5c04504_si_001.pdf]

Supplementary Material for

Secondary phosphines bearing N-heterocyclic imine groups: Polarity umpolung of highly electron-rich P–H bonds

Maike B. Röthel[a], Tobias Eder[a], Franka Brylak[a], Michael Seidl[a], Pawel Löwe[b], Fabian Dielmann[a]*

[a] Maike B. Röthel, Dr. Tobias Eder, Franka Brylak, Dr. Michael Seidl, Prof. Dr. Fabian Dielmann
Institute of General, Inorganic and Theoretical Chemistry,
Universität Innsbruck, 6020 Innsbruck (Austria)
E-mail: Fabian.Dielmann@uibk.ac.at

[b] Dr. Pawel Löwe
Institute of Inorganic and Analytical Chemistry,
Universität Münster, 48149 Münster (Germany)

CONTENT

1 Experimen tal Procedures 5

1.1 Synthetic Details 5

1.2 Synthesis of Bis(1,3-di-*tert*-butylimidazolin-2-ylidenamino)phosphine 1 6

1.3 Synthesis of Bis(1,3-(2,6-diisopropylphenyl)-imidazolin-2-ylidenamino)phosphine 2 8

1.4 Synthesis of Bis(1,3-di-*tert*-butylimidazolidin-2-ylidenamino)phosphine 3 12

1.5 Determination of Donor Strength 14

1.5.1 Reaction with $Ni(CO)_4$ – Tolman Electronic Parameter (TEP) 14

1.5.2 Reaction with Elemental Selenium – $^1J_{PSe}$ 21

1.5.3 Summary of the Experiments on Donor Properties 30

1.6 Oxidation of Secondary IAPs 1-3 with N_2O 30

1.6.1 Characterization data of 10 30

1.6.2 Characterization data of 11 33

1.6.3 Characterization data of 12 35

1.8 Synthesis of Gold Complexes 37

1.8.1 Characterization data of [13][SbF_6] ([1-Au-IDipp][SbF_6]) 37

1.8.2 Characterization data of [14][SbF_6] ([3-Au-IDipp][SbF_6]) 39

1.9 Synthesis of $(R^3)_2HPBH_3$ (15) 42

1.10 Reaction with TEMPO - Hydrogen Atom Transfer 45

1.10.1 EPR Experiments 45

1.10.2 NMR Experiments 49

1.11 Reactions with Trityl chloride – Hydride Transfer 53

1.12 Deprotonation Attempts 55

1.13 Reactions with Alkynes - Hydrophosphinations 60

1.13.1 Reaction with Phenylacetylene 60

1.13.2 Reaction with Diphenylacetylene 73

2 X-ray Crystallography 79

2.1 Crystal Structure Data of Compound 1 80

2.2 Crystal Structure Data of Compound 2 82

2.4 Crystal Structure Data of Compound 3 84

2.5 Crystal Structure Data of Compound 4 86

2.6 Crystal Structure Data of Compound 8 88

2.8 Crystal Structure Data of Compound [14][SbF_6] 90

2.9 Crystal Structure Data of Compound 15 92

2.10 Crystal Structure Data of Compound 16 94

2.11 Crystal Structure Data of Compound 18 96

2.12 Crystal Structure Data of Compound 19 98

2.13 Crystal Structure Data of Compound [22]-CH_2Cl[Cl] 100

3 Computational Details 102

3.1 Geometry Optimization and Natural Bond Orbital (NBO) Analysis 102

3.1.1 Asymmetric P–H Vibration 102

3.1.2 Depicted Frontier Orbitals 103

3.1.3 Natural Bond Orbital Analysis 107

3.1.4 Comparison of Different Functionals and Basis Sets 108

3.1.5 Atomic Coordinates 109

3.2 Determination of pK_a values 114

3.3 Determination of Hydride Ion Affinities 119

3.4 Determination of Bond Dissociation Free Energies (BDFEs) 123

4 References 127

1 Experimental Procedures

1.1 Synthetic Details

General Remarks: Unless otherwise noted, all manipulations were performed under an inert atmosphere of dry argon, using standard Schlenk and drybox techniques. Dry and oxygen-free solvents were employed. All glassware was oven-dried at 150 °C prior to use. ^{1}H, ^{11}B, ^{13}C, ^{19}F, ^{31}P and ^{77}Se NMR spectra were recorded on a 600 MHz Agilent DD2 spectrometer, 400 MHz Bruker AVANCE 4 Neo, Avance I, Avance III spectrometers and a 200 MHz Bruker AVANCE II spectrometer. Chemical shifts (δ) are given in parts per million (ppm) relative to $SiMe_4$ (^{1}H, ^{13}C), $BF_3{\cdot}Et_2O$ in $CDCl_3$ (^{11}B), 85% H_3PO_4 (^{31}P), $CFCl_3$ (^{19}F), Me_2Se (^{77}Se) and they were referenced to the residual solvent signals (C_6D_6: δ_H = 7.16, δ_C = 128.06; CD_3CN: δ_H = 1.94, δ_C = 118.26; CD_2Cl_2: δ_H = 5.32, δ_C = 54.00; all in ppm) or internally by the instrument after locking and shimming to the deuterated solvent (^{31}P, ^{19}F). NMR multiplicities are abbreviated as follows: s = singlet, d = doublet, t = triplet, q = quartet, p = pentet, sept = septet, m = multiplet, br. = broad signal. Mass spectrometry was performed using an Orbitrap LTQ XL (Thermo Scientific) spectrometer and an Orbitrap QExactive (Thermo Scientific) spectrometer. Elemental analysis was performed using a ‘vario MICRO cube’ elemental analyzer. IR spectroscopy was performed using a Bruker ALPHA FT-IR spectrometer. EPR measurements were performed on a Bruker Magnettech MS5000 X-band spectrometer equipped with a temperature control unit in 3 mm Teflon-sealed silica tubes. EPR spectra were simulated with the EasySpin v.6.0.10 toolbox running under MATLAB R2024b.

Reagents and Handling: Bis(1,3-di-*tert*-butyl-imidazolin-2-ylidenamino)phosphenium chloride[1], Bis(1,3-di-*tert*-butyl-imidazolidin-2-ylidenamino)phosphenium chloride[2], Bis(1,3-(2,6-diisopropylphenyl)-imidazolin-2-ylidenamino)phosphenium chloride[3], 1,3-Bis-(2,6-diisopropylphenyl)-imidazol-2-ylidengold(I)chloride[4] and (ethynyl-*d*)benzene[5] were prepared following literature procedures. All other compounds were purchased from commercial sources (Sigma Aldrich, Alfa Aesar, abcr GmbH, Chempur, J&K Scientific, Tokyo Chemical Industry, BLDpharm). Diphenylacetylene was sublimated before use.

1.2 Synthesis of Bis(1,3-di-*tert*-butylimidazolin-2-ylidenamino)phosphine 1

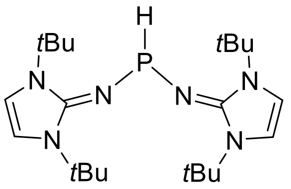

Bis(1,3-di-*tert*-butylimidazolin-2-ylidenamino)phosphenium chloride (910 mg, 2.00 mmol, 1.00 eq.) was suspended in THF (30 mL), and the mixture was cooled to -78 °C. Sodium tri-*sec*-butyl(hydrido)borate (N-Selectride®, 1.0 M in THF, 2.0 mL, 2.0 mmol, 1.0 eq.) was added dropwise to the stirred suspension over 5 minutes using a syringe. The reaction mixture was then allowed to warm to ambient temperature over the course of three hours and stirred continuously for an additional 10 hours. The solvent and all volatile components were removed *in vacuo* at 60 °C. The resulting residue was treated with toluene (30 mL) and heated at 60 °C with vigorous stirring for 15 minutes. The resulting suspension was filtered, and the filtrate was concentrated to a volume of 5 mL. Storage of the solution at -40 °C led to the crystallization of **1**, which was isolated as pale yellow, highly air-sensitive solid.

Brief contact with air or the storage of **1** in a glovebox containing small amounts of oxygen in the atmosphere led to an intense red coloration of the solid. Notably, the secondary phosphine can be extracted from slightly contaminated samples of this type with apolar solvents.

Yield 64% (542 mg, 1.29 mmol).
^{1}H NMR (400 MHz, C_6D_6): δ (ppm) = 8.42 (d, $^{1}J_{PH}$ = 179.6 Hz, 1H, PH), 6.15 (s, 4H, CH), 1.62 (s, 36H, CH_3).
^{13}C{^{1}H} NMR (101 MHz, C_6D_6): δ (ppm) = 143.0 (d, $^{2}J_{PC}$ = 22.4 Hz, C=N), 108.0 (C=C), 54.9 (*C*(CH_3)$_3$), 29.8 (d, $^{5}J_{PC}$ = 7.6 Hz, CH_3).
^{31}P NMR (162 MHz, C_6D_6): δ (ppm) = 57.7 (d, $^{1}J_{PH}$ = 179.6 Hz).
^{31}P{^{1}H} NMR (162 MHz, C_6D_6): δ (ppm) = 57.7 (s).
HRMS (ESI, positive): m/z calculated for $[C_{22}H_{42}N_6P]^+$ (**1**+H)$^+$ 421.3203, found: 421.3205.
Colorless crystals suitable for **SCXRD** were obtained from a saturated toluene solution stored at -40 °C.

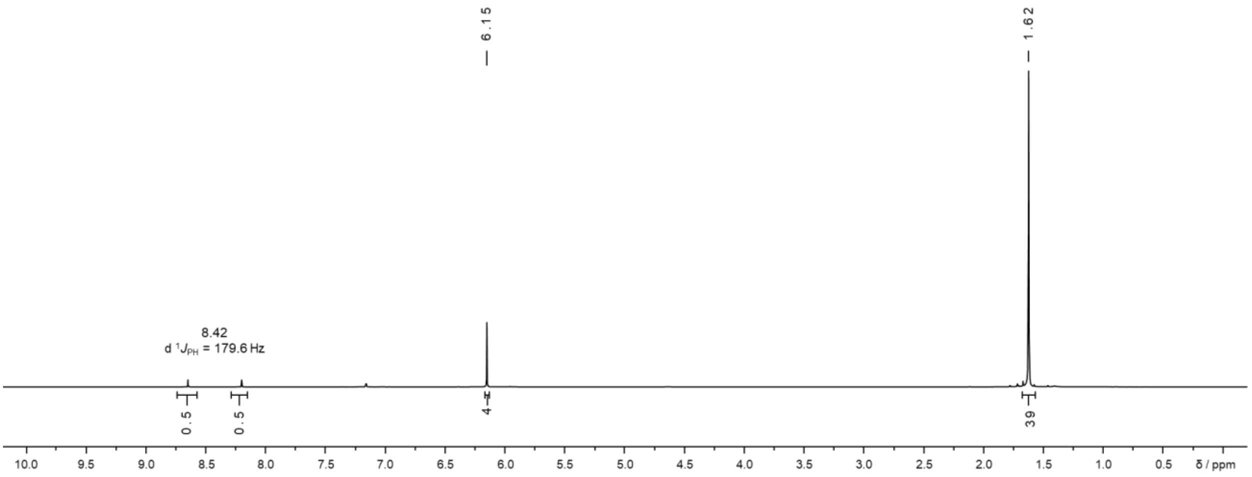

Figure S1: ^{1}H NMR spectrum (C_6D_6, 400 MHz) of **1**.

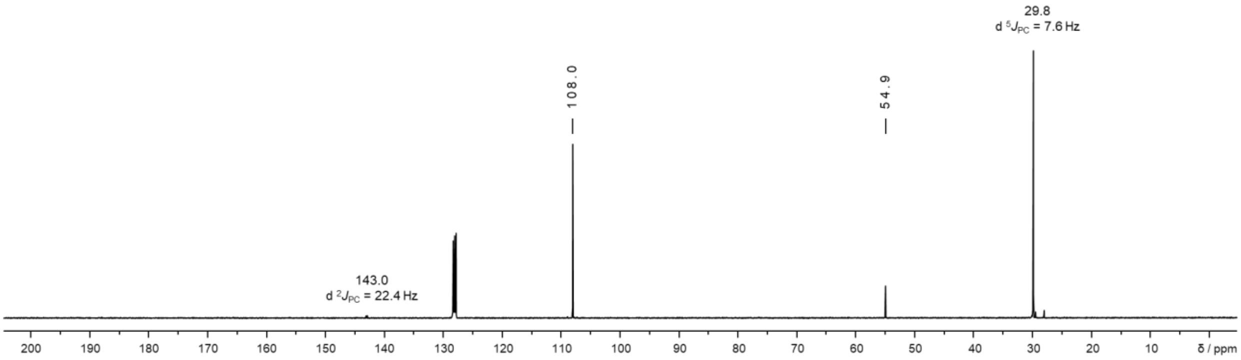

Figure S2: $^{13}C\{^1H\}$ NMR spectrum (C_6D_6, 101 MHz) of **1**.

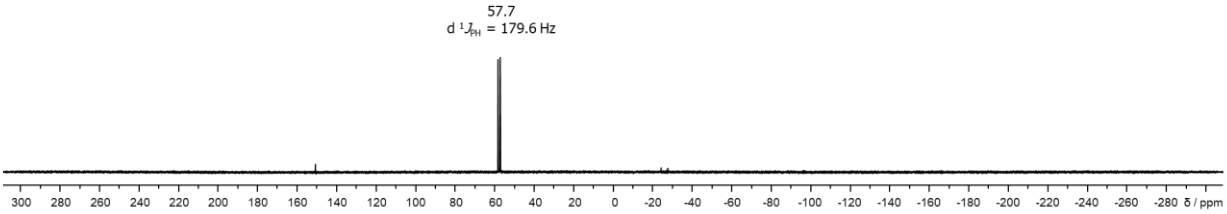

Figure S3: ^{31}P NMR spectrum (C_6D_6, 162 MHz) of **1**.

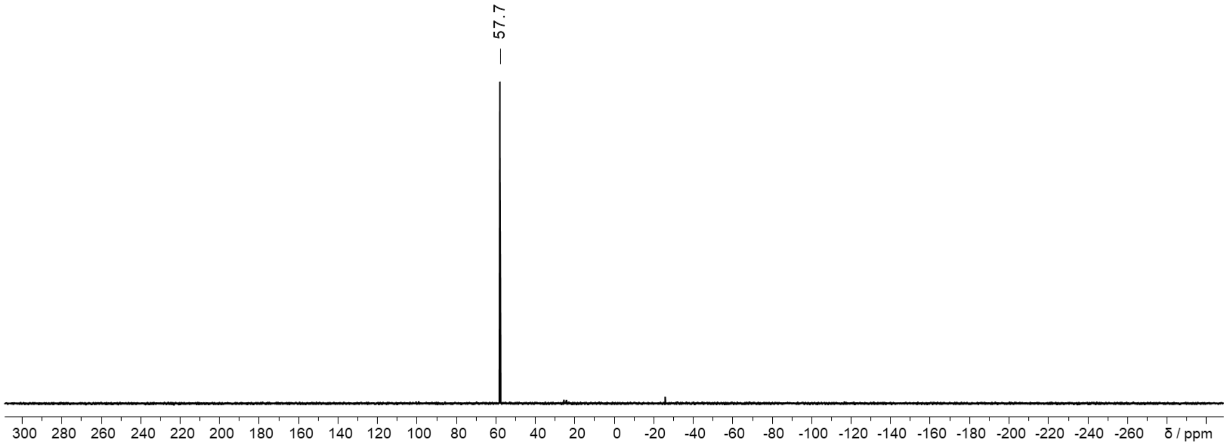

Figure S4: $^{31}P\{^1H\}$ NMR spectrum (C_6D_6, 162 MHz) of **1**.

1.3 Synthesis of Bis(1,3-(2,6-diisopropylphenyl)-imidazolin-2-ylidenamino)phosphine 2

Bis(1,3-(2,6-diisopropylphenyl)-imidazolin-2-ylidenamino)phosphenium chloride (1.00 g, 1.47 mmol, 1.00 eq.) was suspended in THF (30 mL), and the mixture was cooled to -78 °C. Sodium tri-*sec*-butyl(hydrido)borate (N-Selectride®, 1.0 M in THF, 1.47 mL, 1.47 mmol, 1.00 eq.) was added dropwise to the stirred suspension over 5 minutes using a syringe. The reaction mixture was then allowed to warm to ambient temperature over the course of three hours and stirred continuously for an additional 10 hours. The solvent and all volatile components were removed *in vacuo* at 60 °C. The residue was extracted with toluene (2 x 20 mL). After removal of the volatiles *in vacuo*, phosphine **2** was obtained as light-yellow solid.

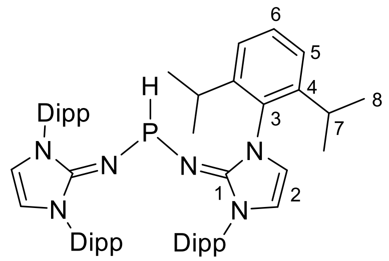

Notably, compound **2** can be recrystallized from *n*-hexane.

Yield 75% (720 mg, 0.86 mmol).

^{1}H NMR (400 MHz, C_6D_6): *δ* (ppm) = 7.26 (t, $^{3}J_{HH}$ = 7.7 Hz, 4H, CH, H6), 7.05 (dm, $^{3}J_{HH}$ = 7.7 Hz, 8H, CH, H5), 5.78 (s, 4H, CH, H2), 5.72 (d, $^{1}J_{PH}$ = 184.3 Hz, 1H, PH), 3.11 (sept, $^{3}J_{HH}$ = 6.9 Hz, 4H, C*H*(CH_3)$_2$, H7), 3.00 (sept, $^{3}J_{HH}$ = 6.9 Hz, 4H, C*H*(CH_3)$_2$, H7), 1.21 (d, $^{3}J_{HH}$ = 6.9 Hz, 12H, CH_3, H8), 1.19 (d, $^{3}J_{HH}$ = 6.9 Hz, 12H, CH_3, H8), 1.17 (d, $^{3}J_{HH}$ = 6.9 Hz, 12H, CH_3, H8), 1.12 (d, $^{3}J_{HH}$ = 6.9 Hz, 12H, CH_3, H8).

^{13}C{^{1}H} NMR (101 MHz, C_6D_6): *δ* (ppm) = 148.7 (d, $^{5}J_{PC}$ = 2.4 Hz, C_q, C4), 148.1 (C_q, C4), 141.3 (d, $^{2}J_{PC}$ = 17 Hz, C=N, C1), 134.7 (C_q, C3), 129.0 (CH, C6), 123.5 (CH, C5), 123.2 (CH, C5), 114.0 (CH, C2), 29.0 (d, $^{6}J_{PC}$ = 1.6 Hz, *C*H(CH_3)$_2$, C7), 28.9 (*C*H(CH_3)$_2$, C7), 24.5 (*C*H$_3$, C8), 24.2 (*C*H$_3$, C8), 23.7 (*C*H$_3$, C8), 23.6 (d, $^{7}J_{PC}$ = 5 Hz, *C*H$_3$, C8).

^{31}P NMR (162 MHz, C_6D_6): *δ* (ppm) = 45.4 (d, $^{1}J_{PH}$ = 184 Hz).

^{31}P{^{1}H} NMR (162 MHz, C_6D_6): *δ* (ppm) = 45.4 (s).

HRMS (ESI, positive): m/z calculated for $[C_{54}H_{74}N_6P]^+$ (**2**+H)$^+$ 837.5707, found: 837.5695; m/z calculated for $[C_{54}H_{75}N_6OP]^+$ (**2**+OH)$^+$ 853.5656, found: 853.5638.

CHN analysis calculated (found) for $[C_{54}H_{73}N_6P]$: C 77.47 (77.80) H 8.79 (8.91) N 10.04 (9.86).

Colorless crystals suitable for **SCXRD** were obtained from a saturated *n*-hexane solution stored at -40 °C.

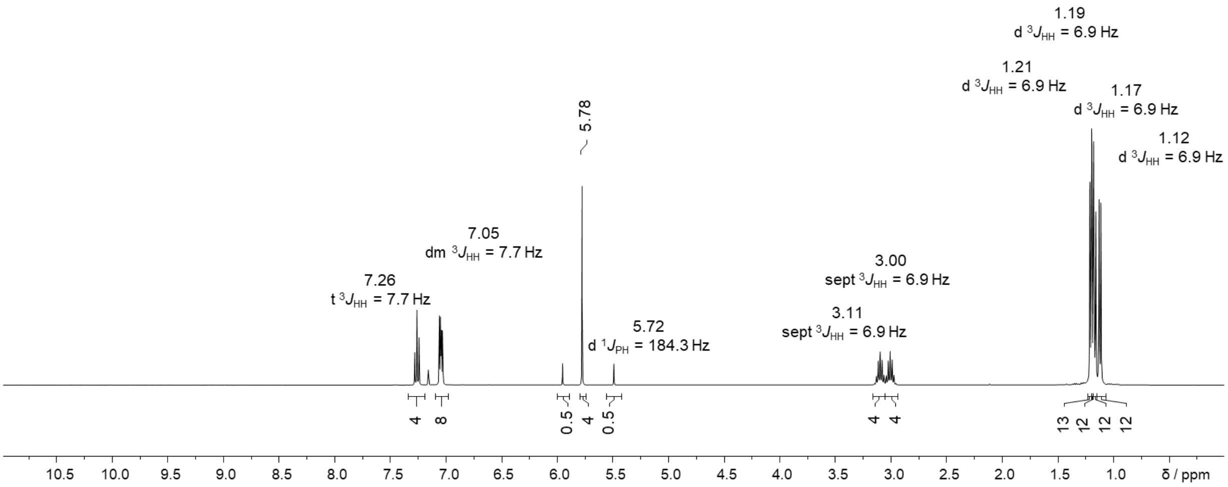

Figure S5: ^{1}H NMR spectrum (C_6D_6, 400 MHz) of **2**.

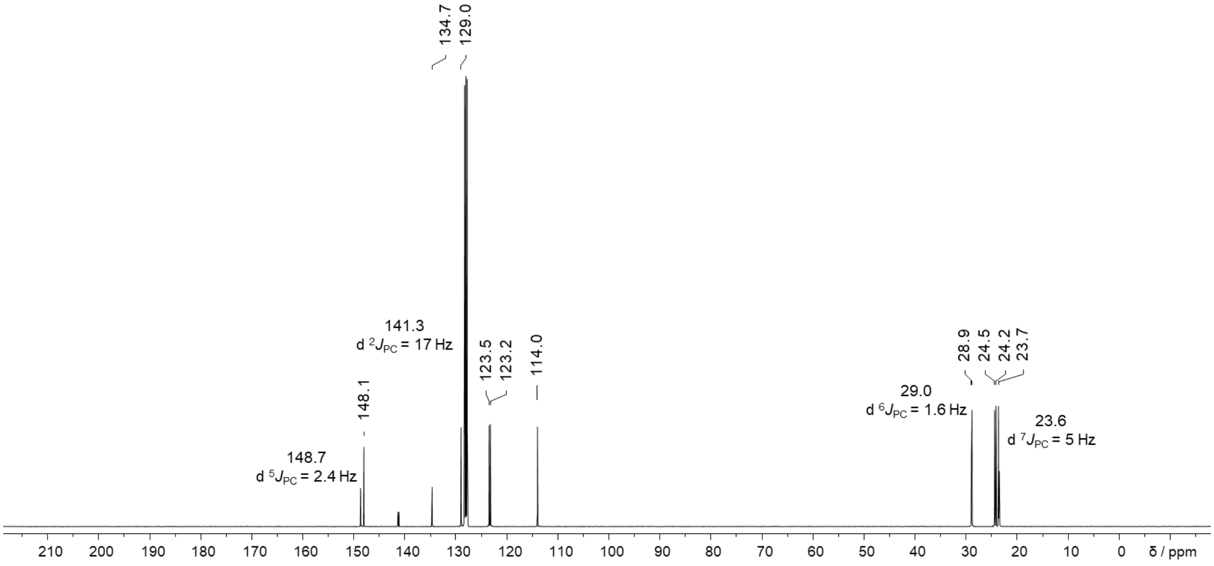

Figure S6: $^{13}C\{^1H\}$ NMR spectrum (C_6D_6, 101 MHz) of **2**.

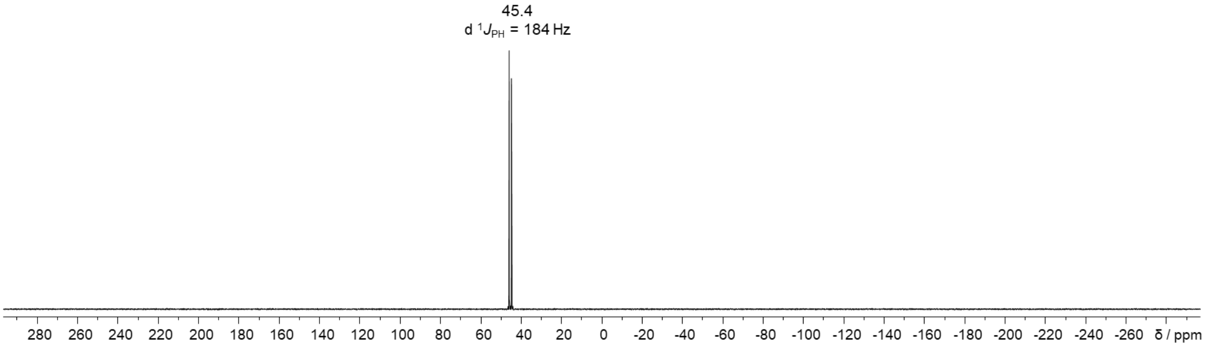

Figure S7: ^{31}P NMR spectrum (C_6D_6, 162 MHz) of **2**.

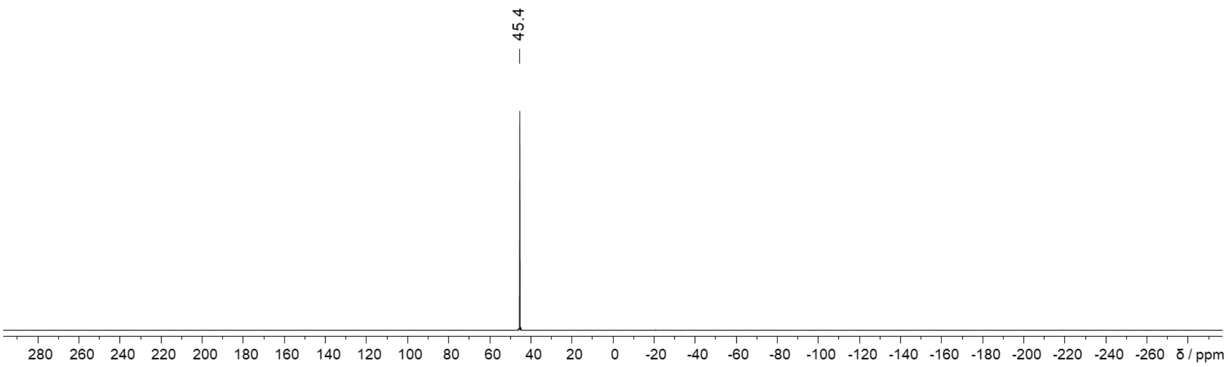

Figure S8: $^{31}P\{^1H\}$ NMR spectrum (C_6D_6, 162 MHz) of **2**.

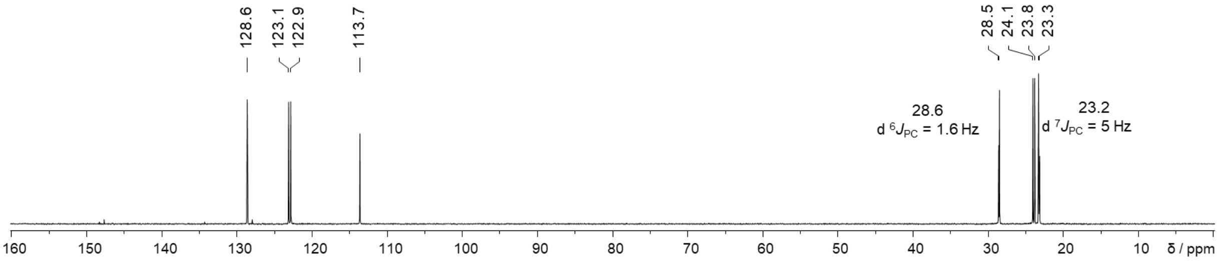

Figure S9: $^{13}C\{^1H\}$ DEPT135 NMR spectrum (C_6D_6, 101 MHz) of **2**.

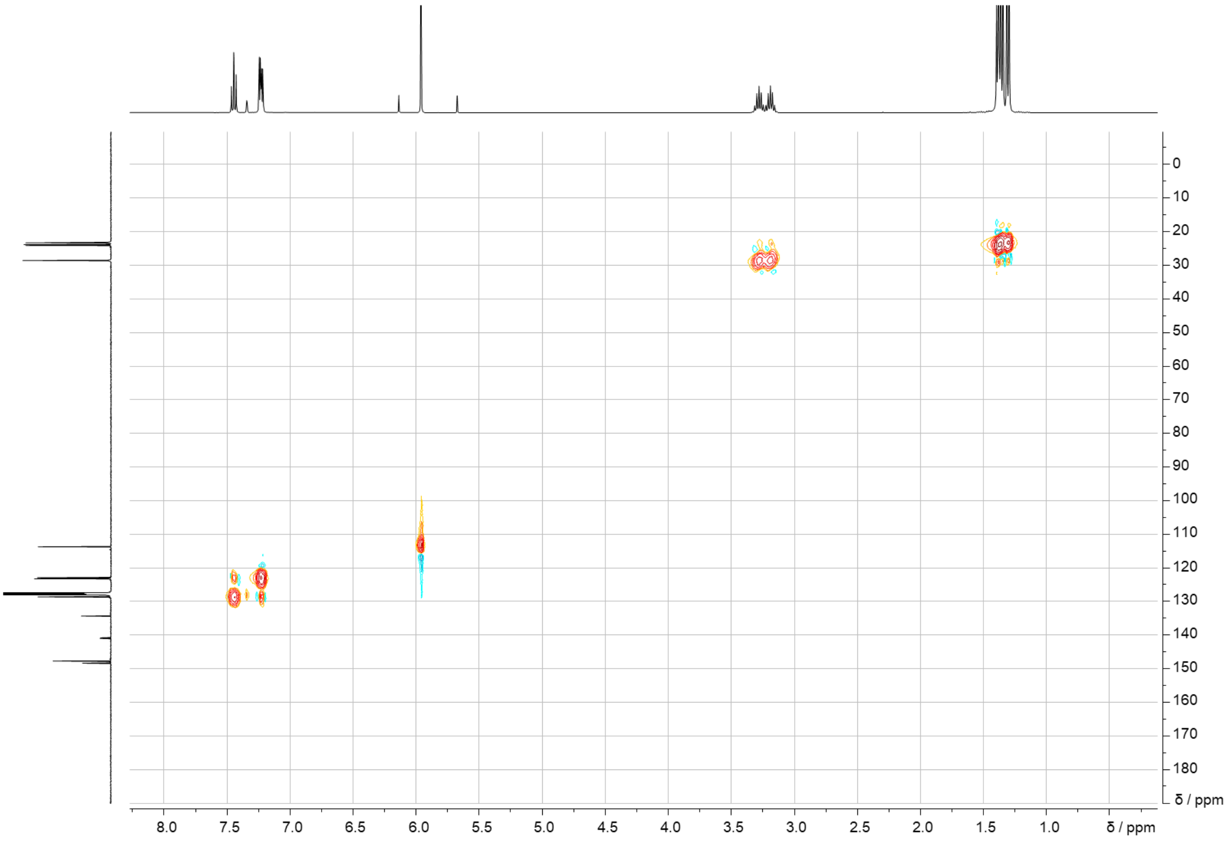

Figure S10: ^1H $^{13}C\{^1H\}$ HSQC 2D NMR spectrum (C_6D_6, 400 MHz, 101 MHz) of **2**.

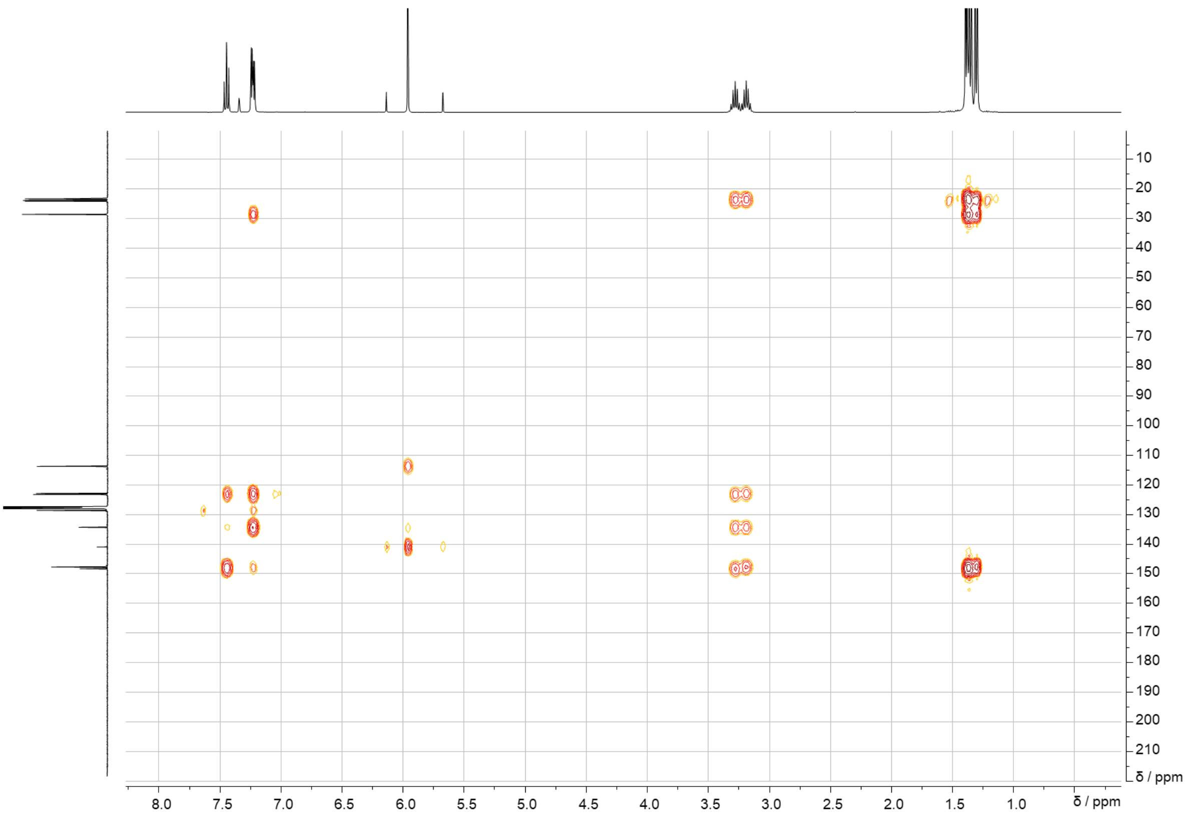

Figure S11: ^{1}H $^{13}C\{^{1}H\}$ HMBC 2D NMR spectrum (C_6D_6, 400 MHz, 101 MHz) of **2**.

1.4 Synthesis of Bis(1,3-di-*tert*-butylimidazolidin-2-ylidenamino)phosphine 3

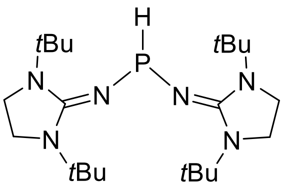

Bis(1,3-di-*tert*-butylimidazolidin-2-ylidenamino)phosphenium chloride (918 mg, 2.00 mmol, 1.00 eq.) was suspended in THF (30 mL), and the mixture was cooled to -78 °C. Sodium tri-*sec*-butyl(hydrido)borate (N-Selectride®, 1.0 M in THF, 2.0 mL, 2.0 mmol, 1.0 eq.) was added dropwise to the stirred suspension over 5 minutes using a syringe. The reaction mixture was then allowed to warm to ambient temperature over the course of three hours and stirred continuously for an additional 10 hours. The solvent and all volatile components were removed *in vacuo* at 60 °C. The residue was extracted with *n*-hexane (50 mL). After removal of the volatiles *in vacuo*, phosphine **3** was obtained as crystalline, highly air-sensitive, white solid.

Brief contact of **3** with air or the storage in a glovebox with small amounts of oxygen in the atmosphere led to an intense red coloration of the solid. The secondary phosphine can be separated by extraction of slightly contaminated samples of this type with apolar solvents.

Yield 79% (651 mg, 1.58 mmol).
^{1}H NMR (400 MHz, C_6D_6): δ (ppm) = 8.07 (d, $^1J_{PH}$ = 199.0 Hz, 1H, PH), 2.84-2.80 (m, 8H, CH_2), 1.45 (s, 36H, CH_3).
^{13}C{^{1}H} NMR (101 MHz, C_6D_6): δ (ppm) = 148.9 (d, $^2J_{PC}$ = 16.0 Hz, C=N), 53.3 (H_2C-CH_2), 42.1 (*C*(CH_3)$_3$), 28.6 (d, $^5J_{PC}$ = 6.9 Hz, CH_3).
^{31}P NMR (162 MHz, C_6D_6): δ (ppm) = 41.0 (d, $^1J_{PH}$ = 199.0 Hz).
^{31}P{^{1}H} NMR (162 MHz, C_6D_6): δ (ppm) = 41.0 (s).
HRMS (ESI, positive): m/z calculated for $[C_{22}H_{46}N_6P]^+$ (**3**+H)$^+$ 425.3516, found: 425.3509.
Colorless crystals suitable for **SCXRD** were obtained from a saturated *n*-hexane solution stored at -40 °C.

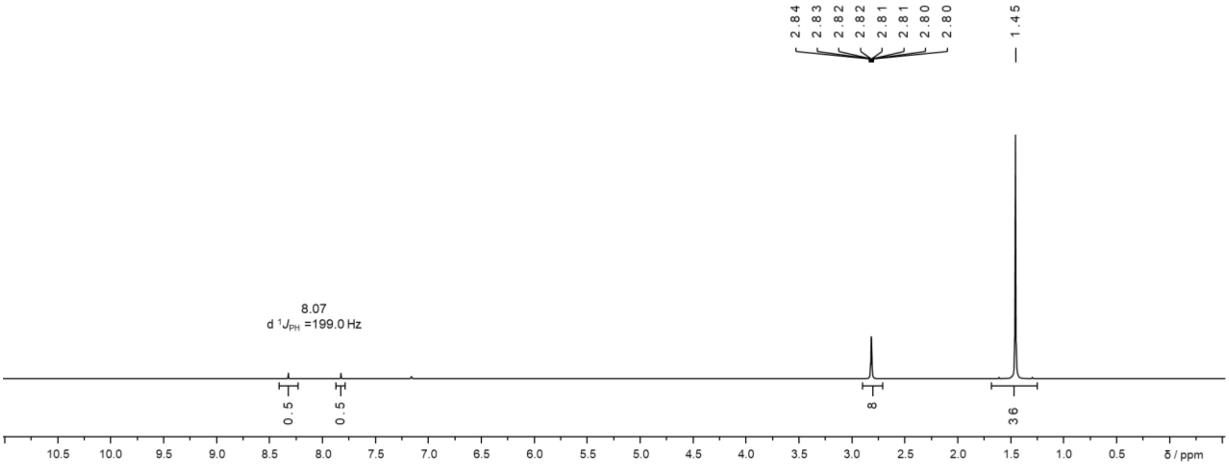

Figure S12: ^{1}H NMR spectrum (C_6D_6, 400 MHz) of **3**.

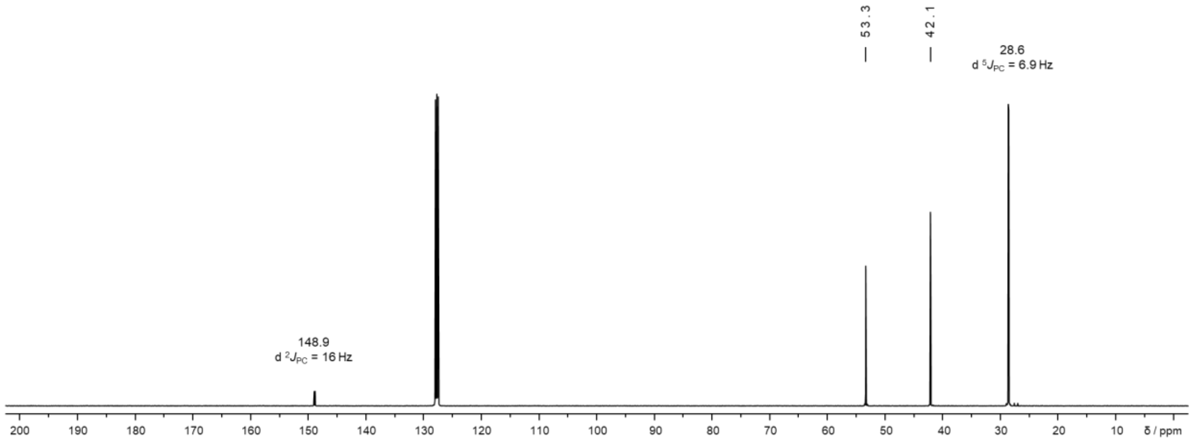

Figure S13: $^{13}C\{^1H\}$ NMR spectrum (C_6D_6, 101 MHz) of **3**.

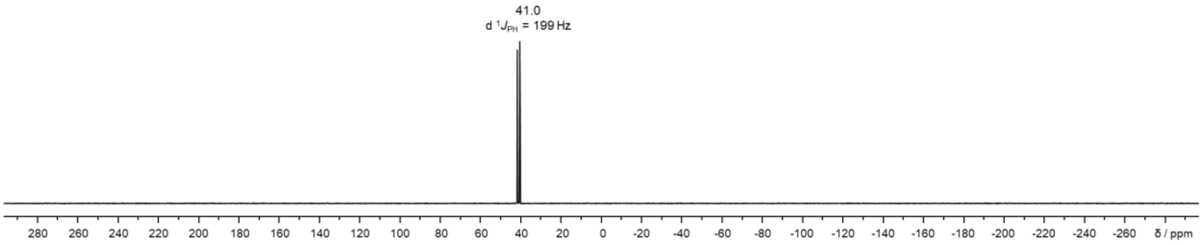

Figure S14: ^{31}P NMR spectrum (C_6D_6, 162 MHz) of **3**.

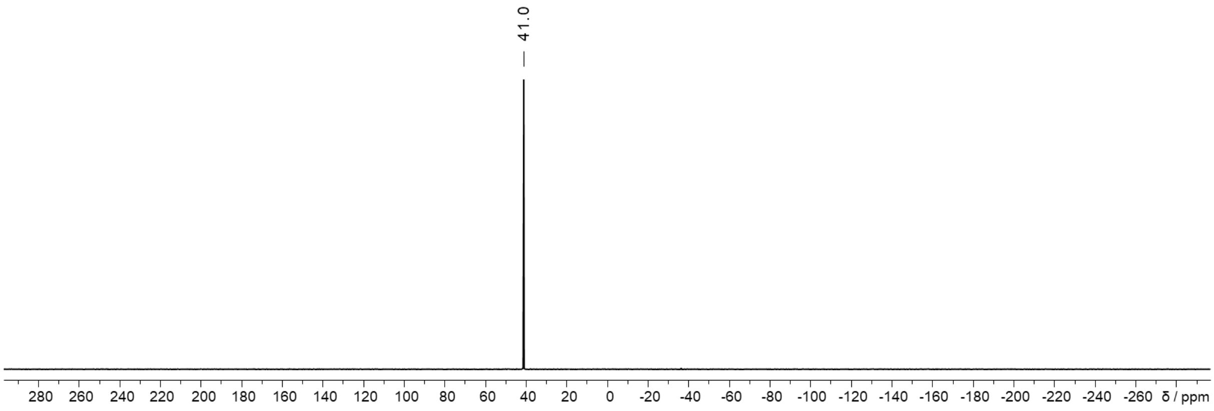

Figure S15: $^{31}P\{^1H\}$ NMR spectrum (C_6D_6, 162 MHz) of **3**.

1.5 Determination of Donor Strength

1.5.1 Reaction with $Ni(CO)_4$ – Tolman Electronic Parameter (TEP)

The secondary phosphine **1**, **2** or **3** (**1**: 25.2 mg, **2**: 50.2 mg, **3**: 25.5 mg, 0.0600 mmol, 1.00 eq.) was dissolved in toluene (1 mL) and tetracarbonylnickel (nickel tetracarbonyl, $Ni(CO)_4$, 0.138 M in hexane, 0.50 mL, 0.070 mmol, 1.15 eq.) was added. After stirring for 60 minutes all volatiles were removed *in vacuo*. The resulting solid was analyzed by ^{31}P NMR spectroscopy and IR spectroscopy.

The reaction between **2** and $Ni(CO)_4$ showed a conversion of ~85% to $[(CO)_3NiP(R^2)_2H]$ (**5**), the other phosphines (**1** and **3**) reacted quantitatively. $[(CO)_3NiP(R^1)_2H]$ (**4**) and $[(CO)_3NiP(R^3)_2H]$ (**6**) were additionally characterized by ^1H NMR, ^{13}C NMR spectroscopy and high-resolution mass spectrometry (HRMS).

1.5.1.1 Characterization data of **4**

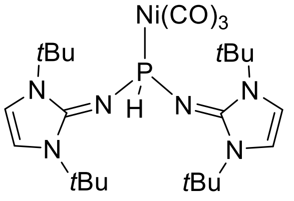

^1H NMR (400 MHz, C_6D_6): δ (ppm) = 9.04 (d, $^1J_{PH}$ = 294.8 Hz, 1H, PH), 6.13 (s, 4H, CH), 1.58 (s, 36H, CH_3).

$^{13}C\{^1H\}$ NMR (101 MHz, C_6D_6): δ (ppm) = 200.0 (d, $^2J_{PC}$ = 3 Hz, CO),143.4 (d, $^2J_{PC}$ = 11 Hz, C=N), 109.0 (C=C), 55.9 ($C(CH_3)_3$), 29.8 (d, $^5J_{PC}$ = 2 Hz, CH_3).

^{31}P NMR (162 MHz, C_6D_6): δ (ppm) = 50.2 (d, $^1J_{PH}$ = 295 Hz).

$^{31}P\{^1H\}$ NMR (162 MHz, C_6D_6): δ (ppm) = 50.2 (s).

HRMS (ESI, positive): m/z calculated for $[C_{25}H_{42}N_6NiO_3P]^+$ (**4**+H)$^+$ 563.2404, found: 563.2399.

IR (DCM): $\tilde{\nu}$ (cm^{-1}) = 2043.10 (A_1 C–O).

IR (neat): $\tilde{\nu}$ (cm^{-1}) = 2037.31 (A_1 C–O).

Crystals suitable for **SCXRD** were obtained from storing a saturated toluene solution at -40 °C.

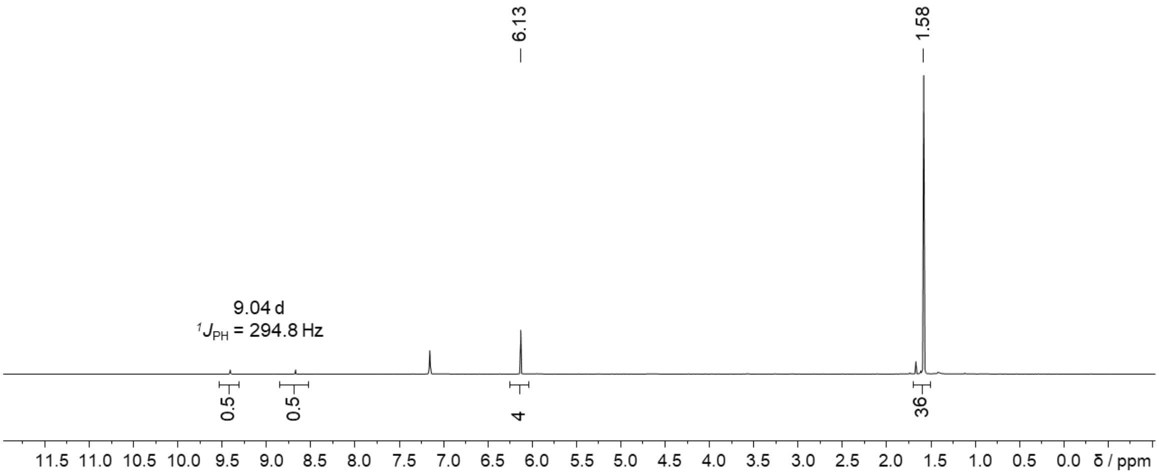

Figure S16: ^1H NMR spectrum (C_6D_6, 400 MHz) of **4**.

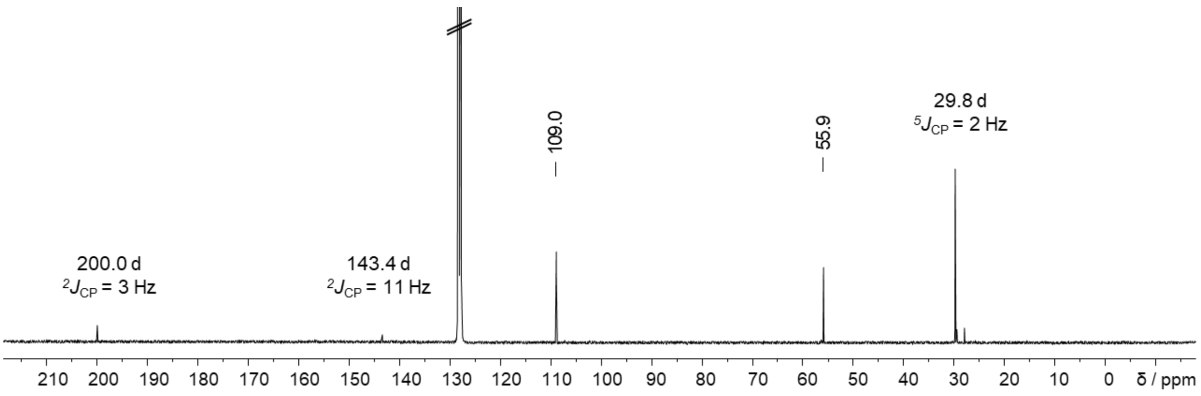

Figure S17: $^{13}C\{^1H\}$ NMR spectrum (C_6D_6, 101 MHz) of **4**.

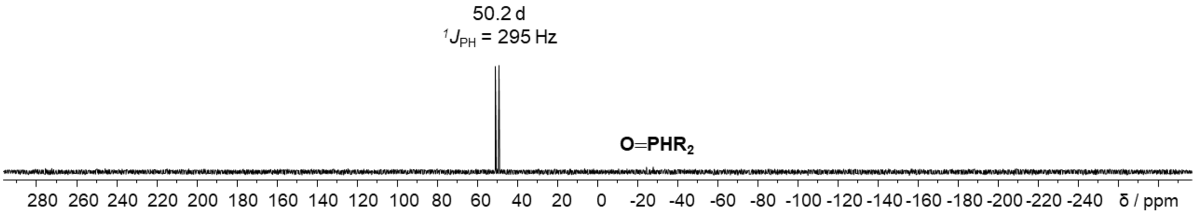

Figure S18: ^{31}P NMR spectrum (C_6D_6, 162 MHz) of **4**.

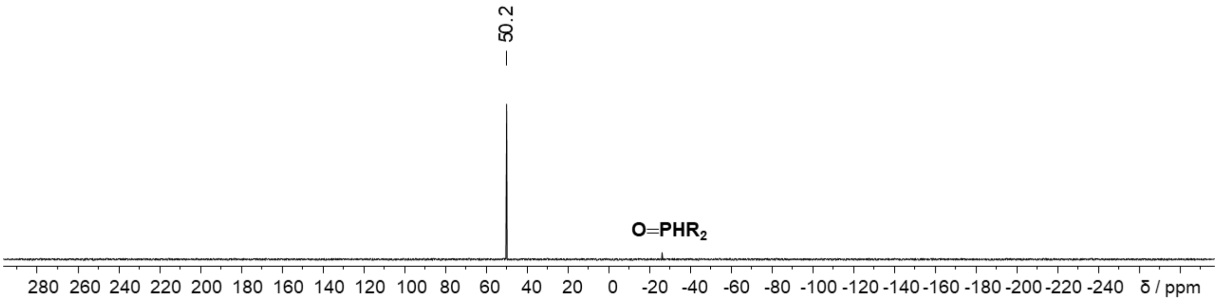

Figure S19: $^{31}P\{^1H\}$ NMR spectrum (C_6D_6, 162 MHz) of **4**.

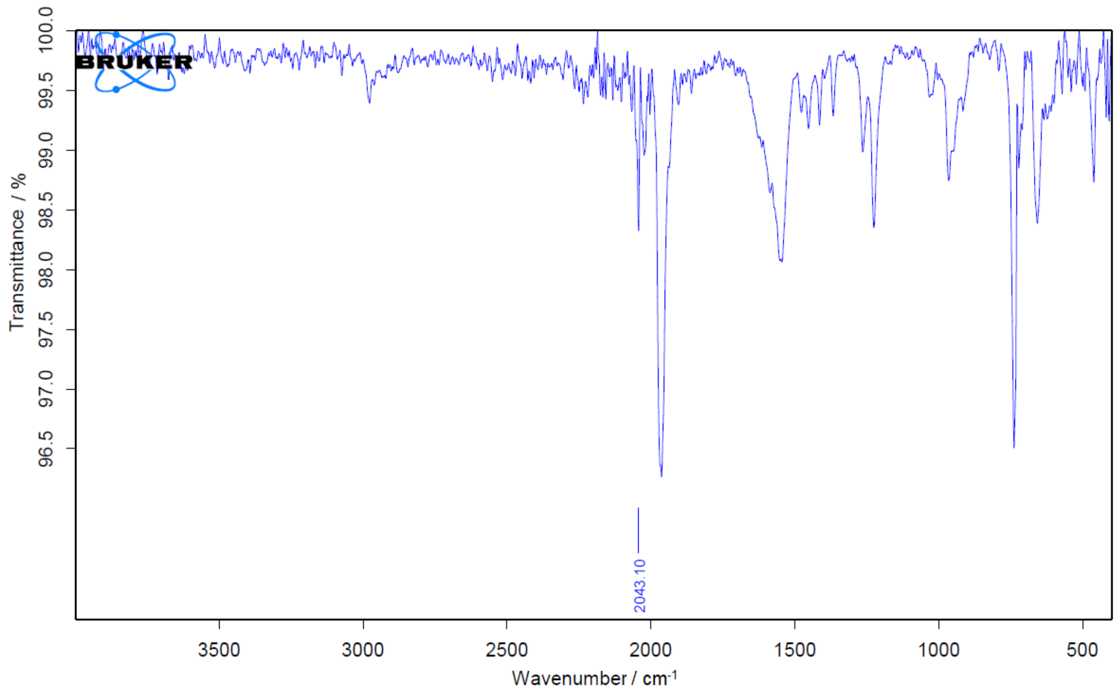

Figure S20: IR spectrum of **4** in DCM.

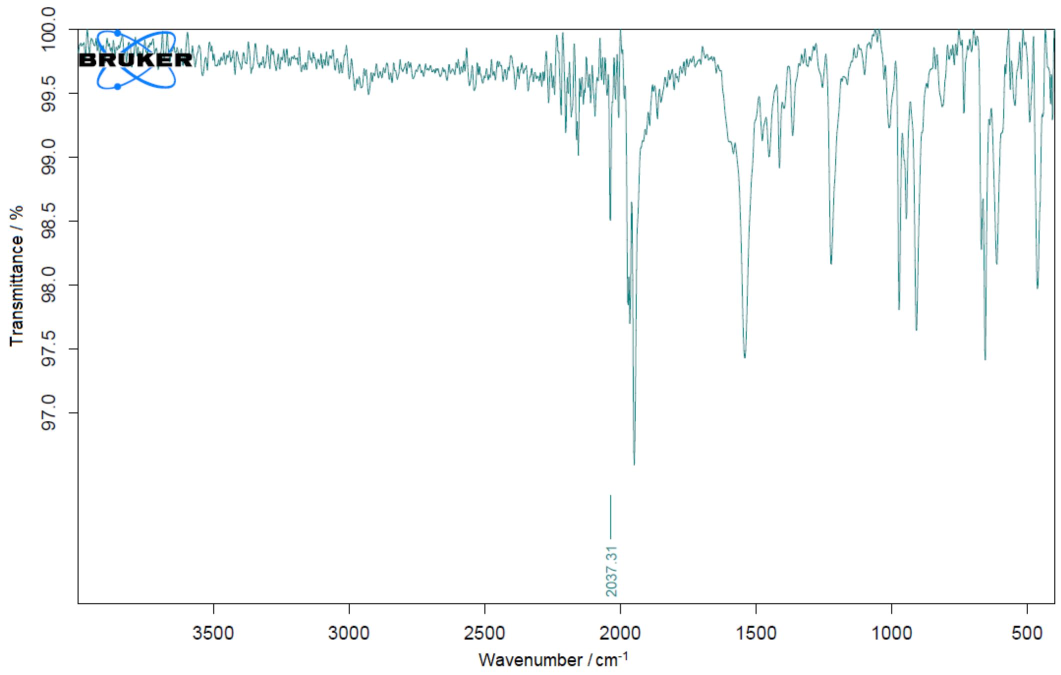

Figure S21: IR spectrum of **4**, neat.

1.5.1.2 Characterization data of **5**

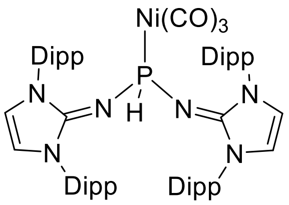

^{31}P NMR (162 MHz, C_6D_6): δ (ppm) = 38.3 (d, $^{1}J_{PH}$ = 319 Hz).

^{31}P{^{1}H} NMR (162 MHz, C_6D_6): δ (ppm) = 38.3 (s).

IR (DCM): $\tilde{\nu}$ (cm^{-1}) = 2048.71 (A_1 C–O).

IR (neat): $\tilde{\nu}$ (cm^{-1}) = 2046.51 (A_1 C–O).

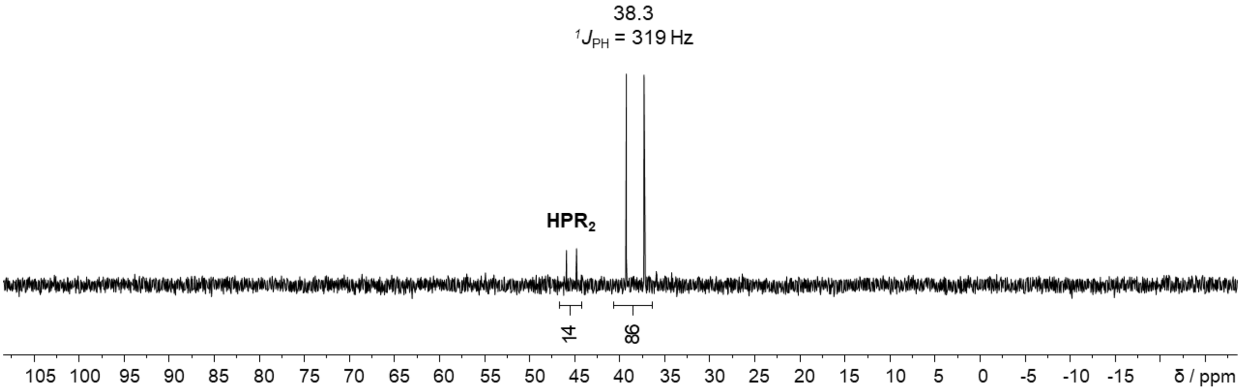

Figure S22: ^{31}P NMR spectrum (C_6D_6, 162 MHz) of **5** and non-reacted **2**.

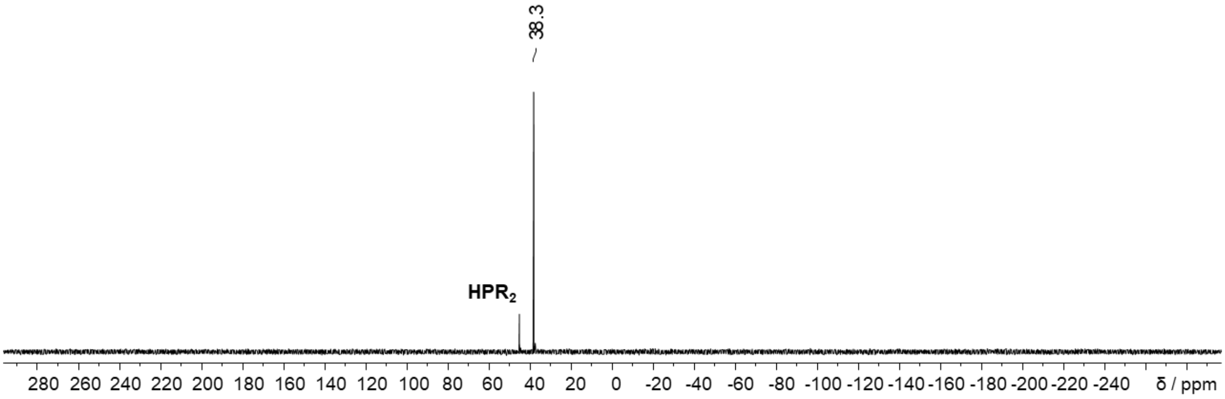

Figure S23: $^{31}P\{^1H\}$ NMR spectrum (C_6D_6, 162 MHz) of **5** and non-reacted **2**.

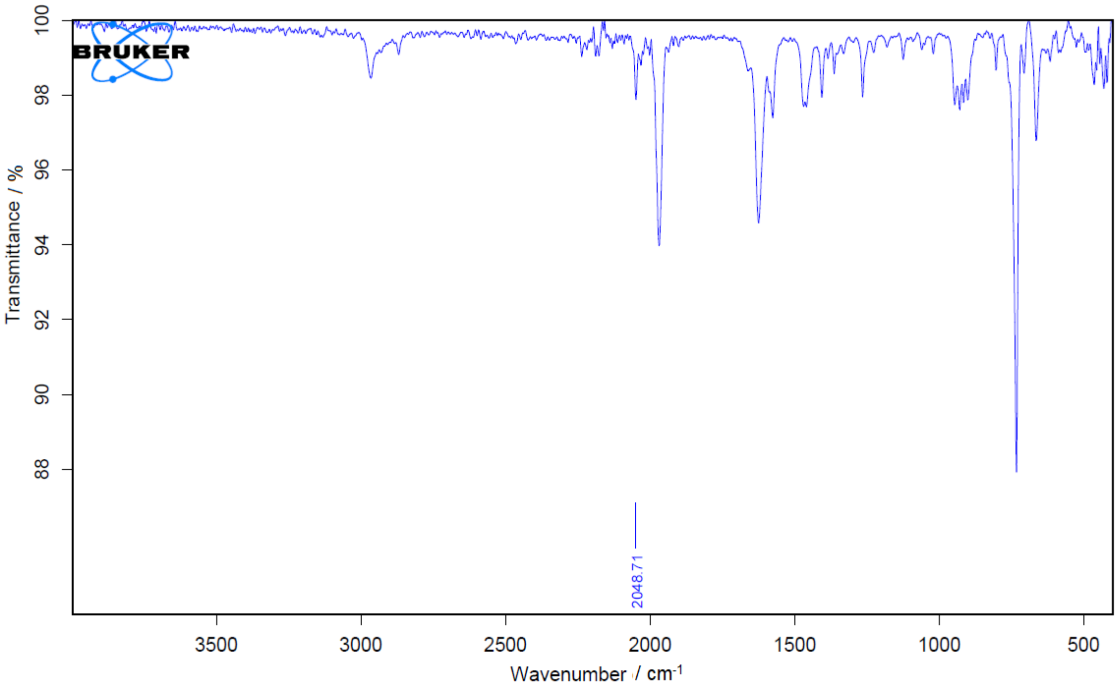

Figure S24: IR spectrum of **5** (and non-reacted **2**) in DCM.

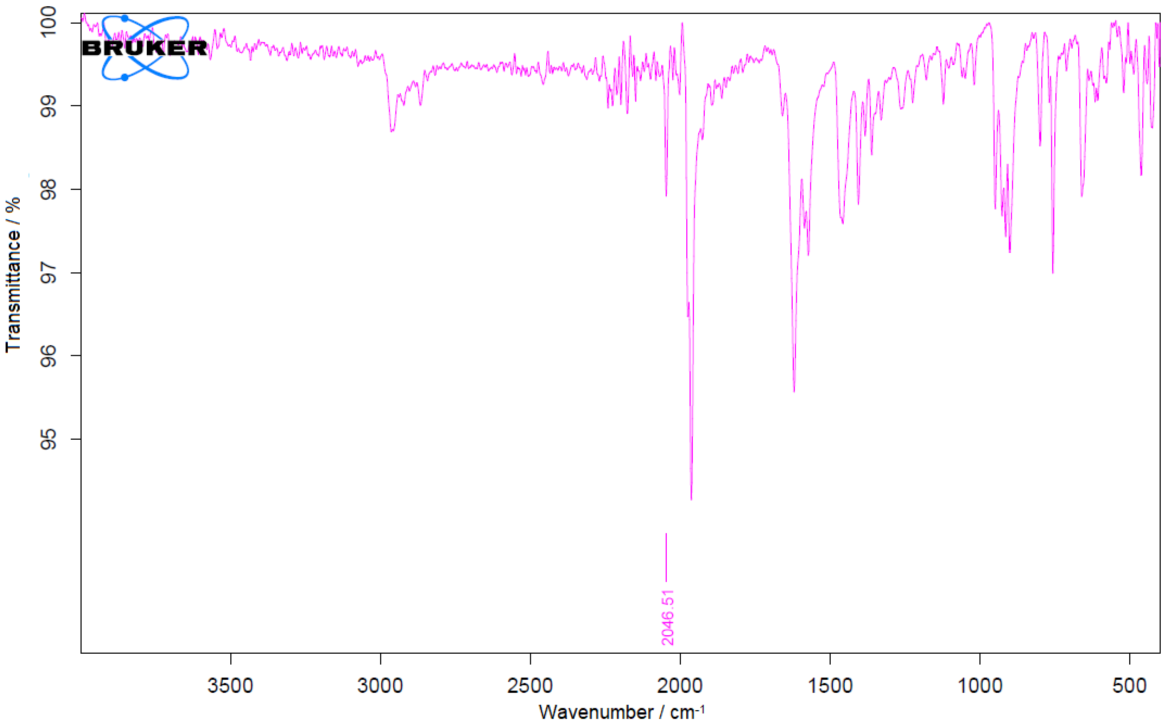

Figure S25: IR spectrum of **5** (and non-reacted **2**), neat.

1.5.1.3 Characterization data of **6**

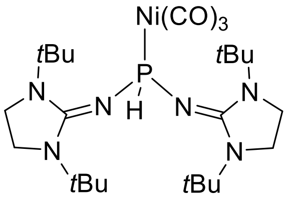

^{1}H NMR (400 MHz, C_6D_6): δ (ppm) = 8.84 (d, $^{1}J_{PH}$ = 319.1 Hz, 1H, PH), 2.73 (s, 8H, CH_2), 1.40 (s, 36H, CH_3).

^{13}C{^{1}H} NMR (101 MHz, C_6D_6): δ (ppm) = 199.5 (d, $^{2}J_{PC}$ = 3 Hz, CO),151.5 (d, $^{2}J_{PC}$ = 8 Hz, C=N), 54.6 (H_2C–CH_2), 42.5 (*C*(CH_3)$_3$), 28.8 (d, $^{5}J_{PC}$ = 1 Hz, CH_3).

^{31}P NMR (162 MHz, C_6D_6): δ (ppm) = 47.7 (d, $^{1}J_{PH}$ = 319 Hz).

^{31}P{^{1}H} NMR (162 MHz, C_6D_6): δ (ppm) = 47.7 (s).

HRMS (ESI, positive): m/z calculated for $[C_{25}H_{46}N_6NiO_3P]^+$ (**6**+H)$^+$ 567.2717, found: 567.2709.

IR (DCM): $\tilde{\nu}$ (cm^{-1}) = 2051.51 (A_1 C–O).

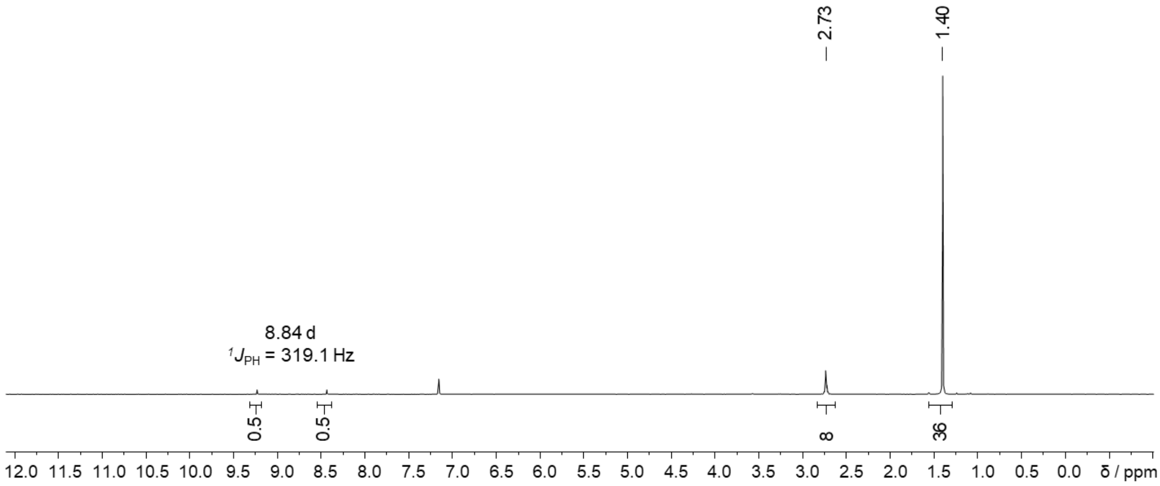

Figure S26: ^{1}H NMR spectrum (C_6D_6, 400 MHz) of **6**.

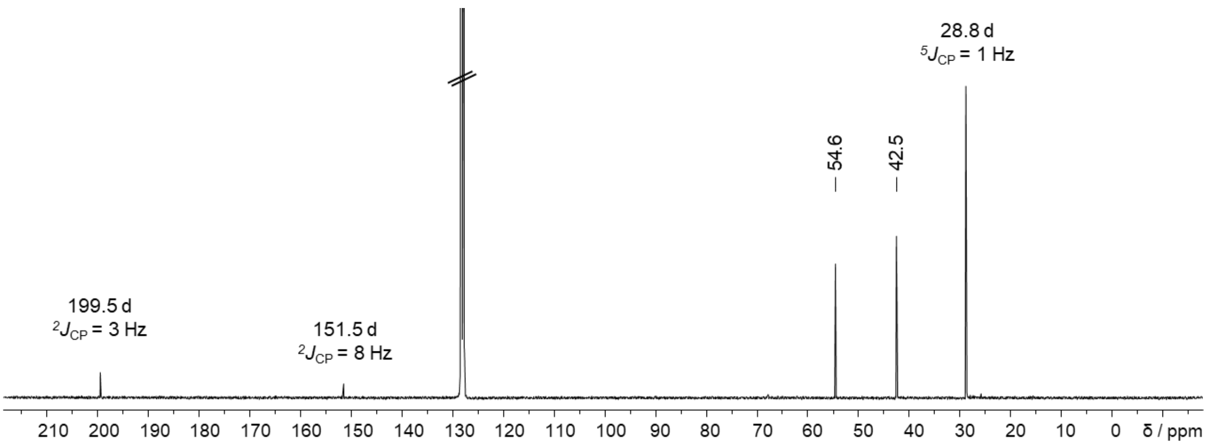

Figure S27: $^{13}C\{^1H\}$ NMR spectrum (C_6D_6, 101 MHz) of **6**.

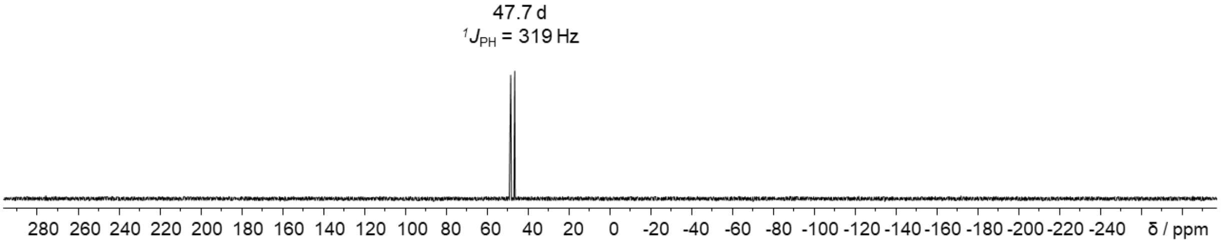

Figure S28: ^{31}P NMR spectrum (C_6D_6, 162 MHz) of **6**.

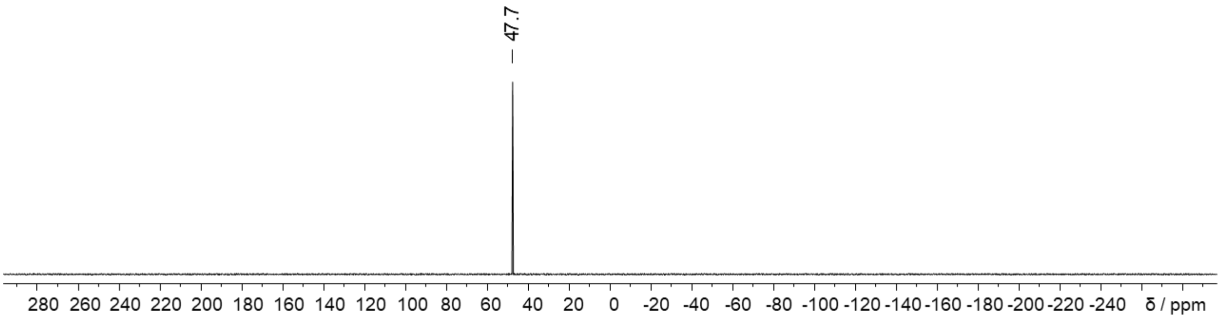

Figure S29: $^{31}P\{^1H\}$ NMR spectrum (C_6D_6, 162 MHz) of **6**.

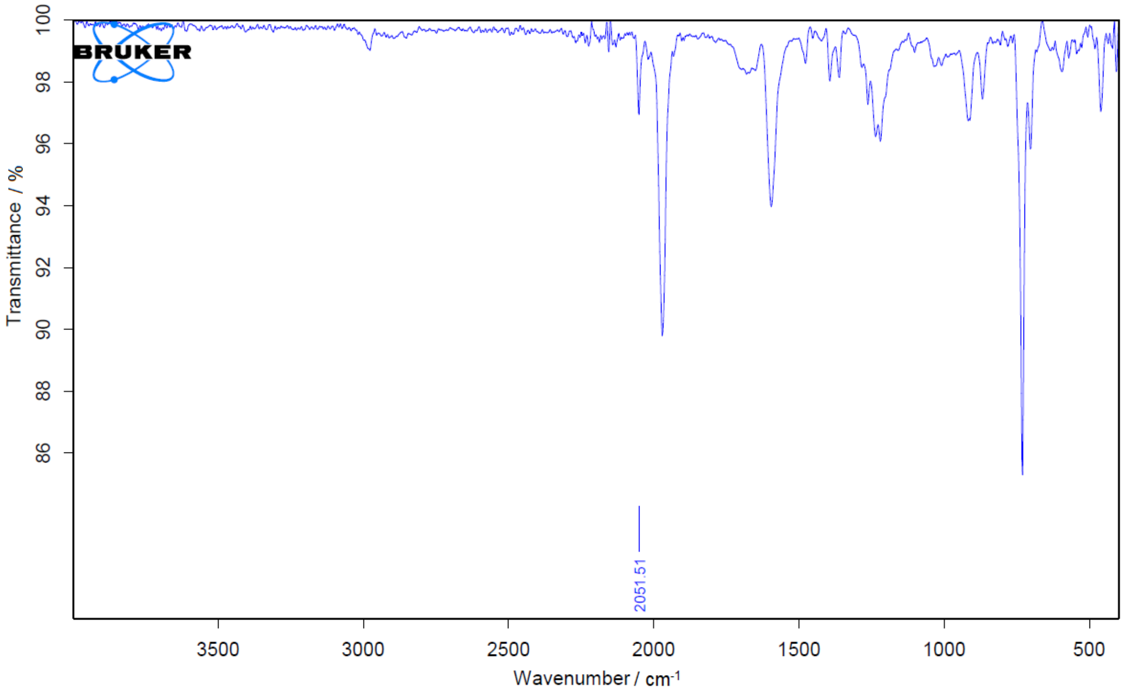

Figure S30: IR spectrum of **6** in DCM.

1.5.2 Reaction with Elemental Selenium – $^{1}J_{PSe}$

1.5.2.1 Phosphine Selenide $(R^1)_2HPSe$ (**7**)

To compound **1** (50 mg, 0.12 mmol, 1.0 eq.) and a stoichiometric excess of grey selenium (15 mg, 0.19 mmol, 1.6 eq.), THF (2 mL) was added. The mixture was stirred for 16 hours at ambient temperature. After filtration and removal of the THF *in vacuo*, the resulting solid was dissolved in CD_2Cl_2, and the solution was analyzed by NMR spectroscopy.

^{1}H NMR (400 MHz, CD_2Cl_2): δ (ppm) = 9.27 (d, $^{1}J_{PH}$ = 504.0 Hz, 1H, PH), 6.61 (s, 4H, CH), 1.71 (s, 36H, CH_3).

^{31}P NMR (162 MHz, CD_2Cl_2): δ (ppm) = -14.7 (d, $^{1}J_{PH}$ = 504 Hz, $^{1}J_{PSe}$ = 638 Hz).

^{31}P{^{1}H} NMR (162 MHz, CD_2Cl_2): δ (ppm) = -14.7 (s, $^{1}J_{PSe}$ = 638 Hz).

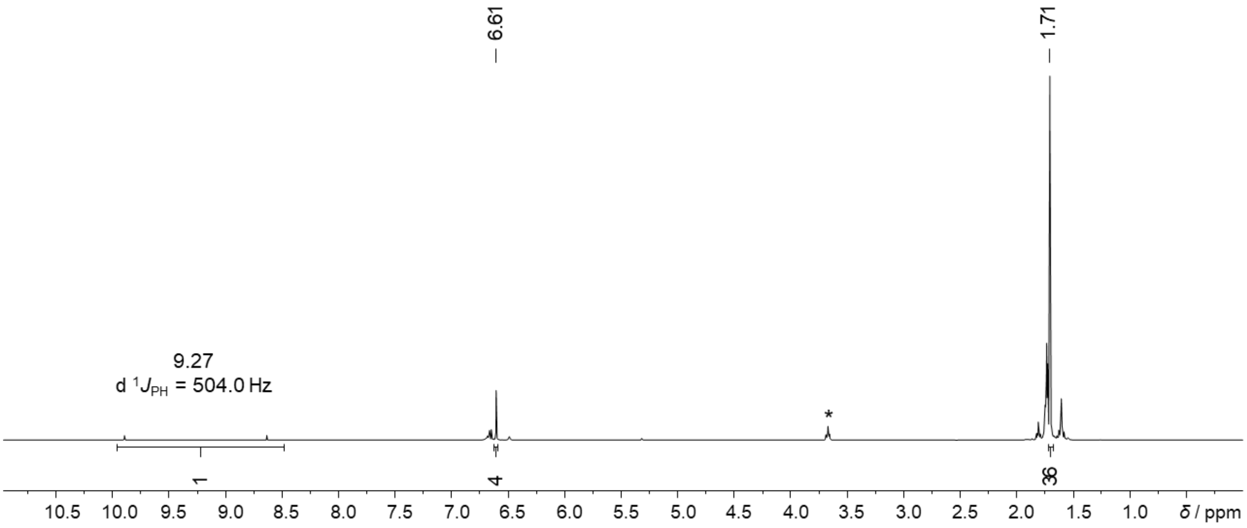

Figure S31: ^{1}H NMR spectrum (CD_2Cl_2, 400 MHz) of the reaction mixture containing **7**, asterisk (*) marks residual THF.

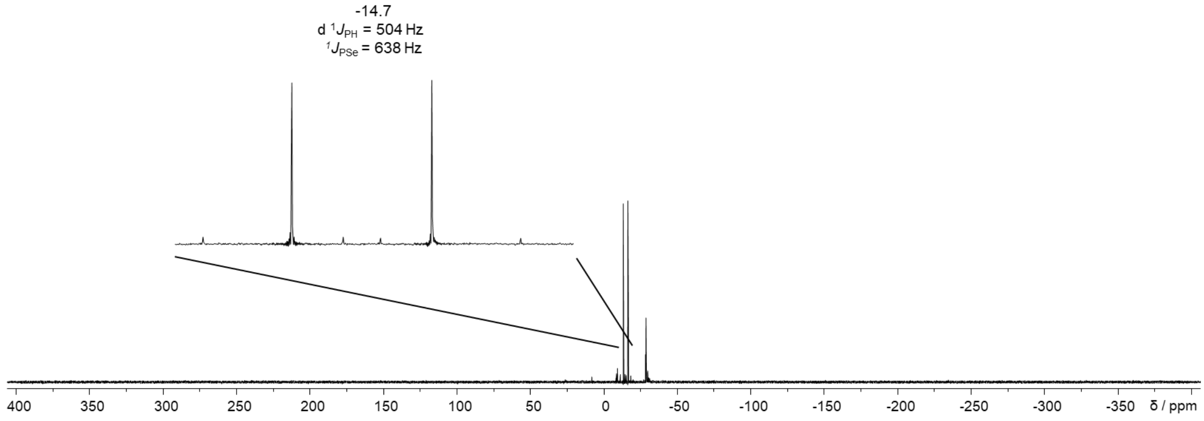

Figure S32: ^{31}P NMR spectrum (CD_2Cl_2, 162 MHz) of the reaction mixture containing **7**.

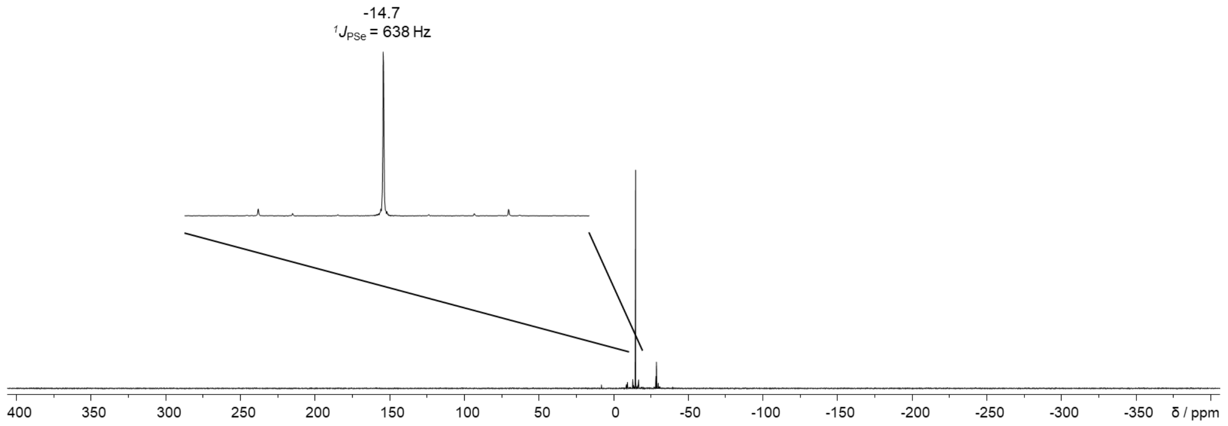

Figure S33: $^{31}P\{^{1}H\}$ NMR spectrum (CD_2Cl_2, 162 MHz) of the reaction mixture containing **7**.

An NMR tube equipped with a PTFE valve was charged with C_6D_6 (0.7 mL), compound **1** (25 mg, 0.059 mmol, 1.0 eq.), and a stoichiometric excess of grey selenium (10 mg, 0.13 mmol, 2.2 eq.). After heating to 50 °C for 90 minutes, the mixture was analyzed by NMR spectroscopy.

^{1}H NMR (400 MHz, C_6D_6): δ (ppm) = 9.67 (d, $^{1}J_{PH}$ = 494.8 Hz, 1H, PH), 7.16 (s, 4H, CH), 1.69 (s, 36H, CH_3).
^{31}P NMR (162 MHz, C_6D_6): δ (ppm) = -11.5 (d, $^{1}J_{PH}$ = 494 Hz, $^{1}J_{PSe}$ = 673 Hz).
^{31}P{^{1}H} NMR (162 MHz, C_6D_6): δ (ppm) = -11.5 (s, $^{1}J_{PSe}$ = 673 Hz).

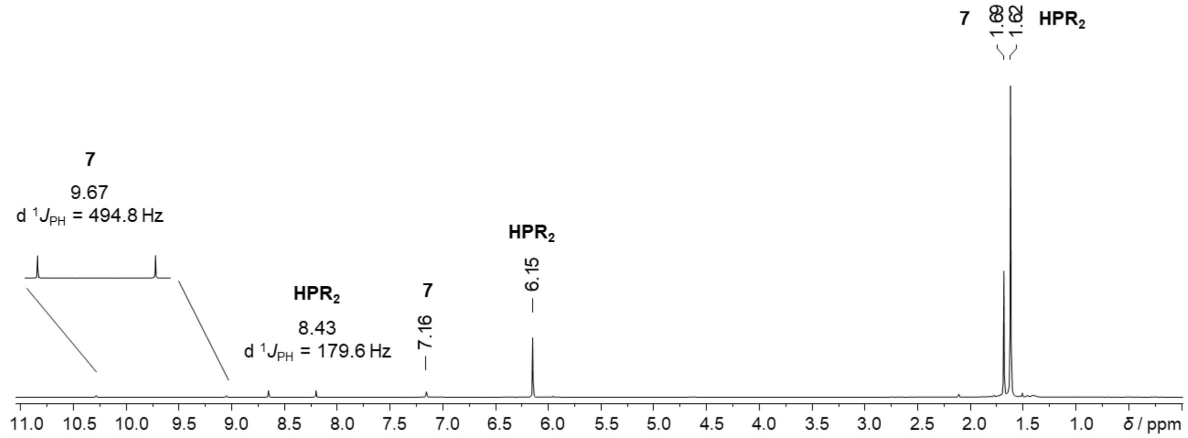

Figure S34: ^{1}H NMR spectrum (C_6D_6, 400 MHz) of the reaction mixture containing **7** (and phosphine **1**).

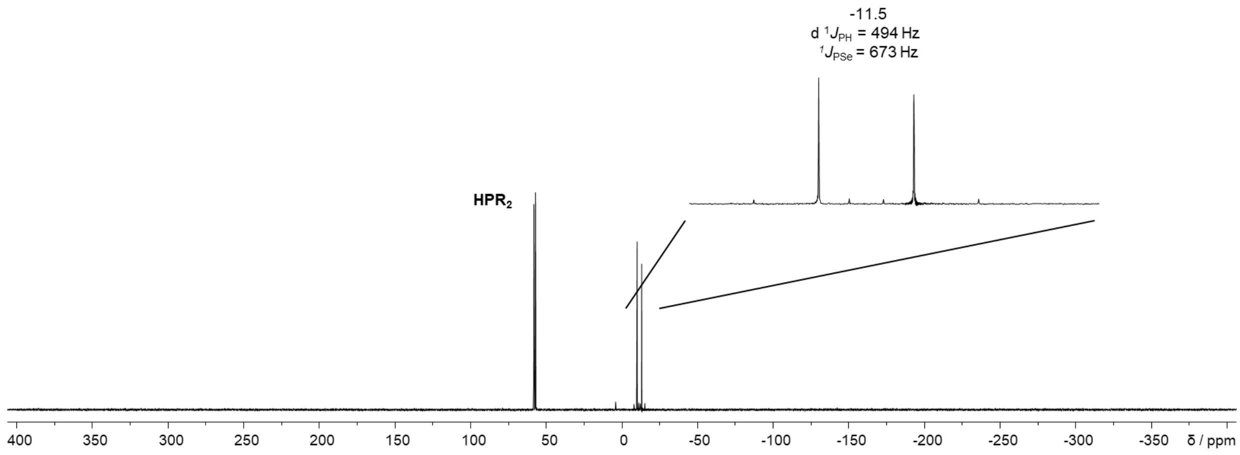

Figure S35: ^{31}P NMR spectrum (C_6D_6, 162 MHz) of the reaction mixture containing **7** (and phosphine **1**).

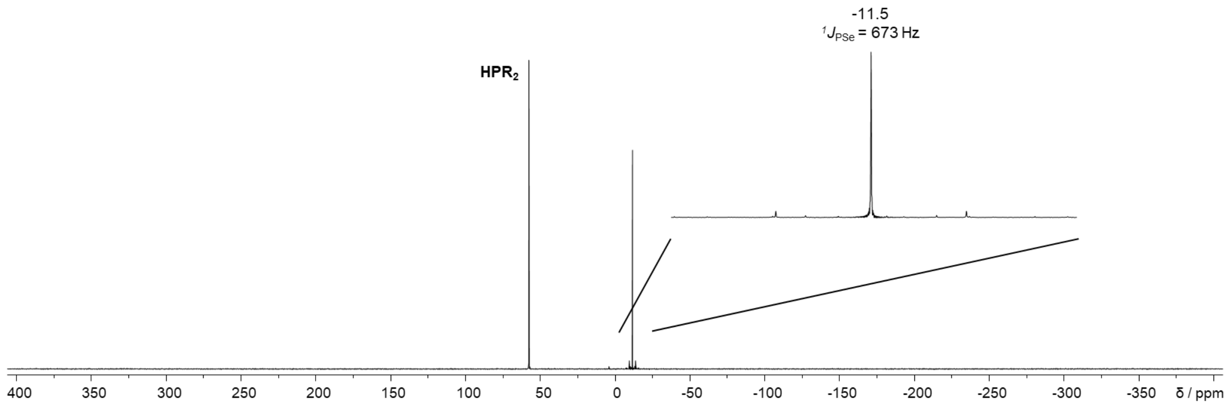

Figure S36: $^{31}P\{^1H\}$ NMR spectrum (C_6D_6, 162 MHz) of the reaction mixture containing **7** (and phosphine **1**).

1.5.2.2 Phosphine Selenide $(R^2)_2HPSe$ (**8**)

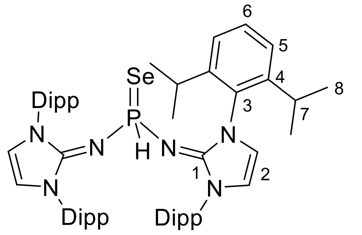

To compound **2** (125 mg, 0.15 mmol, 1.0 eq.) and an stoichiometric excess of grey selenium (15 mg, 0.19 mmol, 1.3 eq.), THF (3 mL) was added. The mixture was stirred for 16 hours at ambient temperature. After filtration, the volatiles were removed *in vacuo*. For further purification, crystals were obtained from cooling a saturated toluene solution and dried at 50 °C *in vacuo*.

^{1}H NMR (400 MHz, C_6D_6): δ (ppm) = 7.57 (d, $^1J_{PH}$ = 499.3 Hz, 1H, PH), 7.28 (t, $^3J_{HH}$ = 7.7 Hz, 4H, H6), 7.11 (dd, $^3J_{HH}$ = 7.7 Hz, $^4J_{HH}$ = 1.5 Hz, 4H, H5), 7.05 (dd, $^3J_{HH}$ = 7.7 Hz, $^4J_{HH}$ = 1.5 Hz, 4H, H5), 6.02 (s, 4H, CH, H2), 3.26 (sept, $^3J_{HH}$ = 6.8 Hz, 4H, C*H*(CH$_3$)$_2$, H7), 2.99 (sept, $^3J_{HH}$ = 6.8 Hz, 4H, C*H*(CH$_3$)$_2$, H7), 1.27 (d, $^3J_{HH}$ = 6.8 Hz, 12H, CH$_3$, H8), 1.18 (d, $^3J_{HH}$ = 6.8 Hz, 12H, CH$_3$, H8), 1.13 (d, $^3J_{HH}$ = 6.8 Hz, 12H, CH$_3$, H8), 1.09 (d, $^3J_{HH}$ = 6.8 Hz, 12H, CH$_3$, H8).

^{13}C{^{1}H} NMR (101 MHz, C_6D_6): δ (ppm) = 148.0 (C$_q$, C4), 147.6 (C$_q$, C4), 145.9 (d, $^2J_{PC}$ = 13 Hz, C=N, C1), 133.6 (C$_q$, C3), 129.5 (C6), 123.9 (C5), 124.0 (C5), 116.0 (C2), 29.1 (*C*H(CH$_3$)$_2$, C7), 28.7 (*C*H(CH$_3$)$_2$, C7), 25.5 (CH$_3$, C8), 25.2 (CH$_3$, C8), 24.0 (CH$_3$, C8).

^{31}P NMR (162 MHz, C_6D_6): δ (ppm) = -14.2 (d, $^1J_{PH}$ = 499 Hz, $^1J_{PSe}$ = 687 Hz).

^{31}P NMR (162 MHz, CD_2Cl_2): δ (ppm) = -16.2 (d, $^1J_{PH}$ = 504 Hz, $^1J_{PSe}$ = 673 Hz).

^{31}P{^{1}H} NMR (162 MHz, C_6D_6): δ (ppm) = -14.2 (s, $^1J_{PSe}$ = 687 Hz).

^{77}Se NMR (76 MHz, C_6D_6): δ (ppm) = 10.7 (d, $^1J_{PSe}$ = 687 Hz).

HRMS (ESI, positive): m/z calculated for $[C_{54}H_{74}N_6SeP]^+$ (**8**+H)$^+$ 917.4872, found: 917.4863.

Crystals suitable for **SCXRD** were obtained from diffusing *n*-hexane into a saturated toluene solution at -40 °C.

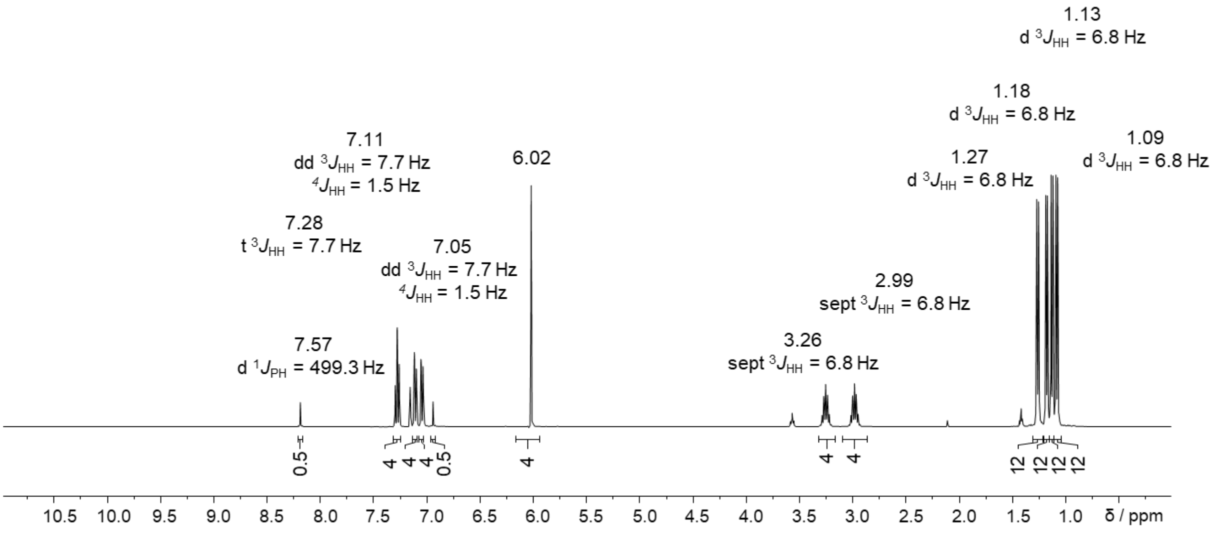

Figure S37: ^{1}H NMR spectrum (C_6D_6, 400 MHz) of **8**.

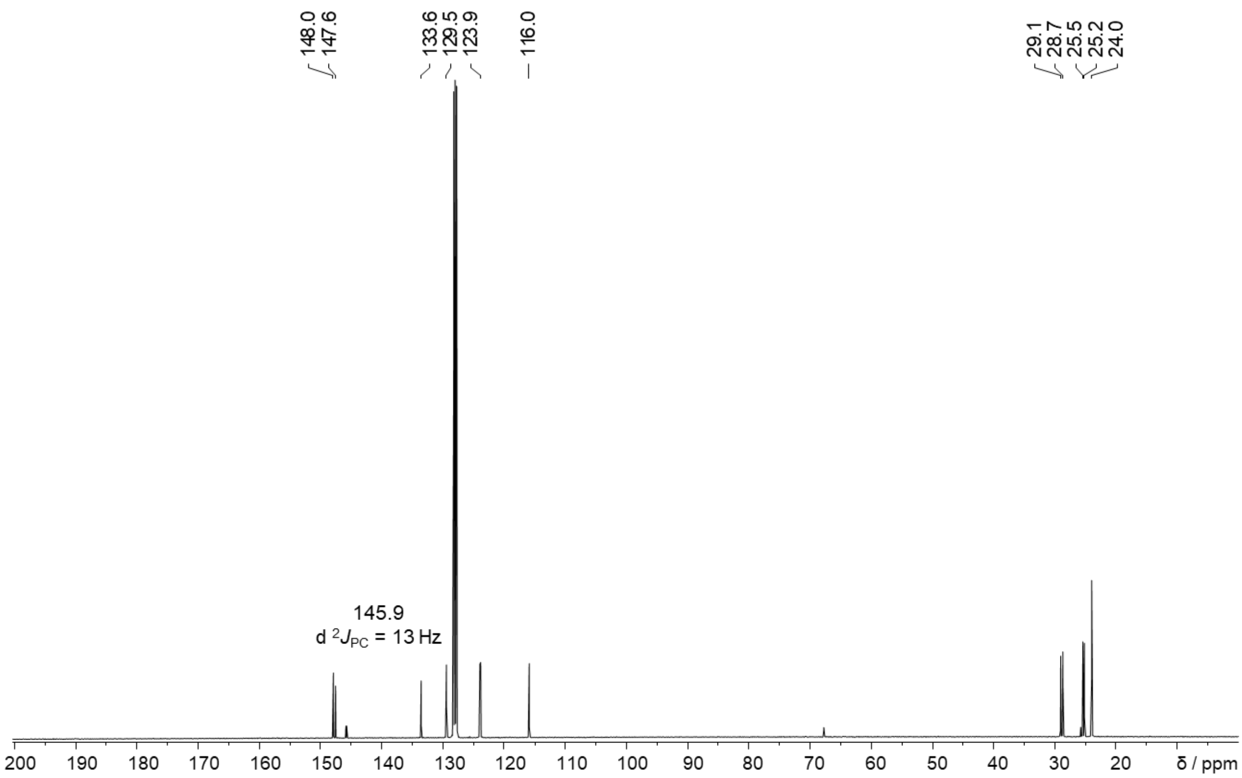

Figure S38: $^{13}C\{^1H\}$ NMR spectrum (C_6D_6, 101 MHz) of **8**.

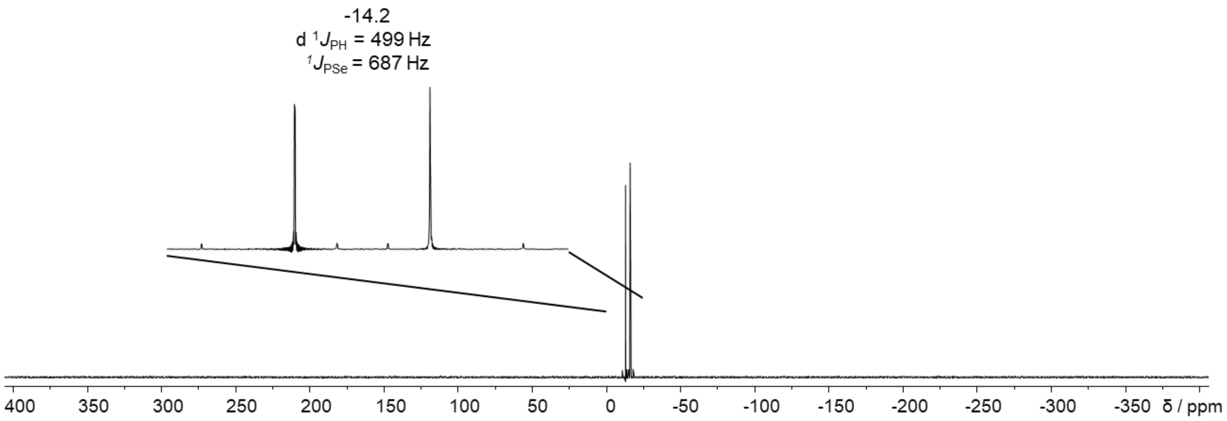

Figure S39: ^{31}P NMR spectrum (C_6D_6, 162 MHz) of **8**.

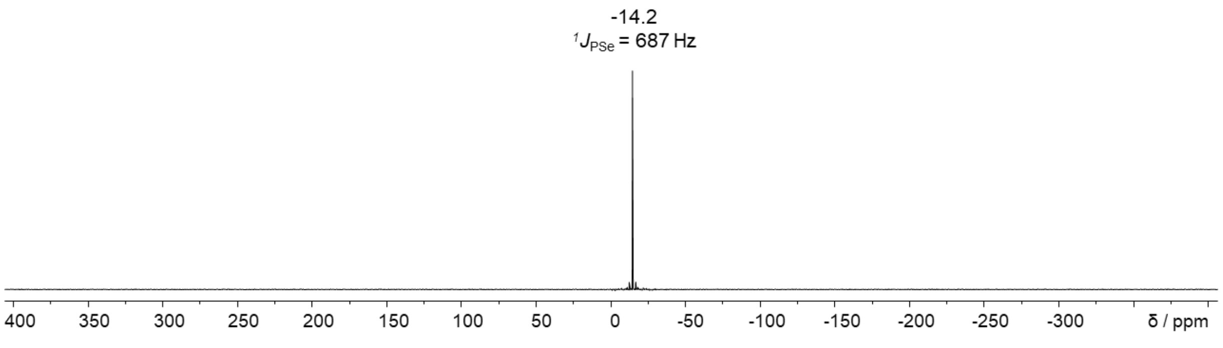

Figure S40: $^{31}P\{^1H\}$ NMR spectrum (C_6D_6, 162 MHz) of **8**.

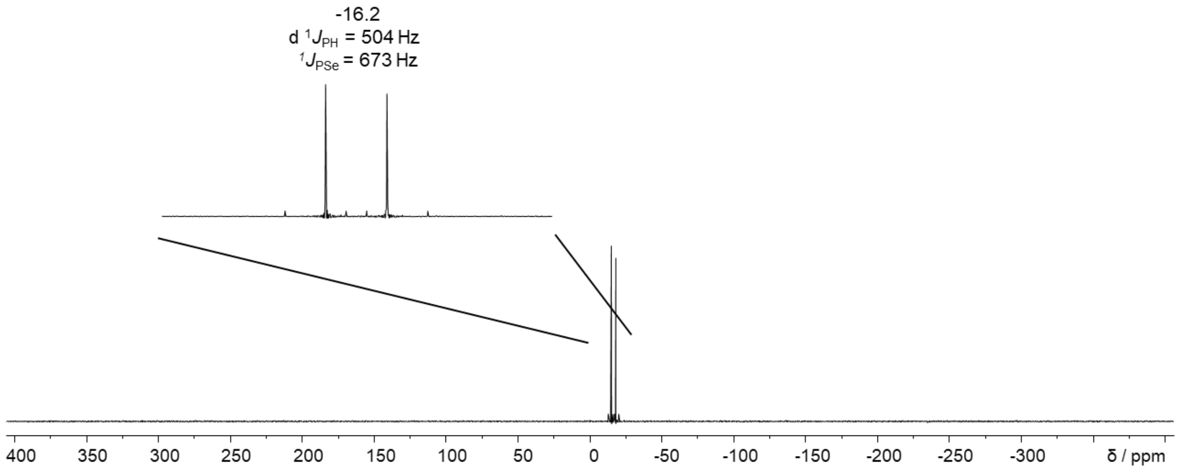

Figure S41: ^{31}P NMR spectrum (CD_2Cl_2, 162 MHz) of **8**.

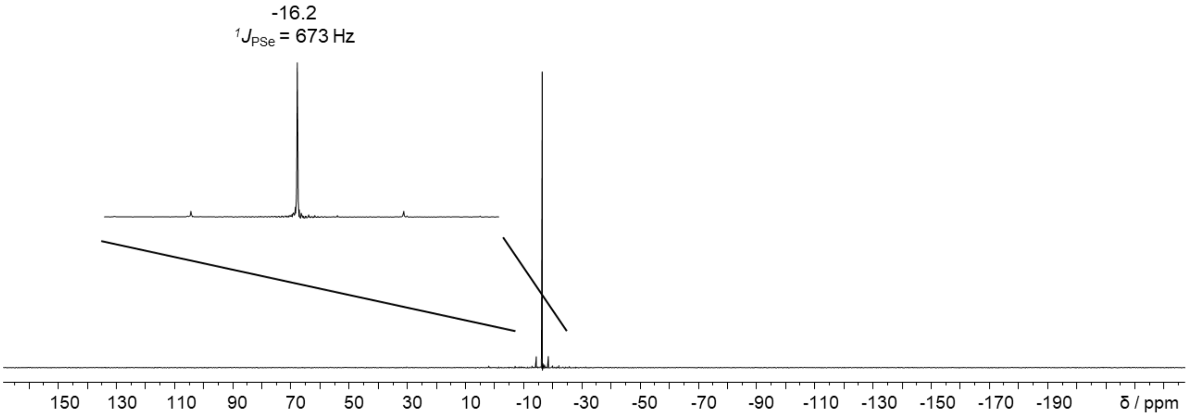

Figure S42: ^{31}P{^{1}H} NMR spectrum (CD_2Cl_2, 162 MHz) of **8**.

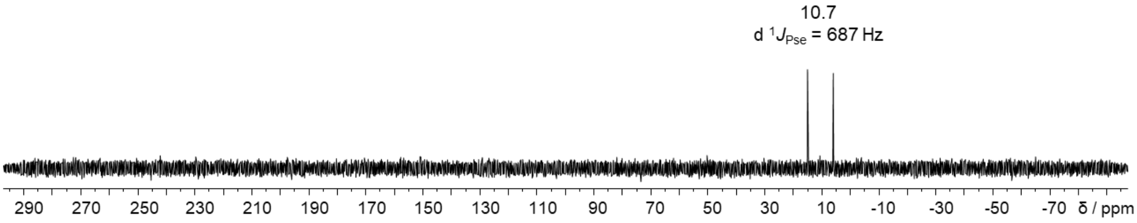

Figure S43: ^{77}Se NMR spectrum (C_6D_6, 76 MHz) of **8**.

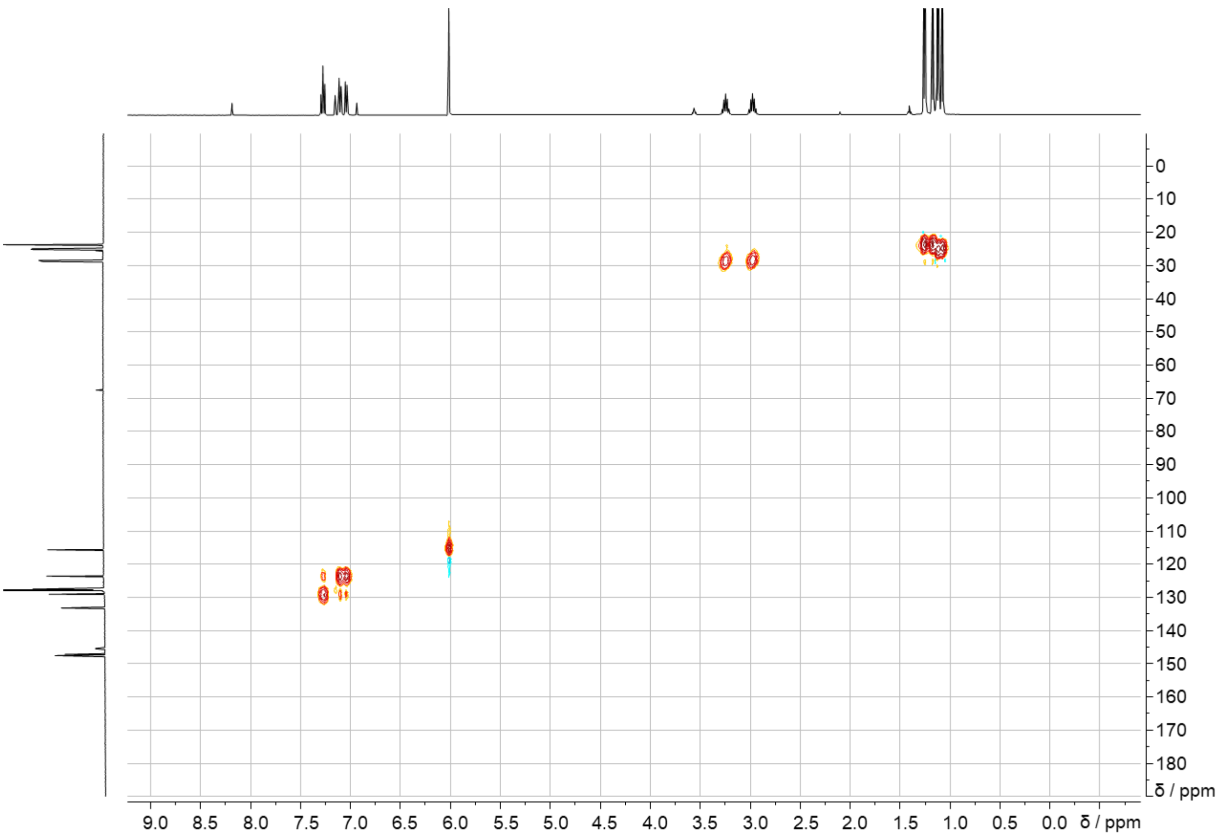

Figure S44: ^{1}H $^{13}C\{^{1}H\}$ HSQC 2D NMR spectrum (C_6D_6, 400 MHz, 101 MHz) of **8**.

1.5.2.3 Phosphine selenide $(R^3)_2HPSe$ (**9**)

To compound **3** (30 mg, 0.076 mmol, 1.0 eq.) and grey selenium (6 mg, 0.08 mmol, 1 eq.), benzene (0.7 mL) was added. The mixture was heated to 70 °C for 60 hours in a closed reaction vessel. After filtration and removal of the solvent from the filtrate *in vacuo*, the resulting solid was dissolved in CH_2Cl_2, and the solution was analyzed by ^{31}P NMR spectroscopy.

^{31}P NMR (162 MHz, CH_2Cl_2): δ (ppm) = -21.9 (d, $^1J_{PH}$ = 530 Hz).
^{31}P{^{1}H} NMR (162 MHz, CH_2Cl_2): δ (ppm) = -21.9 (s, $^1J_{PSe}$ = 659 Hz).

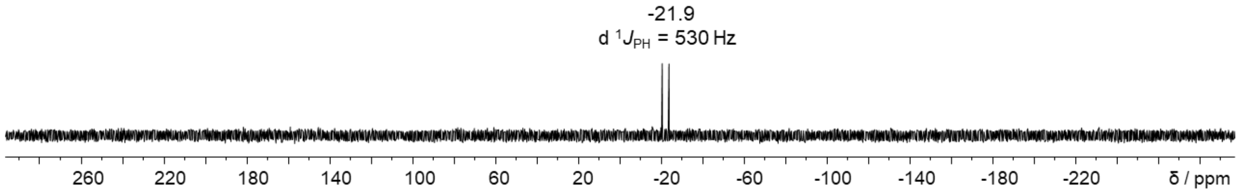

Figure S45: ^{31}P NMR spectrum (CH_2Cl_2, 162 MHz) of **9**.

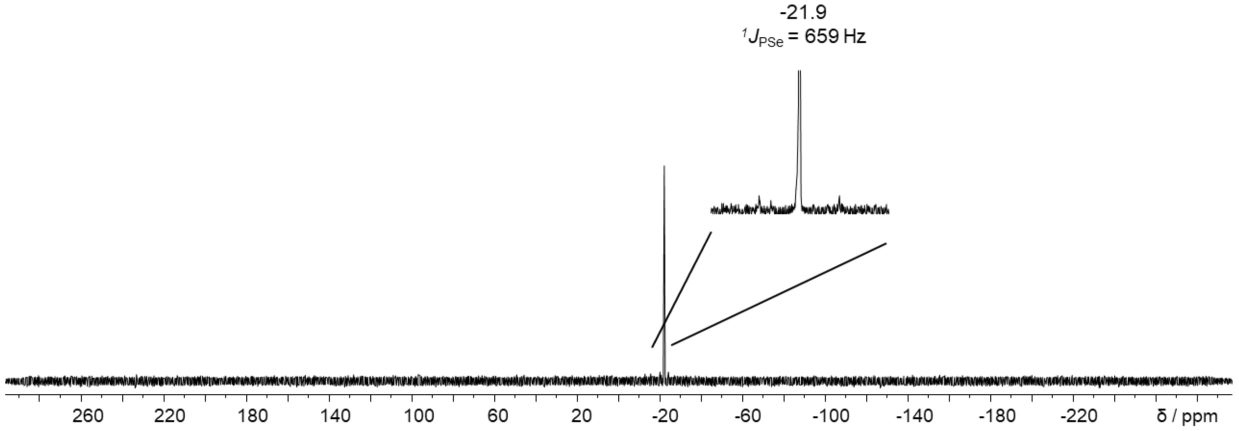

Figure S46: ^{31}P{^{1}H} NMR spectrum (CH_2Cl_2, 162 MHz) of **9**.

As an alternative synthetic procedure to obtain the phosphine selenide **9**, triphenylphosphine selenide was used as a soluble source of selenium. An NMR tube equipped with a PTFE valve was charged with triphenylphosphine selenide (26 mg, 0.076 mmol, 1.0 eq.), **3** (30 mg, 0.076 mmol, 1.0 eq.), and C_6D_6 (1 mL). After storing the tube for 5 days at ambient temperature, the sample was analyzed by NMR spectroscopy.

^{1}H NMR (400 MHz, C_6D_6): δ (ppm) = 9.76 (d, $^1J_{PH}$ = 522.2 Hz, 1H, PH), 2.75 (s, 8H, CH_2), 1.50 (s, 36H, CH_3).
^{31}P NMR (162 MHz, C_6D_6): δ (ppm) = -18.0 (d, $^1J_{PH}$ = 522 Hz, $^1J_{PSe}$ = 695 Hz).
^{31}P{^{1}H} NMR (162 MHz, C_6D_6): δ (ppm) = -18.0 (s, $^1J_{PSe}$ = 695 Hz).

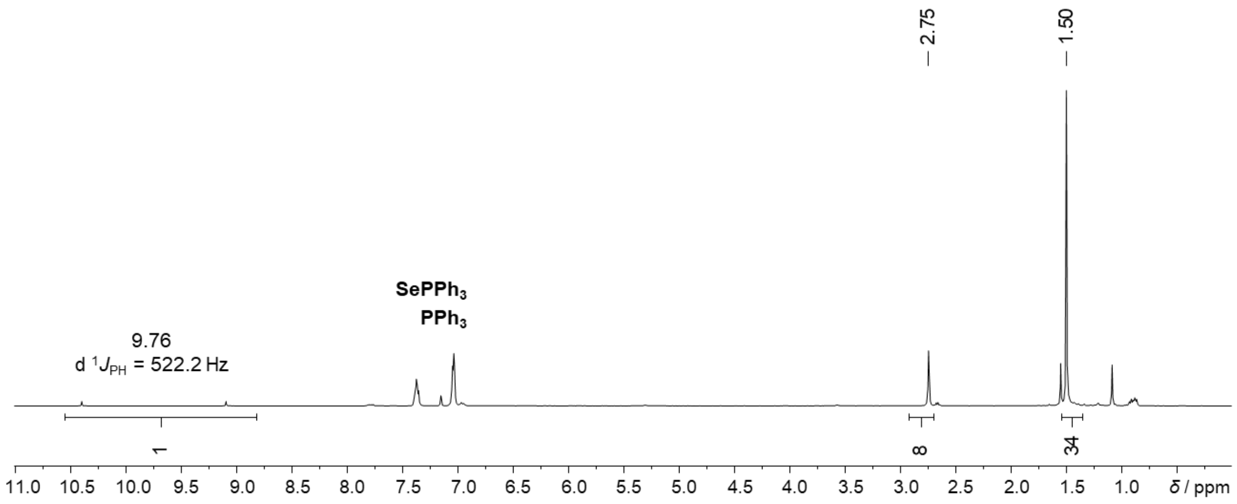

Figure S47: ^{1}H NMR spectrum (C_6D_6, 400 MHz) of the reaction mixture containing **9**, triphenylphosphine and triphenylphosphine selenide.

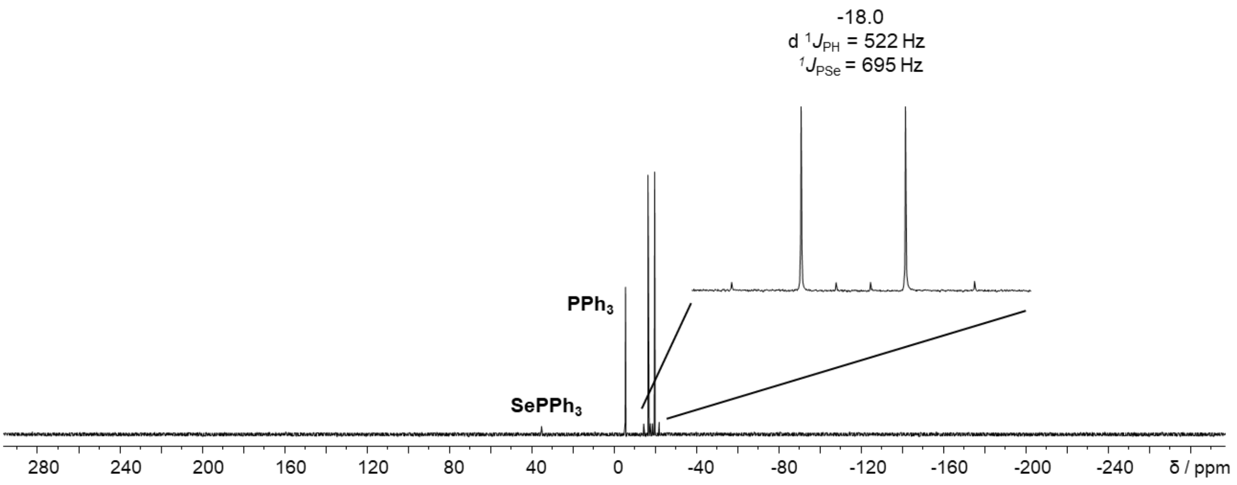

Figure S48: ^{31}P NMR spectrum (C_6D_6, 162 MHz) of the reaction mixture containing **9**, triphenylphosphine and triphenylphosphine selenide.

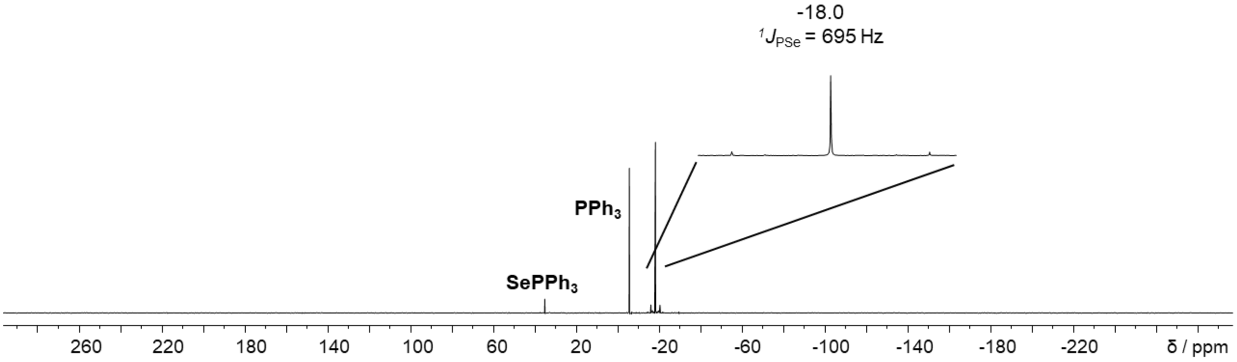

Figure S49: $^{31}P\{^1H\}$ NMR spectrum (C_6D_6, 162 MHz) of the reaction mixture containing **9**, triphenylphosphine and triphenylphosphine selenide.

1.5.3 Summary of the Experiments on Donor Properties

Table S1: Values obtained by methods for the determination of donor strengths of **1**-**3** described in detail above. ([a] in DCM determined coupling constants ($^1J_{PSe}$) are strongly influenced by coordination of solvent protons to the NHI substituents and therefore do not follow the general trend.)

	**1**	**2**	**3**
$^1J_{PSe}$ (C_6D_6) / Hz	673	687	695
$^1J_{PSe}$ (DCM)[a] / Hz	638	673	659
TEP (DCM) / cm^{-1}	2043.1	2048.7	2051.5
TEP (neat) / cm^{-1}	2037.3	2046.5	*not determined*

1.6 Oxidation of Secondary IAPs 1-3 with N_2O

The respective phosphine **1**, **2** or **3** (0.13 mmol) was dissolved in C_6D_6 (0.6 mL) and pressurized with 2 bar N_2O in a Teflon-sealed NMR tube. According to NMR spectroscopy, the conversion to the phosphine oxide (R_2HP=O; **10**: R = R^1, **11**: R = R^2, **12**: R = R^1) was quantitative for **1** and **3** after 15 minutes, for **2** after two weeks.

1.6.1 Characterization data of 10

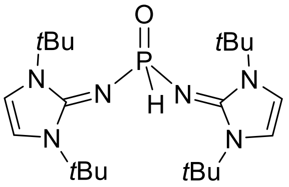

^{1}H NMR (400 MHz, C_6D_6): *δ* (ppm) = 8.22 (d, $^1J_{PH}$ = 539.4 Hz, 1H, PH), 6.18 (s, 4H, CH), 1.67 (s, 36H, CH_3).

$^{13}C\{^1H\}$ NMR (101 MHz, C_6D_6): *δ* (ppm) = 143.4 (d, $^2J_{CP}$ = 18.5 Hz, C=N), 109.0 (C=C), 56.4 (*C*(CH_3)$_3$), 29.5 (CH_3).

^{31}P NMR (162 MHz, C_6D_6): *δ* (ppm) = -26.0 (d, $^1J_{PH}$ = 539.4 Hz).

$^{31}P\{^1H\}$ NMR (162 MHz, C_6D_6): *δ* (ppm) = -26.0 (s).

HRMS (ESI, positive): m/z calculated for $[C_{22}H_{42}ON_6P]^+$ (**10**+H)$^+$ 437.3152, found: 437.3152.

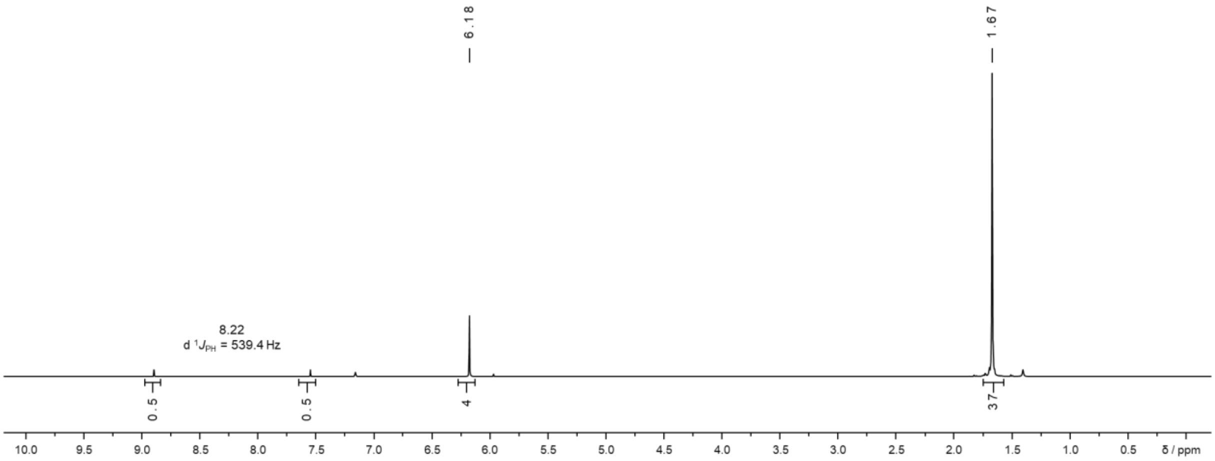

Figure S50: ^{1}H NMR spectrum (C_6D_6, 400 MHz) of **10**.

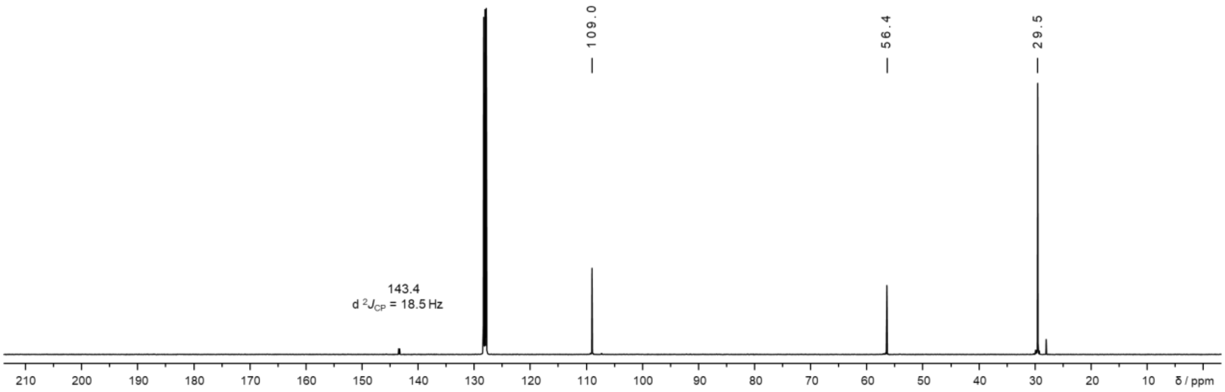

Figure S51: ^{13}C{^{1}H} NMR spectrum (C_6D_6, 101 MHz) of **10**.

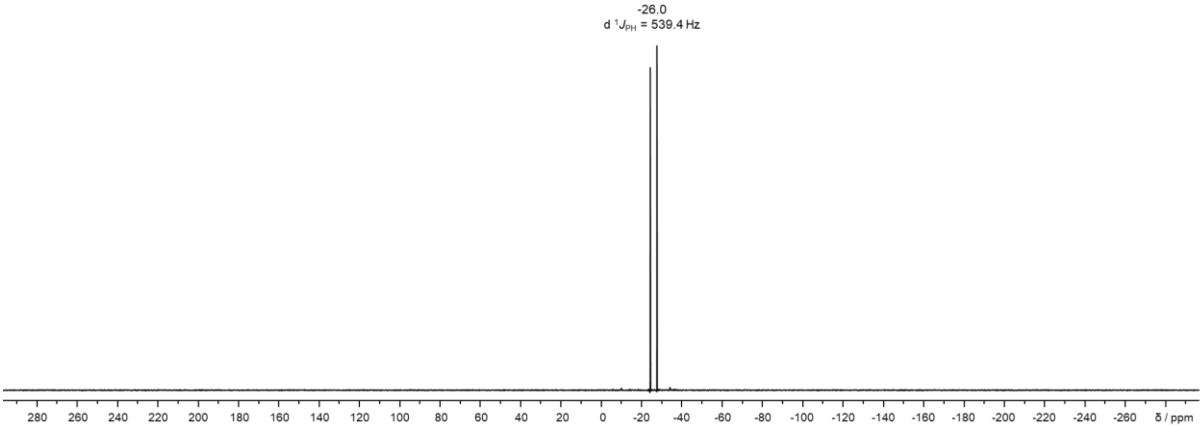

Figure S52: ^{31}P NMR spectrum (C_6D_6, 162 MHz) of **10**.

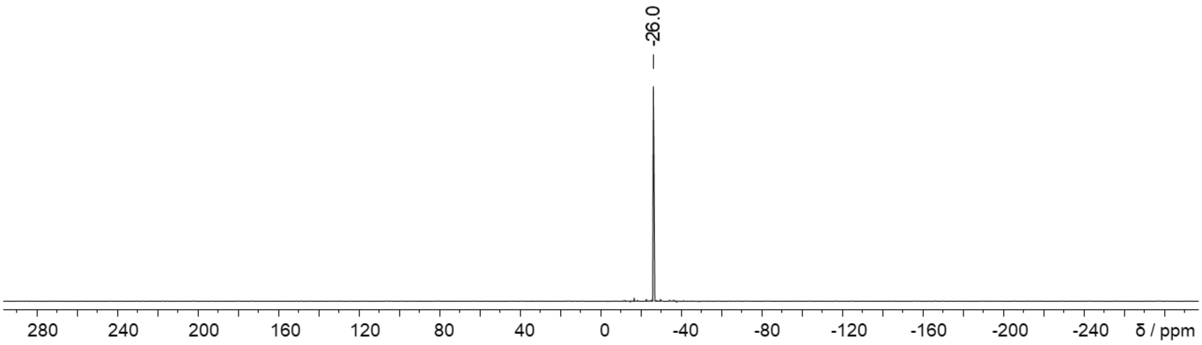

Figure S53: $^{31}P\{^{1}H\}$ NMR spectrum (C_6D_6, 162 MHz) of **10**.

1.6.2 Characterization data of 11

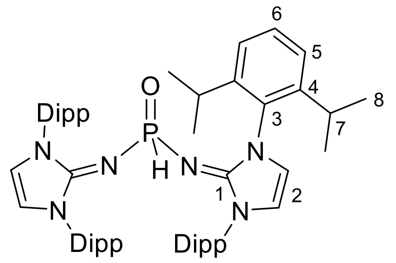

^{1}H NMR (400 MHz, C_6D_6): δ (ppm) = 7.27 (t, $^3J_{HH}$ = 7.7 Hz, 4H, H6), 7.11 (dd, $^3J_{HH}$ = 7.7 Hz, $^4J_{HH}$ = 1.5 Hz, 4H, H5), 7.04 (dd, $^3J_{HH}$ = 7.7 Hz, $^4J_{HH}$ = 1.5 Hz, 4H, H5), 5.88 (s, 4H, CH, H2), 5.88 (d, $^1J_{PH}$ = 535.9 Hz, 1H, PH), 3.07 (sept, $^3J_{HH}$ = 6.9 Hz, 4H, C*H*(CH_3)$_2$, H7), 2.86 (sept, $^3J_{HH}$ = 6.9 Hz, 4H, C*H*(CH_3)$_2$, H7), 1.25 (d, $^3J_{HH}$ = 6.9 Hz, 12H, CH_3, H8), 1.17 (d, $^3J_{HH}$ = 6.9 Hz, 12H, CH_3, H8), 1.15 (d, $^3J_{HH}$ = 6.9 Hz, 12H, CH_3, H8), 1.13 (d, $^3J_{HH}$ = 6.9 Hz, 12H, CH_3, H8).

^{13}C{^{1}H} NMR (101 MHz, C_6D_6): δ (ppm) = 148.3 (C_q, C4), 147.6 (C_q, C4), 146.5 (d, $^2J_{PC}$ = 12 Hz, $C_{q.}$, C=N, C1), 134.0 (C_q, C3), 129.3 (CH, C6), 123.9 (CH, C5), 123.6 (CH, C5), 114.9 (CH, C2), 29.2 (*C*H(CH_3)$_2$, C7), 28.9 (*C*H(CH_3)$_2$, C7), 25.0 (CH_3, C8), 24.5 (CH_3, C8), 23.9 (CH_3, C8), 23.5 (CH_3, C8).

^{31}P NMR (162 MHz, C_6D_6): δ (ppm) = -20.8 (d, $^1J_{PH}$ = 535 Hz).

^{31}P{^{1}H} NMR (162 MHz, C_6D_6): δ (ppm) = -20.8 (s).

HRMS (ESI, positive): m/z calculated for [$C_{54}H_{74}N_6OP$]$^+$ (**11**+H)$^+$ 853.5656, found: 853.5643.

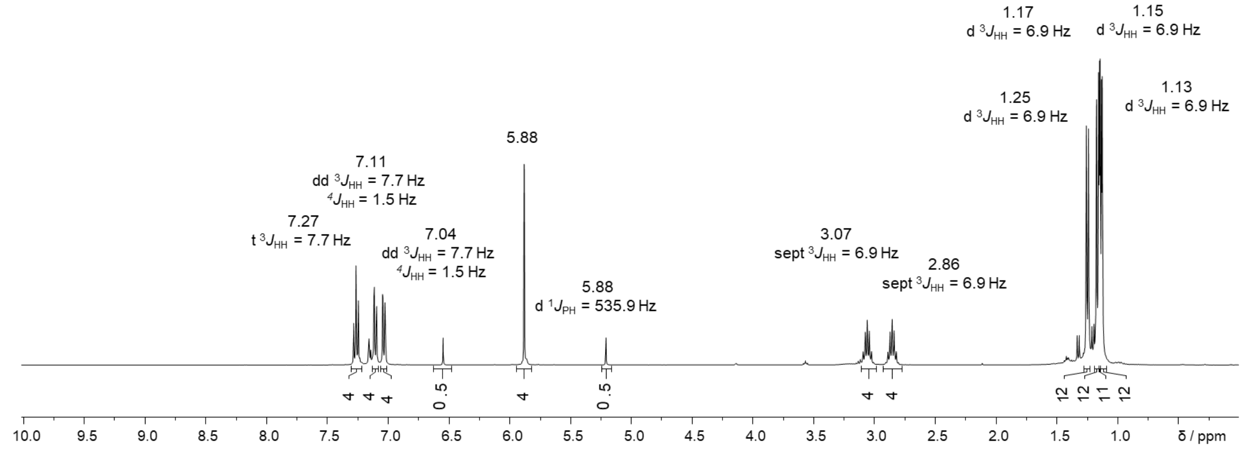

Figure S54: ^{1}H NMR spectrum (C_6D_6, 400 MHz) of **11**.

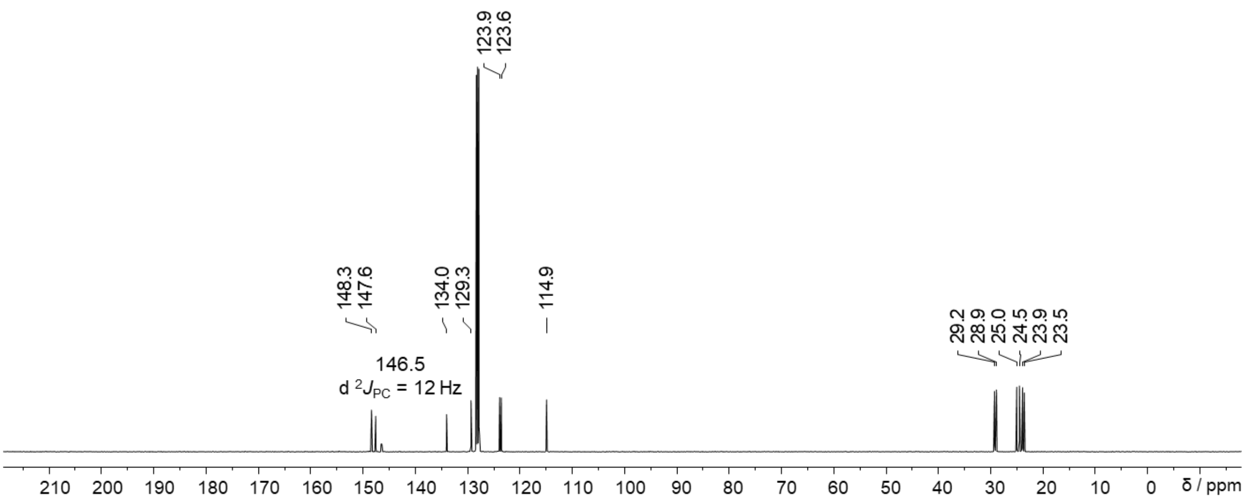

Figure S55: $^{13}C\{^1H\}$ NMR spectrum (C_6D_6, 101 MHz) of **11**.

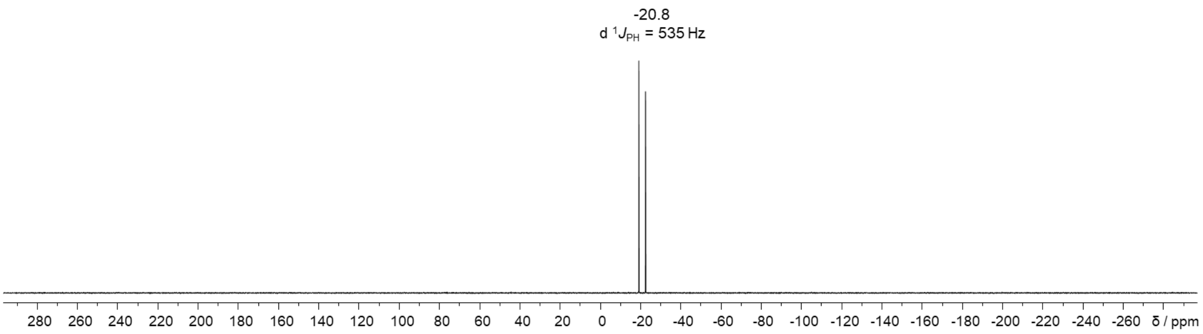

Figure S56: ^{31}P NMR spectrum (C_6D_6, 162 MHz) of **11**.

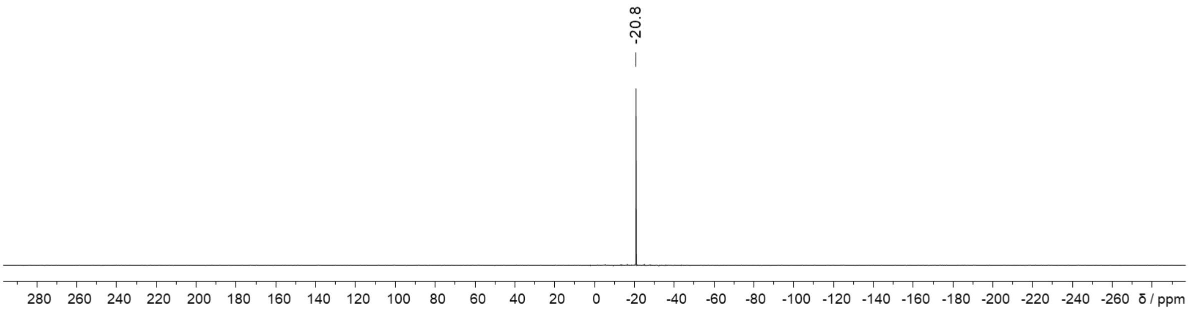

Figure S57: $^{31}P\{^1H\}$ NMR spectrum (C_6D_6, 162 MHz) of **11**.

1.6.3 Characterization data of 12

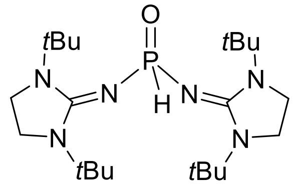

^{1}H NMR (400 MHz, C_6D_6): δ (ppm) = 8.20 (d, $^{1}J_{PH}$ = 580.3 Hz, 1H, PH), 2.78 – 2.72 (m, 8H, CH_2), 1.50 (s, 36H, CH_3).

^{13}C{^{1}H} NMR (101 MHz, C_6D_6): δ (ppm) = 149.6 (d, $^{2}J_{CP}$ = 23.1 Hz, C=N), 54.0 ($C(CH_3)_3$), 41.8 ($H_2C–CH_2$), 28.6 (CH_3).

^{31}P NMR (162 MHz, C_6D_6): δ (ppm) = -36.3 (d, $^{1}J_{PH}$ = 580.3 Hz).

^{31}P{^{1}H} NMR (162 MHz, C_6D_6): δ (ppm) = -36.3 (s).

HRMS (ESI, positive): m/z calculated for $[C_{22}H_{46}ON_6P]^+$ (**12**+H)$^+$ 441.3465, found: 441.3462.

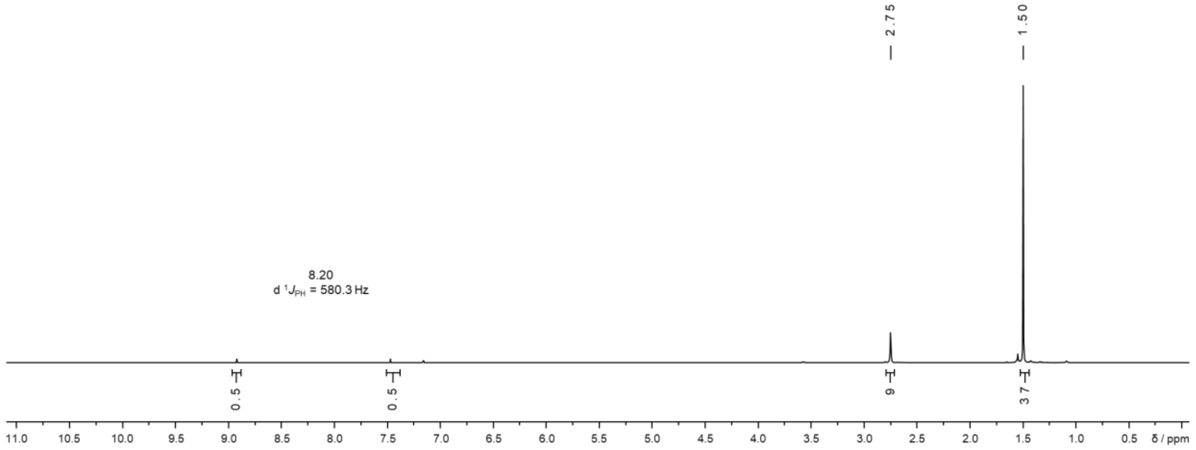

Figure S58: ^{1}H NMR spectrum (C_6D_6, 400 MHz) of **12**.

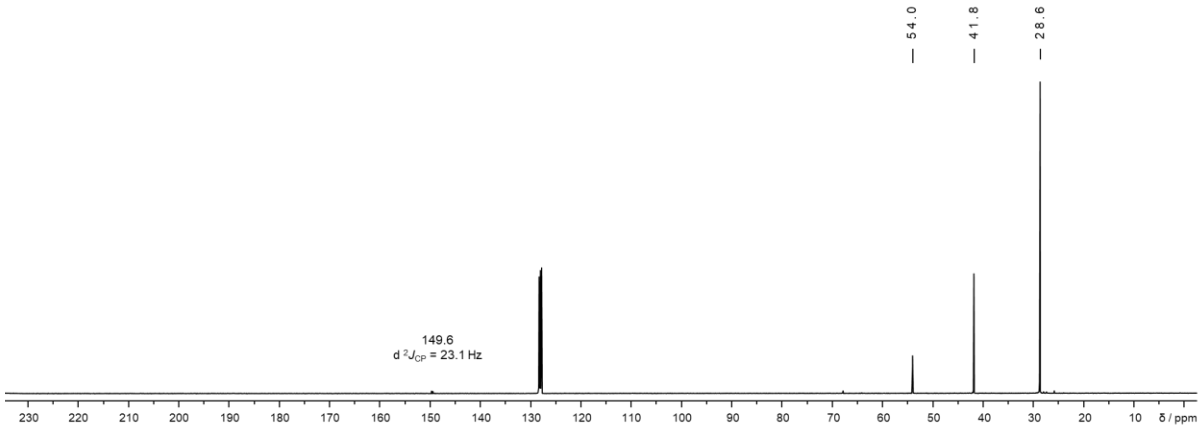

Figure S59: ^{13}C{^{1}H} NMR spectrum (C_6D_6, 101 MHz) of **12**.

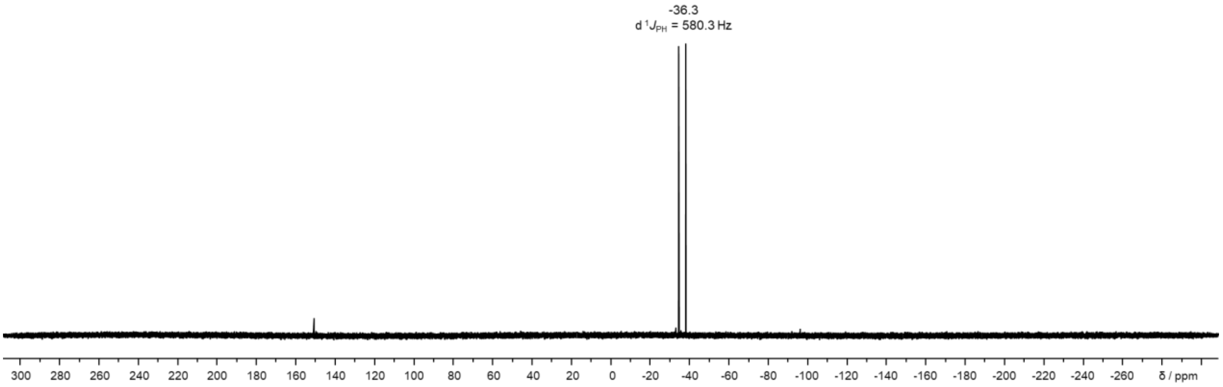

Figure S60: ^{31}P NMR spectrum (C_6D_6, 162 MHz) of **12**.

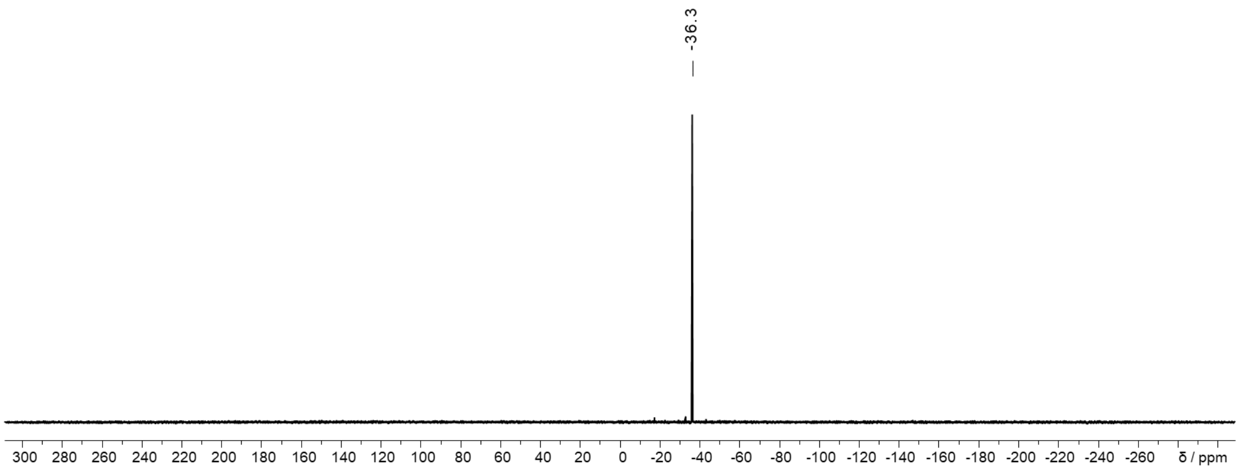

Figure S61: $^{31}P\{^1H\}$ NMR spectrum (C_6D_6, 162 MHz) of 1**2**.

1.8 Synthesis of Gold Complexes

The gold(I) complex (1,3-Bis-(2,6-diisopropylphenyl)-imidazol-2-yliden)gold-chloride (62 mg, 0.10 mmol, 1.0 eq.) and $AgSbF_6$ (34 mg, 0.10 mmol, 1.0 eq.) were stirred in difluorobenzene (3 mL) under exclusion of light for 30 minutes at ambient temperature. The resulting suspension was filtered into a stirred solution of the secondary IAP **1** or **3** (**1**: 42 mg, **3**: 41 mg; 0.10 mmol, 1.0 eq.) in toluene (1 mL). The reaction mixture was stirred for 30 minutes (**1**) or 1 hour (**3**). The solvents were removed *in vacuo* and the residue was dissolved in DCM (1 mL). Slow diffusion of diethyl ether into the DCM solution at -40 °C gave the gold complexes **13** and **14** as white, crystalline solids.

1.8.1 Characterization data of [13][SbF_6] ([1-Au-IDipp][SbF_6])

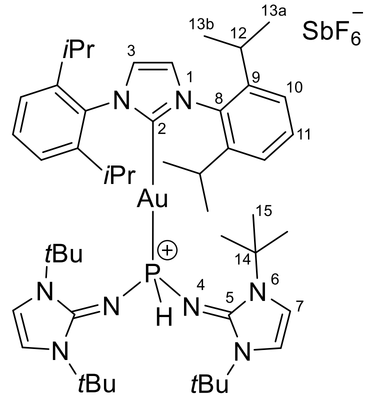

^{1}H NMR (400 MHz, CD_2Cl_2): δ (ppm) = 8.99 (d, $^1J_{PH}$ = 382.5 Hz, 1H, PH), 7.53 (t, $^3J_{HH}$ = 7.8 Hz, 2H, H11), 7.29 (d, $^3J_{HH}$ = 7.8 Hz, 4H, H10), 7.17 (d, $^5J_{PH}$ = 1.1 Hz, 2H, H3), 6.47 (s, 4H, H7), 2.47 (sept, $^3J_{HH}$ = 6.9 Hz, 4H, H12), 1.35 (s, 36H, H15), 1.18 (d, $^3J_{HH}$ = 6.9 Hz, 12H, H13a), 1.10 (d, $^3J_{HH}$ = 6.9 Hz, 12H, H13b).

^{13}C{^{1}H} NMR (101 MHz, CD_2Cl_2): δ (ppm) = *C2 not visible due to low concentration*, 142.5 (d, $^2J_{PC}$ = 18.7 Hz, C5), 145.7 (C9), 134.0 (C8), 130.8 (C11), 124.4 (d, $^4J_{PC}$ = 4.1 Hz, C3), 124.3 (C10), 56.0 (C7), 29.0 (d, $^5J_{PC}$ = 1.7 Hz, C15), 28.6 (C12), 24.4 (C13a), 23.4 (C13b).

^{31}P NMR (162 MHz, CD_2Cl_2): δ (ppm) = 35.2 (d, $^1J_{PH}$ = 382.8 Hz).

^{31}P{^{1}H} NMR (162 MHz, CD_2Cl_2): δ (ppm) = 35.2.

^{19}F NMR (377 MHz, CD_2Cl_2): δ (ppm) = -110 – -138 (m).

HRMS (ESI, positive): m/z calculated for $[C_{49}H_{77}AuN_8P]^+$ (**13**)$^+$ 1005.5669, found: 1005.5646.

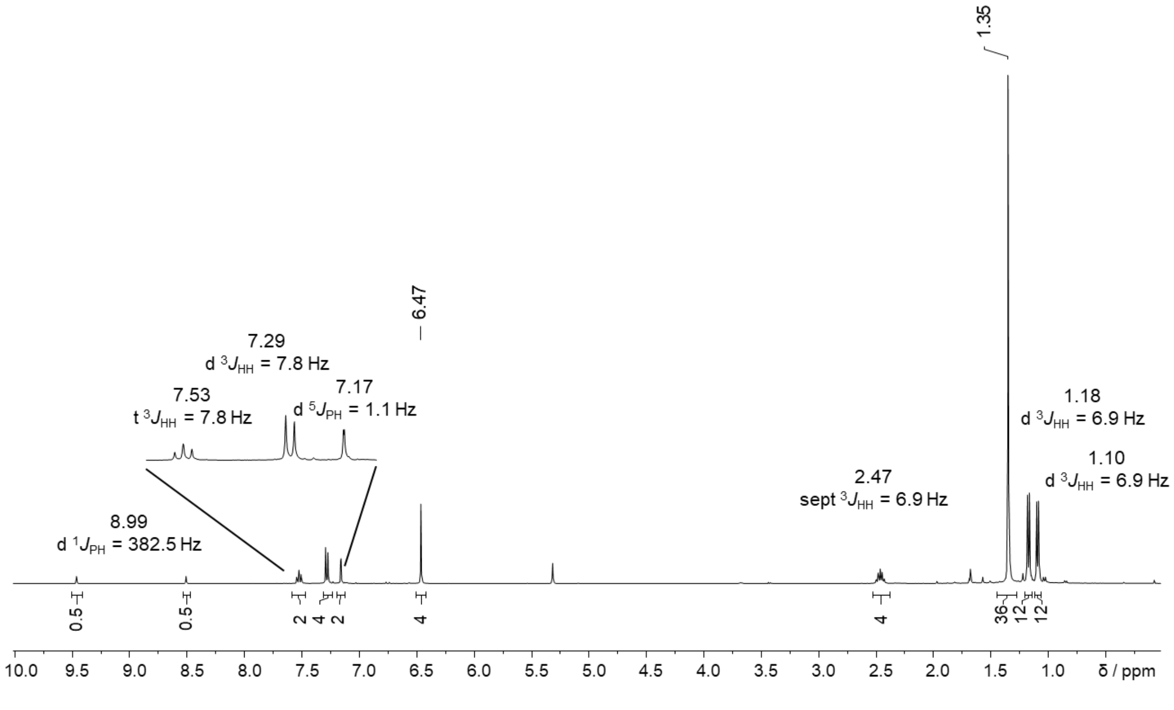

Figure S62: ^{1}H NMR spectrum (CD_2Cl_2, 400 MHz) of [**13**][SbF_6].

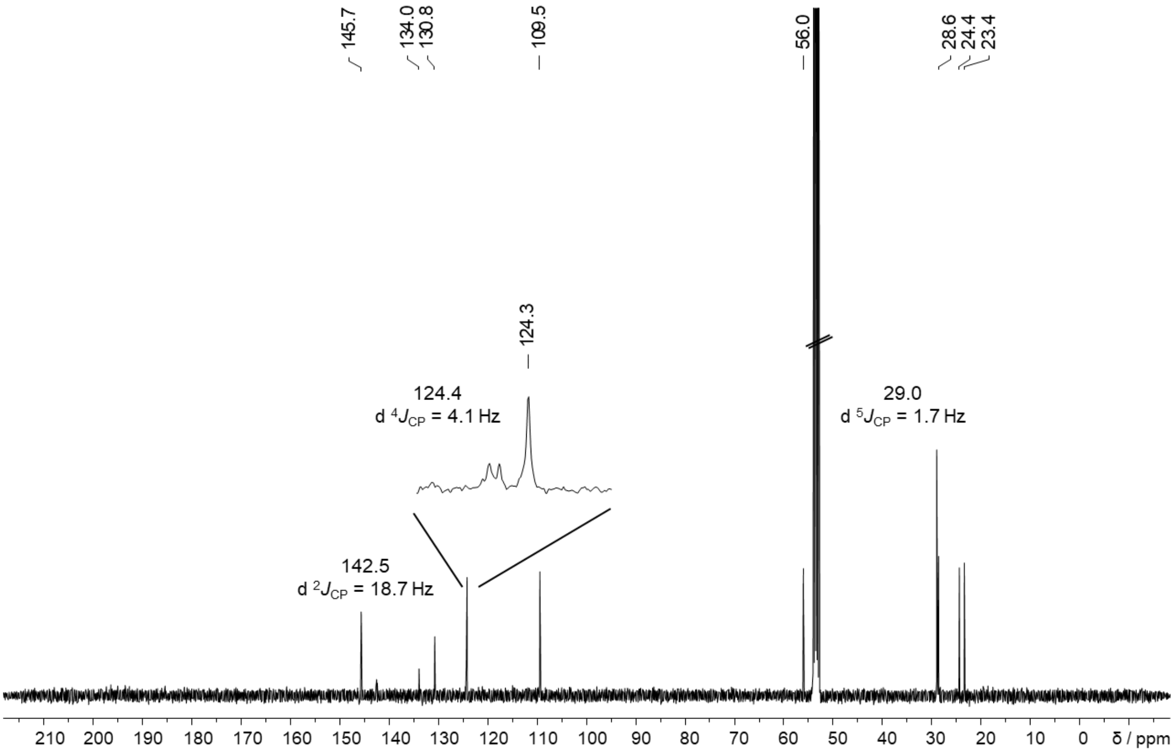

Figure S63: $^{13}C\{^1H\}$ NMR spectrum (CD_2Cl_2, 101 MHz) of [**13**][SbF_6].

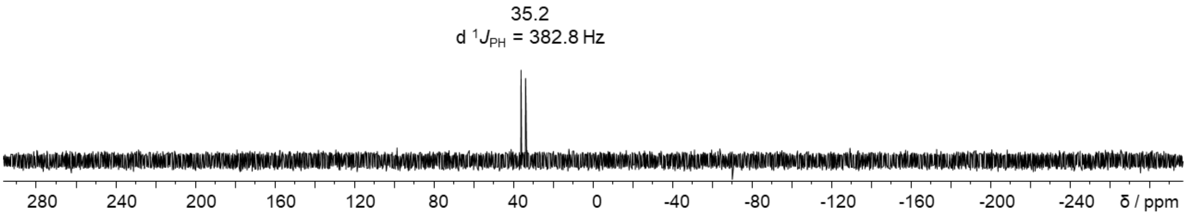

Figure S64: ^{31}P NMR spectrum (CD_2Cl_2, 162 MHz) of [**13**][SbF_6].

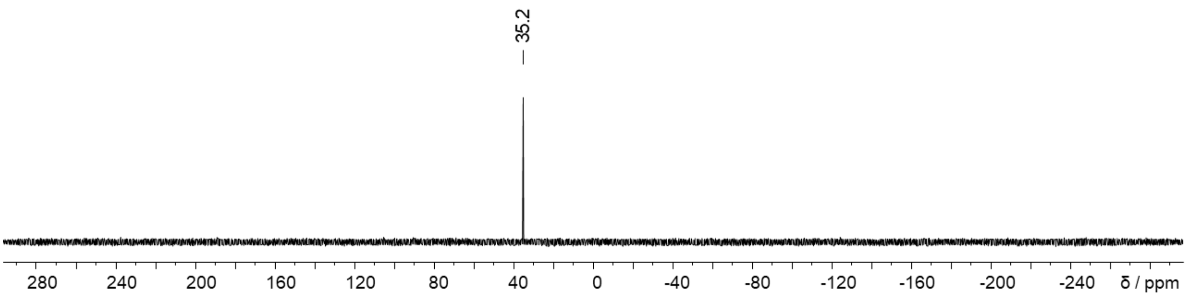

Figure S65: $^{31}P\{^1H\}$ NMR spectrum (CD_2Cl_2, 162 MHz) of [**13**][SbF_6].

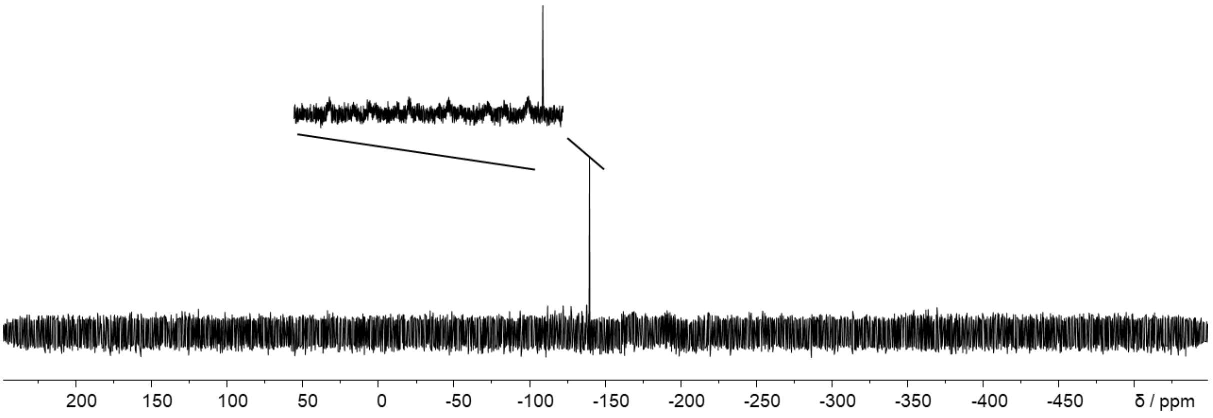

Figure S66: ^{19}F NMR spectrum (CD_2Cl_2, 377 MHz) of [**13**][SbF_6], the sharp singlet is provoked by an unknown contaminant.

1.8.2 Characterization data of [14][SbF6] ([3-Au-IDipp][SbF6])

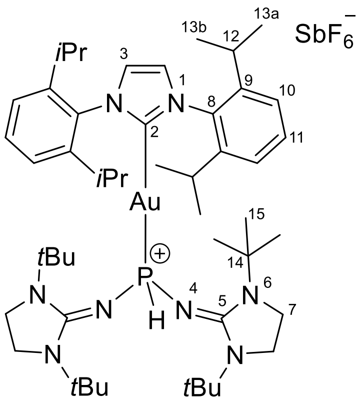

Yield 50% (62 mg, 0.050 mmol).

^{1}H NMR (500 MHz, CD_2Cl_2): δ (ppm) = 8.77 (d, $^1J_{PH}$ = 396.2 Hz, 1H, PH), 7.55 (t, $^3J_{HH}$ = 7.8 Hz, 2H, H11), 7.34 (d, $^3J_{HH}$ = 7.8 Hz, 4H, H10), 7.23 (d, $^5J_{PH}$ = 0.9 Hz, 2H, H3), 3.19 (s, 8H, H7), 2.53 (sept, $^3J_{HH}$ = 6.8 Hz, 4H, H12), 1.27 (d, $^3J_{HH}$ = 6.8 Hz, 12H, H13a), 1.23 (d, $^3J_{HH}$ = 6.8 Hz, 12H, H13b), 1.11 (s, 36H, H15).

^{13}C{^{1}H} NMR (126 MHz, CD_2Cl_2): δ (ppm) = 193.4 (d, $^2J_{PC}$ = 162.9 Hz, C2), 153.5 (d, $^2J_{PC}$ = 14.8 Hz, C5), 146.3 (C9), 134.5 (C8), 131.5 (C11), 125.0 (d, $^4J_{PC}$ = 4.4 Hz, C3), 124.9 (C10), 42.8 (C7), 29.3 (C12), 28.9 (d, $^5J_{PC}$ = 1.6 Hz, C15), 25.2 (C13a), 24.1 (C13b).

^{31}P NMR (202 MHz, CD_2Cl_2): δ (ppm) = 32.5 (d, $^1J_{PH}$ = 396.2 Hz).

^{31}P{^{1}H} NMR (202 MHz, CD_2Cl_2): δ (ppm) = 32.5.

^{19}F NMR (471 MHz, CD_2Cl_2): δ (ppm) = -114.2 – -134.8 (m).

HRMS (ESI, positive): m/z calculated for $[C_{49}H_{81}AuN_8P]^+$ (**14**)$^+$ 1009.5982, found: 1009.5978.

Colorless crystals suitable for **SCXRD** were obtained from diffusing diethyl ether into a saturated THF solution at -40 °C.

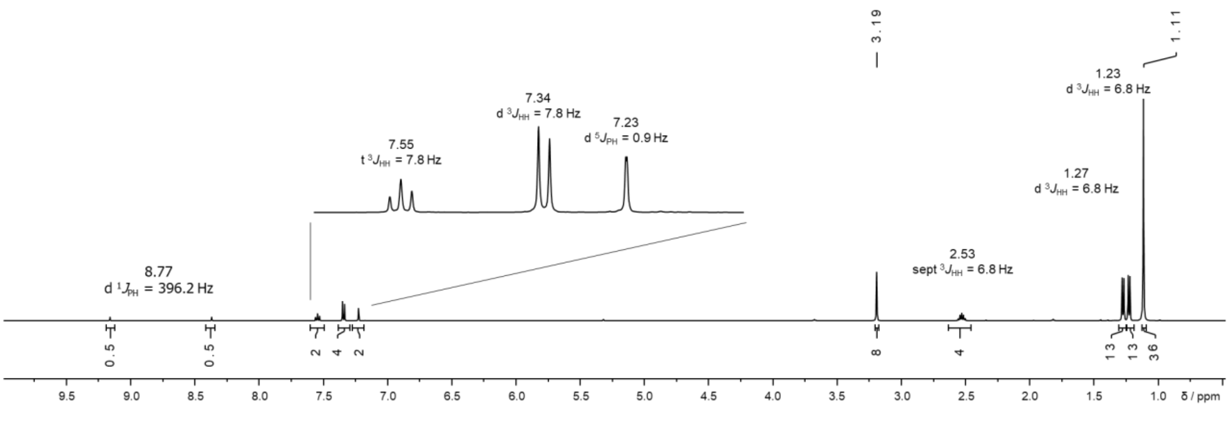

Figure S67: ^{1}H NMR spectrum (CD_2Cl_2, 500 MHz) of [**14**][SbF_6].

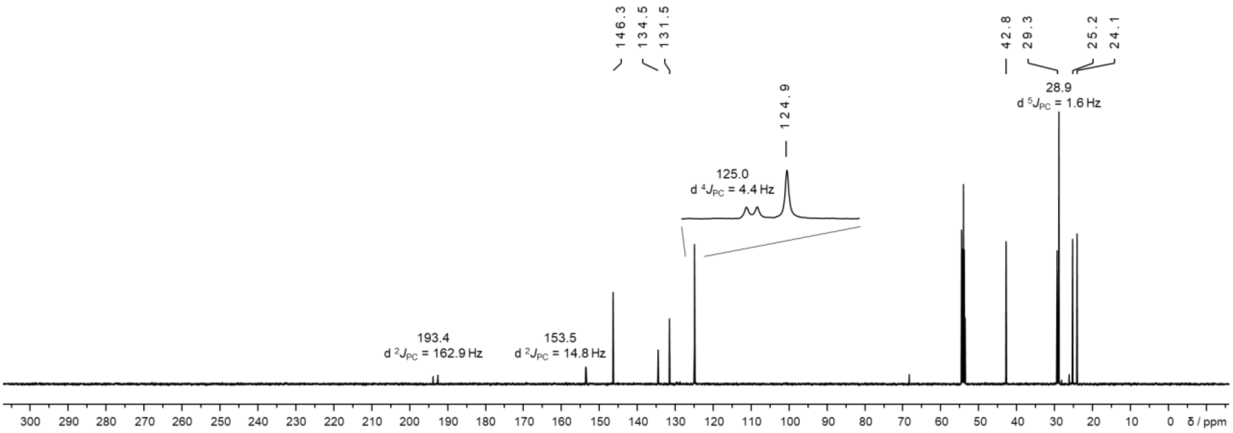

Figure S68: $^{13}C\{^1H\}$ NMR spectrum (CD_2Cl_2, 126 MHz) of [**14**][SbF_6].

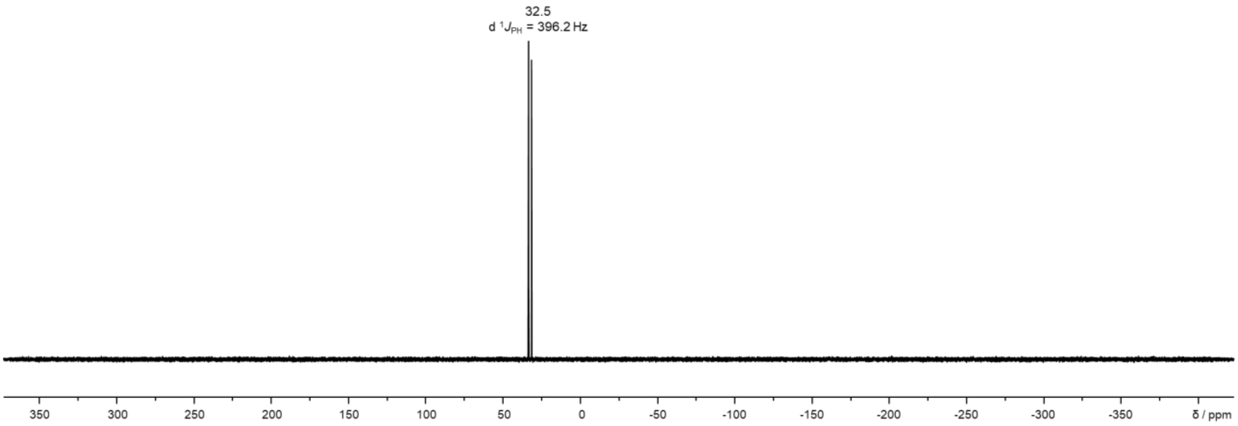

Figure S69: ^{31}P NMR spectrum (CD_2Cl_2, 202 MHz) of [**14**][SbF_6].

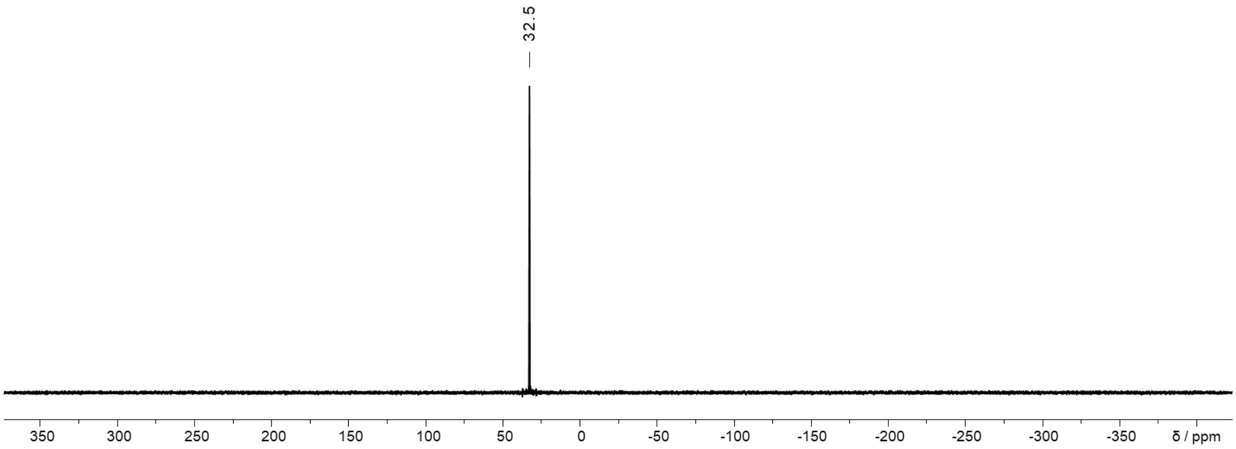

Figure S70: $^{31}P\{^1H\}$ NMR spectrum (CD_2Cl_2, 202 MHz) of [**14**][SbF_6].

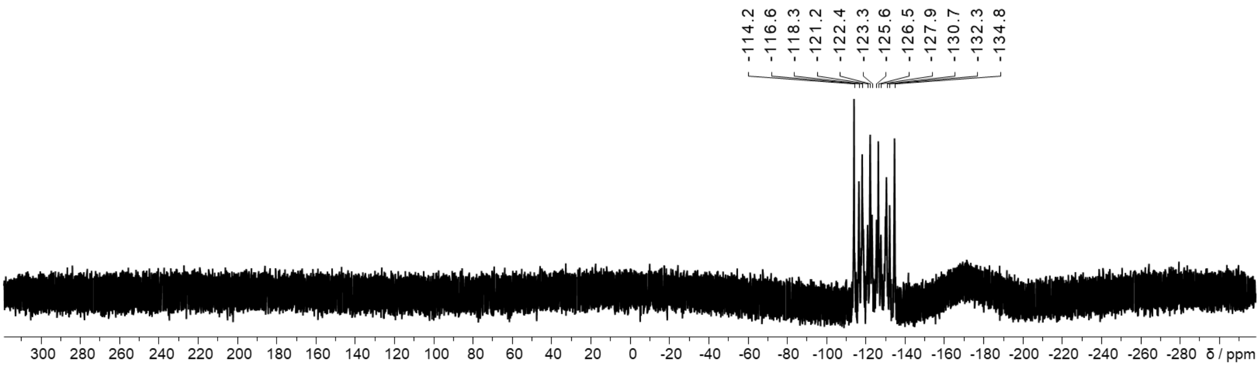

Figure S71: ^{19}F NMR spectrum (CD_2Cl_2, 471 MHz) of [**14**][SbF_6].

1.9 Synthesis of $(R^3)_2HPBH_3$ (15)

In a Schlenk tube, compound **3** (100 mg, 0.240 mmol, 1.00 eq.) was dissolved in THF (2 mL). At -78 °C, the borane tetrahydrofuran complex (1.0 M BH_3·THF in THF, 0.23 mL, 0.24 mmol, 1.0 eq.) was added. The reaction mixture was stirred for 16 hours at ambient temperature. All volatiles were removed *in vacuo*. The residue was dissolved in C_6D_6 and the resulting solution was analyzed by NMR spectroscopy, revealing complete conversion to **15** according to the ^{31}P and ^{1}H NMR spectrum. To purify the residue from contaminations*, the solid material was washed with *n*-hexane (2 x 1 mL) and subsequently dried *in vacuo.* Compound **15** was obtained as a white solid. If further purification is needed **15** can be recrystallized from a concentrated THF solution.

*Commercial samples of BH_3·THF typically contain impurities.[6] The ^{11}B NMR spectrum of the used stock solution showed three additional signals.

The thermal stability of **15** was probed by heating a solution of **15** in C_6D_6 to 110 °C in a Teflon-sealed NMR tube. After several days, PH_3 was detected in the ^{31}P NMR spectrum, indicating the decomposition by the formation of $HB(R^3)_2$.

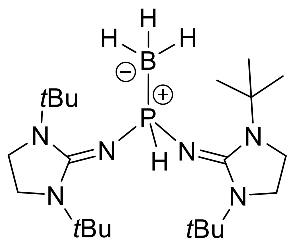

Yield 51% (54 mg, 0.12 mmol).
^{1}H NMR (400 MHz, C_6D_6): δ (ppm) = 8.52 (d, $^{1}J_{PH}$ = 366.0 Hz, 1H, PH), 2.72 (s, 8H, CH_2), 1.42 (s, 36H, CH_3), 1.29 (s, 3H, BH_3).
^{13}C{^{1}H} NMR (101 MHz, C_6D_6): δ (ppm) = 152.2 (d, $^{2}J_{PC}$ = 7 Hz, C=N), 54.4 (H_2C–CH_2), 42.2 (*C*(CH_3)$_3$), 28.8 (CH_3).
^{11}B NMR (128 MHz, C_6D_6): δ (ppm) = -32.8 (pseudo-p (dq), $^{1}J_{PB}$ = 95 Hz, $^{1}J_{BH}$ = 95 Hz).
^{11}B{^{1}H} NMR (128 MHz, C_6D_6): δ (ppm) = -32.8 (d $^{1}J_{PB}$ = 95 Hz).
^{31}P NMR (162 MHz, C_6D_6): δ (ppm) = 28.6 (dq, $^{1}J_{PH}$ = 366 Hz, $^{1}J_{PB}$ = 95 Hz).
^{31}P{^{1}H} NMR (162 MHz, C_6D_6): δ (ppm) = 28.6 (q, $^{1}J_{PB}$ = 95 Hz).
HRMS (ESI, positive): m/z calculated for $[C_{22}H_{49}BN_6P]^+$ (**15**+H)$^+$ 439.3844, found: 439.3835.
Crystals suitable for **SCXRD** were obtained from a saturated THF solution stored at -40 °C.

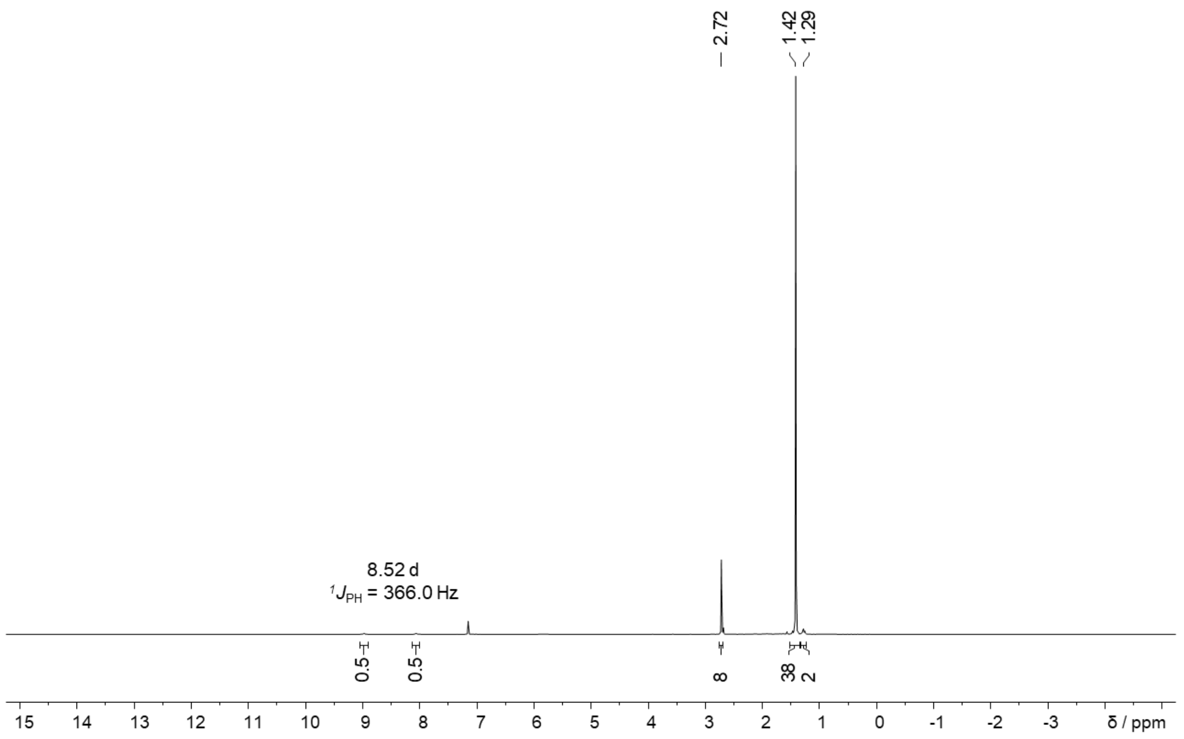

Figure S72: ^{1}H NMR spectrum (C_6D_6, 400 MHz) of **15**.

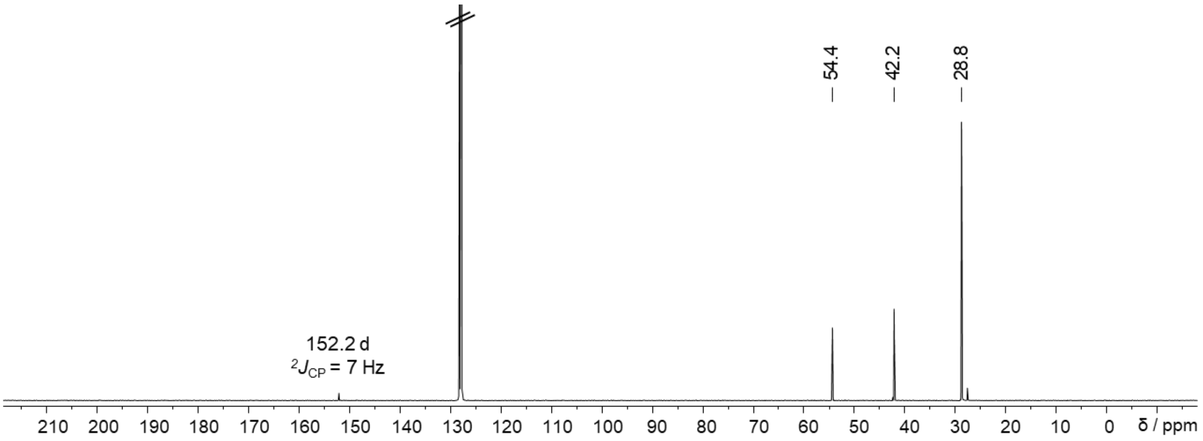

Figure S73: $^{13}C\{^{1}H\}$ NMR spectrum (C_6D_6, 101 MHz) of **15**.

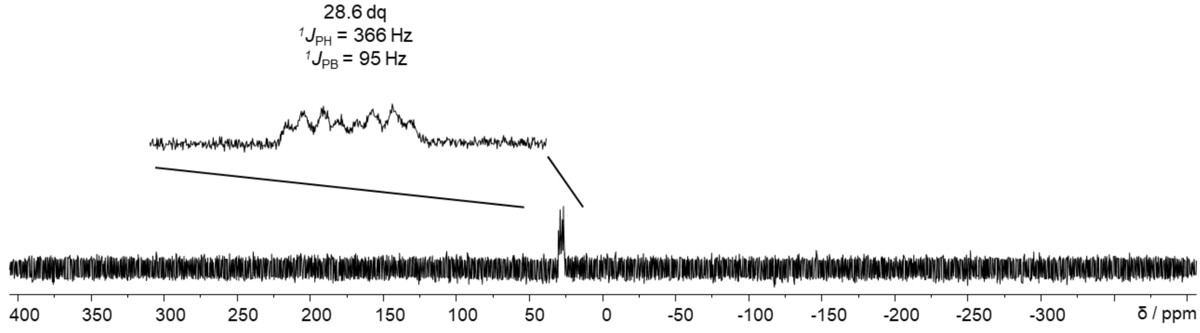

Figure S74: ^{31}P NMR spectrum (C_6D_6, 162 MHz) of **15**.

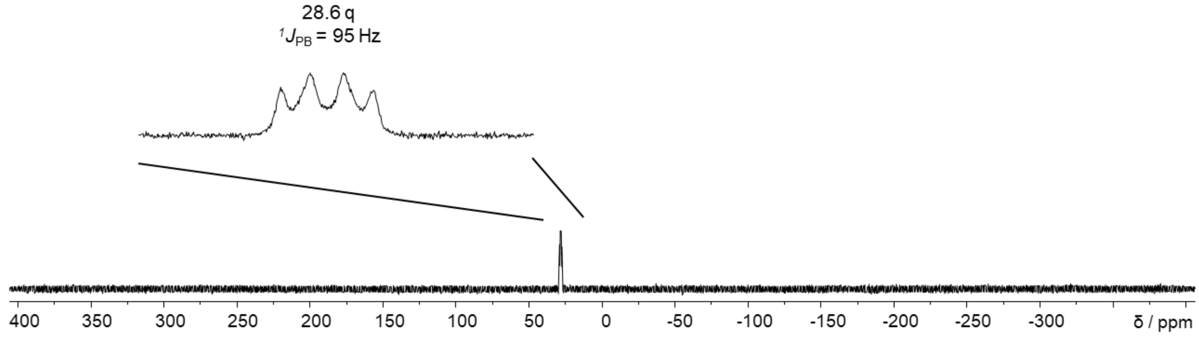

Figure S75: $^{31}P\{^{1}H\}$ NMR spectrum (C_6D_6, 162 MHz) of **15**.

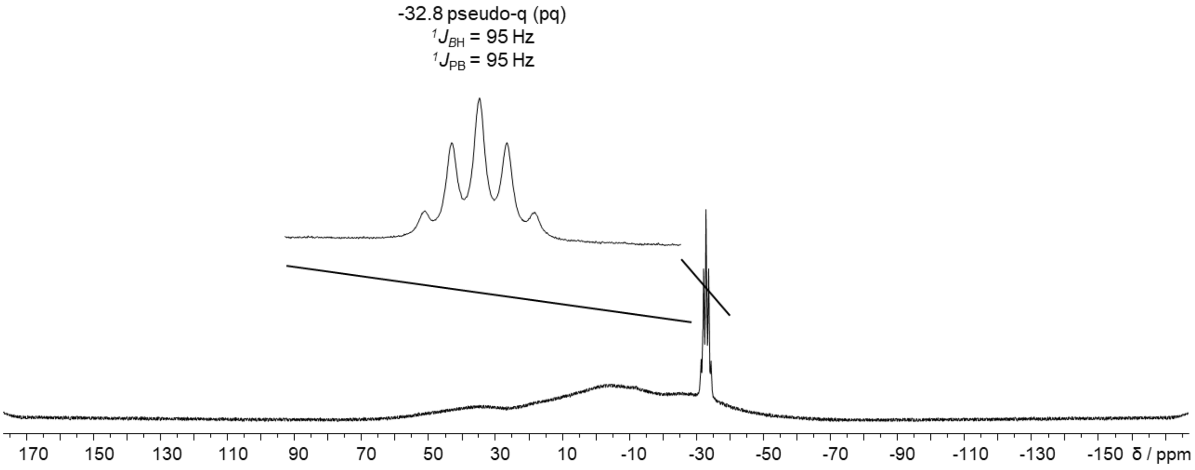

Figure S76: ^{11}B NMR spectrum (C_6D_6, 128 MHz) of **15**.

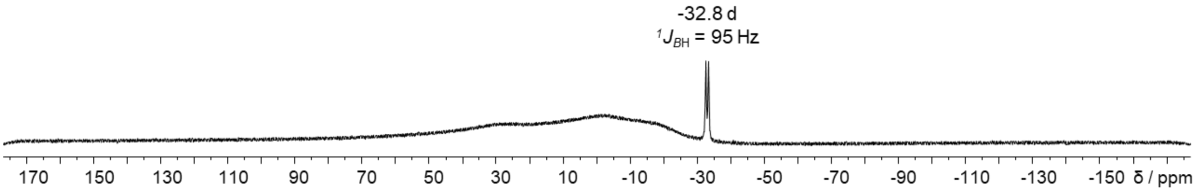

Figure S77: $^{11}B\{^{1}H\}$ NMR spectrum (C_6D_6, 128 MHz) of **15**.

1.10 Reaction with TEMPO - Hydrogen Atom Transfer

1.10.1 EPR Experiments

A stock solution of TEMPO ($12\cdot10^{-3}$ M in toluene, 0.15 mL, 0.18 µmol) was transferred into an EPR tube. Shortly before measurement, 10 mg of the respective phosphine (excess, 12 – 24 µmol) were added to the solution.

In the case of compound **1**, the EPR spectrum (Figure S78) immediately showed only a doublet signal attributed to the phosphinyl radical.*

For compound **2**, the EPR spectra (Figure S80) showed a slight decrease in the TEMPO signal which could be due to diffusion effects or a very slow reaction between **2** and TEMPO.

For compound **3**, it is observed in the EPR spectra that the TEMPO signal decreases and vanishes completely within 3 minutes. After heating the mixture to 100 °C, a doublet signal attributed to the phosphinyl radical appears.*

*The doublet signals are assigned to the corresponding phosphinyl radicals on the basis of comparable literature data (paramagnetic nature of a phosphinyl radical bearing two NHI substituents with a saturated backbone and Dipp groups at the endocyclic nitrogen atoms: $g = 2.005$, $a(^{31}P) = 78$ G, no coupling to ^{15}N nuclei observed).[7]

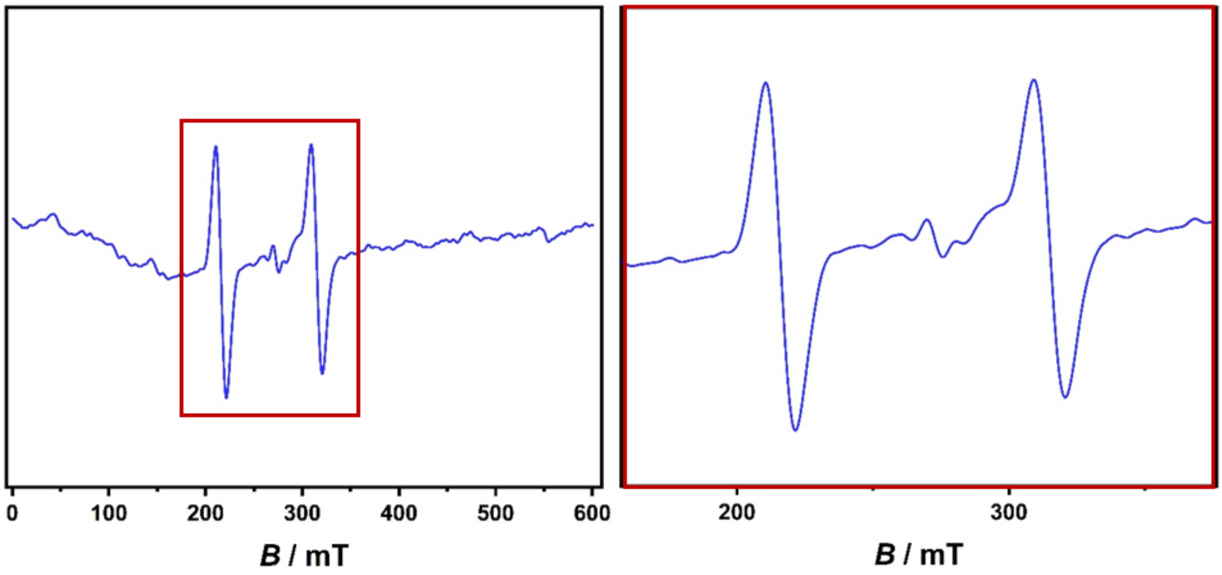

Figure S78: X-band EPR spectrum (toluene) of a reaction mixture containing TEMPO and **1** after 1 minute, phosphinyl radical $\cdot P(R^1)_2$ ($g = 2.003$, $a(^{31}P) = 66$ G).

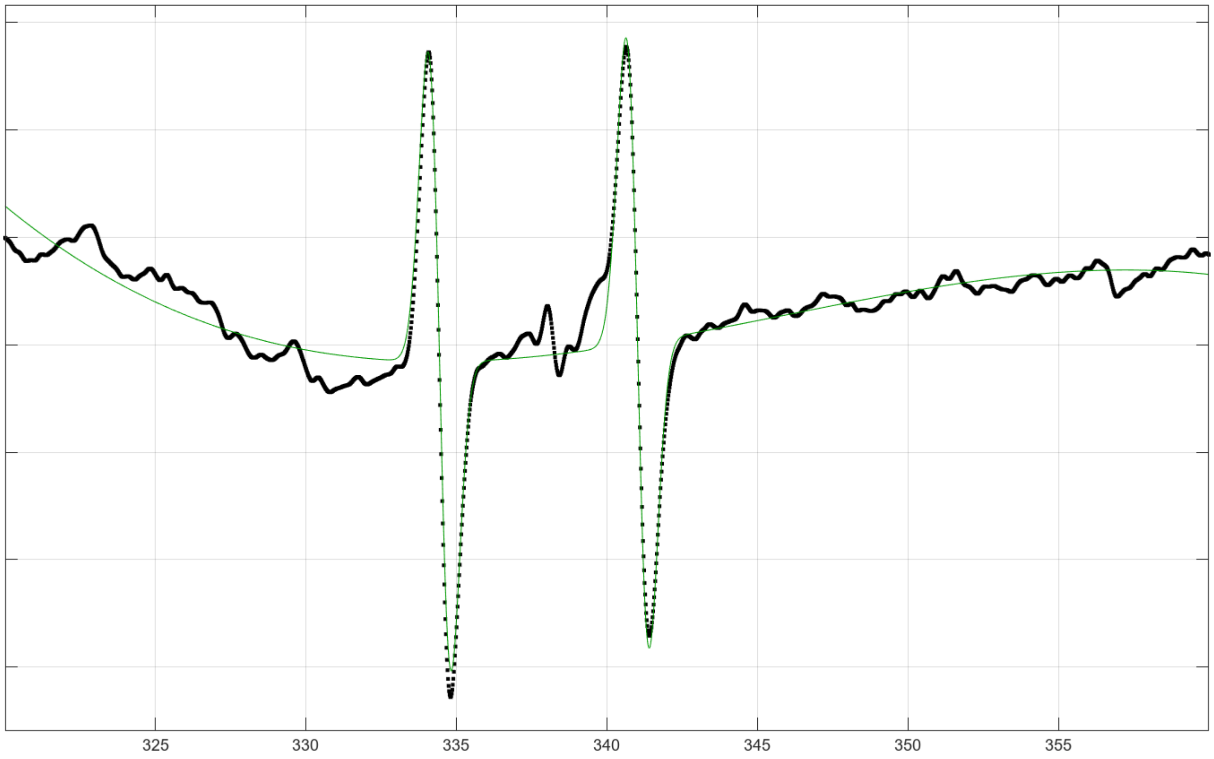

Figure S79: Overlayed simulated (g = 2.0033, $a(^{31}P)$ = 65.8 G) and measured (g = 2.003, $a(^{31}P)$ = 66 G) X-band EPR spectra of phosphinyl radical $\cdot P(R^1)_2$, x-axis: B / mT.

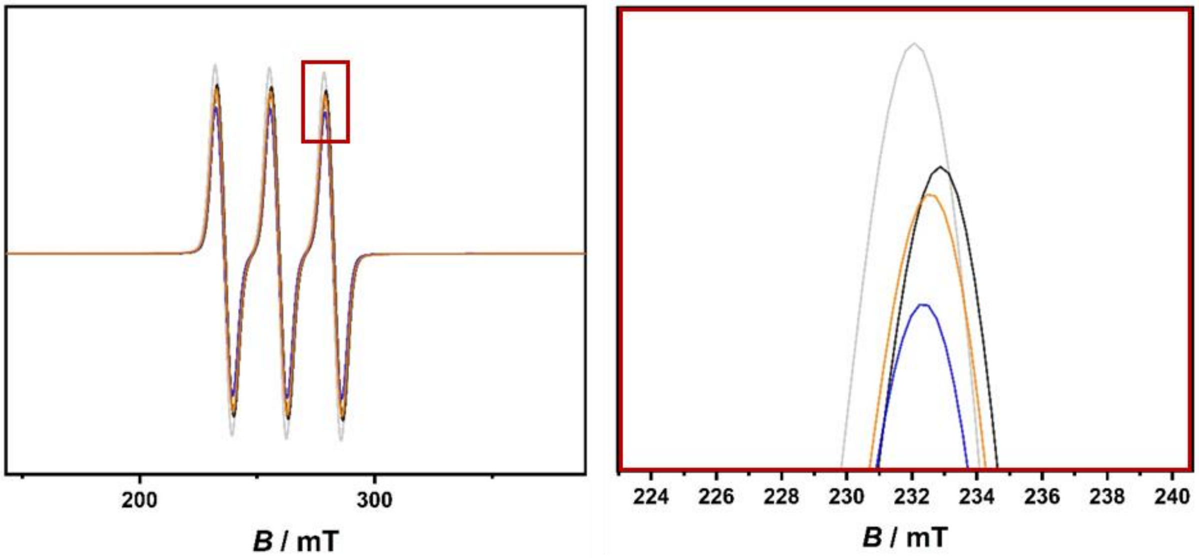

Figure S80: X-band EPR spectra (toluene) of a reaction mixture containing TEMPO and **2**, decrease of TEMPO signal (grey: TEMPO stock solution, black: after minutes, orange: after hours, blue: after 1 day).

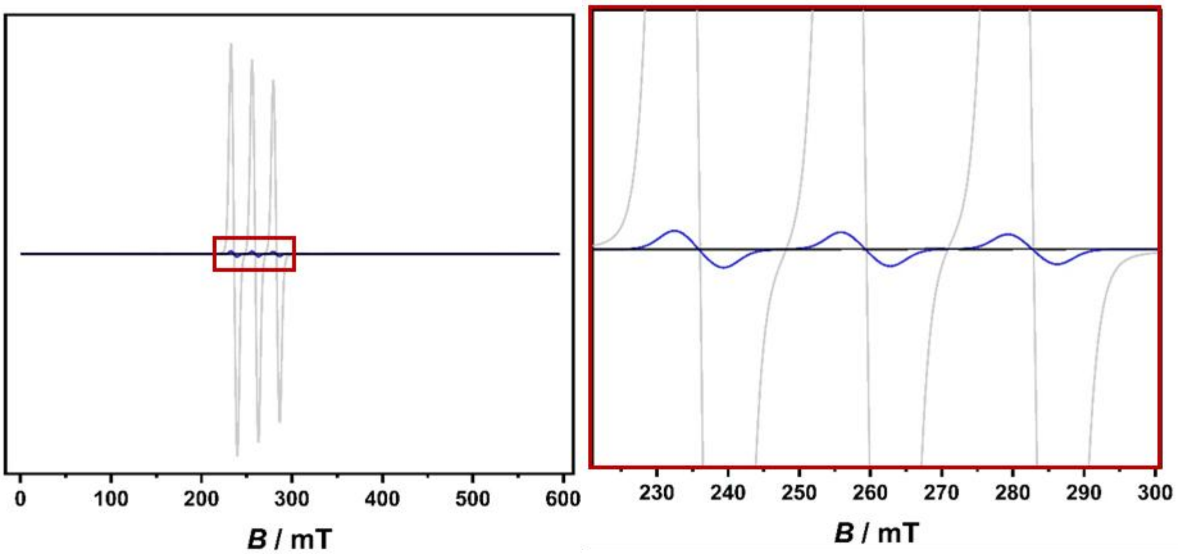

Figure S81: X-band EPR spectra (toluene) of a reaction mixture containing TEMPO and **3**, decrease of TEMPO signal (grey: after 1 minute, blue: after 2 minutes, black: after 3 minutes).

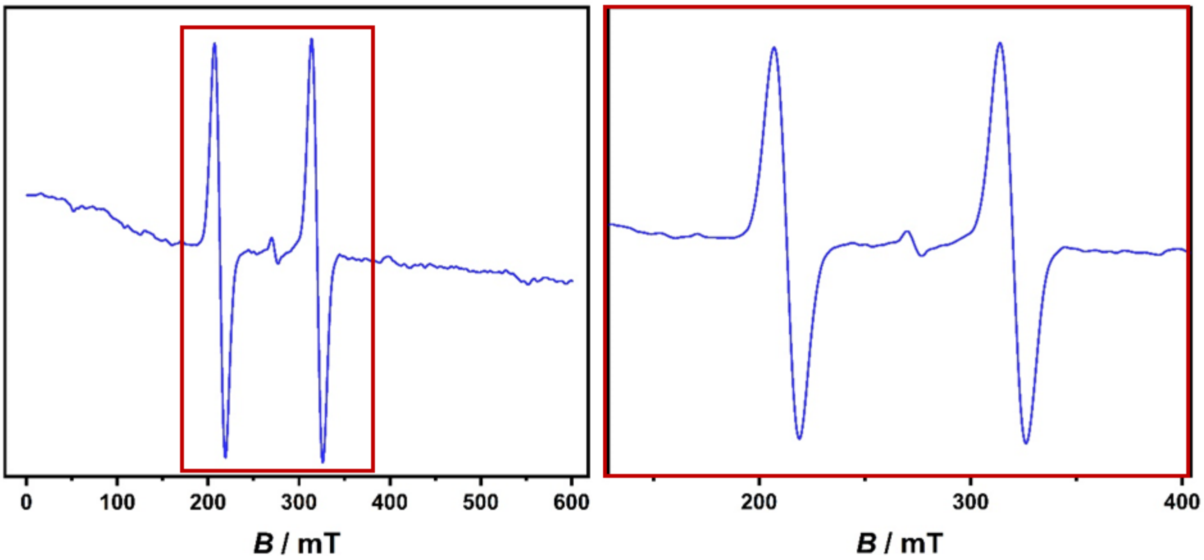

Figure S82: X-band EPR spectrum (toluene) of a reaction mixture containing TEMPO and **3** after heating to 100 °C, phosphinyl radical $\cdot P(R^2)_2$ (g = 2.003, a(^{31}P) = 71 G).

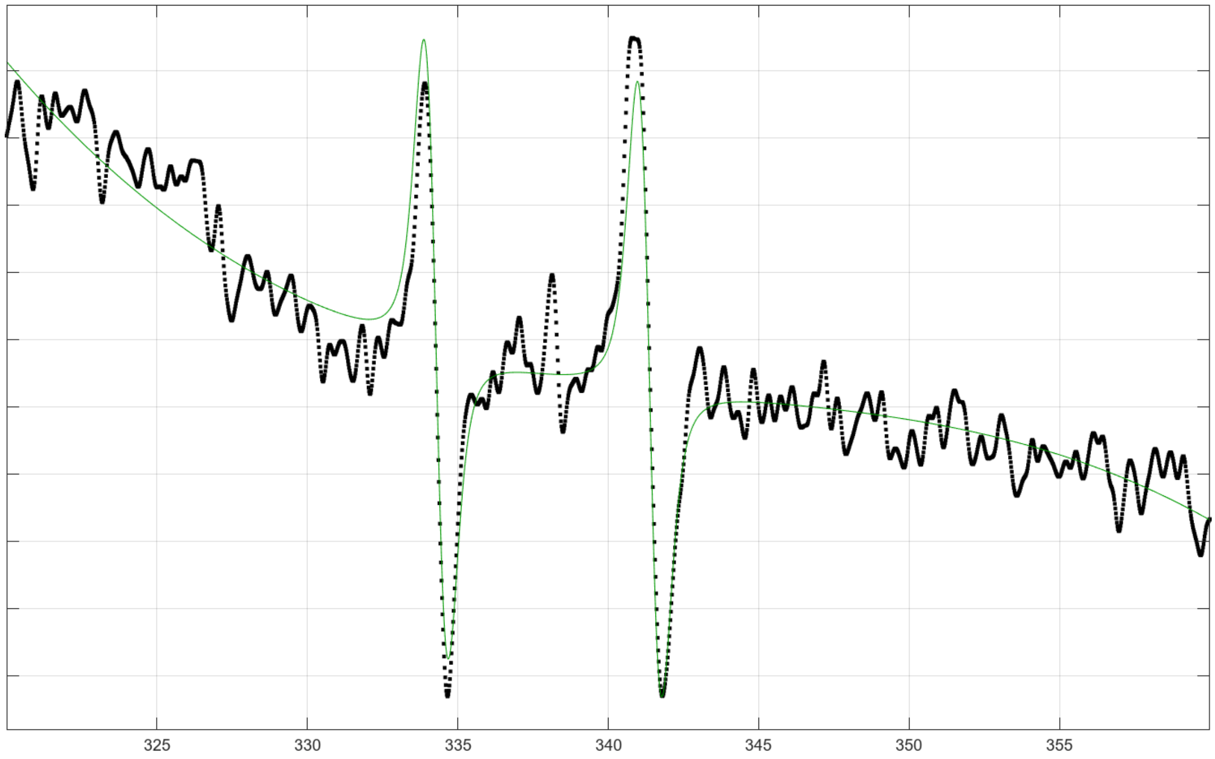

Figure S83: Overlayed simulated (g = 2.0028, $a(^{31}P)$ = 71.0 G) and measured (g = 2.003, $a(^{31}P)$ = 71 G) X-band EPR spectra of phosphinyl radical ·$P(R^3)_2$, x-axis: B / mT.

1.10.2 NMR Experiments

To TEMPO (5 mg, 0.033 mmol, 1.0 eq.) and the respective stoichiometric amount of phosphine (**1**: 14 mg, **2**: 28 mg, **3**: 14 mg; 0.033 mmol, 1.0 eq.), C_6D_6 (0.7 mL) was added.

To achieve complete conversion of **1** and **3**, TEMPO (5 mg, 0.033 mmol, 1.0 eq.) were added.

Phosphine **2** shows no reaction with TEMPO, even after 4 weeks and heating to 100 °C for 16 h.

For phosphine **1** and **3** we suggest the reaction pathway displayed in Figure S84, the underlying characterization data follows in the chapters 1.10.2.1 and 1.10.2.2.

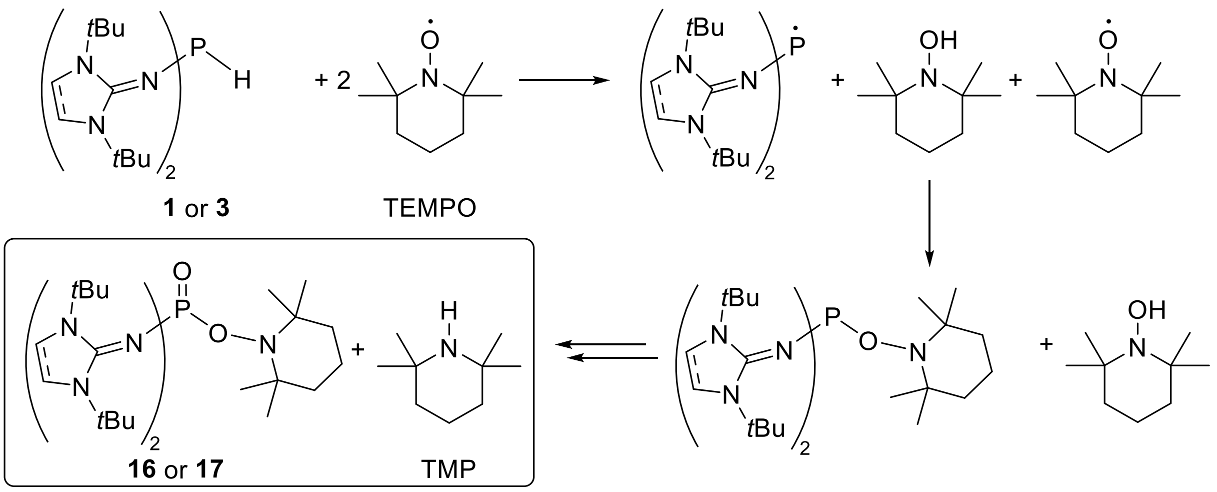

Figure S84: Proposed reaction pathway between **1** or **3** and 2 eq. TEMPO. The thermodynamic feasibility of hydrogen atom abstraction by the TEMPO radical, confirmed through BDFE calculations, provides strong support for the first step of the mechanism. The stability of NHI-substituted phosphinyl radicals at ambient temperature, as demonstrated by EPR spectroscopy, further validates the plausibility of radical coupling with the stable TEMPO radical. Two pathways seem possible for the subsequent oxygen atom transfer to give the products (characterized by NMR, HRMS and SCXRD): a) a stepwise deprotonation of TEMPOH by the phosphine followed by ion association and elimination of the secondary amine, or b) concerted reaction via a five-membered ring intermediate formed by the phosphine and TEMPOH.

1.10.2.1 Characterization data of **16**

Over 24 hours **1** reacts quantitatively with 2 equivalents of TEMPO. Looking at NMR data and an SCXRD study, we propose the formation of the following mixture (see Figure S84 for suggested mechanism):

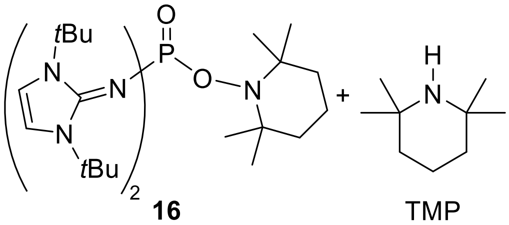

^{1}H NMR (400 MHz, C_6D_6): δ (ppm) = 6.08 (s, 4H, CH(NHI)), 1.69 (s, 36H, CH_3(NHI)), 1.61 (br., m, 20H, 2H: CH_2-C*H*$_2$-CH_2(TMP) + 12H: CH_3(TEMPO@P)+ 6H: CH_2-CH_2-CH_2(TEMPO@P)), 1.24 (m, 4H, C*H*$_2$-CH_2-C*H*$_2$(TMP)), 1.07 (s, 12H, CH_3(TMP)), 0.31 (br., 1H, NH).

^{31}P NMR (162 MHz, C_6D_6): δ (ppm) = -25.0 (s).

^{31}P{^{1}H} NMR (162 MHz, C_6D_6): δ (ppm) = -25.0 (s).

Crystals of **16** suitable for **SCXRD** were obtained by diffusion of *n*-hexane into a saturated toluene solution at -40 °C.

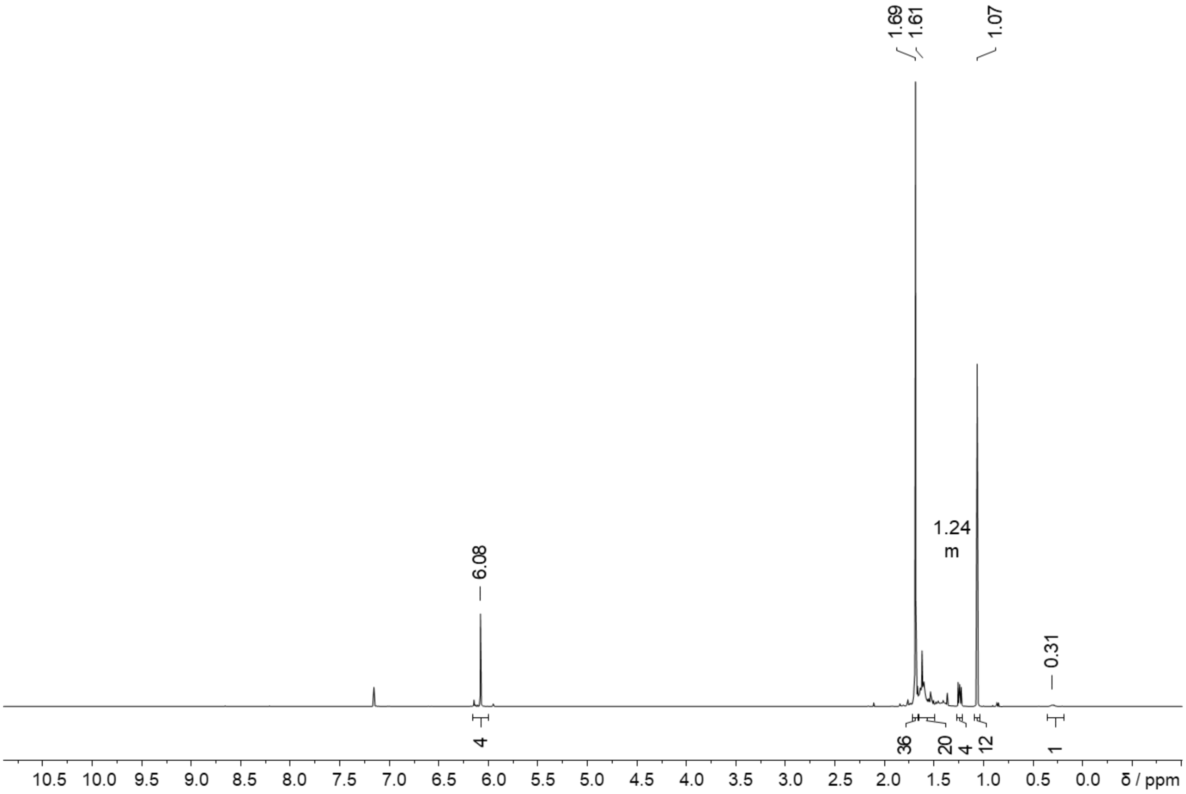

Figure S85: ^{1}H NMR spectrum (C_6D_6, 400 MHz) of **16** and TMP.

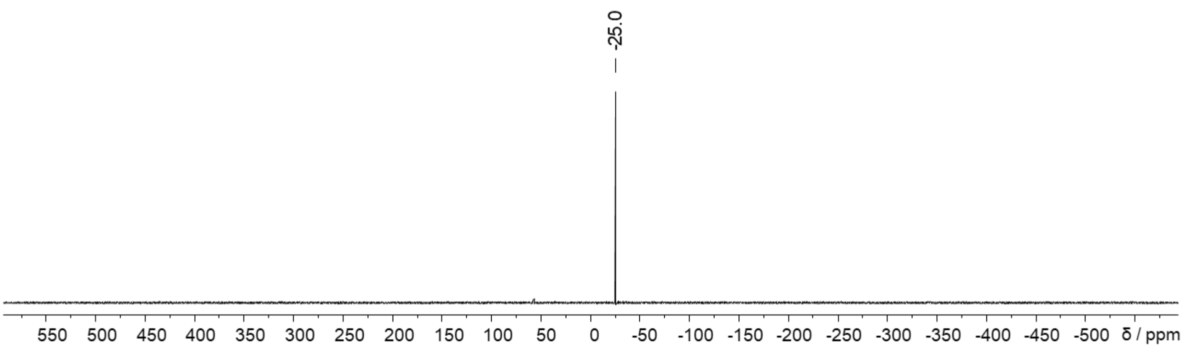

Figure S86: ^{31}P NMR spectrum (C_6D_6, 162 MHz) of **16** (and TMP).

1.10.2.2 Characterization data of **17**

Over 3 weeks **3** reacts quantitatively with 2 equivalents of TEMPO. Analogue to the reactivity of **1**, looking at NMR data and HRMS data, we propose the formation of the following mixture (see Figure S84 for suggested mechanism):

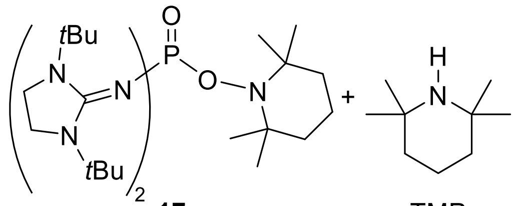

17 TMP

^{1}H NMR (400 MHz, C_6D_6): *δ* (ppm) = 2.77 (s, 8H, CH_2(NHI)), 1.57 (s, 36H, CH_3(NHI)), 1.57 (br., 20H, 2H: CH_2-C*H*$_2$-CH_2(TMP) + 12H: CH_3(TEMPO@P)+ 6H: CH_2-CH_2-CH_2(TEMPO@P)), 1.24 (m, 4H, C*H*$_2$-CH_2-C*H*$_2$(TMP)), 1.07 (s, 12H, CH_3(TMP)), 0.31 (br., 1H, NH).

^{31}P NMR (162 MHz, C_6D_6): *δ* (ppm) = -28.1 (s).

^{31}P{^{1}H} NMR (162 MHz, C_6D_6): *δ* (ppm) = -28.1 (s).

HRMS (ESI, positive): m/z calculated for $[C_{31}H_{63}N_7O_2P]^+$ (**17**+H)$^+$ 596.4775, found: 596.4753.

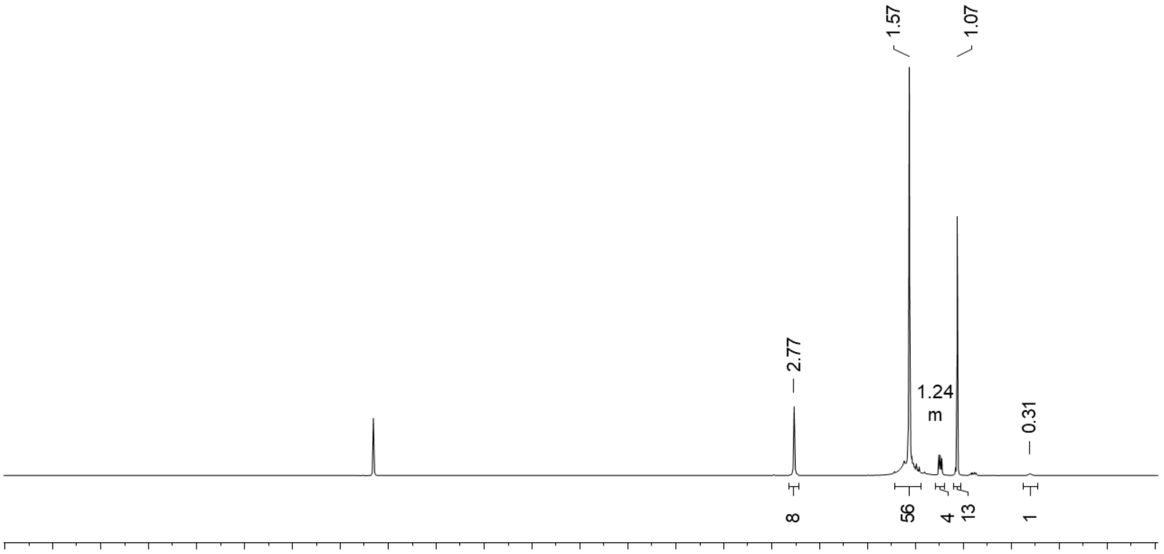

Figure S87: ^{1}H NMR spectrum (C_6D_6, 400 MHz) of **17** and TMP.

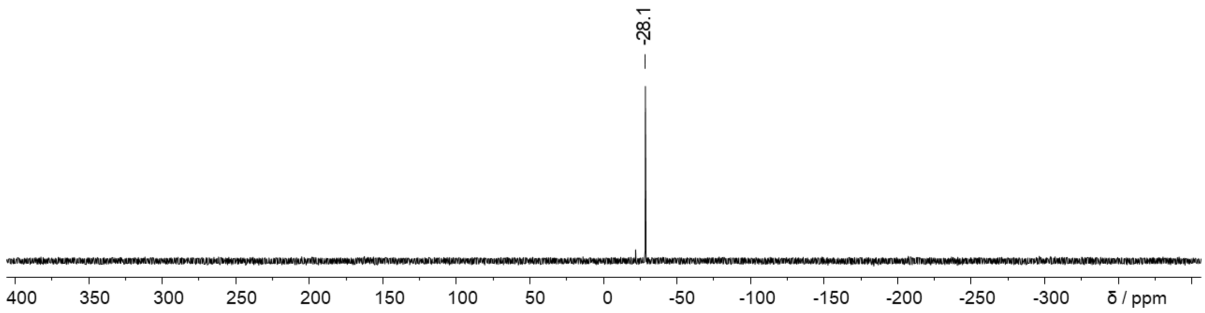

Figure S88: ^{31}P NMR spectrum (C_6D_6, 162 MHz) of **17** (and TMP).

1.11 Reactions with Trityl chloride – Hydride Transfer

To a mixture of chlorotriphenylmethane (trityl chloride, 15 mg, 0.054 mmol, 1.0 eq.) and either **1** or **3** (**1**: 22.6 mg, **3**: 22.9 mg, 0.0540 mmol, 1.00 eq.), 0.5 mL of C_6D_6 was added. The immediate formation of a white solid and a yellow (**1**) or orange (**3**) solution was observed. The precipitate was separated by filtration and subsequently dissolved in CD_3CN.

NMR analysis revealed that the solids are the respective phosphenium ions (PR_2^+),[1,2] as confirmed by their characteristic signals in the ^{31}P NMR spectra. In the C_6D_6 solution (filtrate), triphenylmethane was detected, evidenced by the characteristic resonance of the $R_3C\mathit{H}$ proton at 5.43 ppm.[8]

(Compound **2** also reacts with trityl chloride. However, the reaction proceeds very slow and is less distinct than the reaction with **1** and **3**.)

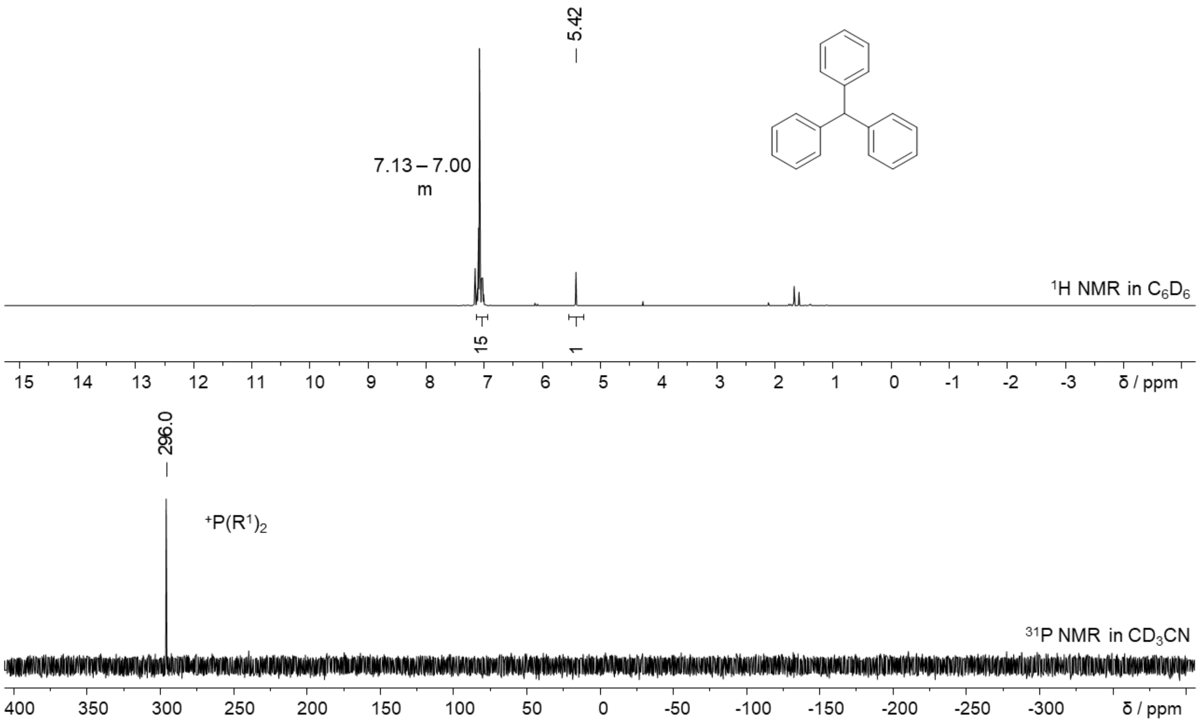

Figure S89: Relevant NMR spectra of the products of the reaction between **1** and trityl chloride.

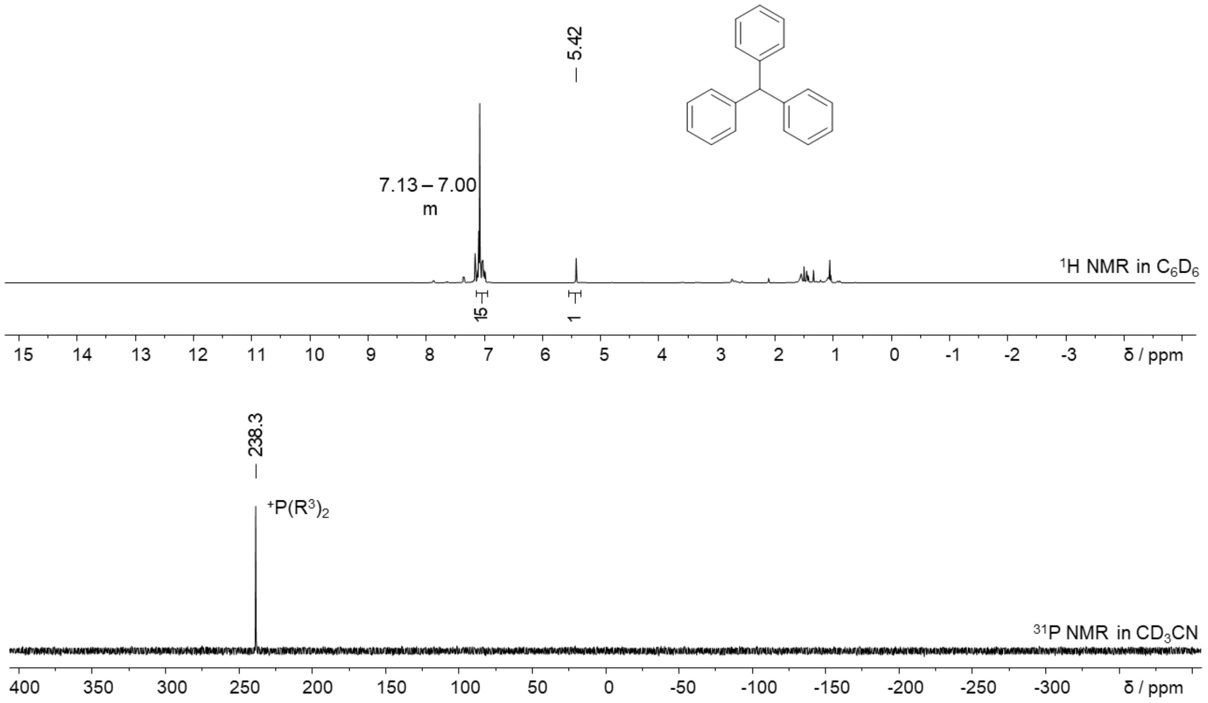

Figure S90: Relevant NMR spectra of the products of the reaction between **3** and trityl chloride.

1.12 Deprotonation Attempts

$NaBH_4$

The respective phosphine (**1**: 55.6 mg, **2**:110.7 mg, **3**: 56.1 mg, 0.132 mmol, 1.00 eq.) and $NaBH_4$ (5.0 mg, 0.13 mmol, 1.0 eq.) were added to a Teflon-sealed NMR tube and suspended in C_6D_6 (0.7 mL). Via ^{31}P NMR spectroscopy the reaction was monitored. Heating up to 100 °C led to no reaction. CD_3CN (a few drops) was added to achieve better solubility of $NaBH_4$. After weeks at ambient temperature **1** and **3** decomposed by reaction with CD_3CN and **2** showed no reaction.

KO*t*Bu

The respective phosphine (**1**: 19.6 mg, **3**: 19.8 mg, 0.047 mmol, 1.0 eq.) and KO*t*Bu (5.2 mg, 0.047 mmol, 1.0 eq.) were added to a Teflon-sealed NMR tube and dissolved in THF (0.7 mL). The reaction was monitored via ^{31}P NMR spectroscopy. Both phosphines showed no reaction up to 70 °C.

LDA

The respective phosphine (**1**: 19.6 mg, **2**: 39.1 mg, **3**: 19.8 mg, 0.047 mmol, 1.0 eq.) and LDA (5.0 mg, 0.047 mmol, 1.0 eq.) were added to a Teflon-sealed NMR tube and dissolved in THF (0.7 mL). The reaction was monitored via ^{31}P NMR spectroscopy. The ^{31}P NMR spectra of the reaction between **1** and LDA show slowly decreasing signals between 50-110 ppm (Figure S91). After 5 days at ambient temperature, roughly 20% of the secondary phosphine had reacted to around 10 new species in the mentioned chemical shift range. The ^{31}P NMR spectra of the reaction between **2** and LDA show a slowly decreasing signal in form of a doublet at 49 ppm with a PH coupling constant of J_{PH} = 178.8 Hz (Figure S92). After 5 days at ambient temperature roughly 20% of the secondary phosphine had reacted. The ^{31}P NMR spectra of the reaction between **3** and LDA show two slowly decreasing signals in form of singlets at 91 and 98 ppm (Figure S93). After 5 days at ambient temperature, roughly 60% of the secondary phosphine had reacted.

n-BuLi

The respective phosphine (**1**: 67.3 mg, **2**:134.0 mg, **3**: 67.9 mg, 0.16 mmol, 1.0 eq.) was added to a Teflon-sealed NMR tube and dissolved in THF. *n*-BuLi (1.6 M in *n*-hexane, 0.1 mL, 0.16 mmol, 1.0 eq.) was added. The reaction was monitored via ^{31}P NMR spectroscopy. The ^{31}P NMR spectra of the reaction between **1** or **3** and *n*-BuLi show more than 2 new species which do not show PH coupling (Figure S94 and Figure S96). The ^{31}P NMR spectrum of the reaction between **2** and *n*-BuLi shows 2 new species which still show PH coupling (Figure S95).

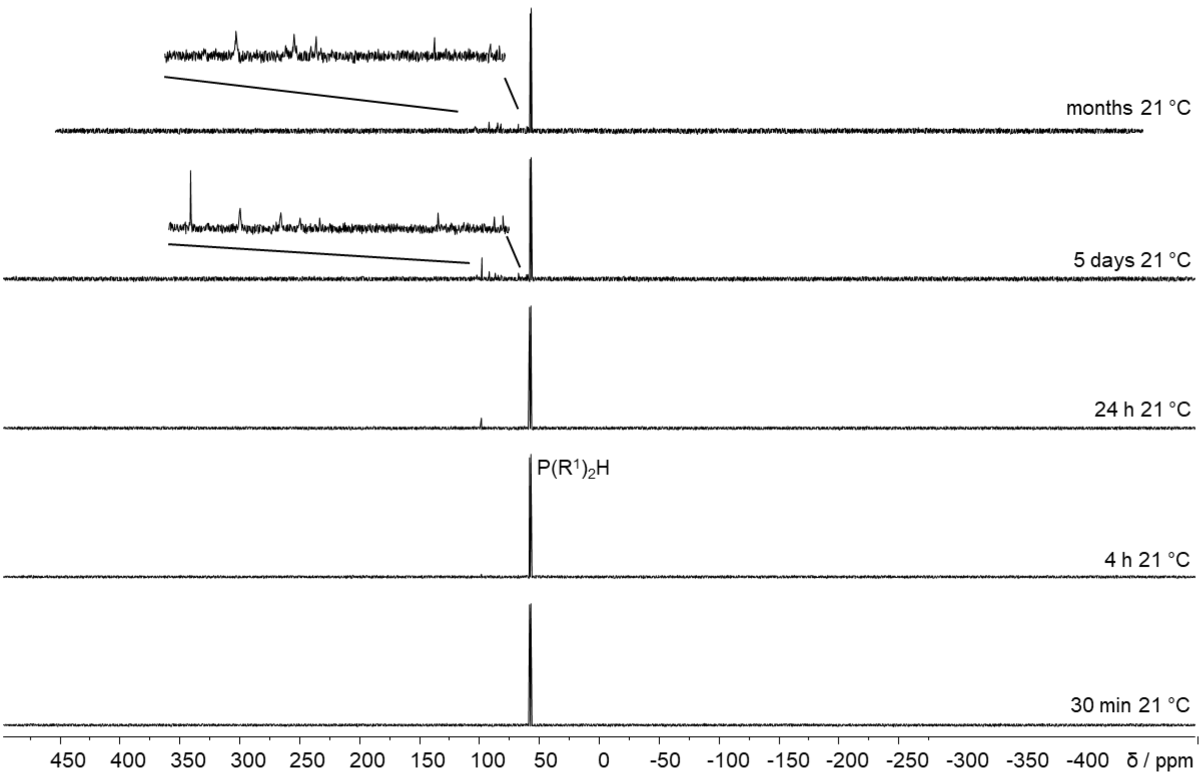

Figure S91: ^{31}P NMR spectra (THF, 162 MHz) of the reaction mixture of **1** and LDA.

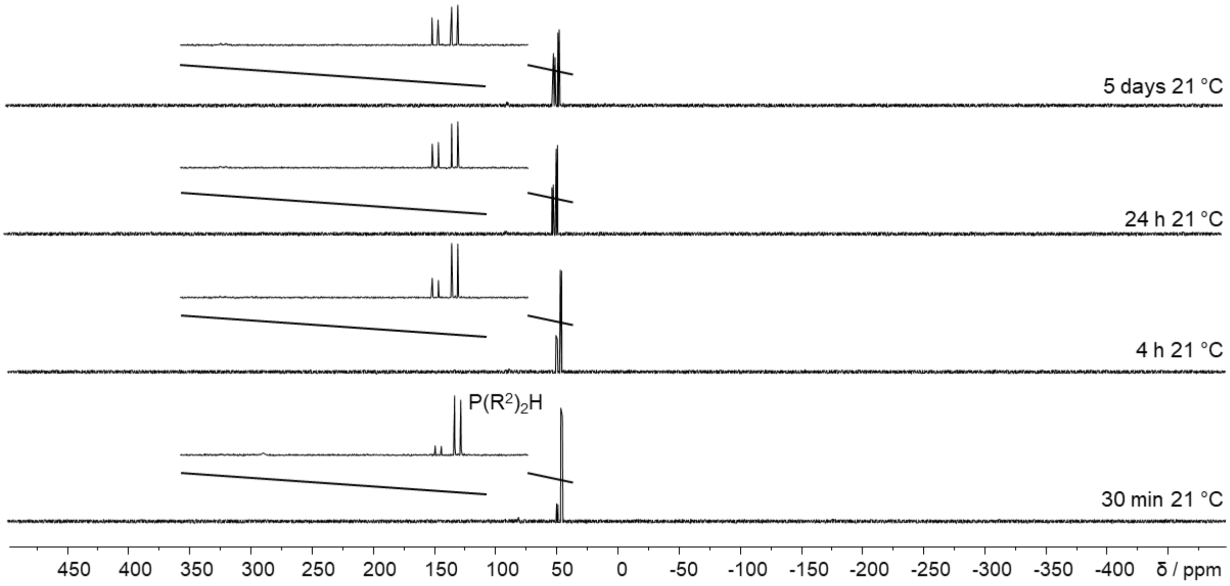

Figure S92: ^{31}P NMR spectra (THF, 162 MHz) of the reaction mixture of **2** and LDA.

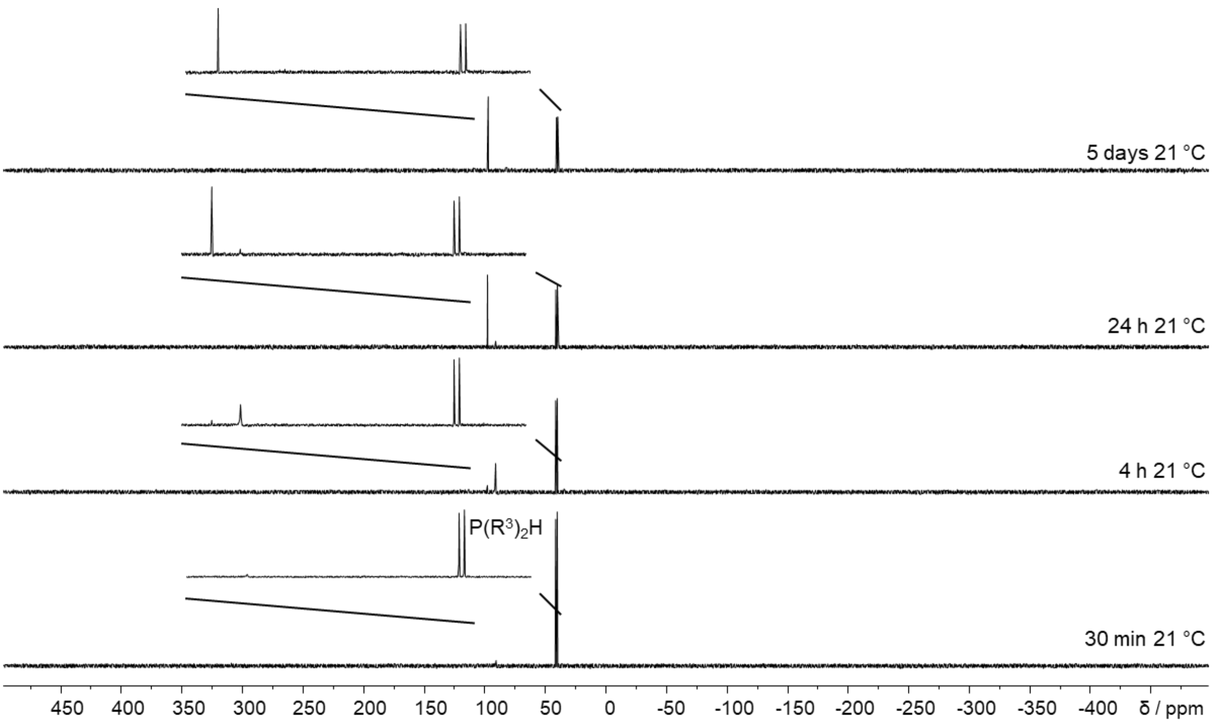

Figure S93: ^{31}P NMR spectra (THF, 162 MHz) of the reaction mixture of **3** and LDA.

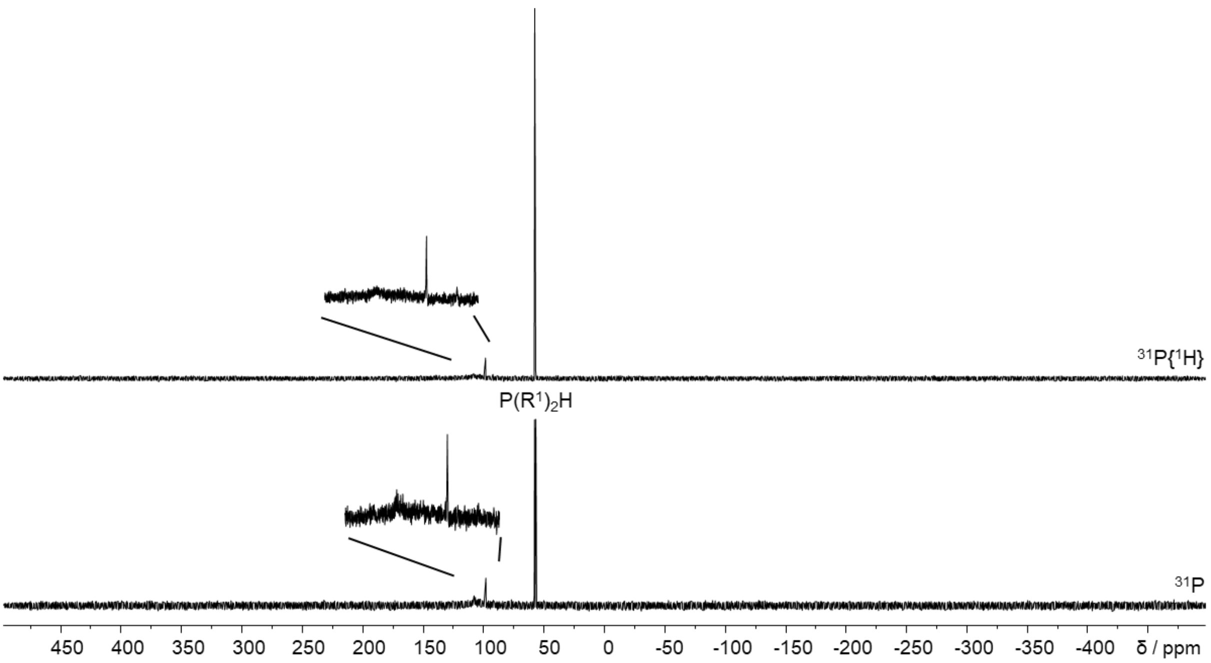

Figure S94: ^{31}P NMR spectra (THF, 162 MHz) of the reaction mixture of **1** and *n*-BuLi after 30 min at ambient temperature.

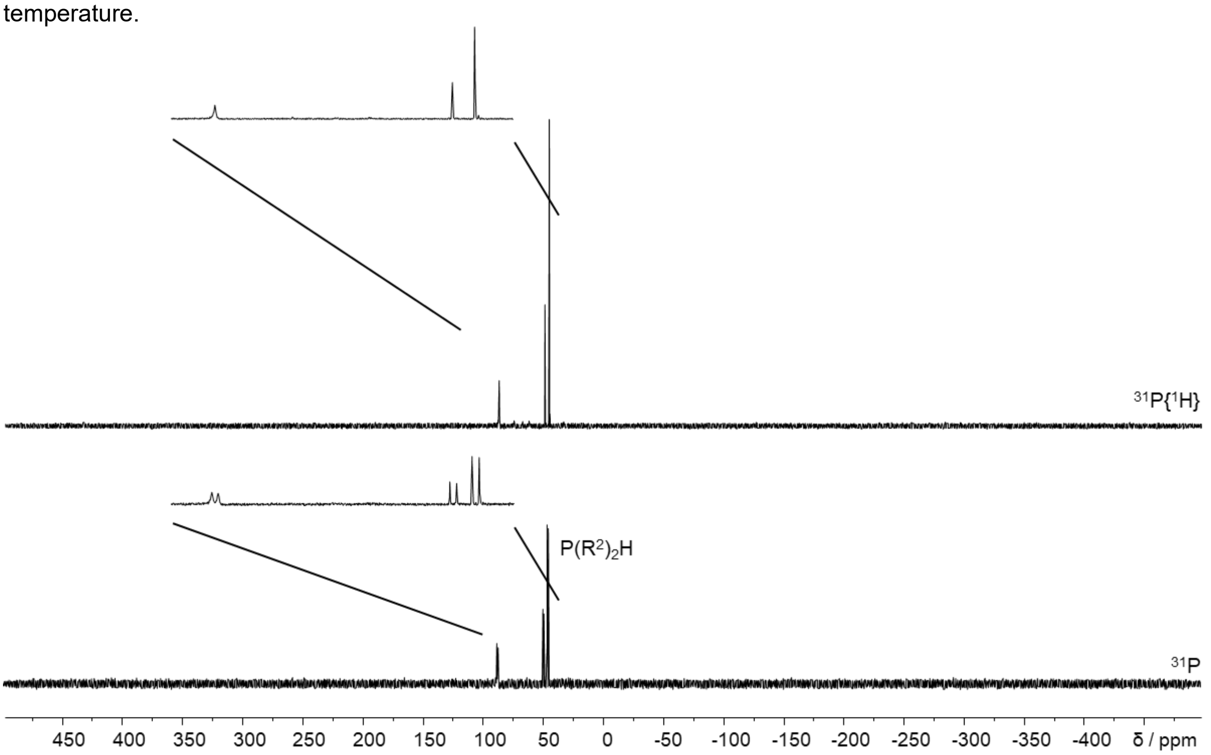

Figure S95: ^{31}P NMR spectra (THF, 162 MHz) of the reaction mixture of **2** and *n*-BuLi after 30 min at ambient temperature.

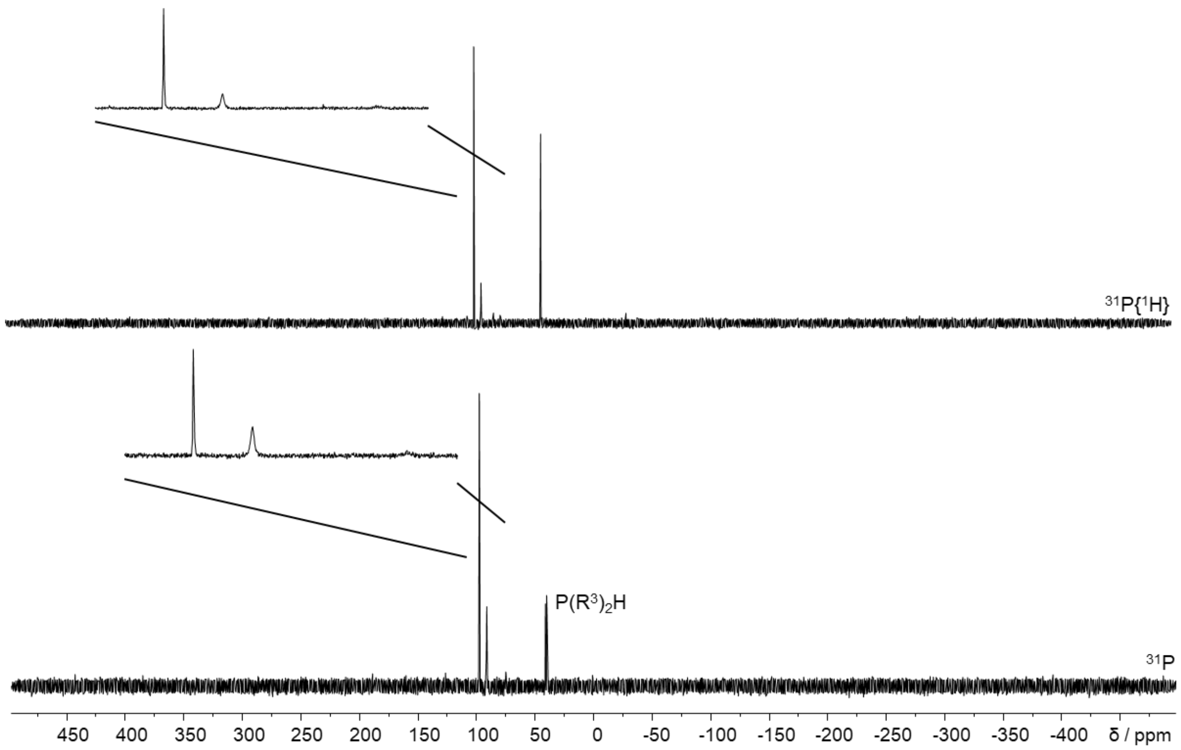

Figure S96: ^{31}P NMR spectra (THF, 162 MHz) of the reaction mixture of **3** and *n*-BuLi after 30 min at ambient temperature.

1.13 Reactions with Alkynes - Hydrophosphinations

1.13.1 Reaction with Phenylacetylene

To a PTFE-sealed NMR tube containing either compound **1** or **3** (**1**: 63.1 mg, **3**: 63.7 mg; 0.150 mmol, 1.00 eq.), a stoichiometric amount of phenylacetylene (0.50 M in toluene, 0.30 mL, 0.15 mmol, 1.0 eq.) was added. The reaction progress was monitored using quantitative ^{31}P NMR spectroscopy. Once the secondary phosphine was fully converted, attempts were made to isolate the main product (see below).

1.13.1.1 Reaction of **1** with phenylacetylene yielding alkenylphosphine **18**

Quantitative ^{31}P NMR spectra are depicted in Figure S97. In this case complete conversion was achieved after 18 hours and the main product represents ~85%* of the phosphorus species. After removing the volatiles *in vacuo*, the main product was isolated via crystallization from a saturated Et_2O solution at -40 °C. It was identified as alkenylphosphine **18**, the hydrophosphination product of **1** and phenylacetylene in which the phenyl group and phosphorus center are in *cis* confirmation, the (Z)-isomer. Compound **18** was characterized by NMR spectroscopy, high resolution mass spectrometry and SCXRD.

*The conversion varied between 60% and 100% in several executions. Other species, apart from the main product **18**, were not identified. However, prolonged reaction times led to the decomposition into multiple phosphorus species (Figure S107).

Notably, the NMR signal assignment of the alkenyl group was done using 2D NMR spectra: The ^{13}C NMR resonance at δ = 132.0 ppm shows coupling to the ^{1}H NMR resonance at δ = 7.01 ppm in the ^{1}H ^{13}C HSQC spectrum. The ^{13}C NMR resonance at δ = 147.7 ppm shows coupling to the ^{1}H NMR resonance at δ = 7.12 ppm in the ^{1}H ^{13}C HSQC spectrum.

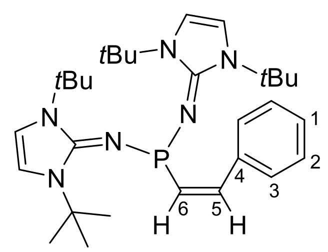

^{1}H NMR (400 MHz, C_6D_6): δ (ppm) = 8.01 - 7.99 (m, 2H, $H_{arom.}$, H2), 7.25 – 7.21 (m, 2H, $H_{arom.}$, H3), 7.12 (dd, $^{2}J_{PH}$ = 1.5 Hz, $^{3}J_{HH}$ = 13.1 Hz, H_{alkene}, H6), 7.08 – 7.03 (m, 1H, $H_{arom.}$, H1), 7.01 (dd, $^{3}J_{PH}$ = 22.7 Hz, $^{3}J_{HH}$ = 13.1 Hz, H_{alkene}, H5), 6.10 (s, 4H, $N(CH)_2N$), 1.60 (s, 36H, CH_3).

^{13}C{^{1}H} NMR (101 MHz, C_6D_6): δ (ppm) = 147.7 (d, $^{1}J_{PC}$ = 13.0 Hz, C6), 143.5 (d, $^{2}J_{PC}$ = 28.8 Hz, C=N), 140.5 (s, C4), 132.0 (d, $^{2}J_{PC}$ = 21.3 Hz, C5), 130.5 (C2), 130.4 (C2), 128.2 (C3), 126.5 (C1), 108.0 ($N(CH)_2N$), 55.2 (*C*$(CH_3)_3$), 29.4 (d, $^{5}J_{PC}$ = 8.4 Hz, CH_3).

^{31}P NMR (162 MHz, C_6D_6): δ (ppm) = 63.5 (d, $^{3}J_{PH}$ = 22.7 Hz).

^{31}P{^{1}H} NMR (162 MHz, C_6D_6): δ (ppm) = 63.5 (s).

HRMS (ESI, positive): m/z calculated for $[C_{30}H_{48}N_6P]^+$ (**18**+H)$^+$ 523.3673, found: 523.3662.

Crystals suitable for **SCXRD** were obtained from diffusing *n*-hexane into a saturated toluene solution at -40 °C.

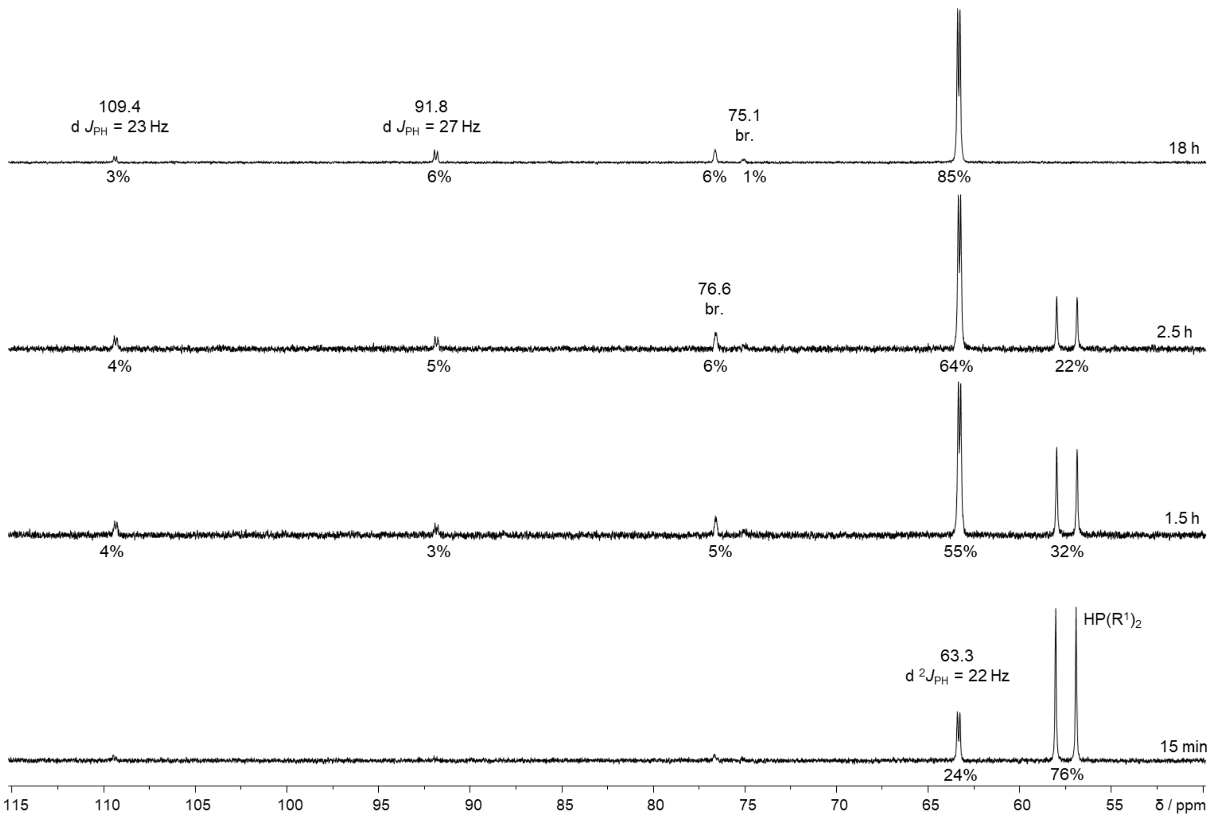

Figure S97: Quantitative ^{31}P NMR spectra (toluene, 162 MHz, relaxation delay D_1 = 25 sec) of the reaction mixture of **1** and phenylacetylene, selected chemical shift range: no other resonances between 400 to -400 ppm.

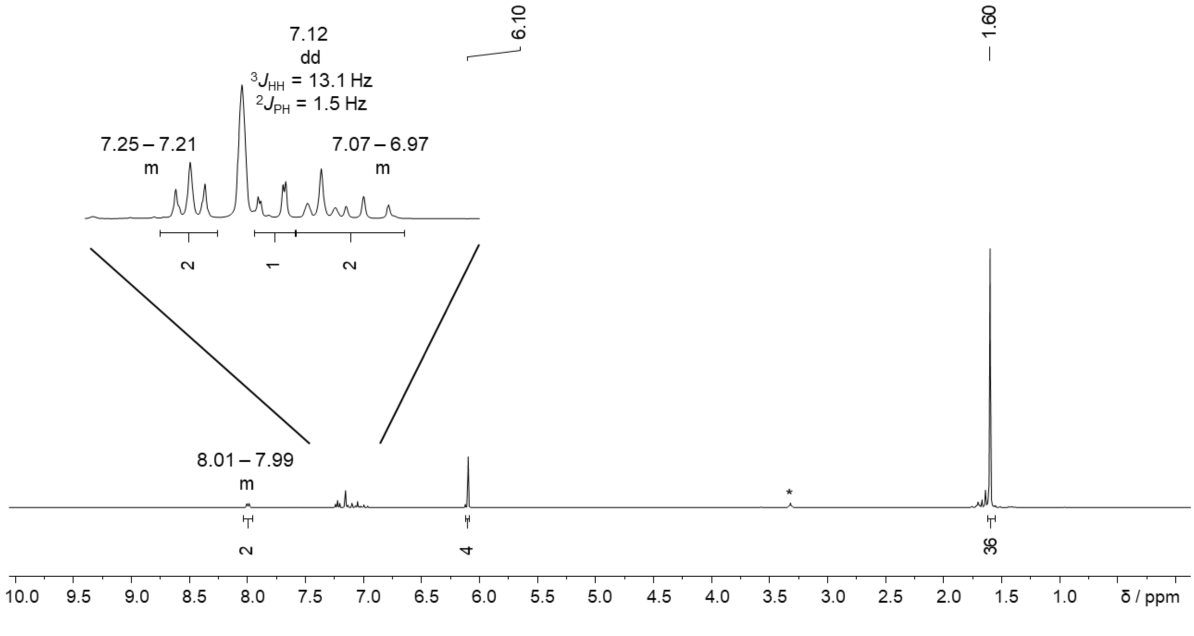

Figure S98: ^1H NMR spectrum (C_6D_6, 400 MHz) of **18**, for a detailed look and breakdown of the multiplets in the shift range from 7.15 to 6.95 ppm see Figure S99**,** asterisk (*) marks residual THF.

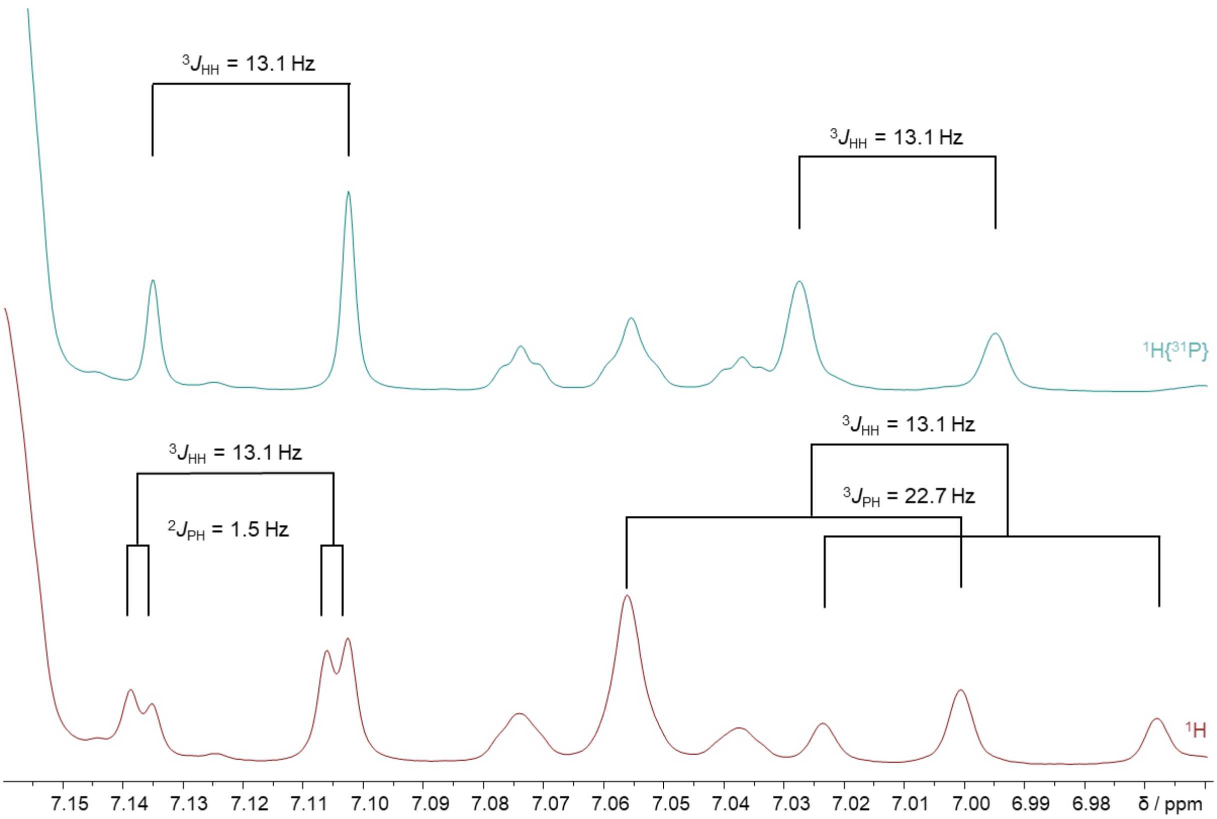

Figure S99: $^1H\{^{31}P\}$ (top) and ^1H (bottom) NMR spectrum (C_6D_6, 400 MHz) of **18**, detailed look and breakdown of the multiplets arising from the alkenyl protons, the non-marked multiplet in the middle is assigned to an aromatic proton.

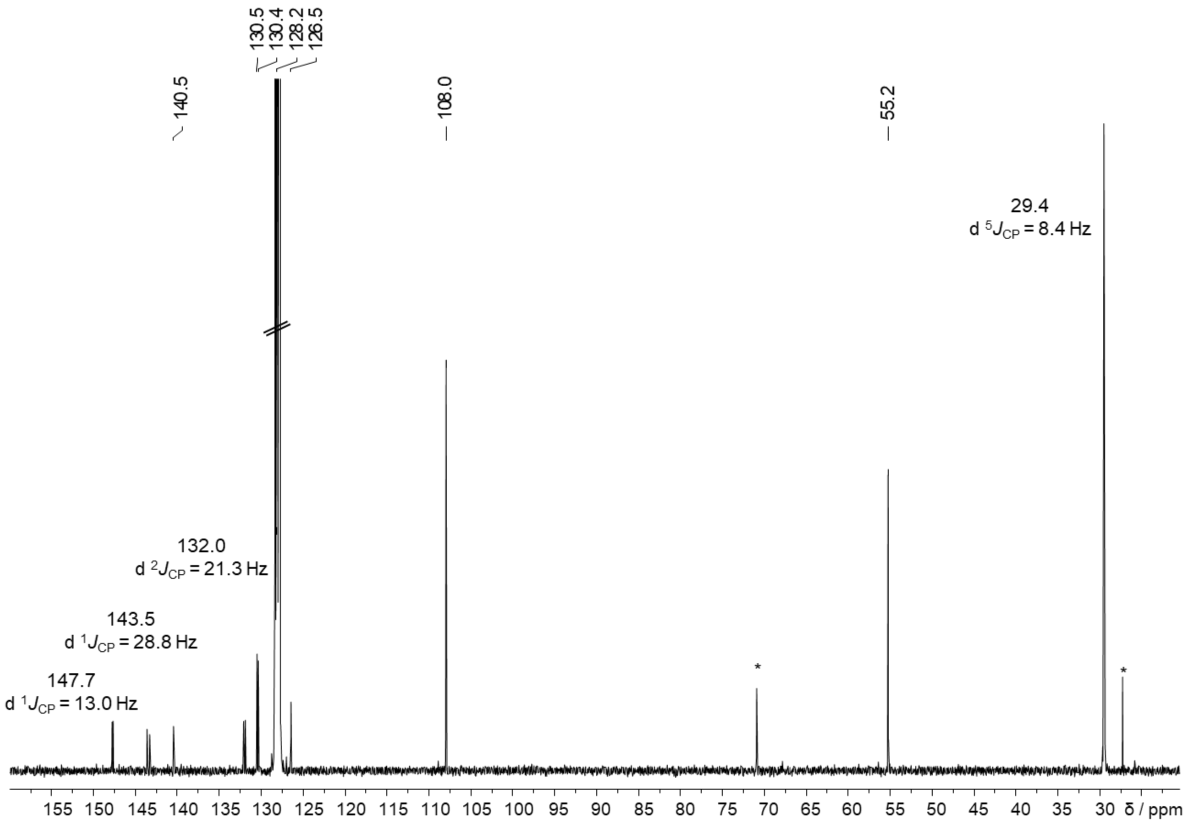

Figure S100: $^{13}C\{^1H\}$ NMR spectrum (C_6D_6, 101 MHz) of **18**, asterisks (*) mark residual THF.

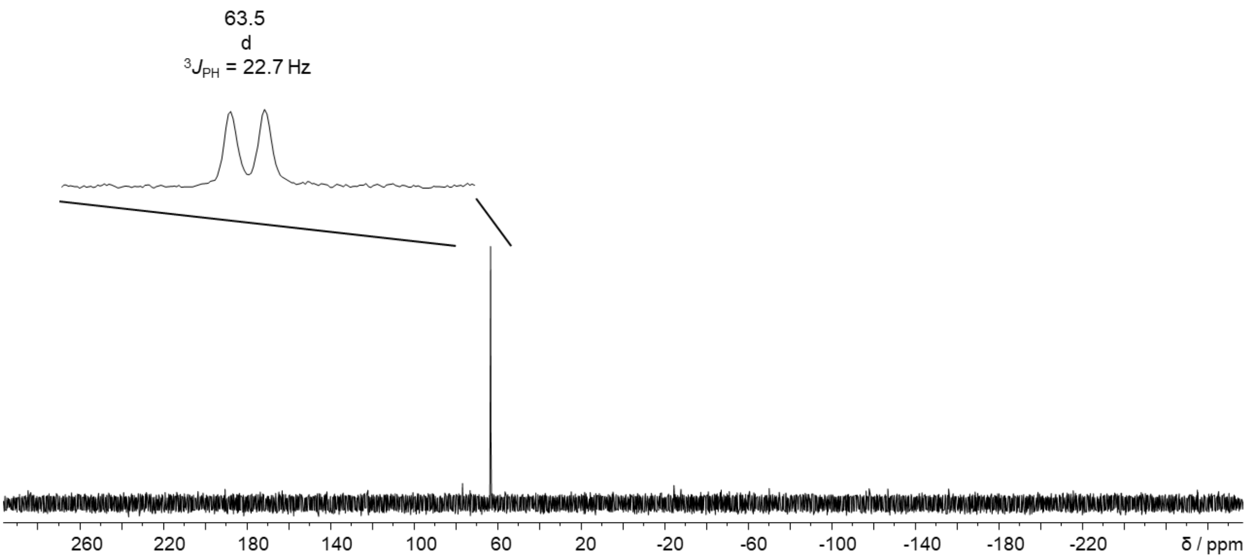

Figure S101: ^{31}P NMR spectrum (C_6D_6, 162 MHz) of **18**.

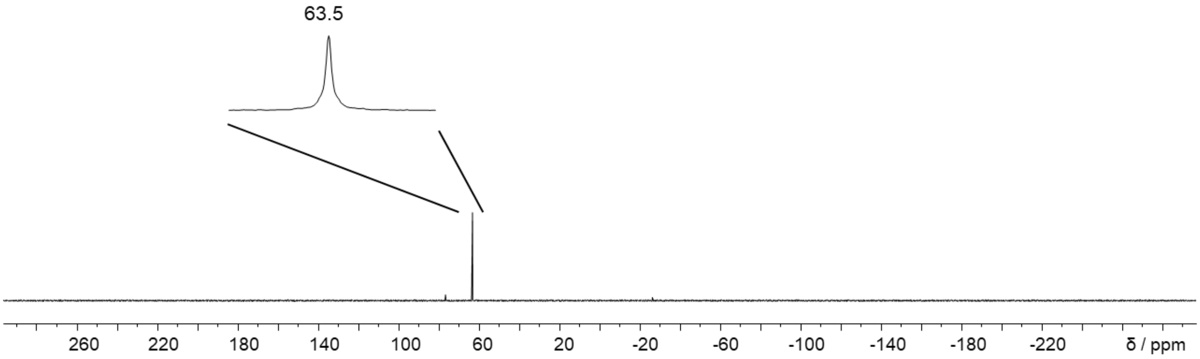

Figure S102: ^{31}P{^{1}H} NMR spectrum (C_6D_6, 162 MHz) of **18**.

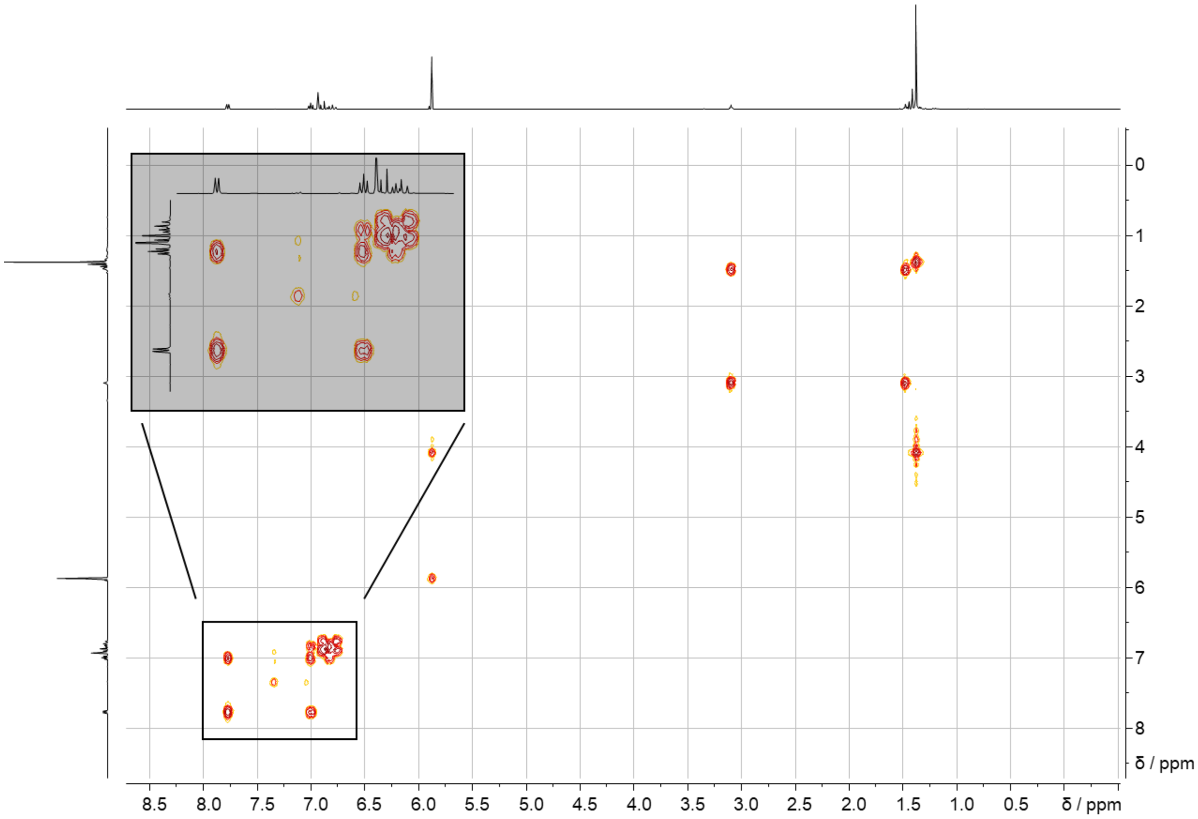

Figure S103: ^{1}H ^{1}H COSY 2D NMR spectrum (C_6D_6, 400 MHz, 400 MHz) of **18**.

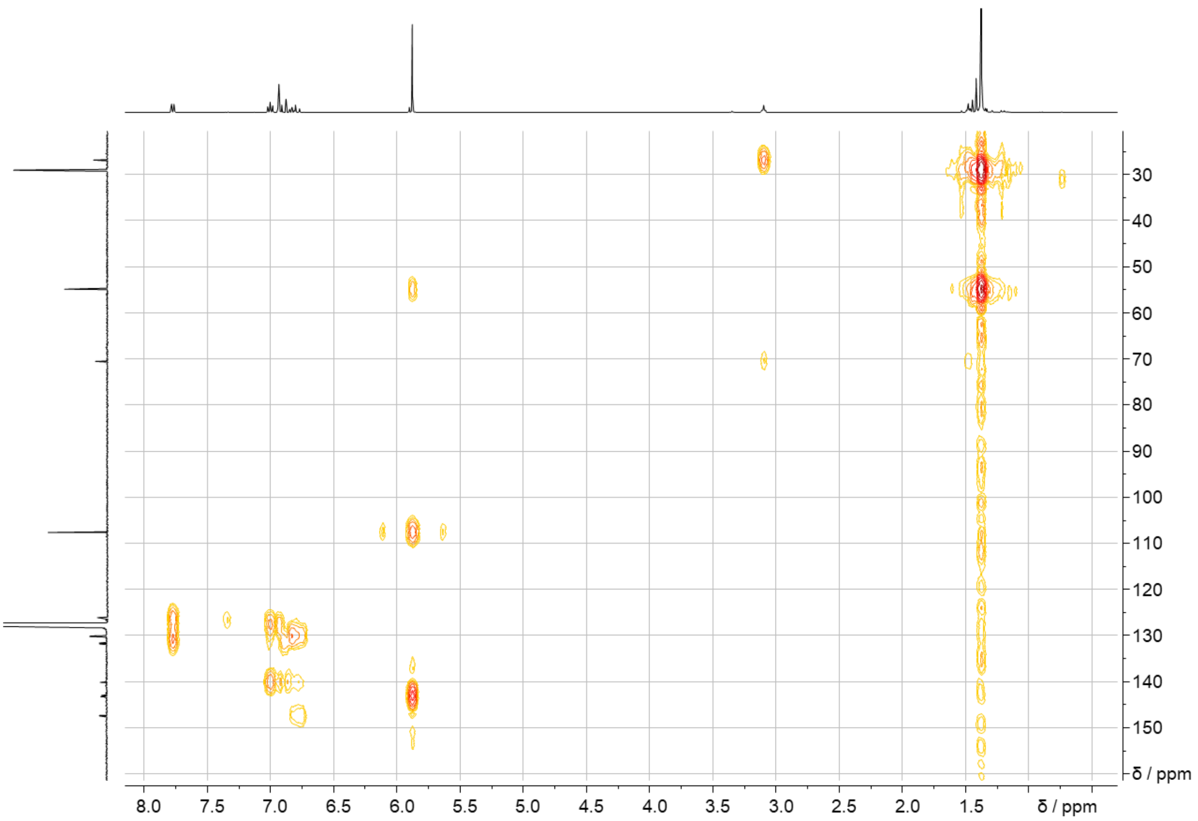

Figure S104: ^{1}H ^{13}C{^{1}H} HMBC 2D NMR spectrum (C_6D_6, 400 MHz, 101 MHz) of **18**.

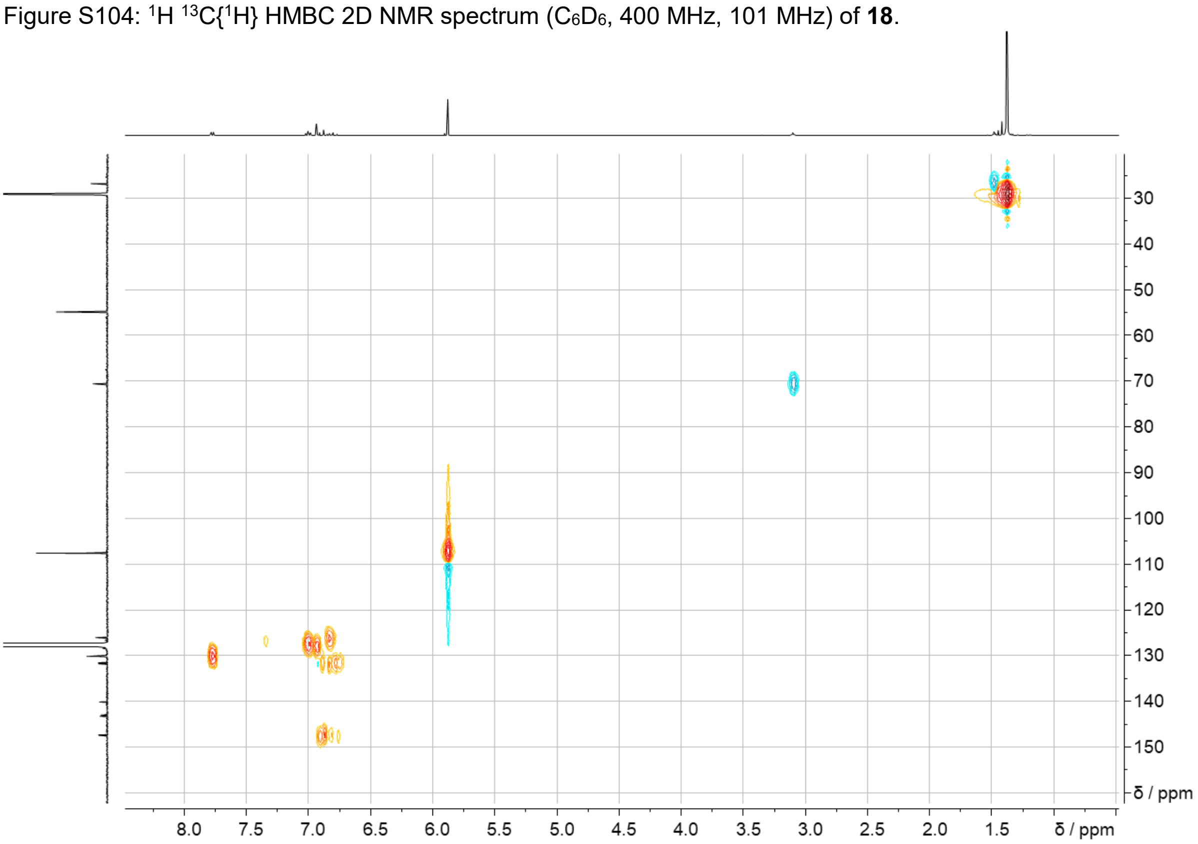

Figure S105: ^{1}H ^{13}C{^{1}H} HSQC 2D NMR spectrum (C_6D_6, 400 MHz, 101 MHz) of **18**.

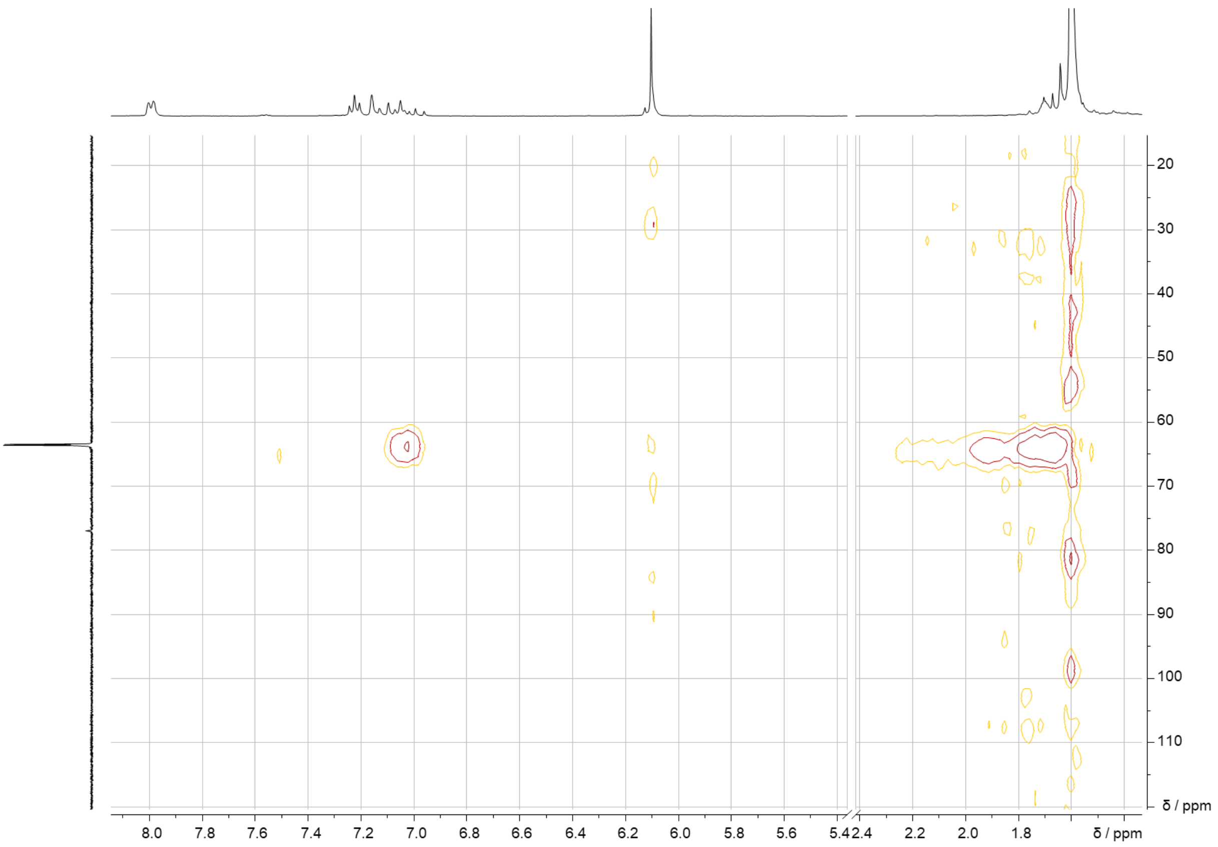

Figure S106: ^{1}H ^{31}P HMBC 2D NMR spectrum (C_6D_6, 400 MHz, 162 MHz) of **18**.

Table S2: Comparison of the coupling constants attributed to the alkene unit in **18** to literature known compounds with the structure **X**$_2$PCH=CH**Y**.

entry	X	Y	isomer	$^{1}J_{PC}$	$^{2}J_{PC}$	$^{2}J_{PH}$	$^{3}J_{PH}$	$^{3}J_{HH}$
1 (**18**)	**R^1**	**Ph**	Z	**13.0**	**21.3**	**1.5**	**22.7**	**13.1**
2 [9], [10]	Ph	Ph	Z			2.7		12.7
3 [9], [11]	Ph	CH_3	Z			1.6	28.3	11.6
4 [12]	EtO	*t*Bu	Z	29.2	19.8	-0.2	23.7	
5 [12]	$(CH_3)_2N$	*t*Bu	Z	11.4	21	+0.2	24.9	
6 [12]	EtO	Ph	E	24.3	33	13.4	11.8	
7 [12]	$(CH_3)_2N$	Ph	E	1.1	20.2	21.1	6.9	

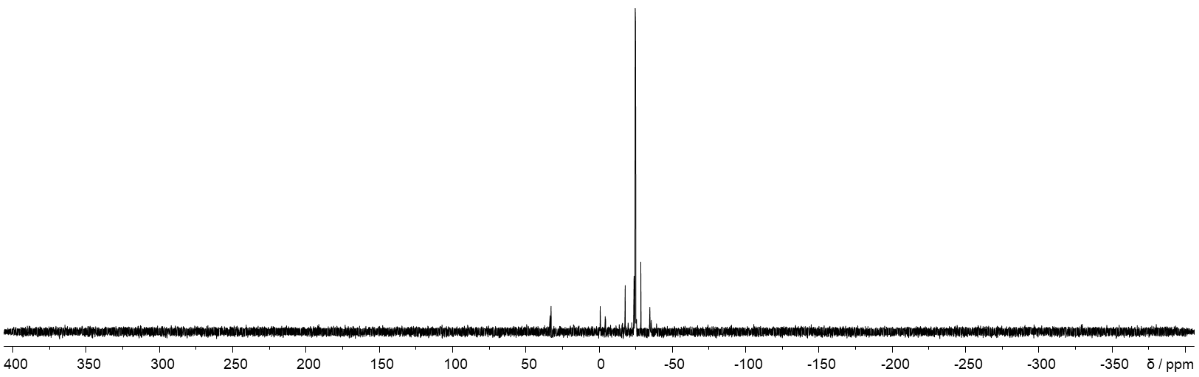

Figure S107: ^{31}P NMR spectrum (C_6D_6, 162 MHz) of the reaction mixture of **1** and phenylacetylene after 2 weeks of stirring showing decomposition products.

1.13.1.2 Reaction of **3** with phenylacetylene yielding alkenylphosphine **19**

Quantitative ^{31}P NMR spectra are shown in Figure S108. Nearly complete conversion was achieved after 5 days, with the main product resonances at around 60 ppm accounting for ~76% of the phosphorus species. Figure S109 offers a more detailed view of the main ^{31}P NMR resonances. One of the products is observed as a doublet at around 60 ppm, with a coupling constant of J_{PH} = 19-20 Hz. This signal can be tentatively assigned to the (Z)-isomer alkenylphosphine **19**, the hydrophosphination product formed from **3** and phenylacetylene. This assignment is consistent with the data for compound **18**. The other resonance at approximately 60 ppm is less well-resolved. The multiplet is tentatively interpreted as a doublet of doublets, with coupling constants of J_{PH} = ~35 Hz and J_{PH} = 1-10 Hz, which likely corresponds to the (E)-isomer.[12] These two stereoisomers are also observed in the molecular structure determined by SCXRD analysis. The (Z)-isomer is the predominant species with an occupation of 90%, while the (E)-isomer accounts for the remaining 10%.

Notably, after removing the volatiles *in vacuo*, an attempt was made to isolate the main product via crystallization from a saturated Et_2O solution at -40 °C. ^{31}P NMR analysis revealed that the number of phosphorus species had been reduced but isolation of a single isomer was not successful (see Figure S110).

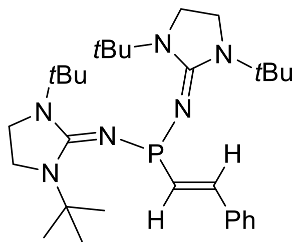

(E)-**19**

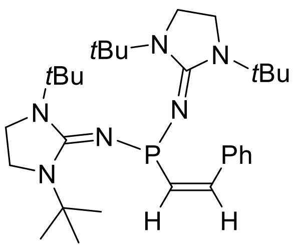

(Z)-**19**

(Z)-**19**:
^{31}P NMR (162 MHz, C_6D_6): δ (ppm) = 60 (d, $^3J_{PH}$ = 19-20 Hz).
(E)-**19**:
^{31}P NMR (162 MHz, C_6D_6): δ (ppm) = 60 (m, *presumably*: dd, J_{PH} = ~35 Hz, J_{PH} = 1-10 Hz).

HRMS (ESI, positive): m/z calculated for $[C_{30}H_{52}N_6P]^+$ (**19**+H)$^+$ 527.3986, found: 527.3970.
Crystals suitable for **SCXRD** were obtained from diffusing hexane into a saturated toluene solution at -40 °C.

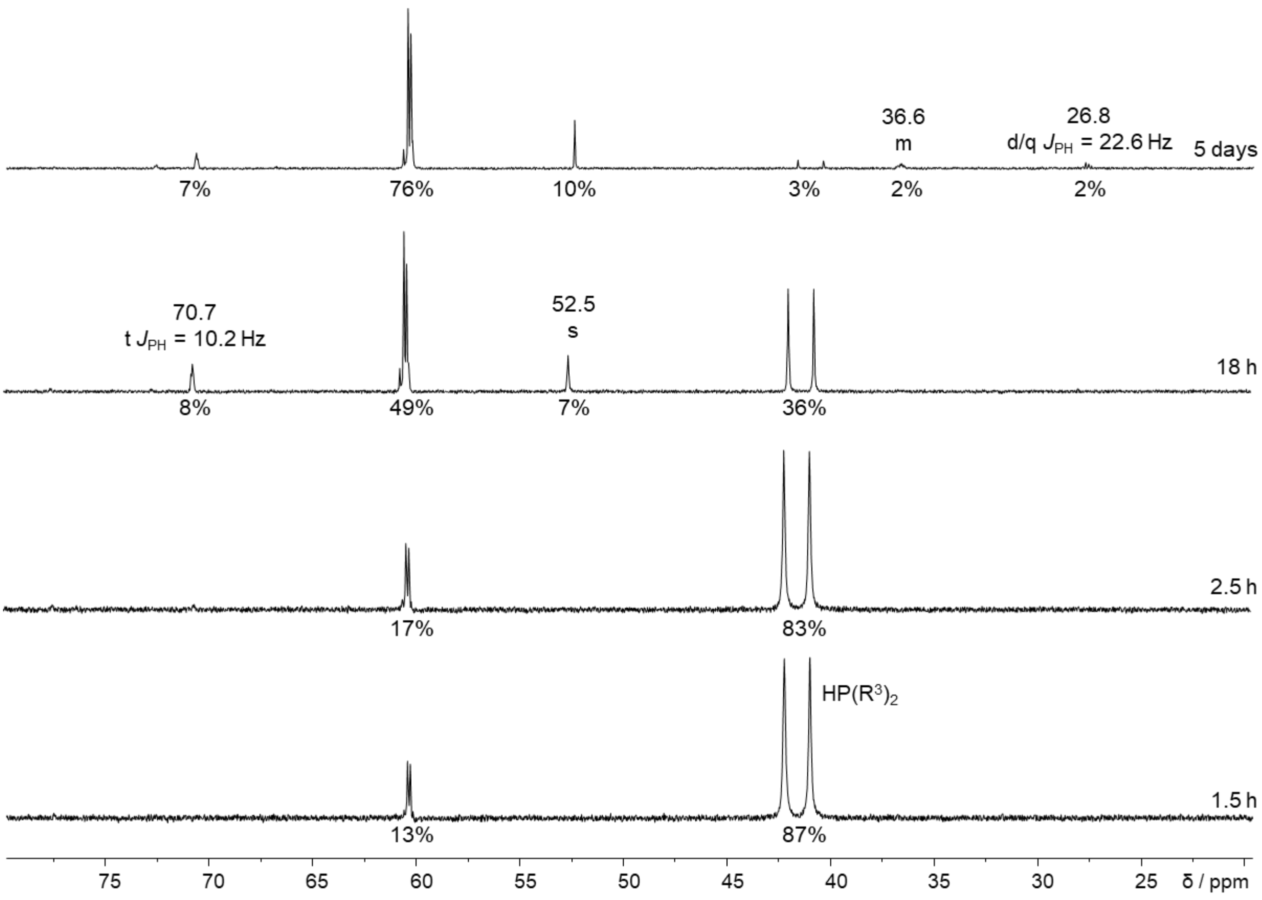

Figure S108: Quantitative ^{31}P NMR spectra (toluene, 162 MHz, relaxation delay D_1 = 25 sec) of the reaction mixture of **3** and phenylacetylene, selected chemical shift range: no other resonances between 400 to -400 ppm.

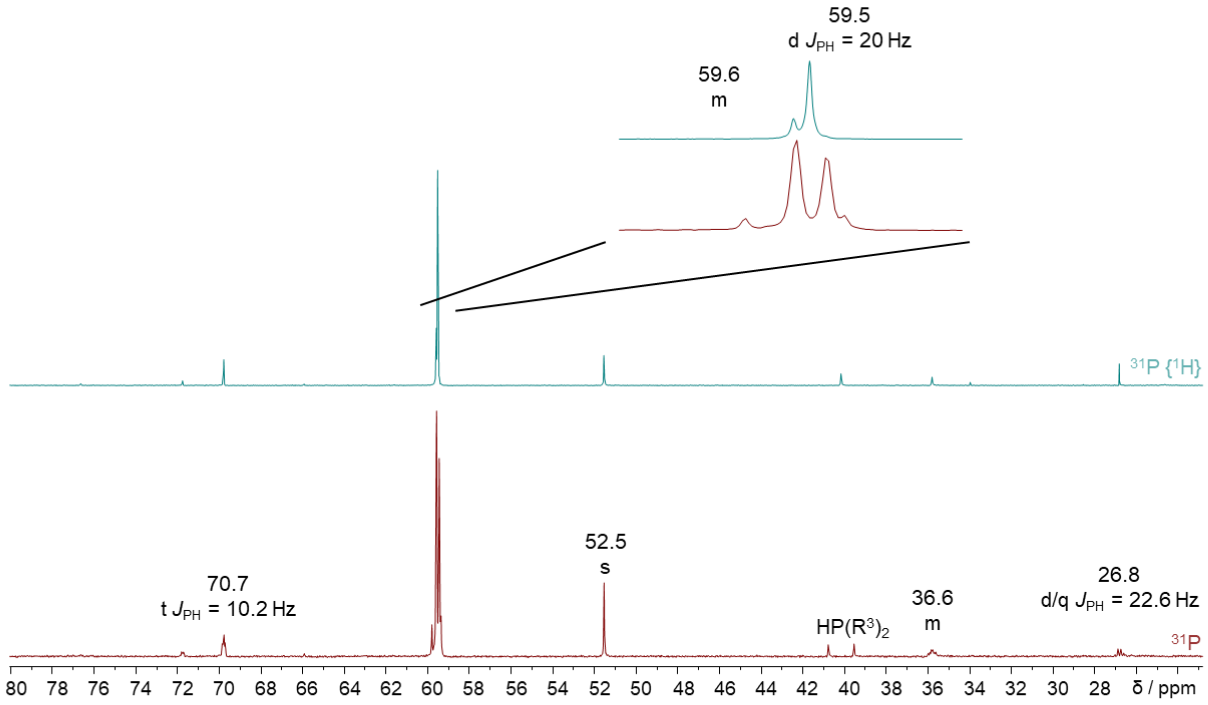

Figure S109: ^{31}P NMR spectrum (bottom) and $^{31}P\{^1H\}$ NMR spectrum (top) (toluene, 162 MHz) of the reaction mixture of **3** and phenylacetylene after 5 days.

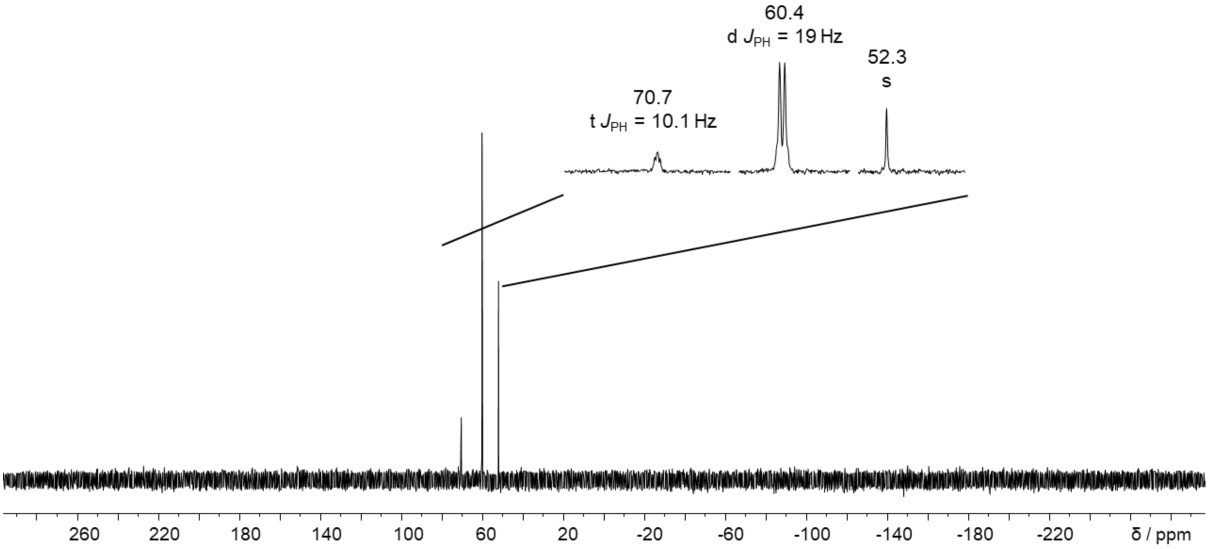

Figure S110: ^{31}P NMR spectrum (C_6D_6, 162 MHz) of an isolation attempt of the reaction mixture of **3** and phenylacetylene.

1.13.1.3 Deuteration experiments - Reactions with (ethynyl-*d*)benzene (phenylacetylene-*d*)

A Teflon-sealed NMR tube was charged with either **1** or **3** (**1**: 46.3 mg, **3**: 46.7 mg; 0.110 mmol, 1.00 eq.) and toluene (0.7 mL), stoichiometric amounts of (ethynyl-*d*)benzene (DCCPh, ~1.1 M in toluene, 0.10 mL, 0.11 mmol, 1.0 eq.) were added. The progress of the reaction was monitored by ^{31}P NMR spectroscopy. Once complete conversion of the secondary phosphine was observed (**1**: 48 hours, **3**: 100 hours), all volatiles were removed *in vacuo*. The residue was dissolved in C_6D_6 and the solution was analyzed by NMR spectroscopy. Notably, phosphorus-deuterium (P–D) couplings are not visible in the spectra, as the heteronuclear coupling constants involving deuterium are significantly smaller than those involving hydrogen (J_{XH}/J_{XD} = 6.5)[13] for nuclei in identical environments.

For both phosphines, the ^{31}P and ^{31}P{^{1}H} NMR spectra (Figure S111 and Figure S112) display several resonances at approximately 60 ppm, which fall within the characteristic region of the alkenylphosphines **18** and **19** described above.

In the case of **1,** the ^{31}P NMR spectra show resonances corresponding to four isomers. Two of these resonances appear as doublets, with coupling constants of J_{PH} = ~25 Hz. Assuming that only the (Z)-isomer is formed, these signals can be attributed to isomers resulting from the incorporation of deuterium into all olefinic positions, specifically: PHC=CHPh (doublet), PHC=CDPh (singlet), PDC=CHPh (doublet), PDC=CDPh (singlet).

For **3**, at least six isomers are observed, with three of the resonances appearing as doublets, having coupling constants of J_{PH} = 17-23 Hz. This can be explained by the formation of both (E)- and (Z)-isomers, each carrying deuterium at different olefinic positions.

Notably, the ^{1}H NMR spectra did not provide conclusive information.

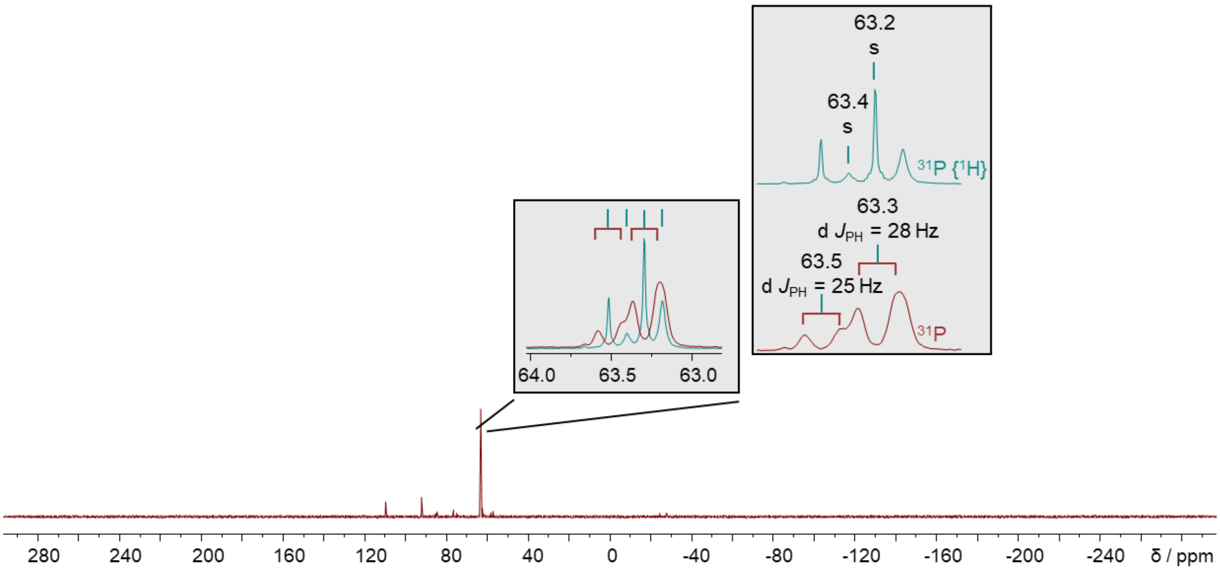

Figure S111: ^{31}P NMR spectra (C_6D_6, 162 MHz) of the reaction mixture of **1** and phenylacetylene-*d*.

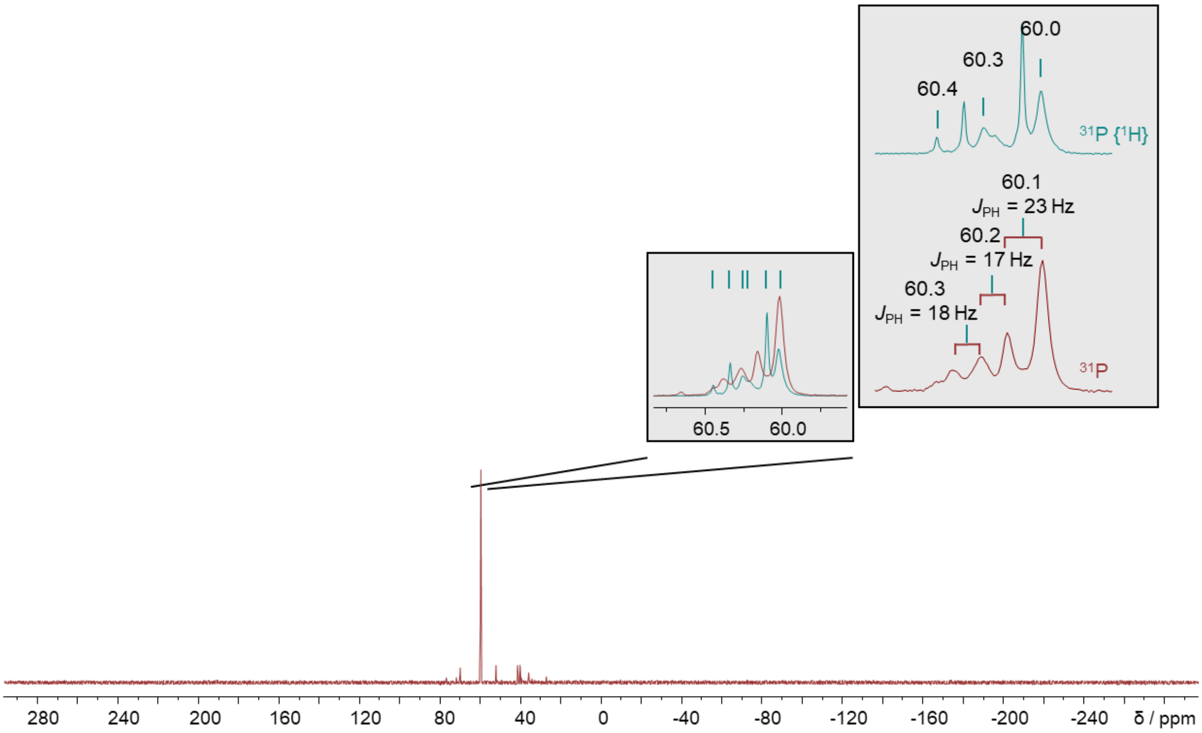

Figure S112: ^{31}P NMR spectra (C_6D_6, 162 MHz) of the reaction mixture of **3** and phenylacetylene-*d*.

1.13.2 Reaction with Diphenylacetylene

The respective phosphine **1** or **3** (**1**: 42.1 mg, **3**: 42.5 mg, 0.100 mmol, 1.00 eq.) and diphenylacetylene (17.8 mg, 0.100 mmol, 1.00 eq.) were dissolved in benzene (0.7 mL) and heated to 70 °C for 2 hours (**1**) or 72 hours (**3**). The conversion was determined by quantitative ^{31}P NMR measurements (**22**: 55%, **23**: 97%). The solvent was then removed *in vacuo* and the residue was dissolved in small amounts of diethyl ether (1 mL) and stored at -40 °C. Product **22** was obtained as yellow, **23** as white crystalline solid.

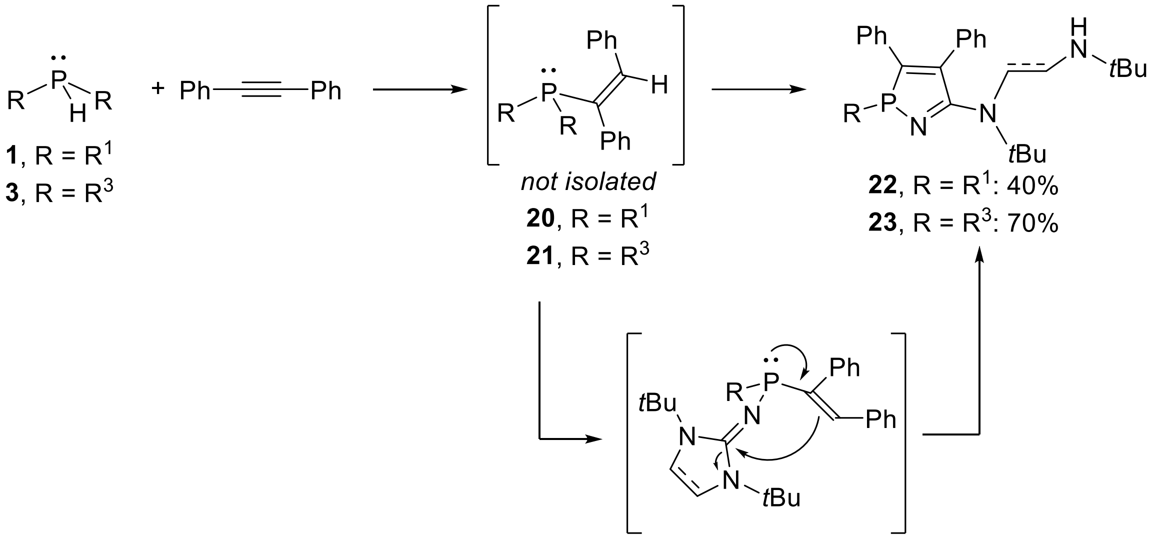

Figure S113: Reaction of secondary IAPs **1** and **3** with diphenylacetylene, forming alkenylphosphines **20** and **21** which react to azaphospholenes **22** and **23**. An intramolecular ring closure is suggested as mechanism.

We postulate that the heterocycles are formed in an intramolecular ring closure reaction followed by proton transfer (Figure S113). The electron-rich phosphorus atom facilitates intramolecular nucleophilic attack of the alkenyl β-carbon atom at the NHI substituent. This results in the formation of the azaphosphole heterocycle after proton transfer.

1.13.2.1 Characterization Data of **22**

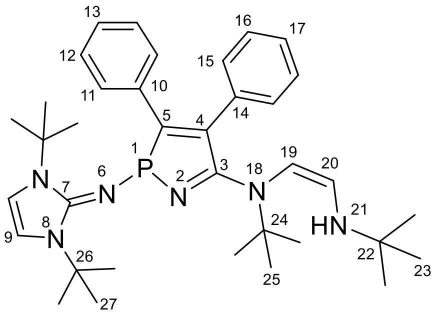

Yield 40% (24 mg, 0.040 mmol).

^{1}H NMR (400 MHz, C_6D_6): δ (ppm) = 7.47 (d, $^3J_{HH}$ = 7 Hz, 2H, H11), 7.39 (d, $^3J_{HH}$ = 7 Hz, 2H, H15), 7.10 (t, $^3J_{HH}$ = 7 Hz, 2H, H16), 7.04 (t, $^3J_{HH}$ = 7 Hz, 2H, H12), 6.98 (t, $^3J_{HH}$ = 7 Hz, 1H, H17), 6.93 (t, $^3J_{HH}$ = 7 Hz, 1H, H13), 6.06 (s, 2H, H9), 5.65 (pseudo-t (dd), $^3J_{HH}$ = 12.0 Hz, $^3J_{HH}$ = 12.0 Hz 1H, H20), 4.62 (dd, $^3J_{HH}$ = 12.0 Hz, $^5J_{PH}$ = 1.3 Hz 1H, H19), 2.24 (d, $^3J_{HH}$ = 12.0 Hz, 1H, H21), 1.73 (s, 9H, H25), 1.47 (s, 18H, H27), 0.77 (s, 9H, H23).

^{13}C{^{1}H} NMR (101 MHz, C_6D_6): δ (ppm) = 166.8 (d, $^1J_{PC}$ = 21.4 Hz, C5), 161.2 (C3), 147.7 (d, $^2J_{PC}$ = 28.7 Hz, C7), 141.6 (d, $^3J_{PC}$ = 30.2 Hz, C4), 140.0 (d, $^2J_{PC}$ = 11.6 Hz, C10), 138.2 (d, $^3J_{PC}$ = 4.7 Hz, C14), 132.6 (C20), 130.2 (d, $^4J_{PC}$ = 2.9 Hz, C15), 130.1 (d, $^3J_{PC}$ = 6.9 Hz, C11), 127.7 (C-12), 127.4 (C16), 125.9 (C17), 125.7 (C13), 109.4 (C19), 108.9 (C9), 56.9 (C24), 55.9 (C26), 50.0 (C22), 29.7 (C23), 29.7 (d, $^5J_{PC}$ = 3.3 Hz, C27), 27.8 (C25).

^{31}P NMR (162 MHz, C_6D_6): δ (ppm) = 93.4.

HRMS (ESI, positive): m/z calculated for $[C_{36}H_{52}N_6P]^+$ (**22**+H)$^+$ 599.39856, found: 599.39728.

Crystals suitable for **SCXRD** were obtained from diffusing diethyl ether into a saturated DCM solution at -40 °C. These crystals reveal the decomposition product of **22** with DCM ([**22**]-CH_2Cl [Cl], see Figure S114) which shows the intact, planar phospholium heterocycle with its substituents, as it is also present in **22**.

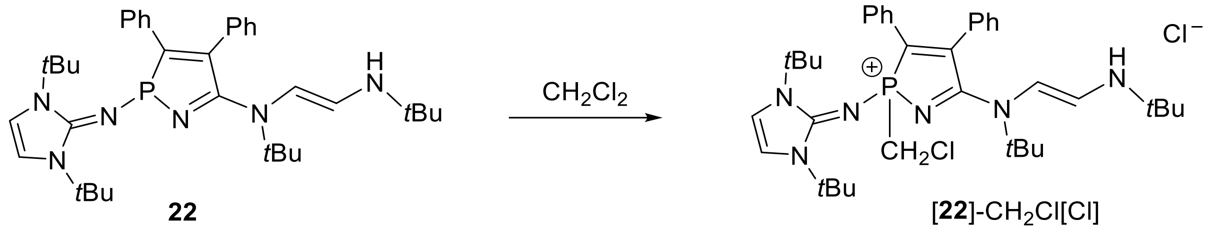

Figure S114: Reaction of **22** with DCM. [**22**]-CH_2Cl[Cl] was characterized by SCXRD.

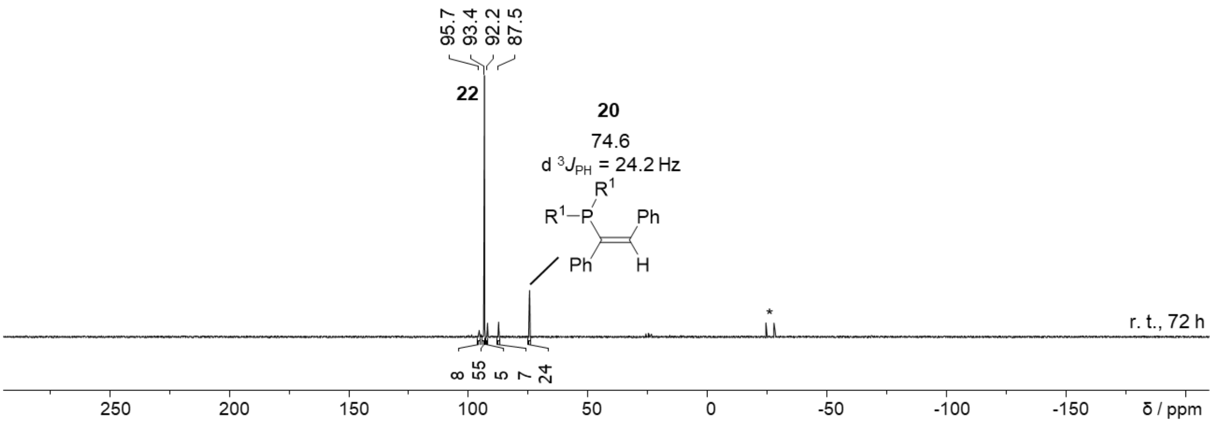

Figure S115: Quantitative ^{31}P NMR spectrum (C_6D_6, 162 MHz, relaxation delay D_1 = 25 sec) of the reaction mixture of **1** and diphenylacetylene, asterisk (*) marks phosphine oxide $H(R^1)_2P{=}O$ (**10**).

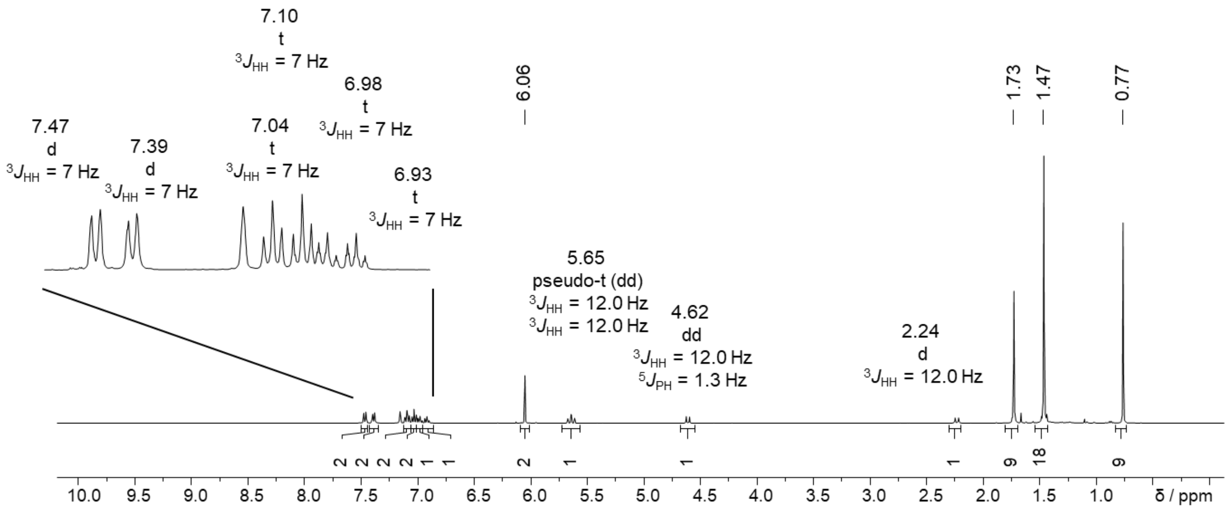

Figure S116: ^{1}H NMR spectrum (C_6D_6, 400 MHz) of **22**.

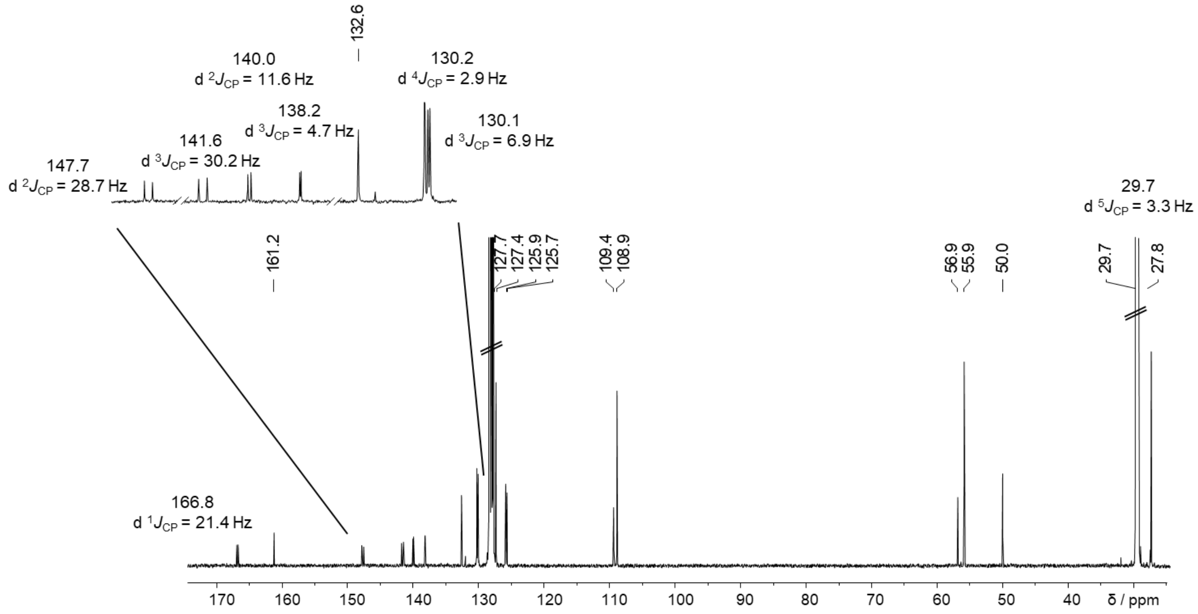

Figure S117: $^{13}C\{^{1}H\}$ NMR spectrum (C_6D_6, 101 MHz) of **22**.

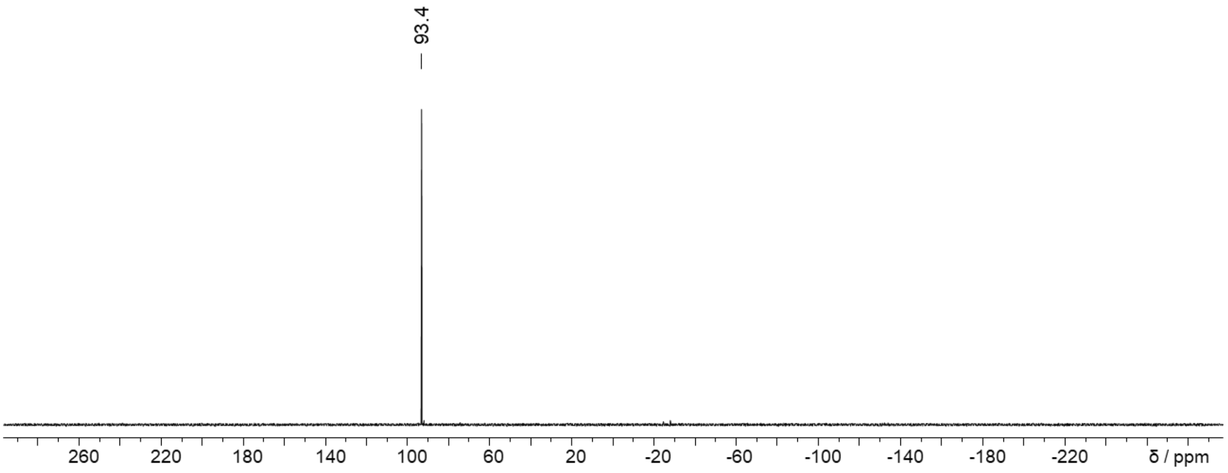

Figure S118: ^{31}P NMR spectrum (C_6D_6, 162 MHz) of **22**.

1.13.2.2 Characterization Data of **23**

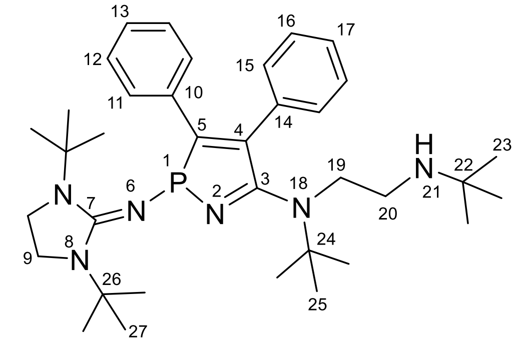

Yield 70% (42 mg, 0.70 mmol).

^{1}H NMR (400 MHz, C_6D_6): δ (ppm) = 7.44–7.39 (m, 4H, H11, H15), 7.09–7.01 (m, 4H, H12, H16), 6.99– 6.90 (m, 2H, H13, H17), 3.00 (td, $^{3}J_{HH}$ = 6.5 Hz, $^{4}J_{PH}$ = 1.5 Hz, 2H, H19), 2.68 (s, H4, H9), 2.46 (pseudo-q (dt), $^{3}J_{HH}$ = 6.5 Hz, $^{3}J_{HH}$ = 7.9 Hz 2H, H20), 1.62 (s, 9H, H25), 1.32 (s, H18, H27), 1.01 (s, H9, H23), 0.61 (t, $^{3}J_{PH}$ = 7.9 Hz, 1H, H21).

^{13}C{^{1}H} NMR (101 MHz, C_6D_6): δ (ppm) = 168.8 (d, $^{1}J_{PC}$ = 21.8 Hz, C5), 164.3 (C3), 154.8 (d, $^{2}J_{PC}$ = 19.1 Hz ,C7), 140.6 (d, $^{3}J_{PC}$ = 29.6 Hz, C4), 138.9 (d, $^{2}J_{PC}$ = 11.2 Hz, C10), 137.4 (d, $^{3}J_{PC}$ = 4.8 Hz, C14), 130.5 (d, $^{4}J_{PC}$ = 2.9 Hz, C15), 129.9 (d, $^{3}J_{PC}$ = 8.8 Hz, C11), 128.1 (C16), 127.9 (C12), 126.8 (C17), 126.1 (C13), 56.7 (C24), 53.7 (C26), 49.9 (C22), 48.7 (d, $^{4}J_{PC}$ = 3.2 Hz, C19), 43.8 (C20), 42.4 (C9), 29.4 (C25), 29.3 (C23), 29.0 (d, $^{5}J_{PC}$ = 3.9 Hz, C27).

^{31}P NMR (162 MHz, C_6D_6): δ (ppm) = 80.1.

HRMS (ESI, positive): m/z calculated for $[C_{36}H_{56}N_6P]^+$ (**23**+H)$^+$ 603.42986, found: 603.42933.

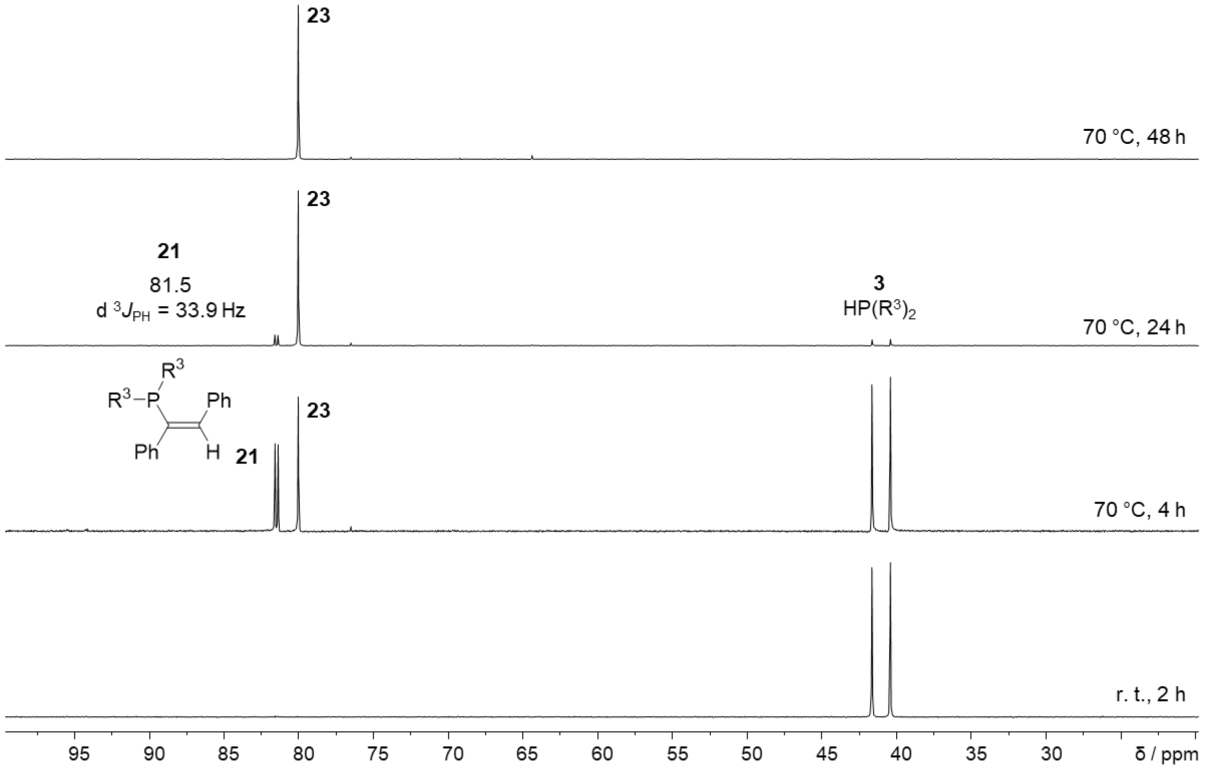

Figure S119: ^{31}P NMR spectra (C_6D_6, 162 MHz) of the reaction mixture of **3** and diphenylacetylene.

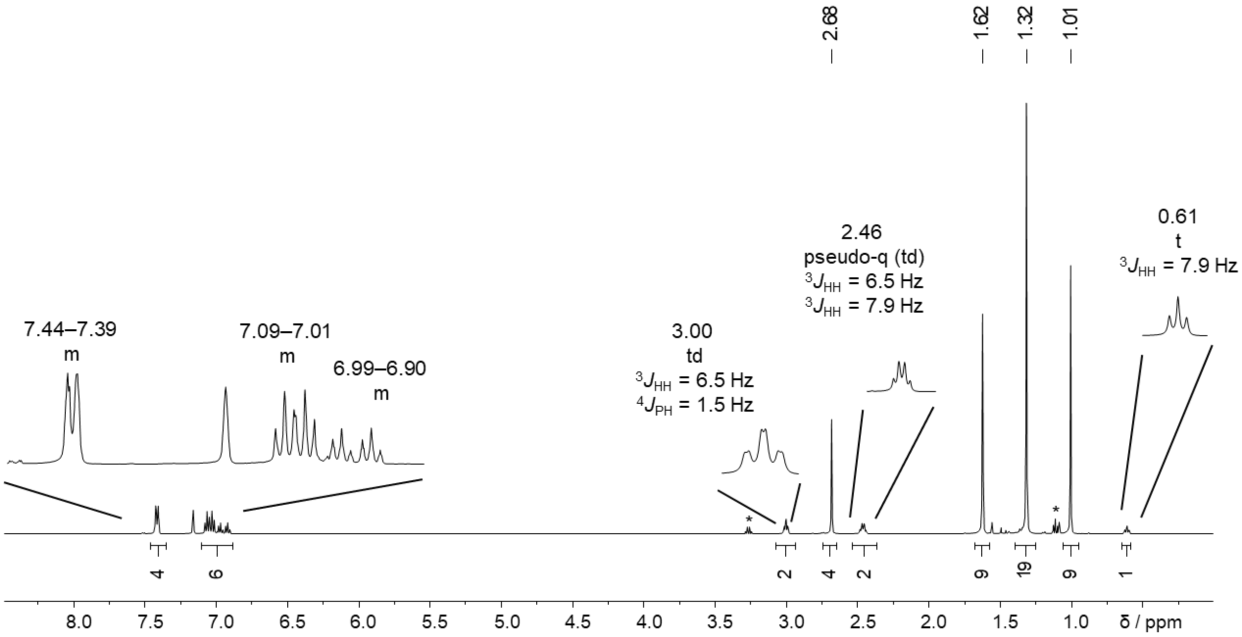

Figure S120: ^{1}H NMR spectrum (C_6D_6, 400 MHz) of **23**, asterisks (*) mark residual Et_2O.

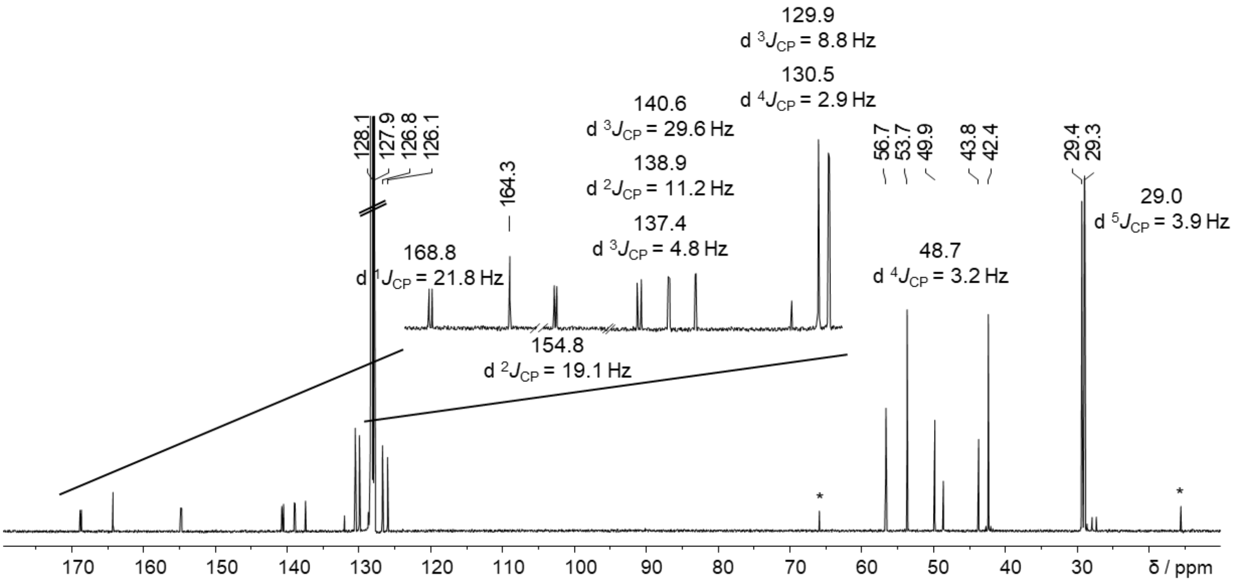

Figure S121: $^{13}C\{^1H\}$ NMR spectrum (C_6D_6, 101 MHz) of **23**.

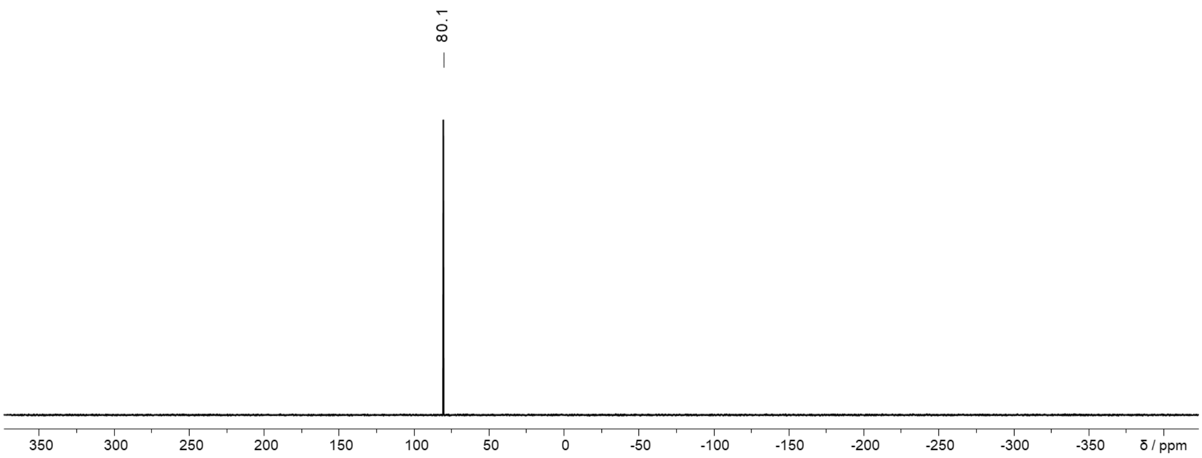

Figure S122: ^{31}P NMR spectrum (C_6D_6, 162 MHz) of **23**.

2 X-ray Crystallography

General:

Single-crystal X-ray diffraction data were collected on a Bruker AXS, on a Bruker D8 QUEST PHOTON III or on a Bruker D8 VENTURE Photon 100 diffractometer.

The structures were solved with the ShelXT[14] solution program using dual methods and by using Olex2 1.5-alpha[15] as the graphical interface. The model was refined with ShelXL 2018/3[16] using full matrix least squares minimization on F^2. All hydrogen atom positions were calculated geometrically and refined using the riding model, if not stated otherwise.

Crystallographic data have been deposited with the Cambridge Crystallographic Data Centre as supplementary publication no. CCDC-2489012 (**1**), CCDC-2489013 (**2**), CCDC-2489021 (**3**), CCDC-2489014 (**4**), CCDC-2489015 (**8**), CCDC-2489016 ([**14**][SbF_6]), CCDC-2489022 (**15**), CCDC-2489017 (**16**), CCDC-2489018 (**18**), CCDC-2489019 (**19**) and CCDC-2489020 ([**22**]-CH_2Cl[Cl]). These data can be obtained free of charge via www.ccdc.cam.uk/data_request/cif (or from the CCDC, 12 Union Road, Cambridge CB2 1EZ, UK; fax: (+44) 1223-336-033; or deposit@ccdc.cam.ac.uk).

2.1 Crystal Structure Data of Compound 1

Colorless crystals were obtained from a saturated toluene solution of **1** after storage at -40 °C. X-ray structure analysis shows that **1** crystallizes in the orthorhombic space group $P2_12_12$. There are two half molecules of **1** in the asymmetric unit. The PH group is located close to a 2-fold axes, showing a disorder over two positions (occupancy 50:50). The hydrogen atoms on the phosphorus atoms were located from the difference Fourier map. The P–H distances were refined freely and the atomic displacement parameters of the hydrogen atoms were linked to the ADPs of the phosphorus atoms (-1.2).

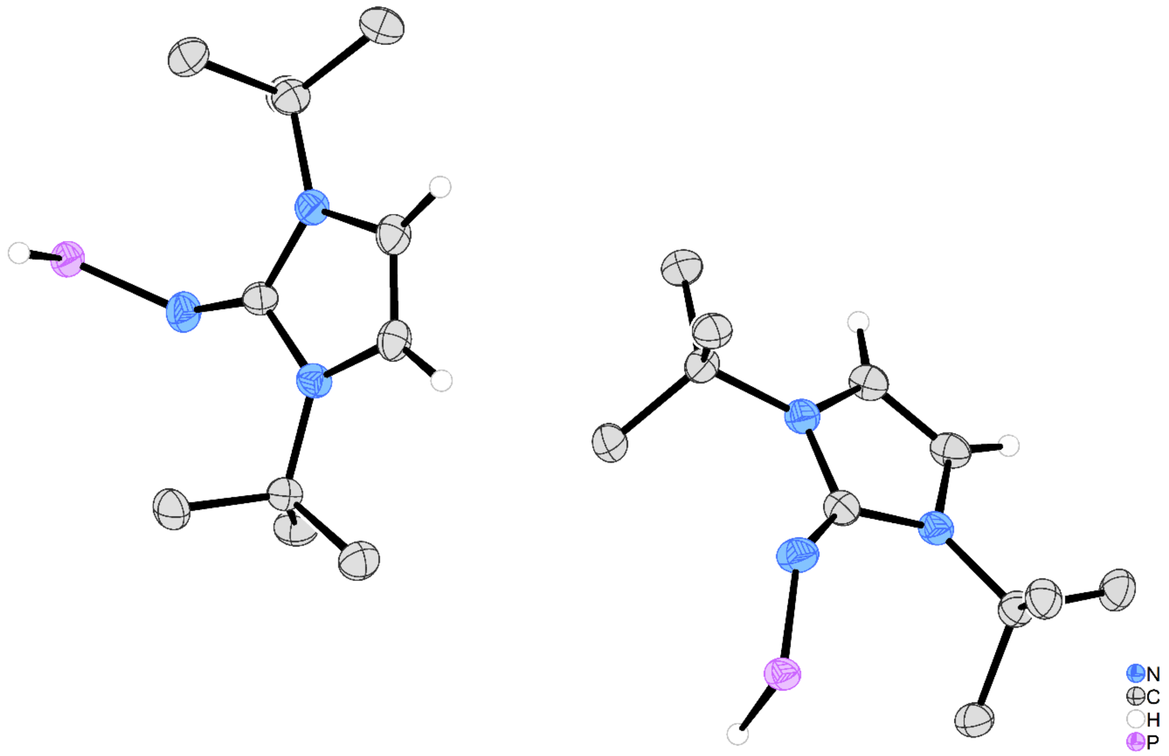

Figure S123: The asymmetric unit of the crystal structure of **1** with thermal ellipsoid plot at the 50% levels of probability. Hydrogen atoms except the phosphorus centered hydrogen atoms and the hydrogen atoms in the NHI backbone are omitted for clarity.

Table S3: Crystal data and structure refinement for **1**.

CCDC number	2489012	ρ_{calc}/ g cm^{-3}	1.166
Empirical formula	$C_{22}H_{41}N_6P$	μ/mm^{-1}	0.135
Formula weight	420.58	F(000)	920.0
Temperature/K	100	Crystal size/mm^3	0.661 × 0.253 × 0.18
Crystal system	orthorhombic	Radiation	MoK$_\alpha$ (λ = 0.71073)
Space group	$P2_12_12$	2Θ range for data collection/°	4.19 to 56.456
a/Å	15.4452(6)	Index ranges	-20 ≤ h ≤ 20, -21 ≤ k ≤ 21, -12 ≤ l ≤ 12
b/Å	15.9546(6)	Reflections collected	36779
c/Å	9.7209(4)	Independent reflections	5848 [R_{int} = 0.0603, R_{sigma} = 0.0379]
α/°	90	Data/restraints/parameters	5848/0/289
β/°	90	Goodness-of-fit on F^2	1.086
γ/°	90	Final R indexes [I>=2σ (I)]	R_1 = 0.0569, wR_2 = 0.1701
Volume/Å^3	2395.44(16)	Final R indexes [all data]	R_1 = 0.0623, wR_2 = 0.1747
Z	4	Largest diff. peak/hole / e Å^{-3}	0.47/-0.26
		Flack parameter	-0.13(5)

2.2 Crystal Structure Data of Compound 2

Clear colorless crystals were obtained from a saturated *n*-hexane solution of **2** after storage at -40 °C. X-ray structure analysis shows that **2** crystallizes in the triclinic space group $P\bar{1}$. The asymmetric unit contains three molecules of **2**, in each of which the phosphorus atom is disordered over two positions (occupation 55:45, 51:49, 51:49). Additionally, some of the *i*Pr groups of the Dipp substituents show a disorder over two positions. The hydrogen atoms on the phosphorus atoms were located from the difference Fourier map and refined freely.

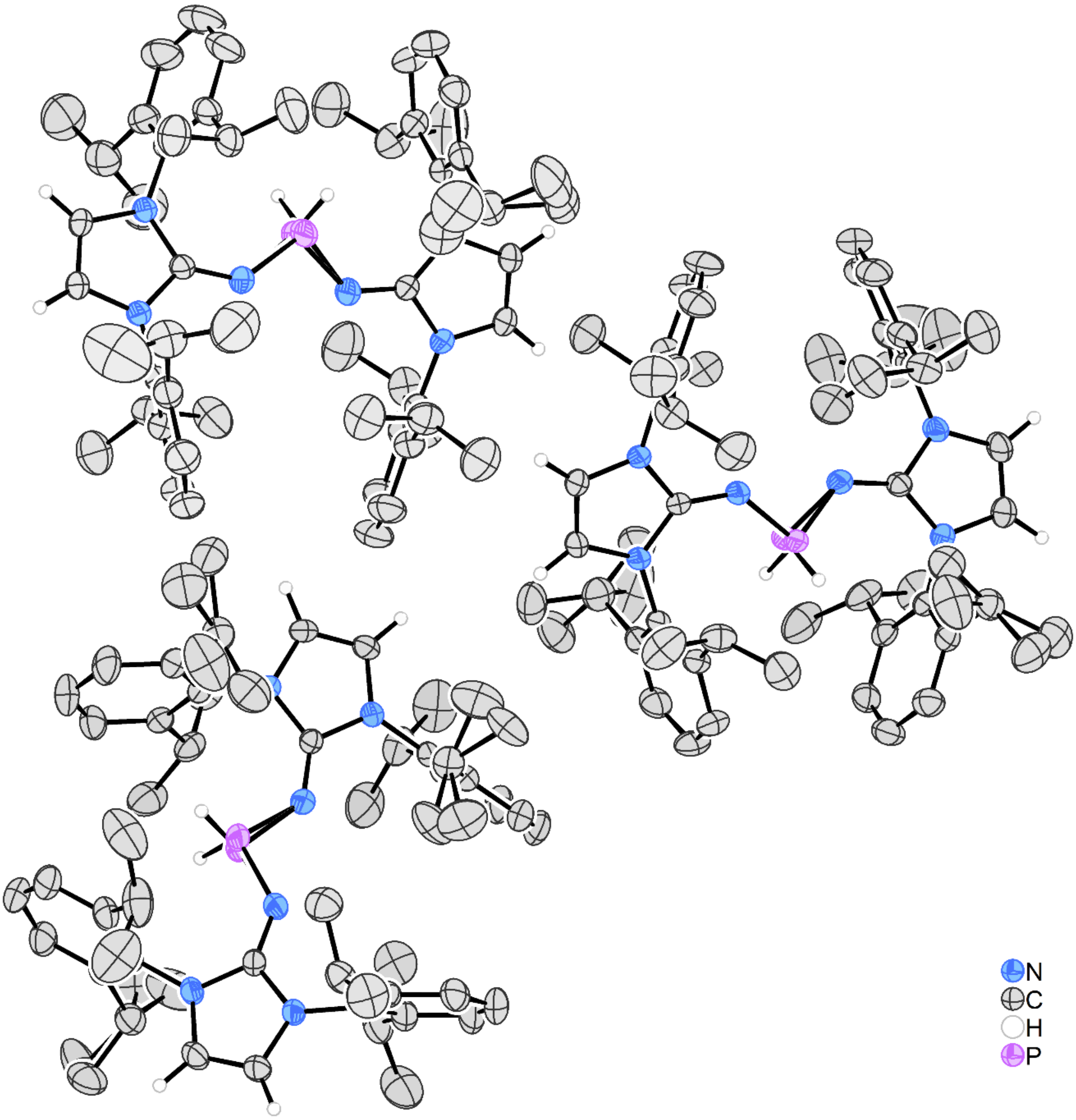

Figure S124: The asymmetric unit of the crystal structure of **2** with thermal ellipsoid plot at the 50% levels of probability. Hydrogen atoms except the phosphorus centered hydrogen atoms and the hydrogen atoms in the NHI backbone are omitted for clarity.

Table S4: Crystal data and structure refinement for **2**.

CCDC number	2489013	ρ_{calc} / g cm^{-3}	1.888
Empirical formula	$C_{54}H_{73}N_6P$	μ/mm^{-1}	0.093
Formula weight	837.15	F(000)	2724
Temperature/K	183.00	Crystal size/mm^3	0.24 x 0.19 x 0.17
Crystal system	triclinic	Radiation	MoK$_\alpha$ (λ = 0.71073)
Space group	$P\bar{1}$.	2Θ range for data collection/°	3.776 to 53.000
a/Å	10.7303(5)	Index ranges	-13 ≤ h ≤ 13, -28 ≤ k ≤ 28, -40 ≤ l ≤ 40
b/Å	22.6065(11)	Reflections collected	178555
c/Å	31.9804(15)	Independent reflections	31914 [R_{int} = 0.0435, R_{sigma} = 0.0335]
α/°	92.3605(17)	Data/restraints/parameters	31914/0/1890
β/°	95.9517(17)	Goodness-of-fit on F^2	1.044
γ/°	92.6529(18)	Final R indexes [I>=2σ (I)]	R_1 = 0.0449, wR_2 = 0.1113
Volume/Å^3	7699.3(6)	Final R indexes [all data]	R_1 = 0.0624, wR_2 = 0.1213
Z	6	Largest diff. peak/hole / e Å^{-3}	0.344/-0.351

2.4 Crystal Structure Data of Compound 3

Colorless crystals were obtained from a saturated *n*-hexane solution of **3** after storage at -40 °C. X-ray structure analysis shows that **3** crystallizes in the triclinic space group $P\bar{1}$. The asymmetric unit contains two molecules of **3**, in each of which the phosphorus atom is disordered over two positions (occupation 48:52, 43:57). The hydrogen atoms on the phosphorus atoms were located from the difference Fourier map. The P–H distances at the P1A and P1B atoms were restrained to 1.48 Å (DFIX) and for the P–H distances at the P2A and P2B atoms a SADI restraint was applied. The atomic displacement parameters of the hydrogen atoms were linked to the ADPs of the respective phosphorus atoms (-1.2).

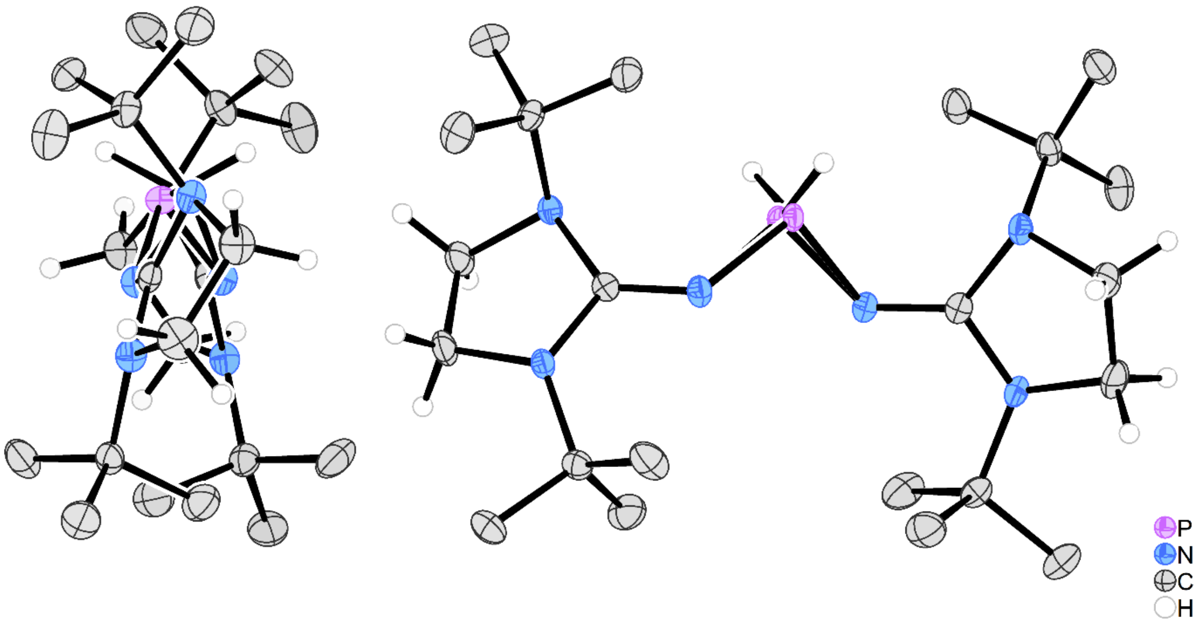

Figure S125: The asymmetric unit of the crystal structure of **3** with thermal ellipsoid plot at the 50% levels of probability. Hydrogen atoms except the phosphorus centered hydrogen atoms and the hydrogen atoms in the NHI backbone are omitted for clarity.

Table S5: Crystal data and structure refinement for **3**.

CCDC number	2489021	ρ_{calc} / g cm^{-3}	1.134
Empirical formula	$C_{22}H_{45}N_6P$	μ/mm^{-1}	0.130
Formula weight	424.61	F(000)	936.0
Temperature/K	100	Crystal size/mm^3	0.256 × 0.234 × 0.147
Crystal system	triclinic	Radiation	MoK$_\alpha$ (λ = 0.71073)
Space group	$P\bar{1}$.	2Θ range for data collection/°	3.106 to 55.92
a/Å	10.1548(4)	Index ranges	-13 ≤ h ≤ 13, -17 ≤ k ≤ 17, -24 ≤ l ≤ 24
b/Å	13.1353(5)	Reflections collected	36035
c/Å	18.8353(7)	Independent reflections	11902 [R_{int} = 0.0526, R_{sigma} = 0.0575]
α/°	93.485(2)	Data/restraints/parameters	11902/2/579
β/°	97.358(2)	Goodness-of-fit on F^2	1.022
γ/°	90.224(2)	Final R indexes [I>=2σ (I)]	R_1 = 0.0491, wR_2 = 0.1128
Volume/Å^3	2486.90(17)	Final R indexes [all data]	R_1 = 0.0716, wR_2 = 0.1218
Z	4	Largest diff. peak/hole / e Å^{-3}	0.33/-0.34

2.5 Crystal Structure Data of Compound 4

Single light yellow block-shaped crystals were obtained from a saturated toluene solution of **4** after storage at -40 °C. X-ray structure analysis shows that **4** crystallizes in the orthorhombic space group *Pca*2$_1$. The asymmetric unit contains one molecule of **4** and a toluene molecule. The $HPNi(CO)_3$ unit of **4** is disordered over two positions (occupation 94:6). Additionally, the toluene molecule shows a disorder over two positions (occupation 63:37). The hydrogen atom on the phosphorus atoms were located from the difference Fourier map. The hydrogen atom on P1 was refined freely and the hydrogen atom at P1B had to be restraint (DFIX, DANG).

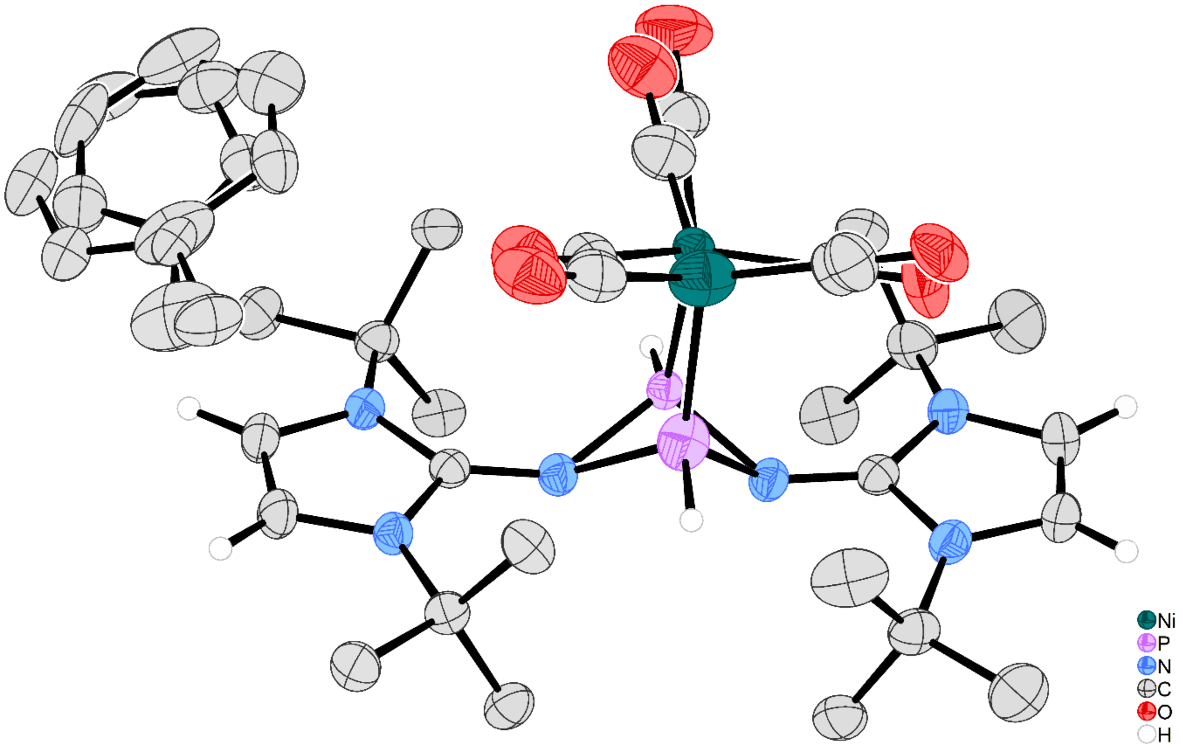

Figure S126: The asymmetric unit of the crystal structure of **4** with thermal ellipsoid plot at the 50% levels of probability. Hydrogen atoms except the phosphorus centered hydrogen atom and the hydrogen atoms in the NHI backbone are omitted for clarity.

Table S6: Crystal data and structure refinement for **4**.

CCDC number	2489014	ρ_{calc}/ g cm^{-3}	1.234
Empirical formula	$C_{32}H_{49}N_6NiO_3P$	μ/mm^{-1}	0.634
Formula weight	655.45	F(000)	1400
Temperature/K	150	Crystal size/mm^3	0.31×0.25×0.17
Crystal system	orthorhombic	Radiation	MoK$_\alpha$ (λ = 0.71073)
Space group	*Pca*2$_1$	2Θ range for data collection/°	4.484 to 56.586
a/Å	18.1640(8)	Index ranges	-24 ≤ h ≤ 24, -14 ≤ k ≤ 14, -23 ≤ l ≤ 22
b/Å	11.0391(5)	Reflections collected	49626
c/Å	17.5892(6)	Independent reflections	8642 [R_{int} = 0.0578, R_{sigma} = 0.0438]
α/°	90	Data/restraints/parameters	8642/289/547
β/°	90	Goodness-of-fit on F^2	1.024
γ/°	90	Final R indexes [I>=2σ (I)]	R_1 = 0.0279, wR_2 = 0.0632
Volume/Å^3	3526.9(3)	Final R indexes [all data]	R_1 = 0.0308, wR_2 = 0.0647
Z	4	Largest diff. peak/hole / e Å^{-3}	0.181/-0.196
		Flack parameter	0.028(10)

2.6 Crystal Structure Data of Compound 8

Crystals were obtained from slow diffusion of *n*-hexane into a saturated toluene solution of **8** at -40 °C. X-ray structure analysis shows that **8** crystallizes in the triclinic space group $P\bar{1}$. The asymmetric unit contains one molecule of **8** and a toluene molecule. The HPSe unit is disordered over two positions (occupation 86:14). The hydrogen atom on the phosphorus atoms were located from the difference Fourier map. The hydrogen atom on P1 was refined freely and the hydrogen atom at P1A had to be restraint (DFIX).

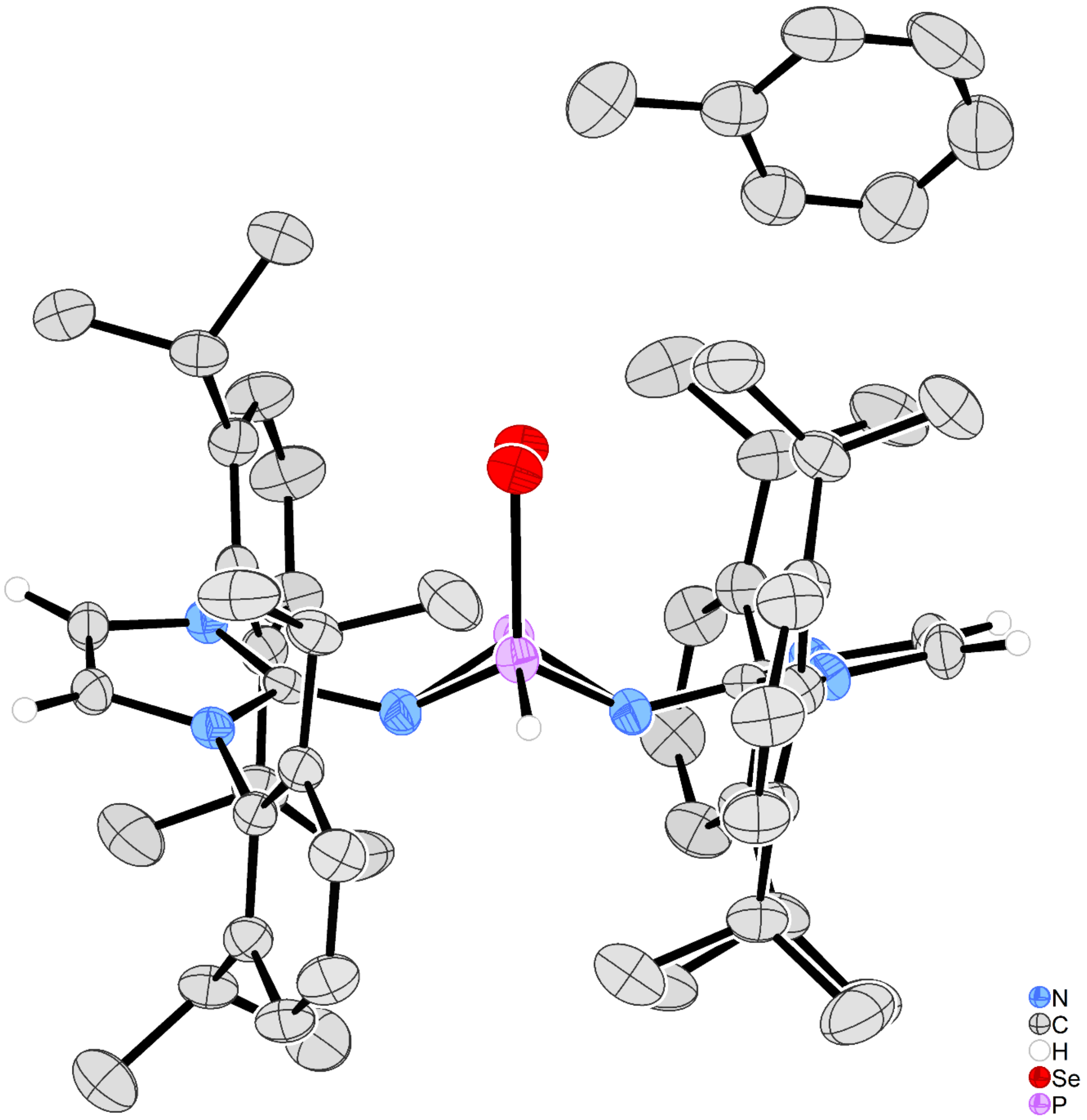

Figure S127: The asymmetric unit of the crystal structure of **8** with thermal ellipsoid plot at the 50% levels of probability. Hydrogen atoms except the phosphorus centered hydrogen atom and the hydrogen atoms in the NHI backbone are omitted for clarity.

Table S7: Crystal data and structure refinement for **8**.

CCDC number	2489015	ρ_{calc} / g cm^{-3}	1.182
Empirical formula	$C_{61}H_{81}N_6PSe$	μ/mm^{-1}	0.733
Formula weight	1008.24	F(000)	1076.0
Temperature/K	173.00	Crystal size/mm^3	0.2 × 0.16 × 0.04
Crystal system	triclinic	Radiation	MoK$_\alpha$ (λ = 0.71073)
Space group	$P\bar{1}$	2Θ range for data collection/°	4.544 to 50.996
a/Å	12.3945(6)	Index ranges	-15 ≤ h ≤ 15, -16 ≤ k ≤ 16, -22 ≤ l ≤ 22
b/Å	13.2287(8)	Reflections collected	69382
c/Å	18.2082(12)	Independent reflections	10506 [R_{int} = 0.0409, R_{sigma} = 0.0292]
α/°	83.997(2)	Data/restraints/parameters	10506/1/664
β/°	80.669(2)	Goodness-of-fit on F^2	1.100
γ/°	74.508(2)	Final R indexes [I>=2σ (I)]	R_1 = 0.0466, wR_2 = 0.1120
Volume/Å^3	2833.0(3)	Final R indexes [all data]	R_1 = 0.0549, wR_2 = 0.1159
Z	2	Largest diff. peak/hole / e Å^{-3}	0.42/-0.62

2.8 Crystal Structure Data of Compound [14][SbF_6]

Colorless crystals were obtained by slow diffusion of diethyl ether into a saturated DCM solution of [**14**][SbF_6] at -40 °C. [**14**][SbF_6] crystallizes in the monoclinic space group *P*2/c. The asymmetric unit contains the one half of the [SbF_6]$^-$ anion, one half of the cation [**14**]$^+$ and half a molecule of DCM. The DCM molecule and the [SbF_6]$^-$ anion are located on a glide plane. The Au-PH unit is located close to a 2-fold rotation axe and therefore disordered over two positions (occupation 50:50). The NHI unit also shows a disorder over two positions (occupation 50:50). The hydrogen atom on the phosphorus atom was located from the difference Fourier map and refined freely.

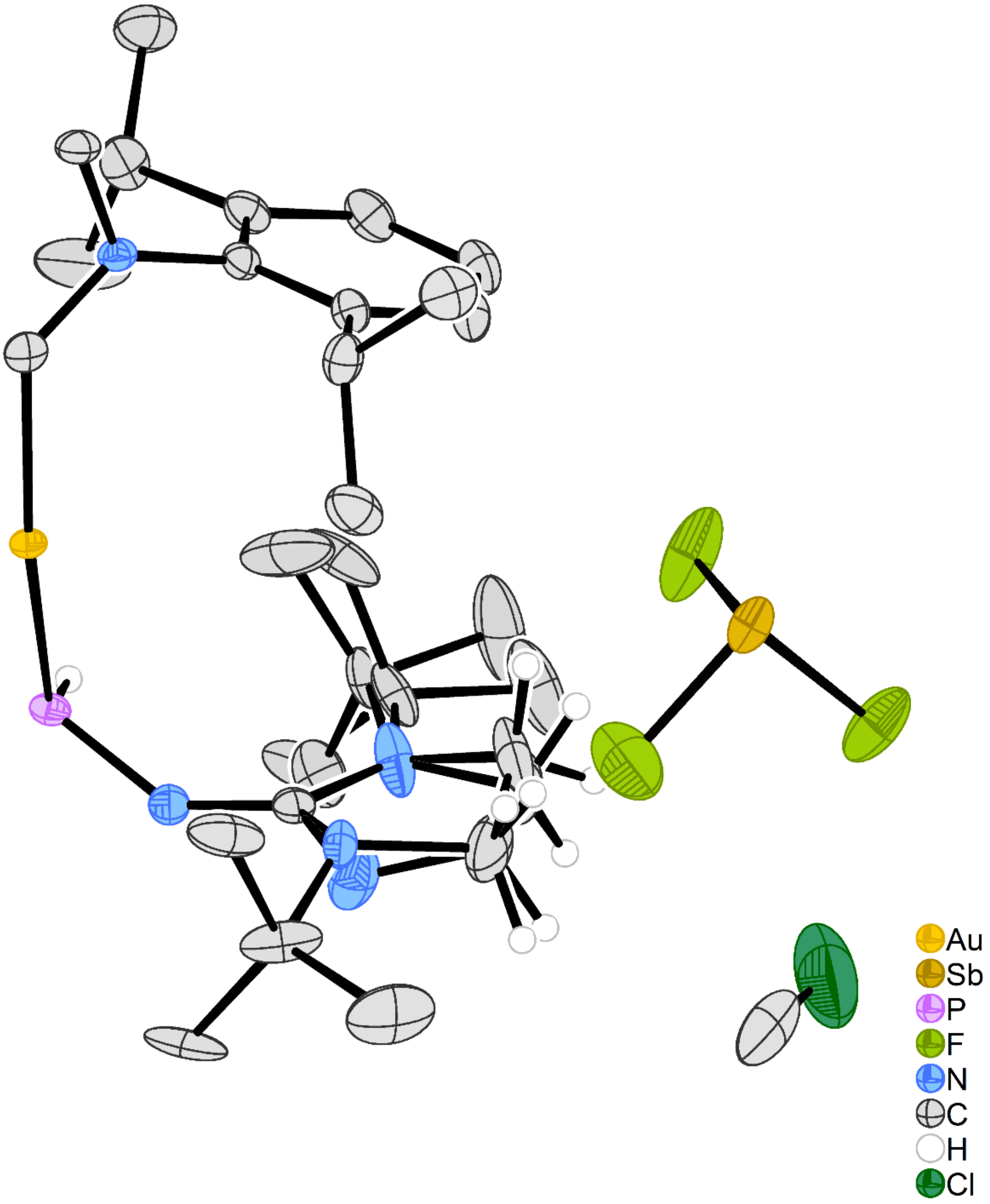

Figure S128: The asymmetric unit of the crystal structure of [**14**][SbF_6] with thermal ellipsoid plot at the 50% levels of probability. Hydrogen atoms except the phosphorus centered hydrogen atom and the hydrogen atoms in the NHI backbone are omitted for clarity.

Table S8: Crystal data and structure refinement for [**14**][SbF_6].

CCDC number	2489016	ρ_{calc} / g cm^{-3}	1.567
Empirical formula	$C_{50}H_{84}AuCl_2F_6N_8PSb$	μ/mm^{-1}	3.259
Formula weight	1331.83	F(000)	1346.0
Temperature/K	100	Crystal size/mm^3	0.739 × 0.427 × 0.369
Crystal system	monoclinic	Radiation	MoK$_\alpha$ (λ = 0.71073)
Space group	*P*2$_{/}$c	2Θ range for data collection/°	3.166 to 59.668
a/Å	10.5524(4)	Index ranges	-14 ≤ h ≤ 14, -14 ≤ k ≤ 14, -35 ≤ l ≤ 35
b/Å	10.3912(4)	Reflections collected	46533
c/Å	25.7698(10)	Independent reflections	8054 [R_{int} = 0.0470, R_{sigma} = 0.0306]
α/°	90	Data/restraints/parameters	8054/1/437
β/°	92.968(2)	Goodness-of-fit on F^2	1.098
γ/°	90	Final R indexes [I>=2σ (I)]	R_1 = 0.0409, wR_2 = 0.0974
Volume/Å^3	2821.92(19)	Final R indexes [all data]	R_1 = 0.0447, wR_2 = 0.0992
Z	2	Largest diff. peak/hole / e Å^{-3}	2.14/-1.59

2.9 Crystal Structure Data of Compound 15

Crystals were obtained from a saturated THF solution of **15** at -40 °C. X-ray structure analysis shows that **15** crystallizes in the monoclinic space group $P2_1$. The asymmetric unit contains one molecule of **15** and a THF molecule. The THF molecule was heavily disordered and therefore a solvent mask was calculated and 68 electrons were found in a volume of 320 Å^3 in 1 void per unit cell. This is consistent with the presence of 1 THF molecule per asymmetric unit, which accounts for 80 electrons per unit cell. The hydrogen atom on the phosphorus atom was located from the difference Fourier map and refined freely.

.

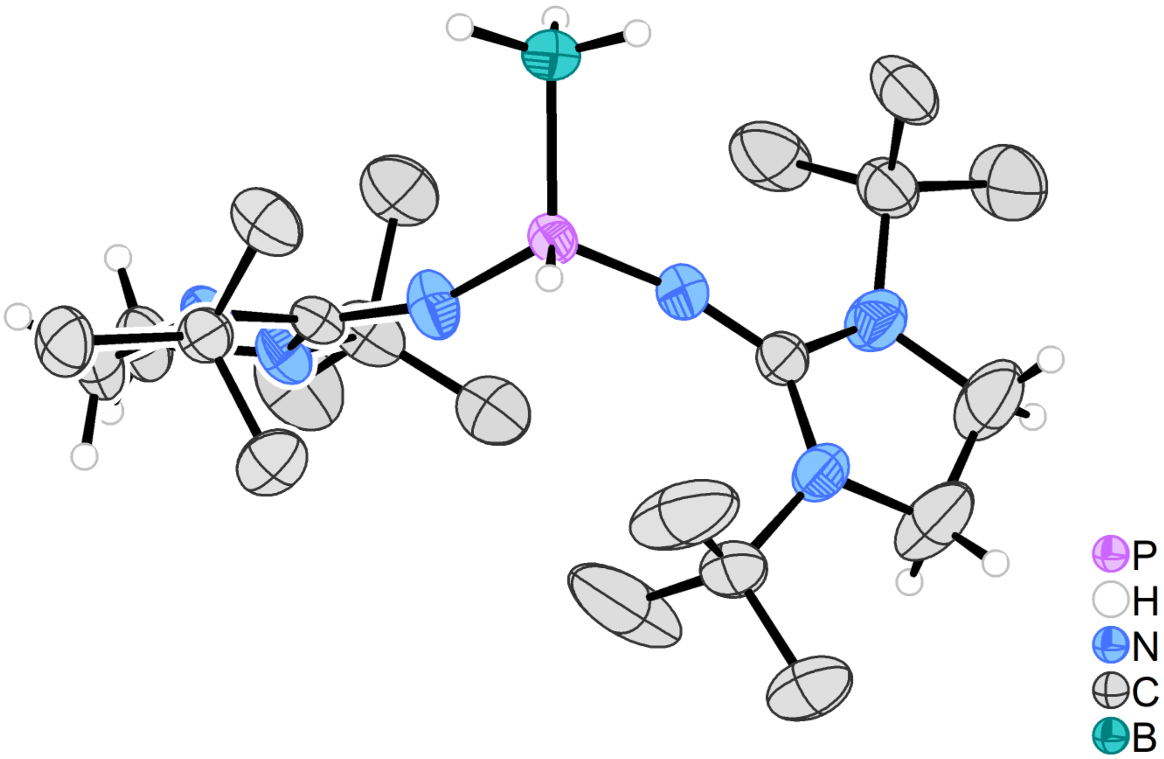

Figure S129: The asymmetric unit of the crystal structure of **15** with thermal ellipsoid plot at the 50% levels of probability. Hydrogen atoms except the phosphorus centered hydrogen atom, the hydrogen atoms in the NHI backbone, and the hydrogen atoms centered at the boron atom are omitted for clarity.

Table S9: Crystal data and structure refinement for **15**.

CCDC number	2489022	ρ_{calc} / g cm^{-3}	1.105
Empirical formula	$BC_{26}H_{56}N_6OP$	μ/mm^{-1}	0.117
Formula weight	510.54	F(000)	564.0
Temperature/K	173.00	Crystal size/mm^3	0.31 × 0.26 × 0.19
Crystal system	monoclinic	Radiation	MoK$_\alpha$ (λ = 0.71073)
Space group	$P2_1$	2Θ range for data collection/°	4.324 to 51.474
a/Å	9.2342(3)	Index ranges	-11 ≤ h ≤ 10, -23 ≤ k ≤ 23, -11 ≤ l ≤ 11
b/Å	18.8434(7)	Reflections collected	32226
c/Å	9.7156(3)	Independent reflections	5849 [R_{int} = 0.0410, R_{sigma} = 0.0357]
α/°	90	Data/restraints/parameters	5849/4/297
β/°	114.7810(10)	Goodness-of-fit on F^2	1.054
γ/°	90	Final R indexes [I>=2σ (I)]	R_1 = 0.0375, wR_2 = 0.1016
Volume/Å^3	1534.88(9)	Final R indexes [all data]	R_1 = 0.0386, wR_2 = 0.1029
Z	2	Largest diff. peak/hole / e Å^{-3}	0.31/-0.20
		Flack parameter	0.00(2)

2.10 Crystal Structure Data of Compound 16

Clear light yellow block-shaped crystals were obtained from slow diffusion of *n*-hexane into a saturated toluene solution of **16** at -40 °C. X-ray structure analysis shows that **16** crystallizes in the trigonal space group $R\overline{3}$ The asymmetric unit contains one molecule of **16** and two solvent molecules (DCM, *n*-hexane).

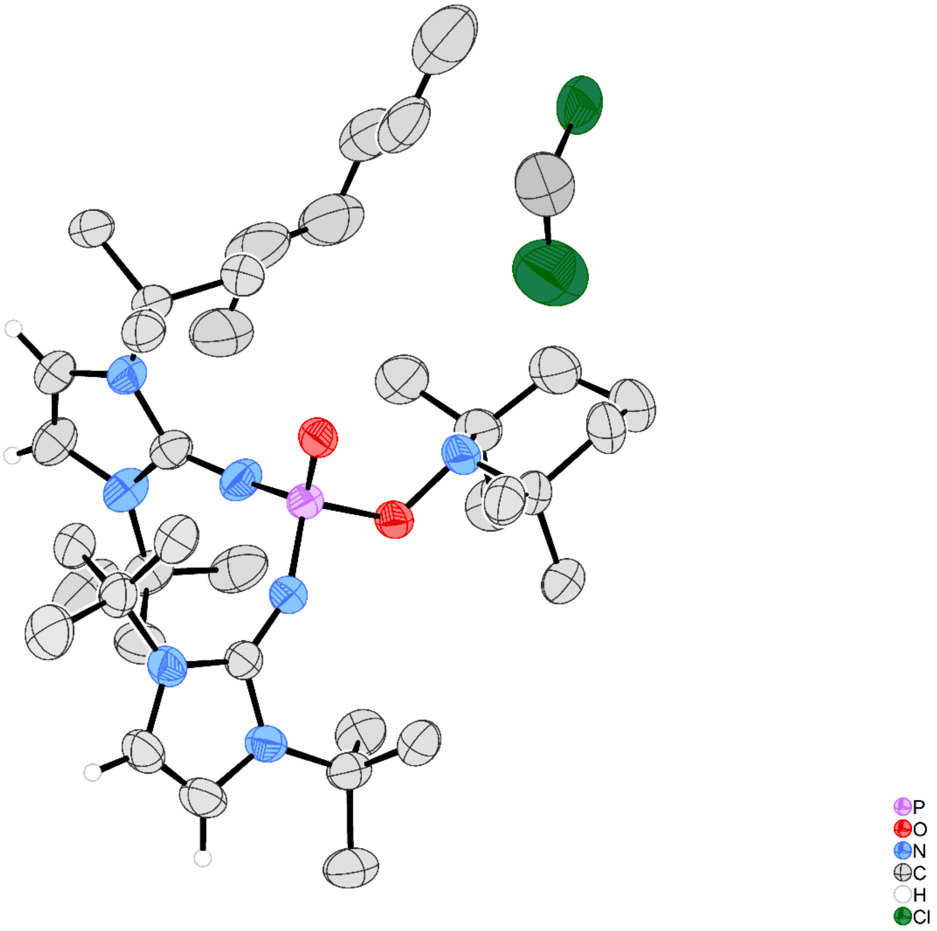

Figure S130: The asymmetric unit of the crystal structure of **16** with thermal ellipsoid plot at the 50% levels of probability. Hydrogen atoms except the hydrogen atoms in the NHI backbone are omitted for clarity.

Table S10: Crystal data and structure refinement for **16**.

CCDC number	2489017	ρ_{calc} / g cm^{-3}	1.108
Empirical formula	$C_{68.17}H_{130.33}Cl_{0.33}N_{14}O_4P_2$	μ/mm^{-1}	0.120
Formula weight	1283.95	F(000)	6345
Temperature/K	133.00	Crystal size/mm^3	0.16 × 0.08 × 0.04
Crystal system	trigonal	Radiation	MoK$_\alpha$ (λ = 0.71073)
Space group	$R\bar{3}$	2Θ range for data collection/°	4.428 to 50.156
a/Å	38.589(2)	Index ranges	-45 ≤ h ≤ 46, -46 ≤ k ≤ 46, -16 ≤ l ≤ 15
b/Å	38.589(2)	Reflections collected	134253
c/Å	13.4281(11)	Independent reflections	6833 [R_{int} = 0.1477, R_{sigma} = 0.0591]
α/°	90	Data/restraints/parameters	6833/79/469
β/°	90	Goodness-of-fit on F^2	1.008
γ/°	120	Final R indexes [I>=2σ (I)]	R_1 = 0.0471, wR_2 = 0.1084
Volume/Å^3	17317(3)	Final R indexes [all data]	R_1 = 0.0893, wR_2 = 0.1315
Z	9	Largest diff. peak/hole / e Å^{-3}	0.29/-0.30

2.11 Crystal Structure Data of Compound 18

Clear light yellow plate-shaped crystals were obtained from slow diffusion of *n*-hexane into a saturated toluene solution of **18** at -40 °C. X-ray structure analysis shows that **18** crystallizes in the orthorhombic space group $P2_12_12_1$. The asymmetric unit contains one molecule of **18**.

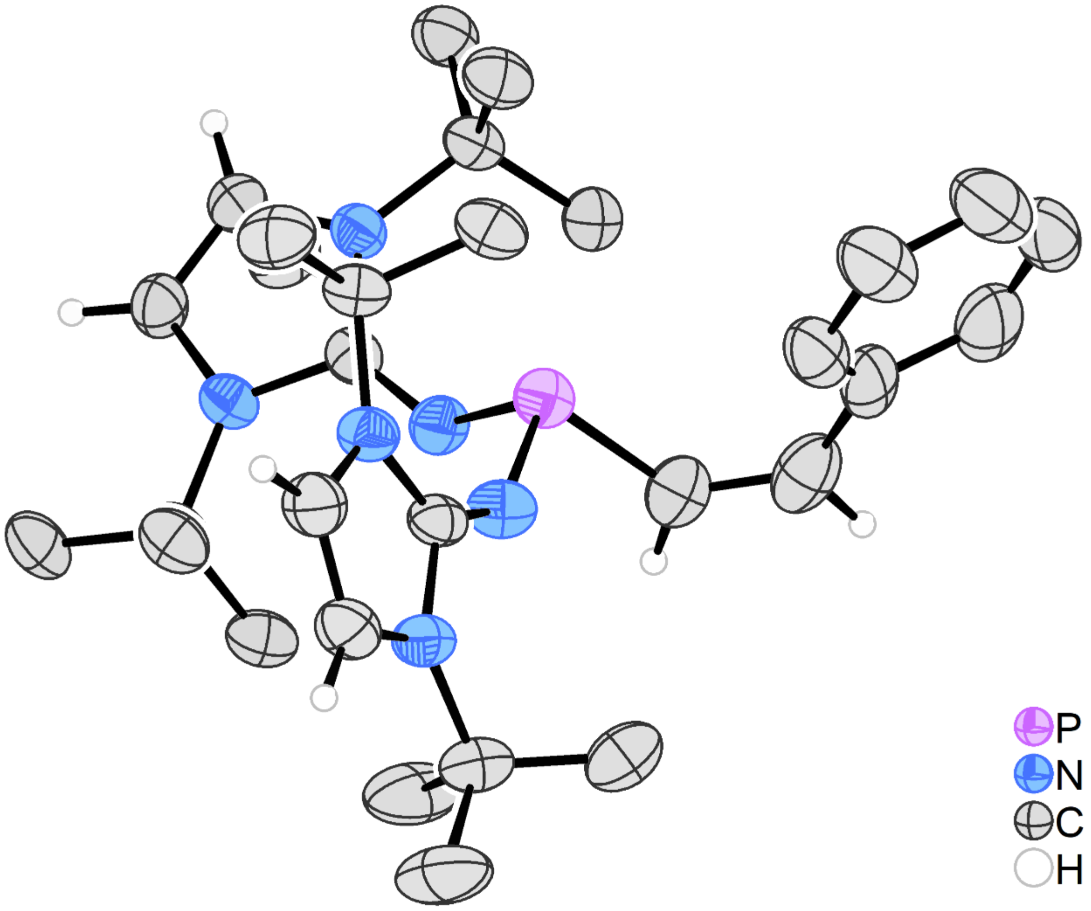

Figure S131: The asymmetric unit of the crystal structure of **18** with thermal ellipsoid plot at the 50% levels of probability. Hydrogen atoms except the alkene hydrogen atoms and the hydrogen atoms in the NHI backbone are omitted for clarity.

Table S11: Crystal data and structure refinement for **18**.

CCDC number	2489018	ρ_{calc} / g cm^{-3}	1.131
Empirical formula	$C_{30}H_{47}N_6P$	μ/mm^{-1}	0.118
Formula weight	522.70	F(000)	1136.0
Temperature/K	153.00	Crystal size/mm^3	0.29 × 0.19 × 0.07
Crystal system	orthorhombic	Radiation	MoK$_\alpha$ (λ = 0.71073)
Space group	$P2_12_12_1$	2Θ range for data collection/°	4.38 to 53.474
a/Å	11.1128(14)	Index ranges	-13 ≤ h ≤ 13, -20 ≤ k ≤ 20, -21 ≤ l ≤ 21
b/Å	16.243(2)	Reflections collected	36906
c/Å	16.9989(19)	Independent reflections	6240 [R_{int} = 0.0630, R_{sigma} = 0.0452]
α/°	90	Data/restraints/parameters	6240/0/347
β/°	90	Goodness-of-fit on F^2	1.086
γ/°	90	Final R indexes [I>=2σ (I)]	R_1 = 0.0465, wR_2 = 0.1074
Volume/Å^3	3068.4(7)	Final R indexes [all data]	R_1 = 0.0831, wR_2 = 0.1331
Z	4	Largest diff. peak/hole / e Å^{-3}	0.35/-0.31
		Flack parameter	0.03(4)

2.12 Crystal Structure Data of Compound 19

Clear yellow crystals were obtained from slow diffusion of *n*-hexane into a saturated toluene solution of **19** at -40 °C. X-ray structure analysis shows that **19** crystallizes in the triclinic space group $P\bar{1}$. The asymmetric unit contains one molecule of **19** in which the (Z) isomer has an occupation of 90% and the (E) isomer accordingly 10%. Additionally, a disordered water molecule is present. A solvent mask was calculated and 15 electrons were found in a volume of 123 Å^3 in 1 void per unit cell. This is consistent with the presence of 0.75 water molecules per asymmetric unit, which accounts for 15 electrons per unit cell.

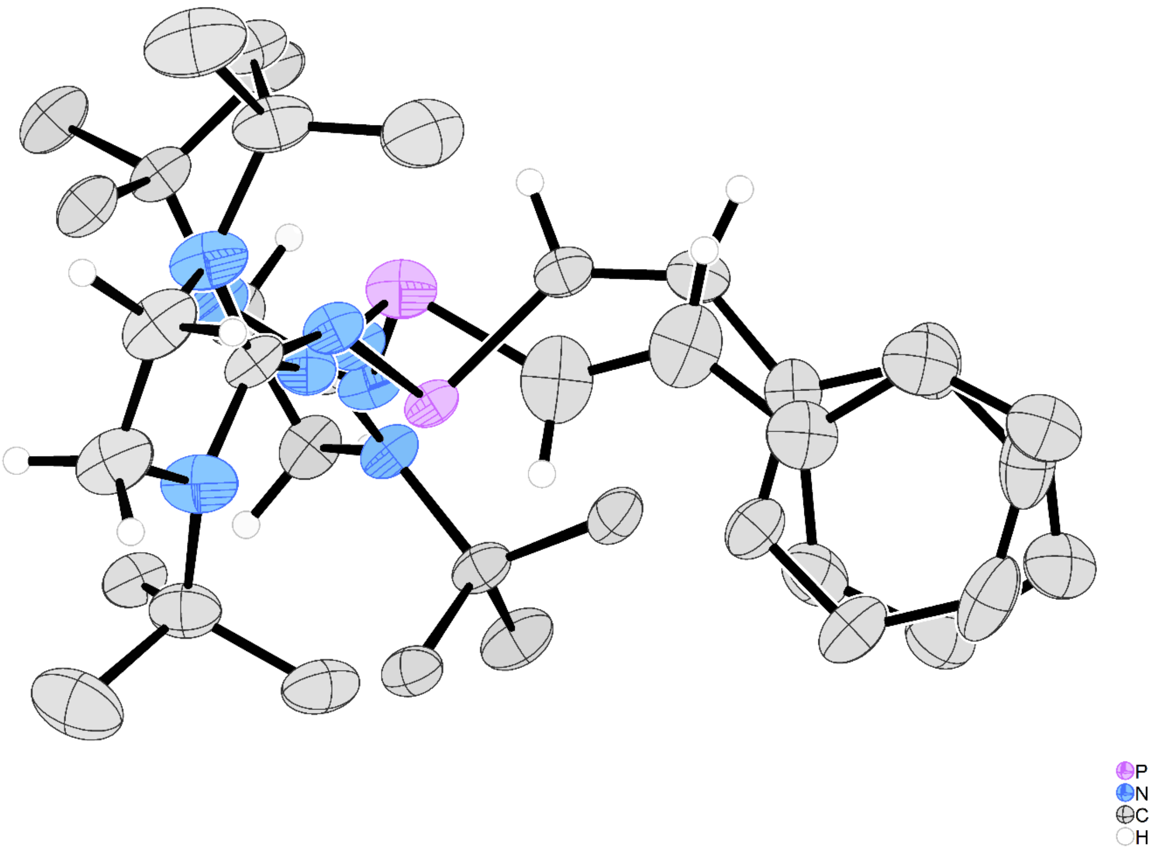

Figure S132: The asymmetric unit of the crystal structure of **19** with thermal ellipsoid plot at the 50% levels of probability. Hydrogen atoms except the alkene hydrogen atoms and the hydrogen atoms in the NHI backbone are omitted for clarity.

Table S12: Crystal data and structure refinement for **19**.

CCDC number	2489019	ρ_{calc} / g cm^{-3}	1.133
Empirical formula	$C_{30}H_{52.5}N_6O_{0.75}P$	μ/mm^{-1}	0.117
Formula weight	540.25	F(000)	591
Temperature/K	133.00	Crystal size/mm^3	0.35 × 0.20 × 0.12
Crystal system	triclinic	Radiation	MoK$_\alpha$ (λ = 0.71073)
Space group	$P\bar{1}$	2Θ range for data collection/°	4.440 to 56.608
a/Å	10.9145(7)	Index ranges	-14 ≤ h ≤ 14, -15 ≤ k ≤ 15, -16 ≤ l ≤ 16
b/Å	11.9958(8)	Reflections collected	32910
c/Å	12.7176(9)	Independent reflections	7795 [R_{int} = 0.0584, R_{sigma} = 0.0478]
α/°	81.459(2)	Data/restraints/parameters	7795/257/446
β/°	74.071(2)	Goodness-of-fit on F^2	1.045
γ/°	88.751(3)	Final R indexes [I>=2σ (I)]	R_1 = 0.0593, wR_2 = 0.1627
Volume/Å^3	1583.09(19)	Final R indexes [all data]	R_1 = 0.0769, wR_2 = 0.1775
Z	2	Largest diff. peak/hole / e Å^{-3}	0.67/-0.27

2.13 Crystal Structure Data of Compound [22]-CH_2Cl[Cl]

Yellow crystals of [**22**]-CH_2Cl[Cl] were obtained from slow diffusion of diethyl ether into a saturated DCM solution of **22** at -40 °C. X-ray structural analysis shows that [**22**]-CH_2Cl[Cl] crystallizes in the monoclinic space group $P2_1$. The asymmetric unit contains the salt [**22**]-CH_2Cl[Cl] and two molecules of DCM. The DCM molecules each show a disorder of one Cl atom over two positions (occupation 50:50). The hydrogen atom at the N3 nitrogen atom were located from the difference Fourier map and refined without any restraints.

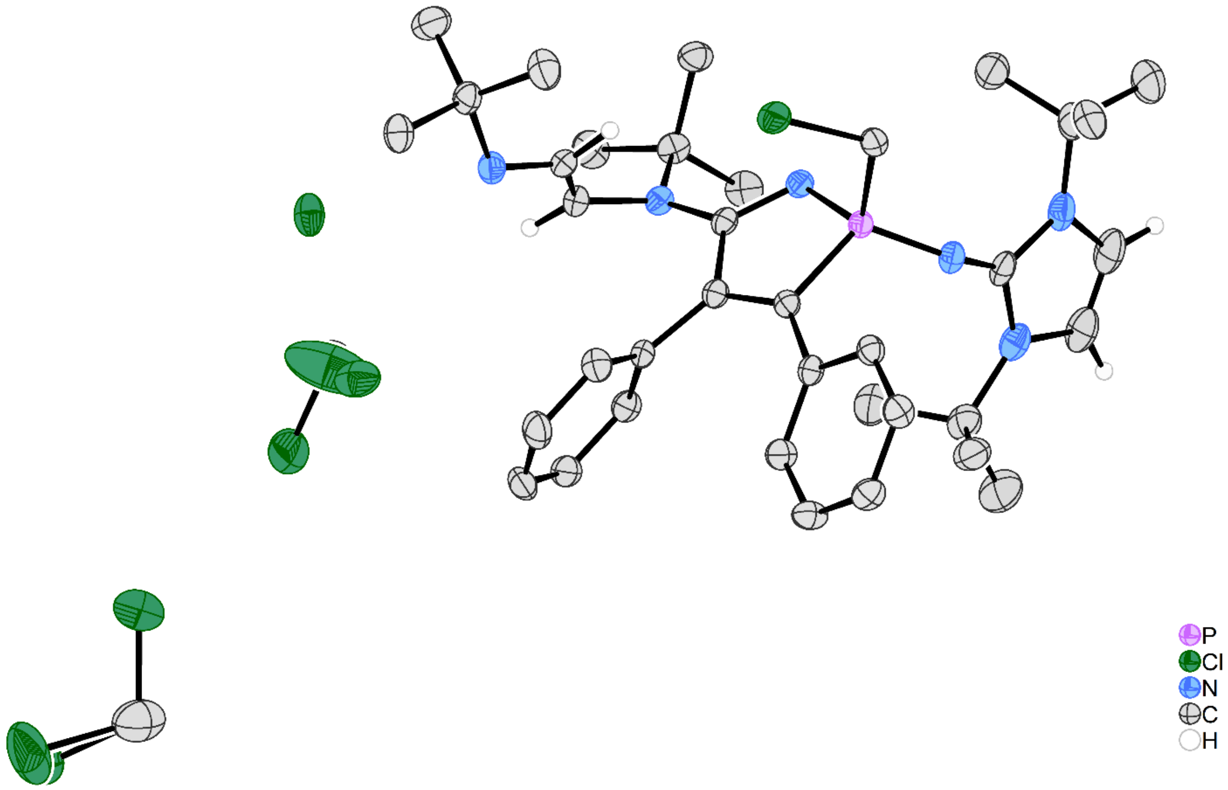

Figure S133: The asymmetric unit of the crystal structure of [**22**]-CH_2Cl[Cl] with thermal ellipsoid plot at the 50% levels of probability. Hydrogen atoms except the hydrogen atoms originating from the NHI backbone are omitted for clarity.

Table S13: Crystal data and structure refinement for [**22**]-$CH_2Cl[Cl]$.

CCDC number	2489020	ρ_{calc}g/cm^3	1.249
Empirical formula	$C_{39}H_{57}Cl_6N_6P$	μ/mm^{-1}	0.448
Formula weight	853.57	F(000)	900.0
Temperature/K	100	Crystal size/mm^3	0.715 × 0.633 × 0.215
Crystal system	monoclinic	Radiation	MoKα (λ = 0.71073)
Space group	$P2_1$	2Θ range for data collection/°	2.632 to 57.13
a/Å	9.5935(5)	Index ranges	-12 ≤ h ≤ 12, -20 ≤ k ≤ 20, -20 ≤ l ≤ 20
b/Å	15.2902(7)	Reflections collected	35411
c/Å	15.5000(7)	Independent reflections	11280 [R_{int} = 0.0447, R_{sigma} = 0.0447]
α/°	90	Data/restraints/parameters	11280/1/503
β/°	93.631(3)	Goodness-of-fit on F^2	1.033
γ/°	90	Final R indexes [I>=2σ (I)]	R_1 = 0.0453, wR_2 = 0.1100
Volume/Å^3	2269.08(19)	Final R indexes [all data]	R_1 = 0.0512, wR_2 = 0.1151
Z	2	Largest diff. peak/hole / e Å^{-3}	0.55/-0.35
		Flack parameter	-0.011(19)

3 Computational Details

General

All DFT calculations were performed using Gaussian16.[17]

3.1 Geometry Optimization and Natural Bond Orbital (NBO) Analysis

The geometry optimizations, frequency calculations and NBO analysis were carried out using the B3LYP functional[18,19] and the 6-311+g(d,p)[20–27] basis set. No imaginary frequencies were obtained, therefore confirming the calculated structure is a local minimum.

The Chemcraft software (v. 1.8)[28] was employed to render and depict the molecule and its orbitals.

3.1.1 Asymmetric P–H Vibration

Table S14: Calculated asymmetric P-H vibration.

Compound	v(P-H) / cm^{-1}
**1**	2128.4052
**3**	2261.3369

3.1.2 Depicted Frontier Orbitals

Table S15: **HOMO-3** to **HOMO** and **LUMO** to **LUMO+5** with the respective energies of **1** (left) and **3** (right) obtained at the B3LYP/6-311+g(d,p) level of theory.

**1**	**3**
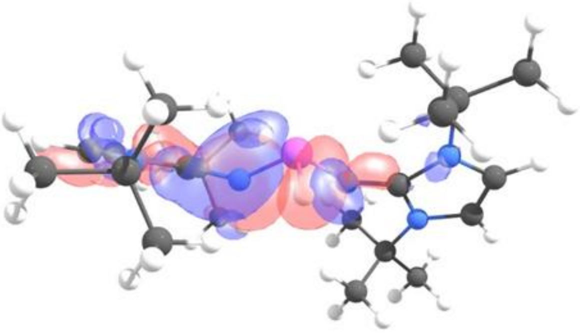 HOMO−3 (-6.015 eV)	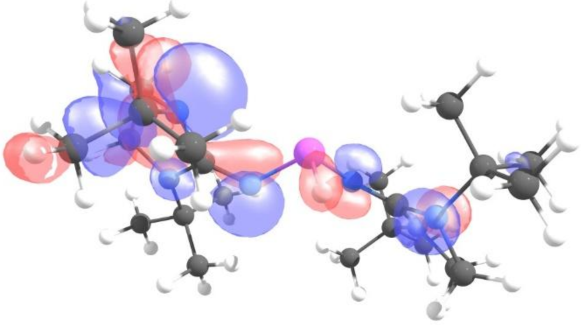 HOMO−3 (-6.068 eV)
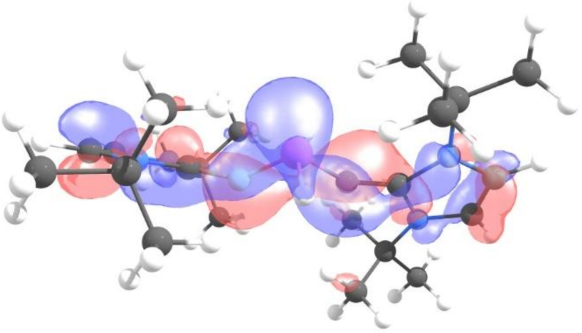 HOMO−2 (-5.712 eV)	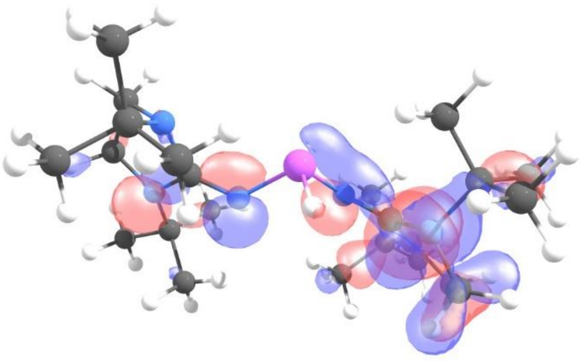 HOMO−2 (-5.991 eV)
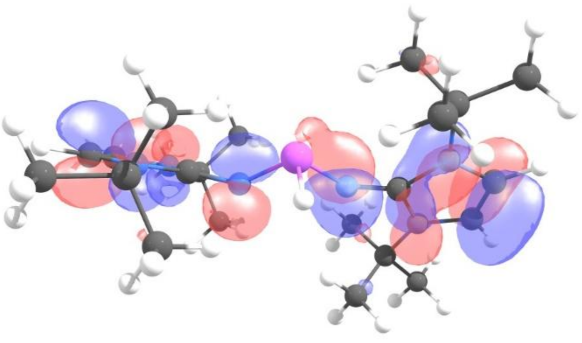 HOMO−1 (-4.792 eV)	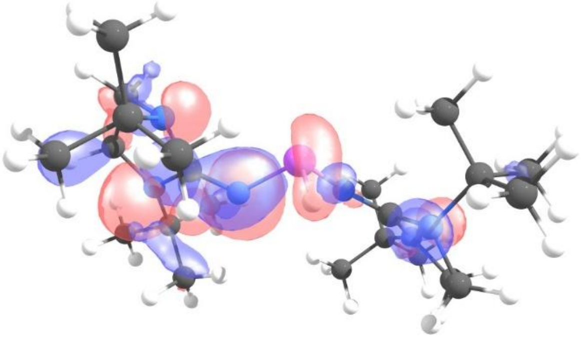 HOMO−1 (-5.809 eV)

**1**	**3**
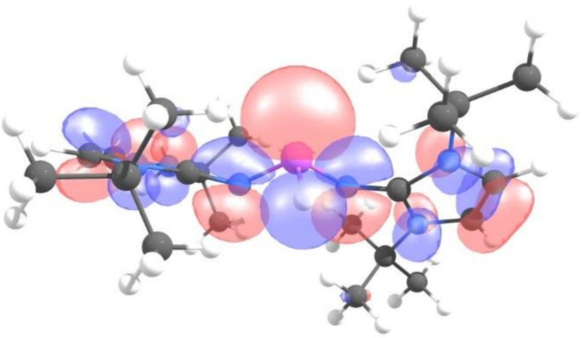	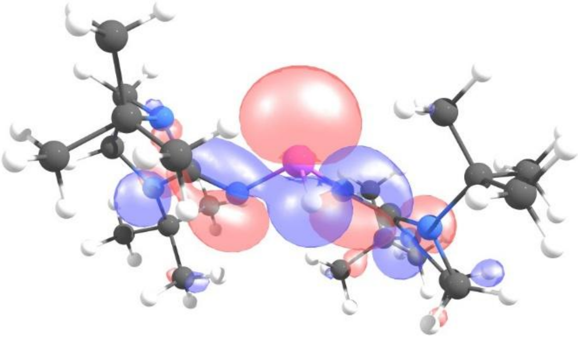
HOMO (-4.116 eV)	HOMO (-4.873 eV)
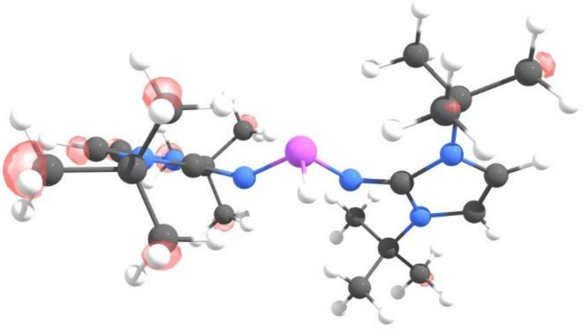	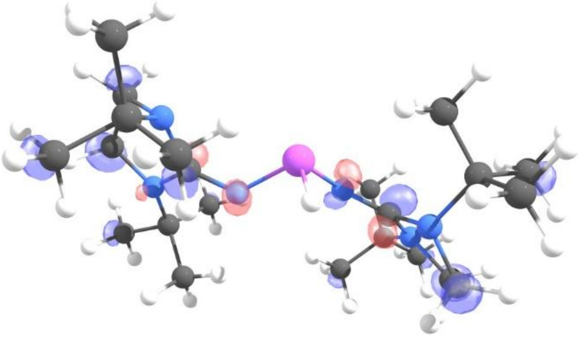
LUMO (-0.052 eV)	LUMO (-0.175 eV)
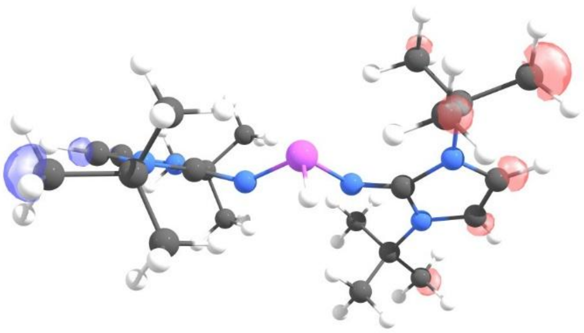	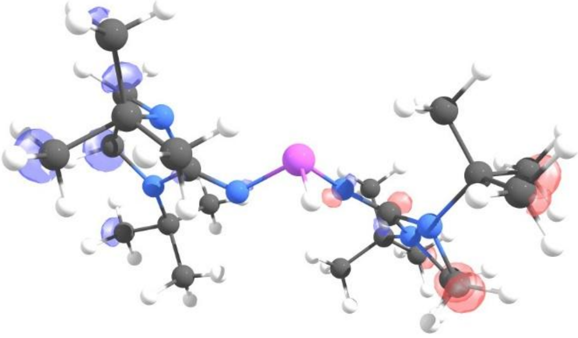
LUMO+1 (0.055 eV)	LUMO+1 (-0.091 eV)

Continuation of Table S15

1

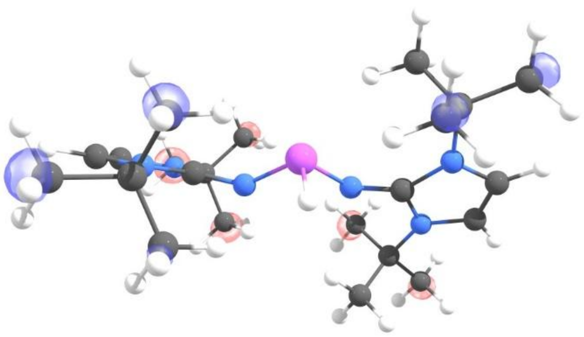

LUMO+2 (0.151 eV)

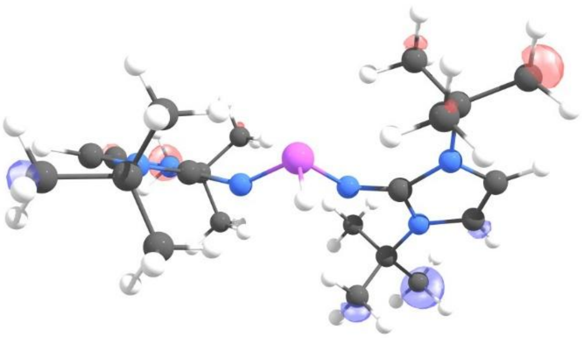

LUMO+3 (0.277 eV)

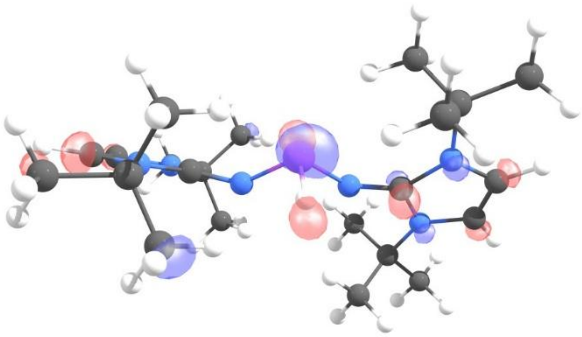

LUMO+4 (0.449 eV)

3

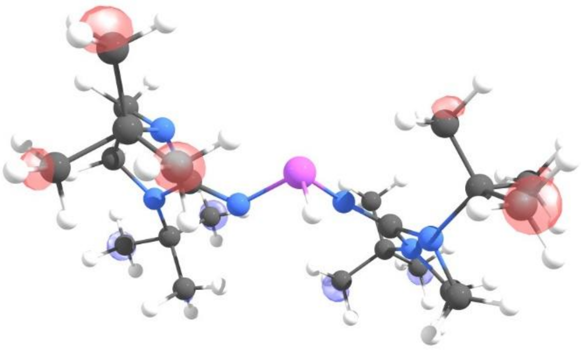

LUMO+2 (0.174 eV)

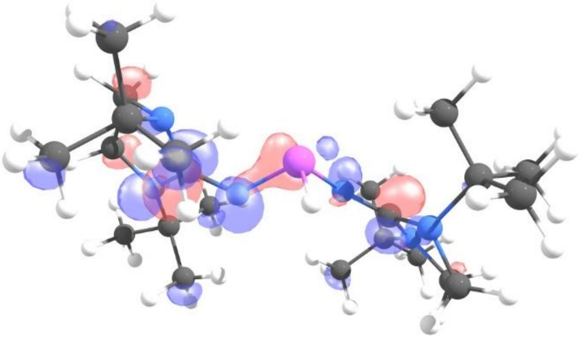

LUMO+3 (0.281 eV)

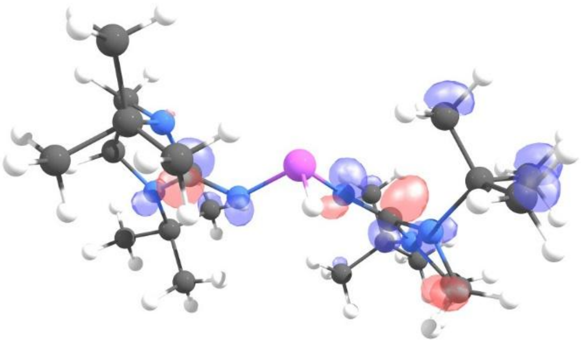

LUMO+4 (0.318 eV)

Continuation of Table S15

1	3
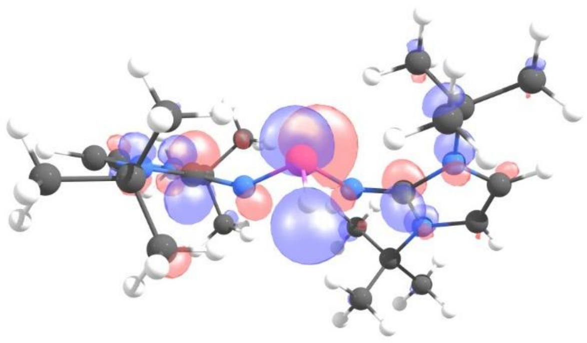	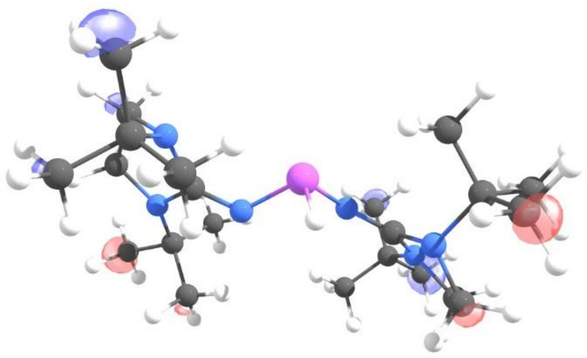
LUMO+5 (0.578 eV)	LUMO+5 (0.363 eV)

3.1.3 Natural Bond Orbital Analysis

The percentage of the total electron density of the calculated structures is 98.459% for **1** and 98.846% for **3**.

Specifically for P–H in **1**:

- occupancy of the natural bond orbital of 1.95167
- 45.04% localization on P and 54.96% localization on H
- composition of 0.6712 P ($sp^{7.12}d^{0.07}$) + 0.7413 H ($sp^{0.00}$) (P: s (12.20%), p (86.92%), d (0.88%), H: s (99.79%), p (0.21%))

Specifically for P–H in **3**:

- occupancy of the natural bond orbital of 1.96179
- 46.22% localization on P and 53.78% localization on H
- composition of 0.6799 P ($sp^{6.48}d^{0.06}$) + 0.7333 H ($sp^{0.00}$) (P: s (13.26%), p (85.94%), d (0.80%), H: s (99.78%), p (0.22%))

1 **3**

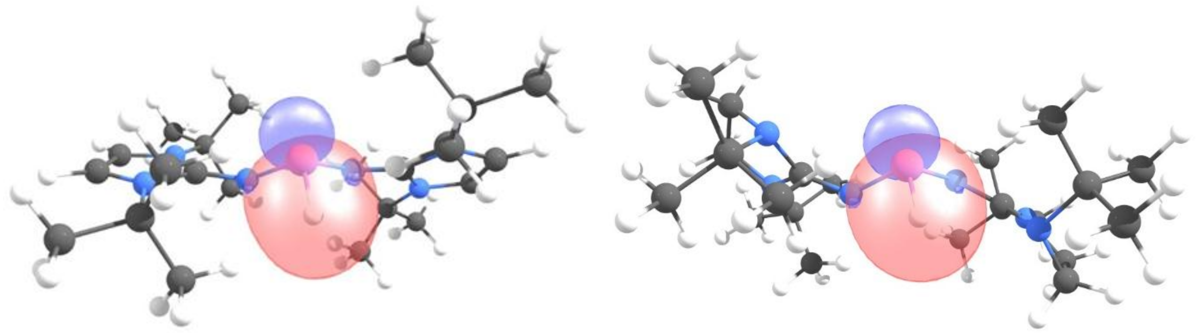

E = -11.130 eV *E* = -11.368 eV

Figure S134: Depicted natural bond orbitals of the P–H σ bond in **1** (left) and **3** (right) obtained at the B3LYP/6-311+g(d,p) level of theory.

Table S16: Charges of the hydrogen atom at the phosphorus center (*q*(H)) derived from NBO analysis at the B3LYP/6-311+g(d,p) level of theory.

Compound	Natural atomic charge	Mulliken charge
**1**	-0.11412	-0.109381
**3**	-0.08412	-0.070710

3.1.4 Comparison of Different Functionals and Basis Sets

To validate calculated energies for HOMO and LUMO with the chosen functional and basis set, different functionals (CAM-B3LYP,[29] HSEH1PBE,[30–36] wB97XD[37]) in combination with the triple zeta basis set 6-311+g(d,p)[20–27]) were tested. The combination of the functional B3LYP[18,19] and the double zeta basis set (6-31+g(d,p),[22–27,38–41] was also applied. As listed below the different functionals lead to divergent HOMO-LUMO energy gaps. The differences in the HOMO-LUMO energy gaps between **1** and **3** are rather consistent (0.6 eV to 0.8 eV) and show the same trend, independent from the used functional. Further, the ionization energies to describe the HOMO energies more precisely were calculated. The ionization energies show to be less dependent on the method used. The ionization energy differences between **1** and **3** are significantly consistent (0.85 eV to 0.87 eV). Overall, the ΔΔE (HOMO/ LUMO) and the ΔIonization energy show the same trend for both compounds.

Table S17: Application of different functionals and basis sets for the determination of the HOMO LUMO energy gap (ΔE (HOMO/ LUMO)) and ionization energy of **1** and **3**. All values in eV.

**Level of theory**	ΔE (HOMO/ LUMO) of **1**	Ionization energy of **1**	ΔE (HOMO/ LUMO) of **3**	Ionization energy of **3**	ΔΔE (HOMO/ LUMO) of **1** and **3**	ΔIonization energy of **1** and **3**
B3LYP/ 6-31+g(d,p)	4.041	5.373	4.679	6.250	0.638	0.877
B3LYP/ 6-311+g(d,p)	4.064	5.427	4.698	6.288	0.634	0.861
CAM-B3LYP/ 6-311+g(d,p)	6.117	5.671	6.908	6.539	0.791	0.868
HSEH1PBE/ 6-311+g(d,p)	3.746	5.404	4.371	6.259	0.625	0.855
wB97XD/ 6-311+g(d,p)	7.469	5.642	8.270	6.513	0.801	0.871

3.1.5 Atomic Coordinates

Table S18: Cartesian coordinates (x y z) of the refined geometry of **1** and **3** at the B3LYP/6-31+g(d,p) level of theory.

1

N	3.244421000	1.119852000	-0.669693000
C	2.587781000	0.028362000	-0.075532000
C	2.575642000	2.382426000	-1.103302000
C	4.610466000	0.868096000	-0.725139000
N	3.618048000	-0.890166000	0.217565000
N	1.322357000	-0.000836000	0.180171000
C	1.938686000	3.060638000	0.125135000
C	3.621843000	3.333268000	-1.710238000
C	1.519883000	2.065338000	-2.180791000
C	4.838766000	-0.351205000	-0.193909000
H	5.316547000	1.556893000	-1.153044000
C	3.481880000	-2.145789000	1.017021000
P	0.034829000	-1.047181000	-0.103949000
H	1.217149000	2.393497000	0.597920000
H	1.427582000	3.979510000	-0.181426000
H	2.711284000	3.324321000	0.856017000
H	4.391241000	3.618603000	-0.985549000
H	3.111485000	4.247951000	-2.024628000
H	4.105309000	2.903614000	-2.593862000
H	1.988124000	1.572729000	-3.039858000
H	1.059278000	2.996544000	-2.528123000
H	0.739735000	1.416052000	-1.785105000
H	5.772149000	-0.874908000	-0.095495000
C	2.763180000	-1.834863000	2.345721000
C	4.880765000	-2.695794000	1.355876000
C	2.742928000	-3.216920000	0.189828000
N	-1.288419000	-0.008983000	0.093259000
H	-0.073540000	-1.719813000	1.178888000
H	3.356025000	-1.128967000	2.937314000
H	2.650341000	-2.757739000	2.924219000
H	1.776050000	-1.402210000	2.187614000
H	5.441368000	-2.984693000	0.461157000
H	4.754802000	-3.594501000	1.966157000
H	5.474117000	-1.980823000	1.934038000
H	1.745204000	-2.895344000	-0.114330000
H	2.644634000	-4.137887000	0.775104000
H	3.309395000	-3.445849000	-0.719192000
C	-2.575282000	-0.030255000	0.108775000
N	-3.325743000	1.065920000	0.580874000
N	-3.550082000	-0.985939000	-0.272376000
C	-2.738744000	2.332965000	1.106932000
C	-4.682778000	0.780514000	0.490621000
C	-3.326229000	-2.278519000	-0.990326000
C	-4.822141000	-0.457888000	-0.021608000
C	-1.955648000	3.032544000	-0.020460000
C	-3.870012000	3.265710000	1.573796000
C	-1.827921000	2.024990000	2.313062000
H	-5.448740000	1.465889000	0.806196000
C	-2.538350000	-2.023768000	-2.290750000
C	-4.684579000	-2.895248000	-1.379637000
C	-2.614014000	-3.283407000	-0.062231000
H	-5.728132000	-1.002215000	-0.213539000
H	-1.159526000	2.385467000	-0.387771000
H	-1.513744000	3.961966000	0.354965000
H	-2.625442000	3.280985000	-0.851373000
H	-4.542148000	3.542732000	0.755013000
H	-3.419157000	4.186366000	1.954810000
H	-4.458713000	2.825309000	2.385202000
H	-2.402926000	1.538574000	3.108842000
H	-1.417381000	2.960012000	2.709871000
H	-1.006132000	1.371663000	2.019859000
H	-3.081634000	-1.316942000	-2.927081000
H	-2.425210000	-2.964642000	-2.840331000
H	-1.540028000	-1.631107000	-2.095633000
H	-5.298368000	-3.134620000	-0.505449000
H	-4.491429000	-3.831757000	-1.909980000
H	-5.254775000	-2.245864000	-2.050765000
H	-1.630290000	-2.938494000	0.252297000
H	-2.479111000	-4.237630000	-0.582648000
H	-3.218636000	-3.461067000	0.833281000

3

N	1.260096000	0.076797000	0.438343000
N	-1.259592000	0.054597000	-0.209247000
C	2.445962000	-0.039710000	-0.024442000
P	-0.046517000	-1.039726000	0.354365000
C	-2.503953000	-0.007617000	0.104577000
N	3.381635000	1.003901000	-0.027833000
N	3.021897000	-1.177084000	-0.696276000
H	0.276751000	-1.664028000	-0.897837000
N	-3.370602000	1.078577000	-0.026061000
N	-3.222742000	-1.108300000	0.681991000
C	3.090097000	2.393103000	0.428507000
C	4.389533000	0.763711000	-1.066649000
C	3.612810000	-2.295268000	0.130059000
C	3.936679000	-0.573818000	-1.683252000
C	-2.949686000	2.452368000	-0.430069000
C	-4.588942000	0.861090000	0.759064000
C	-3.637324000	-2.274939000	-0.180058000
C	-4.305925000	-0.478202000	1.459129000
C	2.564154000	2.373541000	1.877895000
C	4.407696000	3.195969000	0.429002000
C	2.068245000	3.063313000	-0.513053000
H	5.400754000	0.719959000	-0.642832000
H	4.378927000	1.554690000	-1.825327000
C	3.780731000	-3.509568000	-0.803002000
C	4.976637000	-1.912364000	0.755588000
C	2.663886000	-2.692301000	1.270198000
H	3.380638000	-0.393254000	-2.610541000
H	4.773037000	-1.229814000	-1.923641000
C	-4.208229000	3.326859000	-0.608732000
C	-2.039017000	3.066003000	0.652968000
C	-2.227008000	2.406891000	-1.791947000
H	-5.481195000	0.830374000	0.119735000
H	-4.734672000	1.654714000	1.499279000
C	-2.467382000	-2.764934000	-1.043755000
C	-4.041915000	-3.424663000	0.763165000
C	-4.813063000	-1.910064000	-1.118575000
H	-3.950617000	-0.304821000	2.481831000
H	-5.190956000	-1.110558000	1.520913000
H	1.620144000	1.835516000	1.948965000
H	2.418840000	3.403140000	2.223075000
H	3.292954000	1.891517000	2.538990000
H	5.166095000	2.709138000	1.051509000
H	4.217040000	4.189131000	0.846835000
H	4.819713000	3.338573000	-0.574218000
H	2.452344000	3.106969000	-1.539293000
H	1.866100000	4.090474000	-0.188697000
H	1.129997000	2.504905000	-0.513767000
H	2.815484000	-3.789284000	-1.235308000
H	4.173379000	-4.363856000	-0.241067000
H	4.476495000	-3.310973000	-1.624373000
H	5.737595000	-1.699022000	-0.001638000
H	5.352234000	-2.741302000	1.364982000
H	4.876163000	-1.035593000	1.402835000
H	2.500005000	-1.873955000	1.975726000
H	3.106920000	-3.528241000	1.821483000
H	1.691704000	-3.017935000	0.894216000
H	-4.901210000	2.883842000	-1.331931000
H	-3.904041000	4.304814000	-0.993515000
H	-4.744825000	3.499638000	0.328569000
H	-2.562003000	3.119659000	1.615350000
H	-1.746106000	4.084415000	0.373352000
H	-1.136970000	2.463386000	0.776492000
H	-1.319580000	1.807589000	-1.735535000
H	-1.969406000	3.426754000	-2.098500000
H	-2.885090000	1.977033000	-2.555495000
H	-2.118393000	-1.999593000	-1.740853000
H	-2.799916000	-3.628781000	-1.628687000
H	-1.628397000	-3.082779000	-0.420258000
H	-3.211009000	-3.671969000	1.430392000
H	-4.298505000	-4.314399000	0.178298000
H	-4.912567000	-3.177756000	1.378416000
H	-5.710955000	-1.617468000	-0.565018000
H	-5.081807000	-2.772639000	-1.737828000
H	-4.536102000	-1.088154000	-1.786682000

Table S19: Cartesian coordinates (x y z) of the refined geometry of **1** and **3** at the B3LYP/6-311+g(d,p) level of theory.

1

N	3.236970000	1.119433000	-0.677991000
C	2.584006000	0.033016000	-0.073296000
C	2.565781000	2.378966000	-1.117672000
C	4.602298000	0.870109000	-0.734629000
N	3.617043000	-0.878725000	0.223648000
N	1.323769000	0.000314000	0.186397000
C	1.934960000	3.064479000	0.107638000
C	3.608419000	3.324490000	-1.734824000
C	1.506715000	2.054997000	-2.187388000
C	4.834147000	-0.340690000	-0.195363000
H	5.305085000	1.555495000	-1.168246000
C	3.486106000	-2.131338000	1.029027000
P	0.037409000	-1.044355000	-0.095286000
H	1.217329000	2.401528000	0.587828000
H	1.422966000	3.979818000	-0.201527000
H	2.709653000	3.332592000	0.831964000
H	4.379682000	3.612963000	-1.016294000
H	3.098520000	4.235954000	-2.053187000
H	4.087520000	2.888541000	-2.615394000
H	1.970343000	1.555099000	-3.042382000
H	1.047058000	2.982193000	-2.541010000
H	0.728118000	1.411383000	-1.784768000
H	5.767773000	-0.859182000	-0.094156000
C	2.763357000	-1.819374000	2.353190000
C	4.886441000	-2.669578000	1.373238000
C	2.756464000	-3.209660000	0.206202000
N	-1.287667000	-0.008839000	0.101692000
H	-0.071101000	-1.714065000	1.188194000
H	3.348454000	-1.106562000	2.940852000
H	2.657517000	-2.738322000	2.935904000
H	1.774770000	-1.396287000	2.191312000
H	5.449142000	-2.958683000	0.482256000
H	4.766481000	-3.563969000	1.987911000
H	5.472729000	-1.947438000	1.946043000
H	1.759235000	-2.896360000	-0.102306000
H	2.662025000	-4.127261000	0.794097000
H	3.325928000	-3.439358000	-0.698622000
C	-2.570553000	-0.034487000	0.111469000
N	-3.328496000	1.054862000	0.584977000
N	-3.539930000	-0.990220000	-0.278529000
C	-2.750724000	2.322640000	1.120085000
C	-4.682574000	0.764162000	0.486378000
C	-3.309291000	-2.278242000	-1.002992000
C	-4.814007000	-0.468605000	-0.031165000
C	-1.963728000	3.028882000	0.002021000
C	-3.888472000	3.247192000	1.581973000
C	-1.848012000	2.014050000	2.329883000
H	-5.452088000	1.442912000	0.800674000
C	-2.518723000	-2.014190000	-2.297834000
C	-4.663337000	-2.895133000	-1.400479000
C	-2.599246000	-3.285862000	-0.079168000
H	-5.715523000	-1.013492000	-0.230831000
H	-1.164053000	2.387304000	-0.361380000
H	-1.529271000	3.957642000	0.382508000
H	-2.627740000	3.276575000	-0.831353000
H	-4.555367000	3.521951000	0.760698000
H	-3.446352000	4.167641000	1.968660000
H	-4.479526000	2.801067000	2.385938000
H	-2.425276000	1.522411000	3.118256000
H	-1.444544000	2.947138000	2.733507000
H	-1.023086000	1.366503000	2.040070000
H	-3.061262000	-1.306334000	-2.930526000
H	-2.401610000	-2.949725000	-2.852133000
H	-1.523801000	-1.620248000	-2.098217000
H	-5.278491000	-3.139120000	-0.530944000
H	-4.467069000	-3.826845000	-1.934515000
H	-5.231345000	-2.243532000	-2.068162000
H	-1.619596000	-2.941414000	0.242942000
H	-2.459231000	-4.234969000	-0.603891000
H	-3.207108000	-3.470951000	0.810416000

3

N	-1.258657000	0.074206000	-0.438122000
N	1.260306000	0.049225000	0.209878000
C	-2.439918000	-0.040937000	0.023993000
P	0.047153000	-1.040473000	-0.354920000
C	2.500087000	-0.007657000	-0.104269000
N	-3.373850000	1.002150000	0.034113000
N	-3.017219000	-1.178977000	0.691192000
H	-0.276955000	-1.666003000	0.895970000
N	3.361592000	1.081402000	0.018278000
N	3.223263000	-1.106585000	-0.675956000
C	-3.082327000	2.392537000	-0.418359000
C	-4.381678000	0.758625000	1.071125000
C	-3.604950000	-2.294655000	-0.139879000
C	-3.934548000	-0.583158000	1.678613000
C	2.935649000	2.454334000	0.419648000
C	4.582877000	0.863689000	-0.760491000
C	3.633629000	-2.272010000	0.188734000
C	4.309635000	-0.481040000	-1.450342000
C	-2.560641000	2.376930000	-1.867546000
C	-4.397422000	3.196173000	-0.412304000
C	-2.058584000	3.057940000	0.521902000
H	-5.391311000	0.720048000	0.648563000
H	-4.368208000	1.543334000	1.833192000
C	-3.774388000	-3.510841000	0.787705000
C	-4.966525000	-1.910421000	-0.765916000
C	-2.655409000	-2.687217000	-1.278710000
H	-3.384710000	-0.411970000	2.608877000
H	-4.772067000	-1.238272000	1.907640000
C	4.188303000	3.336465000	0.586542000
C	2.015864000	3.058415000	-0.658759000
C	2.222601000	2.408673000	1.784733000
H	5.471663000	0.840933000	-0.119597000
H	4.727334000	1.650765000	-1.504753000
C	2.461369000	-2.763185000	1.045454000
C	4.045305000	-3.420628000	-0.750047000
C	4.802440000	-1.904496000	1.132339000
H	3.963707000	-0.318786000	-2.475818000
H	5.195554000	-1.109837000	-1.497558000
H	-1.620754000	1.836260000	-1.942335000
H	-2.412352000	3.405447000	-2.209053000
H	-3.291582000	1.901132000	-2.527764000
H	-5.156338000	2.714344000	-1.034768000
H	-4.207058000	4.189683000	-0.824791000
H	-4.806190000	3.332557000	0.591069000
H	-2.439857000	3.099761000	1.547306000
H	-1.855097000	4.083769000	0.200338000
H	-1.123053000	2.498818000	0.519703000
H	-2.811354000	-3.791611000	1.219483000
H	-4.165615000	-4.362031000	0.223522000
H	-4.469763000	-3.314597000	1.607527000
H	-5.726546000	-1.697190000	-0.010402000
H	-5.341432000	-2.737289000	-1.375403000
H	-4.865022000	-1.034990000	-1.411752000
H	-2.488139000	-1.866246000	-1.977485000
H	-3.098443000	-3.517365000	-1.835253000
H	-1.686564000	-3.017268000	-0.903020000
H	4.886844000	2.902692000	1.307119000
H	3.880943000	4.313121000	0.967083000
H	4.717372000	3.505794000	-0.353486000
H	2.532469000	3.113390000	-1.622426000
H	1.717445000	4.073626000	-0.380484000
H	1.119209000	2.450217000	-0.776415000
H	1.323619000	1.799548000	1.736591000
H	1.955280000	3.425297000	2.087374000
H	2.889024000	1.991320000	2.545378000
H	2.106428000	-1.998427000	1.737075000
H	2.792038000	-3.623572000	1.633271000
H	1.628461000	-3.084199000	0.418490000
H	3.219827000	-3.669805000	-1.420234000
H	4.299756000	-4.308091000	-0.164121000
H	4.917064000	-3.172538000	-1.359951000
H	5.700281000	-1.608570000	0.584100000
H	5.070625000	-2.765377000	1.751114000
H	4.519920000	-1.085879000	1.799213000

Table S20: Cartesian coordinates (x y z) of the refined geometry of **1** and **3** at the CAM-B3LYP/6-311+g(d,p) level of theory.

1

N	3.211825183	1.120189569	-0.643833062
C	2.573309517	0.017713319	-0.078365555
C	2.527562540	2.371586181	-1.055283074
C	4.576681637	0.887469940	-0.701019358
N	3.603069382	-0.887060976	0.197773193
N	1.317334479	-0.037033001	0.172237894
C	1.875864110	3.012159448	0.173815310
C	3.556659040	3.343928013	-1.633732133
C	1.489829884	2.059891669	-2.138497626
C	4.815571720	-0.329132937	-0.196549619
H	5.273738674	1.592647034	-1.110803245
C	3.472988782	-2.152981387	0.964835257
P	0.034547918	-1.062655984	-0.135678607
H	1.163308836	2.326461009	0.627001786
H	1.354523415	3.926627334	-0.117676342
H	2.638077102	3.271514446	0.912822632
H	4.314829049	3.623577207	-0.899569192
H	3.036748505	4.254757456	-1.933214658
H	4.050456360	2.937852606	-2.519119771
H	1.970664156	1.592673006	-3.001073274
H	1.016687314	2.987008156	-2.470286093
H	0.720289798	1.391710235	-1.761406971
H	5.752425201	-0.842203637	-0.101268906
C	2.748760609	-1.877823505	2.287465869
C	4.867195549	-2.692888133	1.297123358
C	2.752549085	-3.207301221	0.117856648
N	-1.282772822	-0.036912245	0.072651317
H	-0.073421292	-1.756323176	1.125672766
H	3.334499366	-1.185796800	2.896976061
H	2.635598235	-2.811639990	2.842287227
H	1.764688357	-1.444057639	2.132694234
H	5.431340608	-2.956778609	0.400565476
H	4.748612515	-3.601884099	1.888162967
H	5.448712121	-1.983808929	1.888761128
H	1.754812703	-2.893166926	-0.182681898
H	2.663675231	-4.139216695	0.681336447
H	3.323423844	-3.409463509	-0.791273036
C	-2.559115263	-0.032336454	0.116129610
N	-3.277921517	1.074169389	0.581986715
N	-3.544916517	-0.972050869	-0.228046831
C	-2.662497839	2.336998076	1.058319255
C	-4.636142760	0.808747718	0.522563000
C	-3.347057758	-2.262957982	-0.937169082
C	-4.801027707	-0.425317359	0.036663696
C	-1.909049937	2.995373829	-0.100889112
C	-3.762416378	3.287892915	1.533473719
C	-1.725109921	2.047922738	2.236328561
H	-5.384120323	1.508328396	0.842237689
C	-2.591791709	-2.023509489	-2.248798146
C	-4.710952177	-2.866485111	-1.289247899
C	-2.627023172	-3.262338703	-0.025351744
H	-5.715250341	-0.959356627	-0.130248546
H	-1.135094158	2.329714888	-0.474155437
H	-1.445123799	3.924848478	0.237333900
H	-2.598929046	3.231357200	-0.915010605
H	-4.451381566	3.553659245	0.729096831
H	-3.292378042	4.208367995	1.882066523
H	-4.331745898	2.870480251	2.366594023
H	-2.280018757	1.585649294	3.056422087
H	-1.295448665	2.984505544	2.599728638
H	-0.920932331	1.381336382	1.934363882
H	-3.146455293	-1.321505417	-2.875677798
H	-2.495368079	-2.966341646	-2.792239680
H	-1.590800972	-1.632918830	-2.082080256
H	-5.304673461	-3.089913440	-0.400853337
H	-4.539594863	-3.806894488	-1.814230201
H	-5.287375985	-2.217476531	-1.950467593
H	-1.639969704	-2.922779299	0.273596364
H	-2.505872269	-4.215761078	-0.544418575
H	-3.216429692	-3.434731511	0.877803697

3

N	-1.253675000	0.047764000	-0.473497000
N	1.241747000	0.029169000	0.191204000
C	-2.419806000	-0.048396000	0.011819000
P	0.046603000	-1.058217000	-0.385008000
C	2.478012000	-0.014566000	-0.111034000
N	-3.335656000	0.999647000	0.032272000
N	-2.995040000	-1.171052000	0.689374000
H	-0.284583000	-1.687923000	0.854949000
N	3.325484000	1.073580000	0.025908000
N	3.210219000	-1.099768000	-0.677456000
C	-3.025992000	2.376916000	-0.417938000
C	-4.334002000	0.770253000	1.071626000
C	-3.594526000	-2.273000000	-0.131068000
C	-3.896383000	-0.568568000	1.676777000
C	2.881096000	2.432048000	0.421844000
C	4.552346000	0.870275000	-0.735443000
C	3.616727000	-2.249428000	0.191135000
C	4.296713000	-0.467704000	-1.432381000
C	-2.529992000	2.359291000	-1.868174000
C	-4.318713000	3.201364000	-0.387666000
C	-1.979619000	3.012658000	0.504974000
H	-5.345826000	0.740392000	0.656640000
H	-4.306522000	1.555484000	1.830761000
C	-3.790148000	-3.477619000	0.793873000
C	-4.938252000	-1.864063000	-0.759800000
C	-2.650842000	-2.687866000	-1.257372000
H	-3.341661000	-0.400764000	2.603113000
H	-4.737279000	-1.215946000	1.910144000
C	4.115857000	3.324186000	0.598355000
C	1.968001000	3.020503000	-0.659791000
C	2.160065000	2.379494000	1.773889000
H	5.432220000	0.850523000	-0.084543000
H	4.698860000	1.660877000	-1.473594000
C	2.441643000	-2.748825000	1.026790000
C	4.055050000	-3.392680000	-0.729258000
C	4.759274000	-1.862867000	1.146088000
H	3.963836000	-0.302832000	-2.460206000
H	5.185313000	-1.091458000	-1.470395000
H	-1.600058000	1.806077000	-1.959377000
H	-2.373315000	3.385917000	-2.207951000
H	-3.277770000	1.896094000	-2.516634000
H	-5.095298000	2.734796000	-0.998031000
H	-4.116732000	4.191007000	-0.800718000
H	-4.707317000	3.342118000	0.621803000
H	-2.346049000	3.058194000	1.534584000
H	-1.757852000	4.033626000	0.184131000
H	-1.058108000	2.432226000	0.487129000
H	-2.835376000	-3.770730000	1.233136000
H	-4.188439000	-4.322800000	0.227994000
H	-4.488793000	-3.269739000	1.606264000
H	-5.695435000	-1.636207000	-0.007183000
H	-5.326110000	-2.681739000	-1.371161000
H	-4.815610000	-0.990124000	-1.402584000
H	-2.460800000	-1.874486000	-1.957523000
H	-3.107215000	-3.510099000	-1.812405000
H	-1.693915000	-3.037251000	-0.871603000
H	4.813000000	2.895564000	1.321792000
H	3.794979000	4.295048000	0.979131000
H	4.648384000	3.501305000	-0.336890000
H	2.493503000	3.081431000	-1.617127000
H	1.653384000	4.030363000	-0.384705000
H	1.081785000	2.399916000	-0.785331000
H	1.267724000	1.763107000	1.717130000
H	1.881899000	3.392631000	2.074282000
H	2.823420000	1.967369000	2.538523000
H	2.065929000	-1.985906000	1.707945000
H	2.772873000	-3.601556000	1.623060000
H	1.625121000	-3.082623000	0.386851000
H	3.243977000	-3.652578000	-1.411154000
H	4.307536000	-4.273187000	-0.134275000
H	4.933746000	-3.138973000	-1.324495000
H	5.662172000	-1.563730000	0.610165000
H	5.024181000	-2.713685000	1.777661000
H	4.456576000	-1.040299000	1.797707000

Table S21: Cartesian coordinates (x y z) of the refined geometry of **1** and **3** at the HSEH1PBE/6-311+g(d,p) level of theory.

1

N	3.204793873	1.135223738	-0.647208462
C	2.570197949	0.041434163	-0.062771995
C	2.516330690	2.378596097	-1.061616437
C	4.564455441	0.902300275	-0.719429266
N	3.600869169	-0.862784372	0.205909019
N	1.314455458	0.000360692	0.211834969
C	1.881655278	3.030163740	0.168955122
C	3.535503888	3.342513379	-1.665176014
C	1.462960126	2.051879528	-2.123022070
C	4.807381544	-0.316133542	-0.205644462
H	5.257549804	1.605041487	-1.144357692
C	3.470174184	-2.123708942	0.972375059
P	0.043001461	-1.046275535	-0.079452515
H	1.176380710	2.343634710	0.638417240
H	1.352615102	3.941104094	-0.125751480
H	2.654433225	3.299784036	0.895467216
H	4.304991623	3.632183211	-0.943897089
H	3.009649026	4.250749579	-1.968119379
H	4.017290319	2.925897942	-2.554579806
H	1.931102669	1.576765350	-2.990209445
H	0.979749292	2.975228924	-2.456019607
H	0.702108971	1.383148380	-1.722244108
H	5.744421924	-0.833694636	-0.116236652
C	2.759504104	-1.841910489	2.299140238
C	4.862190395	-2.671227374	1.290670745
C	2.733234300	-3.169797323	0.132125793
N	-1.273807692	-0.015251007	0.104757553
H	-0.064885038	-1.720317750	1.204379420
H	3.354565079	-1.150965713	2.903628607
H	2.644827277	-2.775725857	2.856688310
H	1.774154200	-1.402137541	2.148697956
H	5.416842104	-2.940366039	0.387370003
H	4.742844674	-3.580545259	1.884125557
H	5.454801046	-1.964451165	1.877678622
H	1.735700419	-2.838945034	-0.166108294
H	2.633253879	-4.100262904	0.699255685
H	3.297583252	-3.381923241	-0.780826082
C	-2.555284898	-0.041319364	0.112839919
N	-3.305801321	1.049263148	0.562047533
N	-3.514647991	-0.995405594	-0.259447577
C	-2.724237123	2.314457512	1.062475090
C	-4.653589607	0.764828493	0.465257252
C	-3.272566334	-2.282429302	-0.953304612
C	-4.784008181	-0.475820225	-0.030010329
C	-1.941957782	2.985999830	-0.067844955
C	-3.850087962	3.246642337	1.505571716
C	-1.821057540	2.029224995	2.265960857
H	-5.421459017	1.453472613	0.766667342
C	-2.483350088	-2.034270049	-2.241019357
C	-4.612739653	-2.911729189	-1.339806202
C	-2.559148612	-3.260509654	-0.016070501
H	-5.684015812	-1.027816600	-0.225097079
H	-1.147215757	2.327221331	-0.416384270
H	-1.499584092	3.920294077	0.290602503
H	-2.607794564	3.218023008	-0.904865097
H	-4.517688560	3.507769761	0.679325017
H	-3.402374486	4.172947516	1.872721660
H	-4.440484504	2.818013586	2.320583554
H	-2.396617228	1.559959104	3.069687751
H	-1.408132378	2.969396671	2.643822422
H	-1.002723755	1.367956209	1.981772380
H	-3.027236247	-1.337616446	-2.885743707
H	-2.358796305	-2.977800662	-2.780753047
H	-1.489113394	-1.633031645	-2.039841317
H	-5.228120177	-3.143800898	-0.466024623
H	-4.407935405	-3.852279147	-1.855917425
H	-5.182969785	-2.275688293	-2.021919174
H	-1.583029766	-2.898182146	0.303473818
H	-2.405788573	-4.216156722	-0.525719761
H	-3.168312870	-3.437706545	0.874852984

3

N	-1.240564000	0.066228000	-0.464084000
N	1.242218000	0.039553000	0.229351000
C	-2.413459000	-0.045746000	0.015185000
P	0.051289000	-1.049305000	-0.354475000
C	2.477774000	-0.008609000	-0.098514000
N	-3.340264000	0.990194000	0.027954000
N	-2.976790000	-1.173639000	0.686531000
H	-0.304356000	-1.673948000	0.890782000
N	3.324110000	1.079050000	0.020138000
N	3.196464000	-1.094293000	-0.670681000
C	-3.036982000	2.370902000	-0.405240000
C	-4.343685000	0.747679000	1.054266000
C	-3.559269000	-2.273232000	-0.144715000
C	-3.895111000	-0.585096000	1.661957000
C	2.880523000	2.437988000	0.403660000
C	4.553106000	0.862083000	-0.725699000
C	3.592056000	-2.248698000	0.191737000
C	4.288646000	-0.473159000	-1.420721000
C	-2.518665000	2.367690000	-1.845888000
C	-4.335344000	3.182865000	-0.388231000
C	-2.010459000	3.004884000	0.538593000
H	-5.353630000	0.708047000	0.629110000
H	-4.332379000	1.534242000	1.816053000
C	-3.738623000	-3.486984000	0.768089000
C	-4.905908000	-1.876665000	-0.770922000
C	-2.609253000	-2.659535000	-1.273031000
H	-3.351986000	-0.410919000	2.596275000
H	-4.730749000	-1.246816000	1.886213000
C	4.109180000	3.340936000	0.544882000
C	1.944759000	3.001589000	-0.669723000
C	2.183918000	2.397729000	1.767174000
H	5.429113000	0.835603000	-0.065255000
H	4.715531000	1.650750000	-1.465714000
C	2.413435000	-2.738611000	1.023949000
C	4.019171000	-3.390512000	-0.732615000
C	4.736535000	-1.874340000	1.146223000
H	3.960661000	-0.306832000	-2.452064000
H	5.173660000	-1.106735000	-1.454881000
H	-1.585891000	1.812574000	-1.925641000
H	-2.355839000	3.399258000	-2.172646000
H	-3.257222000	1.910917000	-2.511952000
H	-5.099005000	2.715949000	-1.017437000
H	-4.133816000	4.179588000	-0.788834000
H	-4.742758000	3.311180000	0.617685000
H	-2.394231000	3.040492000	1.564076000
H	-1.788465000	4.030976000	0.228997000
H	-1.084405000	2.427262000	0.529928000
H	-2.778009000	-3.773743000	1.203030000
H	-4.130359000	-4.332529000	0.195127000
H	-4.438636000	-3.293056000	1.585638000
H	-5.670138000	-1.669755000	-0.016263000
H	-5.281401000	-2.692914000	-1.394979000
H	-4.793187000	-0.991390000	-1.403245000
H	-2.436646000	-1.834482000	-1.967423000
H	-3.049363000	-3.488317000	-1.834889000
H	-1.641245000	-2.990775000	-0.891395000
H	4.824843000	2.929650000	1.262859000
H	3.784726000	4.315154000	0.918864000
H	4.623530000	3.511613000	-0.404000000
H	2.454974000	3.053943000	-1.637625000
H	1.623800000	4.013481000	-0.402996000
H	1.061997000	2.367811000	-0.770625000
H	1.301423000	1.761156000	1.733370000
H	1.890295000	3.412280000	2.053660000
H	2.868302000	2.012044000	2.529380000
H	2.048429000	-1.974762000	1.713203000
H	2.733917000	-3.603337000	1.612029000
H	1.589719000	-3.053916000	0.379024000
H	3.203509000	-3.641779000	-1.414998000
H	4.266247000	-4.276246000	-0.140244000
H	4.900735000	-3.141393000	-1.329400000
H	5.642914000	-1.579219000	0.609496000
H	4.997336000	-2.729706000	1.776453000
H	4.438425000	-1.050431000	1.801421000

Table S22: Cartesian coordinates (x y z) of the refined geometry of **1** and **3** at the wB97XD/6-311+g(d,p) level of theory.

1

N	3.137570540	1.115587468	-0.665474205
C	2.551146880	0.001655858	-0.075509246
C	2.395555751	2.332923591	-1.072808207
C	4.507917159	0.931958268	-0.741675766
N	3.610631801	-0.862671281	0.193268148
N	1.305004114	-0.096863882	0.219030365
C	1.743994976	2.957948048	0.166096215
C	3.373759719	3.339888136	-1.683212915
C	1.345459392	1.968632861	-2.129952043
C	4.796837057	-0.272419990	-0.225408576
H	5.174305655	1.656493163	-1.170848448
C	3.515560864	-2.134647814	0.951031616
P	0.038917862	-1.103868880	-0.177716960
H	1.054049609	2.252632999	0.626808483
H	1.193772776	3.857809552	-0.121405087
H	2.510207717	3.236626287	0.895389374
H	4.134522601	3.658797800	-0.965784860
H	2.810603512	4.226207772	-1.982118891
H	3.865604587	2.942185753	-2.575191826
H	1.823953161	1.497304131	-2.992842578
H	0.838943116	2.877006927	-2.468328011
H	0.599010469	1.287690403	-1.725234288
H	5.753099161	-0.752263735	-0.137976737
C	2.783061307	-1.891139753	2.277396583
C	4.926307161	-2.638991171	1.275506197
C	2.815007727	-3.197691595	0.096266012
N	-1.266063035	-0.060035473	-0.052026560
H	-0.145495550	-1.800768122	1.077609046
H	3.332840146	-1.162641289	2.879760459
H	2.726467002	-2.829066358	2.836041613
H	1.774098795	-1.513586476	2.123728490
H	5.493573228	-2.884428453	0.373929924
H	4.834787543	-3.554475898	1.863370558
H	5.490568401	-1.915380760	1.869077550
H	1.802869141	-2.903875378	-0.182573930
H	2.760493945	-4.140359934	0.648252084
H	3.378340076	-3.368337091	-0.825243400
C	-2.532601123	-0.035638110	0.114547224
N	-3.190950983	1.076820546	0.640505421
N	-3.551224728	-0.956713513	-0.158053236
C	-2.508671706	2.320341323	1.067204395
C	-4.553248853	0.835746616	0.683148402
C	-3.414552622	-2.220894741	-0.923679883
C	-4.774531347	-0.395890982	0.204467878
C	-1.847598642	2.970703459	-0.153795912
C	-3.541668390	3.290770116	1.645832781
C	-1.474648133	1.998498435	2.154966140
H	-5.263373606	1.544389545	1.066471029
C	-2.702689920	-1.946522460	-2.254837784
C	-4.808501267	-2.775865364	-1.242725620
C	-2.677835729	-3.270941765	-0.083421039
H	-5.707454607	-0.917388505	0.108932153
H	-1.116766982	2.293629053	-0.591955800
H	-1.341775355	3.892757000	0.145775691
H	-2.603030741	3.216961494	-0.905702033
H	-4.291543366	3.581791811	0.905188297
H	-3.021112629	4.197126085	1.961777746
H	-4.046015346	2.874352045	2.522044566
H	-1.967054109	1.544223304	3.019632616
H	-0.989422790	2.923050926	2.480785118
H	-0.716073493	1.313388229	1.779119253
H	-3.253187651	-1.192190166	-2.823747433
H	-2.668879573	-2.867249238	-2.843569916
H	-1.679323420	-1.602804718	-2.112573497
H	-5.366587940	-3.033494800	-0.338800200
H	-4.684643490	-3.692369699	-1.822828490
H	-5.397732349	-2.078251373	-1.842739541
H	-1.660449096	-2.970956638	0.156843269
H	-2.623794619	-4.213613840	-0.634889247
H	-3.215297385	-3.446293388	0.852203065

3

N	-1.230946000	-0.032229000	-0.548106000
N	1.207040000	-0.064202000	0.191591000
C	-2.383058000	-0.084740000	-0.019469000
P	0.050957000	-1.150351000	-0.443520000
C	2.446123000	-0.049566000	-0.112155000
N	-3.244947000	1.003622000	0.047251000
N	-2.983960000	-1.195068000	0.646371000
H	-0.320572000	-1.812717000	0.772457000
N	3.227021000	1.085161000	0.005565000
N	3.234432000	-1.108118000	-0.641721000
C	-2.845148000	2.380387000	-0.321801000
C	-4.242495000	0.784775000	1.087882000
C	-3.630553000	-2.248094000	-0.197783000
C	-3.848726000	-0.583107000	1.657875000
C	2.682325000	2.425029000	0.326692000
C	4.496525000	0.913867000	-0.685910000
C	3.651395000	-2.215759000	0.272174000
C	4.321300000	-0.445073000	-1.367608000
C	-2.343125000	2.418379000	-1.771558000
C	-4.083526000	3.284302000	-0.245247000
C	-1.761957000	2.884059000	0.642134000
H	-5.257921000	0.801885000	0.677349000
H	-4.179586000	1.549395000	1.866892000
C	-3.879201000	-3.466054000	0.698416000
C	-4.953418000	-1.756463000	-0.815206000
C	-2.704050000	-2.678587000	-1.335274000
H	-3.273914000	-0.457627000	2.579649000
H	-4.713049000	-1.201897000	1.889873000
C	3.843222000	3.421997000	0.444625000
C	1.724928000	2.877480000	-0.784818000
C	1.971392000	2.388665000	1.686939000
H	5.341148000	0.935064000	0.012589000
H	4.653852000	1.695255000	-1.432264000
C	2.457486000	-2.763767000	1.053336000
C	4.201165000	-3.353510000	-0.595873000
C	4.715054000	-1.740131000	1.279014000
H	4.017213000	-0.311588000	-2.409611000
H	5.238074000	-1.030443000	-1.362701000
H	-1.438584000	1.827769000	-1.891431000
H	-2.138295000	3.455294000	-2.053414000
H	-3.109986000	2.023838000	-2.444665000
H	-4.887750000	2.897466000	-0.877519000
H	-3.818122000	4.279972000	-0.607880000
H	-4.463826000	3.398703000	0.772121000
H	-2.135850000	2.899910000	1.671360000
H	-1.456992000	3.901128000	0.378611000
H	-0.887223000	2.234450000	0.596604000
H	-2.936783000	-3.806141000	1.133677000
H	-4.308409000	-4.281231000	0.109517000
H	-4.574653000	-3.250238000	1.513262000
H	-5.703351000	-1.525521000	-0.053979000
H	-5.374790000	-2.530127000	-1.462467000
H	-4.785281000	-0.860235000	-1.418671000
H	-2.493155000	-1.861759000	-2.027364000
H	-3.188444000	-3.481512000	-1.896956000
H	-1.755631000	-3.061334000	-0.954728000
H	4.586596000	3.080893000	1.170490000
H	3.447798000	4.378054000	0.794807000
H	4.342361000	3.606339000	-0.509123000
H	2.250167000	2.922587000	-1.744529000
H	1.331606000	3.874458000	-0.565180000
H	0.888534000	2.183435000	-0.873664000
H	1.146260000	1.680493000	1.675554000
H	1.588229000	3.385696000	1.923266000
H	2.677115000	2.095983000	2.470246000
H	2.017497000	-2.015201000	1.713571000
H	2.795976000	-3.602390000	1.667393000
H	1.687011000	-3.131763000	0.372915000
H	3.445749000	-3.664617000	-1.320879000
H	4.456813000	-4.209688000	0.034128000
H	5.104554000	-3.069824000	-1.140806000
H	5.634494000	-1.416393000	0.783711000
H	4.980475000	-2.552544000	1.960773000
H	4.331887000	-0.906702000	1.874860000

3.2 Determination of pK_a values

Similar to Guo and coworkers the pK_a values in DMSO were calculated.[42] Therefore, all structures were preoptimized using the B3LYP functional[18,19] and the 6-31g(d) basis set[22,25–27,38–41] including a SMD solvent model for DMSO. Here we used the SMD solvent model[43] contrary to Guo and coworkers as the Gaussian 16 manual suggests more precise values for ΔG in comparison to the PCM solvent model.[17] The preoptimized structures were reoptimized with the B3LYP functional[18,19] and the 6-311++G(2df,2p) basis set[42] including the SMD solvent model[43] for DMSO. A frequency calculation at the same level of theory was performed of the final structures to obtain ΔG values and confirm the calculated structures are a local minimum.

The free Gibbs energy of the proton exchange with aniline and the chosen base is calculated for the following reaction from the obtained ΔG values (X represents the chosen base):

ΔG: $XH + C_6H_5NH^- \rightarrow X + C_6H_5NH_2$

$\Delta G_{exchange} = G(X) + G(C_6H_5NH_2) - G(XH) – G(C_6H_5NH^-)$

X = PR_2^- for pK_a of secondary phosphine

($HPR_2 + C_6H_5NH^- \rightarrow PR_2^- + C_6H_5NH_2$)

X = HPR_2 for pK_a of protonated secondary phosphine/ pK_{BH^+} of secondary phosphine

($[H_2PR_2]^+ + C_6H_5NH^- \rightarrow HPR_2 + C_6H_5NH_2$)

To calculate the pK_a at standard conditions (T = 298.15 K) the calculated free Gibbs energy and the experimental value of pK_a($C_6H_5NH_2$) = 30.6 is used in the calculation similar to Guo and coworkers.[42,44]

pK_a: $pK_a(XH) = pK_a(C_6H_5NH_2) + \Delta G_{exchange} / (2.303 \cdot RT)$

Table S23: Calculated $\Delta G_{exchange}$ and pK_a values at the B3LYP/6-311++G(2df,2p) level of theory using the SMD solvent model for DMSO, **A**: 1,3-di-*tert*-butyl-2-hydrido-1,3,2-diazaphosphole, **B**: 1,3-di-*tert*-butyl-2-hydrido-1,3,2-diazaphospholidine. *calculated for validation of computations, values in brackets () are extracted from the underlying work.[42]

Compound	$\Delta G_{exchange}$ / kJ mol^{-1}	**pK_a**
**1**	44.43866	38.4
**3**	25.83140	35.1
**A**	43.79139	38.3
**B**	62.48423	41.5
Me_2PH*	20.47500	34.2 (34.8)
Ph_2PH*	-37.41953	24.0 (22.9)
$(Me_2N)_2PH$*	34.48513	36.6 (36.1)
**[1H]$^+$**	-59.98795	20.1
**[3H]$^+$**	-74.85354	17.5
**[AH]$^+$**	-178.53127	-0.672
**[BH]$^+$**	-150.39616	4.26

Table S24: Cartesian coordinates (x y z) of the refined geometry of **aniline** and **its corresponding base** at the B3LYP/6-311++G(2df,2p) level of theory using the SMD solvent model for DMSO.

$C_6H_5NH_2$				$C_6H_5NH^-$			
N	-2.326435	0.000002	-0.073280	C	0.306600	1.186792	0.000037
H	-2.779823	-0.838472	0.258219	C	1.046073	-0.043743	0.000559
H	-2.779818	0.838471	0.258238	C	0.233488	-1.225650	0.000096
C	-0.937426	-0.000002	-0.008364	C	-1.149517	-1.173315	-0.000151
C	-0.220587	1.205341	-0.003833	C	-1.838953	0.045593	-0.000131
C	1.168264	1.199399	0.003224	C	-1.078554	1.219743	-0.000163
C	1.876936	0.000002	0.005837	N	2.387284	-0.135295	-0.000015
C	1.168264	-1.199398	0.003224	H	2.783779	0.802420	-0.000407
C	-0.220587	-1.205344	-0.003832	H	0.735750	-2.187197	0.000049
H	-0.762152	2.143518	-0.005409	H	-1.708711	-2.103119	-0.000283
H	1.700639	2.142243	0.008812	H	-2.920170	0.077665	-0.000281
H	2.958517	-0.000006	0.012160	H	-1.579960	2.182093	-0.000347
H	1.700647	-2.142236	0.008810	H	0.863507	2.118679	-0.000108
H	-0.762151	-2.143521	-0.005404				

Table S25: Cartesian coordinates (x y z) of the refined geometry of **[1H]**$^+$, **1** and its corresponding phosphide (**P(R^1)$_2$**$^-$) at the B3LYP/6-311++G(2df,2p) level of theory using the SMD solvent model for DMSO.

**[1H]**$^+$ ($[H_2PR_2]^+$)				**1** (HPR_2)				**P(R^1)$_2$**$^-$			
N	-3.22067	-0.84704	-0.97487	N	3.241737	1.086008	-0.689441	N	3.316574	-1.201157	0.317503
C	-2.58085	-0.03710	-0.06880	C	2.585194	0.033847	-0.049844	C	2.594620	0.008005	0.065565
C	-2.58495	-1.89995	-1.84444	C	2.596613	2.325047	-1.225150	C	2.707606	-2.528910	0.576212
C	-4.57646	-0.56800	-0.94318	C	4.604458	0.831174	-0.714214	C	4.679073	-0.941178	0.322422
N	-3.55431	0.73024	0.52717	N	3.598663	-0.861388	0.305958	N	3.633273	0.988286	-0.075416
N	-1.29403	-0.09870	0.21912	N	1.315098	0.001237	0.201094	N	1.325858	0.069992	0.022234
C	-1.92606	-2.96080	-0.95508	C	1.951251	3.101287	-0.06908 0	C	1.881086	-2.968516	-0.642789
C	-3.67134	-2.57306	-2.68897	C	3.662755	3.215481	-1.873238	C	3.808903	-3.572490	0.802760
C	-1.57059	-1.24750	-2.79170	C	1.565185	1.954984	-2.300929	C	1.841118	-2.468327	1.846156
C	-4.78017	0.39773	-0.02947	C	4.821524	-0.352536	-0.117470	C	4.871930	0.364430	0.084829
H	-5.29090	-1.06717	-1.56590	H	5.316505	1.497125	-1.158957	H	5.417824	-1.696549	0.505094
C	-3.37852	1.76420	1.60968	C	3.455619	-2.112683	1.117284	C	3.489448	2.396592	-0.531809
P	-0.00003	0.79105	0.00012	P	0.044372	-1.014963	-0.168237	P	0.000003	1.168646	-0.000197
H	-1.14075	-2.53624	-0.33649	H	1.200501	2.50303 0	0.438643	H	1.100831	-2.24812 0	-0.864941
H	-1.48750	-3.73409	-1.58587	H	1.475830	4.004253	-0.452960	H	1.421063	-3.939173	-0.453639
H	-2.66735	-3.43069	-0.30721	H	2.709977	3.399319	0.656624	H	2.525458	-3.064308	-1.519089
H	-4.42393	-3.06541	-2.07385	H	4.417993	3.540926	-1.158144	H	4.460182	-3.676552	-0.065423
H	-3.19327	-3.33558	-3.30174	H	3.169306	4.105931	-2.260660	H	3.334274	-4.537586	0.978243
H	-4.16554	-1.86976	-3.35901	H	4.158390	2.720276	-2.708391	H	4.421993	-3.341764	1.674166
H	-2.04339	-0.45887	-3.37810	H	2.043753	1.405627	-3.113300	H	2.456245	-2.204378	2.708639
H	-1.19238	-2.00222	-3.48131	H	1.134086	2.8662 00	-2.716954	H	1.389830	-3.442902	2.037740
H	-0.72201	-0.82853	-2.25922	H	0.760961	1.346895	-1.898296	H	1.049283	-1.731642	1.747896
H	-5.69840	0.86658	0.26051	H	5.749900	-0.866502	0.029505	H	5.801705	0.894405	0.031984
C	-2.58104	1.17466	2.77913	C	2.679373	-1.814306	2.407704	C	2.761220	2.437489	-1.885042
C	-4.75914	2.17290	2.13669	C	4.846391	-2.624042	1.516358	C	4.875456	3.028149	-0.730418
C	-2.69966	3.00760	1.02237	C	2.774428	-3.203783	0.279143	C	2.755289	3.227300	0.533550
N	1.29399	-0.09868	-0.21892	N	-1.281635	-0.013367	0.062467	N	-1.325868	0.070045	-0.022498
H	0.23131	1.67746	1.07443	H	-0.085129	-1.791662	1.047142	H	3.313993	1.860628	-2.629336
H	-3.08100	0.29051	3.17652	H	3.204974	-1.065486	3.002497	H	2.694354	3.468351	-2.237821
H	-2.52667	1.91846	3.57372	H	2.608935	-2.72717 0	2.99954 0	H	1.749750	2.04044 0	-1.812243
H	-1.56829	0.90172	2.50103	H	1.673365	-1.455798	2.212489	H	5.453876	3.055006	0.193224
H	-5.36275	2.66837	1.37734	H	5.444150	-2.914479	0.652955	H	4.733444	4.057075	-1.059435
H	-4.60963	2.88075	2.95023	H	4.713639	-3.5094 00	2.136574	H	5.458149	2.513486	-1.494458
H	-5.31257	1.32103	2.53107	H	5.400756	-1.888148	2.098529	H	1.758097	2.842109	0.739602
H	-1.68956	2.81009	0.67293	H	1.771254	-2.92338 0	-0.033723	H	2.658097	4.262255	0.200280
H	-2.63574	3.77949	1.78946	H	2.702474	-4.123744	0.860657	H	3.322439	3.223409	1.466402
H	-3.28059	3.40241	0.18848	H	3.360937	-3.412604	-0.616747	C	-2.594634	0.008094	-0.065666
C	2.58084	-0.03703	0.06885	C	-2.567977	-0.025253	0.105024	N	-3.316682	-1.201025	-0.317514
N	3.22076	-0.84705	0.97478	N	-3.304828	1.040856	0.640402	N	-3.633203	0.988453	0.075370
N	3.55423	0.73039	-0.52714	N	-3.538365	-0.952822	-0.314099	C	-2.707804	-2.528835	-0.576158
C	2.58512	-1.90002	1.84432	C	-2.732117	2.292004	1.224178	C	-4.679159	-0.940917	-0.322506
C	4.57654	-0.56796	0.94300	C	-4.659761	0.762214	0.546903	C	-3.489252	2.396703	0.531893
C	3.37836	1.76439	-1.60959	C	-3.332307	-2.223205	-1.080432	C	-4.871909	0.364711	-0.084931
C	4.78015	0.39783	0.02935	C	-4.803382	-0.441816	-0.025665	C	-1.881308	-2.968422	0.642860
C	1.92605	-2.96073	0.95492	C	-1.976322	3.065522	0.134864	C	-3.809166	-3.572363	-0.802634
C	3.67157	-2.57328	2.68865	C	-3.869213	3.179161	1.744756	C	-1.841301	-2.468393	-1.846097
C	1.57089	-1.24762	2.79177	C	-1.814801	1.949233	2.407944	H	-5.417972	-1.696227	-0.505177
H	5.29105	-1.06719	1.56560	H	-5.420219	1.429358	0.900184	C	-2.760799	2.437412	1.885013
C	2.58085	1.17489	-2.77904	C	-2.576405	-1.926489	-2.383042	C	-4.875201	3.02829 0	0.730797
C	4.75896	2.17313	-2.13664	C	-4.694257	-2.82159 0	-1.460170	C	-2.755212	3.227498	-0.533478
C	2.69952	3.00776	-1.02219	C	-2.606769	-3.258455	-0.208922	H	-5.801635	0.894777	-0.032128
H	5.69834	0.86671	-0.26068	H	-5.708123	-0.972422	-0.241314	H	-1.101012	-2.248055	0.864956
H	1.14069	-2.53606	0.33648	H	-1.167989	2.472704	-0.281798	H	-1.421345	-3.939117	0.453756
H	1.48754	-3.73408	1.58567	H	-1.556150	3.979494	0.555912	H	-2.525681	-3.064129	1.519168
H	2.66724	-3.43058	0.30689	H	-2.656120	3.34516 0	-0.672033	H	-4.460553	-3.676205	0.065495
H	4.42405	-3.06561	2.07338	H	-4.549551	3.48517 0	0.950145	H	-3.334606	-4.537535	-0.977890
H	3.19354	-3.33584	3.30139	H	-3.428344	4.081061	2.167591	H	-4.422141	-3.341747	-1.674150
H	4.16589	-1.87008	3.35870	H	-4.444007	2.690287	2.531464	H	-2.456383	-2.204413	-2.708603
H	2.04382	-0.45911	3.37822	H	-2.373289	1.415982	3.17931 0	H	-1.390112	-3.443023	-2.037633
H	1.19270	-2.00241	3.48132	H	-1.429570	2.870597	2.846166	H	-1.049389	-1.731787	-1.747854
H	0.72229	-0.82852	2.25943	H	-0.975265	1.334037	2.098798	H	-3.313505	1.860543	2.629351
H	3.08080	0.29074	-3.17647	H	-3.139922	-1.219414	-2.993976	H	-2.693775	3.468239	2.237864

H 2.52649 1.91871 -3.57361	H -2.460076 -2.849240 -2.952628	H -1.749379 2.040270 1.812017
H 1.56811 0.90196 -2.50095	H -1.585036 -1.519686 -2.202270	H -5.453773 3.055280 -0.192747
H 5.36258 2.66862 -1.37731	H -5.286937 -3.087286 -0.585101	H -4.733097 4.057172 1.059913
H 4.60940 2.88098 -2.95017	H -4.511712 -3.735200 -2.024175	H -5.457788 2.513553 1.494868
H 5.31241 1.32128 -2.53104	H -5.276639 -2.151917 -2.092156	H -1.758059 2.842295 -0.739686
H 1.68946 2.81020 -0.67267	H -1.611599 -2.942079 0.087511	H -2.657951 4.262419 -0.200120
H 2.63550 3.77966 -1.78926	H -2.505249 -4.193454 -0.760758	H -3.322476 3.22371 0 -1.466259
H 3.28051 3.40257 -0.18834	H -3.183895 -3.458350 0.694987	
H -0.23137 1.67752 -1.07414		

Table S26: Cartesian coordinates (x y z) of the refined geometry of **[3H]**$^+$, **3** and its corresponding phosphide (**P(R^3)$_2$**$^-$) at the B3LYP/6-311++G(2df,2p) level of theory using the SMD solvent model for DMSO.

**[3H]**$^+$ ([H_2PR_2]$^+$)	**3** (HPR_2)	**P(R^3)$_2$**$^-$
N -1.31041 -0.12170 0.10526	N -1.264757 0.032846 -0.505323	N -1.304209 0.060772 -0.043676
N 1.31049 0.12181 0.10537	N 1.240678 0.020670 0.209218	N 1.304233 0.060764 0.043538
C -2.56077 0.28097 0.09498	C -2.436821 -0.031364 0.008993	C -2.548561 -0.088411 0.152515
P 0.00004 -0.00028 0.98328	P 0.053513 -1.032827 -0.419224	P 0.000011 -1.075174 -0.000106
C 2.56083 -0.28099 0.09502	C 2.489816 0.014369 -0.108408	C 2.548594 -0.088425 -0.152586
N -2.99358 1.40671 -0.55447	N -3.332564 1.022403 0.009096	N -3.489664 0.976666 0.207004
N -3.61996 -0.38065 0.63455	N -3.006849 -1.130044 0.729149	N -3.254956 -1.309301 0.490508
H -0.17278 1.08302 1.86646	H -0.272615 -1.756213 0.772076	N 3.489714 0.976642 -0.206983
N 2.99363 -1.40667 -0.55446	N 3.314104 1.107985 0.044248	N 3.254998 -1.309322 -0.490531
N 3.62001 0.38075 0.63445	N 3.226337 -1.049695 -0.710754	C -3.144005 2.412922 0.080752
C -2.18204 2.27161 -1.47332	C -3.087606 2.390737 -0.529162	C -4.600223 0.593179 1.083749
C -4.45409 1.35584 -0.69811	C -4.400713 0.792216 0.987583	C -3.782105 -2.193098 -0.609832
C -3.71789 -1.71942 1.29707	C -3.552484 -2.331641 -0.011023	C -4.27465 0 -0.860412 1.456993
C -4.85981 0.36427 0.37900	C -3.956685 -0.500982 1.669483	C 3.144055 2.4129 00 -0.080747
C 2.18208 -2.27159 -1.47330	C 2.911972 2.458653 0.535320	C 4.600355 0.593147 -1.083621
C 4.45415 -1.35576 -0.69818	C 4.588639 0.906073 -0.652361	C 3.782000 -2.193156 0.609849
C 3.71780 1.71943 1.29717	C 3.613432 -2.278277 0.082185	C 4.274803 -0.860439 -1.456903
C 4.85991 -0.36403 0.37878	C 4.342929 -0.390207 -1.41858 0	C -2.395755 2.669134 -1.236969
C -1.72341 1.46500 -2.69727	C -2.566114 2.329825 -1.973261	C -4.449246 3.226052 0.021871
C -3.06798 3.43452 -1.94717	C -4.425137 3.149358 -0.569101	C -2.303529 2.901708 1.275436
C -0.98123 2.88987 -0.74267	C -2.102273 3.14931 0 0.373679	H -5.561828 0.687385 0.575005
H -4.72575 1.00224 -1.69652	H -5.374720 0.704422 0.503107	H -4.639517 1.212884 1.982452
H -4.90928 2.32702 -0.53810	H -4.452735 1.606701 1.708158	C -4.127556 -3.551695 0.018513
C -4.78078 -1.62543 2.40562	C -3.727163 -3.457751 1.017933	C -5.031488 -1.617961 -1.308991
C -4.14947 -2.75391 0.24435	C -4.902610 -2.028368 -0.691145	C -2.702957 -2.420544 -1.668534
C -2.41555 -2.17969 1.95387	C -2.582575 -2.823905 -1.085965	H -3.850228 -0.900023 2.462101
H -5.18654 0.87201 1.28790	H -3.434201 -0.275684 2.600666	H -5.154006 -1.4959 00 1.449601
H -5.65784 -0.29318 0.04599	H -4.789621 -1.152148 1.907215	C 4.449293 3.226017 -0.021631
C 3.06799 -3.43451 -1.94717	C 4.177334 3.298042 0.77848 0	C 2.303775 2.901731 -1.27555 0
C 0.98127 -2.88982 -0.74264	C 2.036806 3.162205 -0.513444	C 2.395595 2.669077 1.236862
C 1.72344 -1.46495 -2.69723	C 2.178812 2.356763 1.882155	H 5.561911 0.687341 -0.574784
H 4.72571 -1.00225 -1.69665	H 5.421443 0.83363 0 0.04988 0	H 4.639742 1.212855 -1.982318
H 4.90938 -2.32690 -0.53809	H 4.793164 1.724574 -1.339099	C 2.702713 -2.420606 1.66841 0
C 2.41538 2.17945 1.95399	C 2.436182 -2.833885 0.883055	C 4.127501 -3.551744 -0.018487
C 4.78070 1.62546 2.40571	C 4.044827 -3.355579 -0.923627	C 5.031308 -1.61806 0 1.309178
C 4.14929 2.75411 0.24458	C 4.76532 0 -1.982191 1.063121	H 3.850487 -0.900039 -2.462056
H 5.18685 -0.87167 1.28767	H 4.032981 -0.171785 -2.441963	H 5.154149 -1.495938 -1.449422
H 5.65781 0.29350 0.04560	H 5.226052 -1.01586 0 -1.466306	H -1.445159 2.146673 -1.266576
H -1.06959 0.64437 -2.40970	H -1.593382 1.854606 -2.036212	H -2.21224 0 3.738926 -1.349151
H -1.17509 2.11285 -3.38253	H -2.487047 3.344676 -2.365056	H -2.997158 2.340214 -2.086997
H -2.58012 1.05599 -3.23538	H -3.261555 1.777869 -2.608436	H -5.093305 2.877581 -0.787187
H -3.92616 3.09878 -2.52675	H -5.166163 2.61576 0 -1.165652	H -4.210113 4.272996 -0.167037
H -2.46943 4.07834 -2.58993	H -4.260404 4.121698 -1.032705	H -5.012548 3.181973 0.953148
H -3.42395 4.03506 -1.10940	H -4.839366 3.325482 0.422138	H -2.832127 2.736039 2.216315
H -1.28842 3.32999 0.20647	H -2.483831 3.211477 1.394484	H -2.103903 3.971769 1.190695
H -0.56327 3.68449 -1.36170	H -1.958851 4.166999 0.0066 00	H -1.34999 0 2.378994 1.320312
H -0.18749 2.17327 -0.56258	H -1.131878 2.657300 0.397575	H -3.246444 -3.987285 0.492070
H -4.51117 -0.86837 3.14321	H -2.773525 -3.692779 1.492336	H -4.479961 -4.241905 -0.749690
H -4.84145 -2.58664 2.91450	H -4.09327 0 -4.357708 0.522165	H -4.912324 -3.471669 0.771056
H -5.77214 -1.39635 2.01948	H -4.441881 -3.200979 1.799249	H -5.868926 -1.494595 -0.622348
H -5.08714 -2.47117 -0.23487	H -5.671537 -1.738890 0.024009	H -5.356904 -2.298739 -2.097625
H -4.29602 -3.72595 0.71703	H -5.259298 -2.920545 -1.207822	H -4.818302 -0.653316 -1.769491
H -3.38714 -2.86390 -0.52733	H -4.802770 -1.23446 0 -1.432012	H -2.42075 0 -1.496246 -2.171118
H -1.63881 -2.40110 1.22947	H -2.406615 -2.080927 -1.861477	H -3.08376 0 -3.108564 -2.424846
H -2.62882 -3.09637 2.50423	H -3.016437 -3.702445 -1.564565	H -1.80453 0 -2.853612 -1.234194
H -2.04361 -1.44613 2.66863	H -1.626038 -3.122204 -0.661587	H 5.093223 2.877511 0.787515
H 3.92619 -3.09880 -2.52673	H 4.852581 2.808499 1.481042	H 4.21014 0 4.272956 0.167278
H 2.46942 -4.07829 -2.58995	H 3.880263 4.252558 1.212094	H 5.012742 3.181969 -0.952822
H 3.42391 -4.03509 -1.10940	H 4.724107 3.511897 -0.138197	H 2.832521 2.736085 -2.216350
H 1.28844 -3.32991 0.20652	H 2.572531 3.255723 -1.459922	H 2.104147 3.971792 -1.190808
H 0.56330 -3.68445 -1.36165	H 1.774644 4.165981 -0.174914	H 1.350237 2.379029 -1.320593
H 0.18751 -2.17323 -0.56258	H 1.115719 2.611239 -0.691457	H 1.444992 2.146618 1.266301
H 1.06965 -0.64431 -2.40964	H 1.249214 1.804821 1.79733 0	H 2.212065 3.738866 1.349045
H 1.17509 -2.11278 -3.38248	H 1.960583 3.361569 2.246007	H 2.996861 2.340130 2.086977
H 2.58016 -1.05596 -3.23535	H 2.808791 1.860665 2.623025	H 2.420466 -1.496314 2.170984
H 1.63858 2.40067 1.22960	H 2.067755 -2.130799 1.627003	H 3.083404 -3.108656 2.424750
H 2.62850 3.09617 2.50434	H 2.770339 -3.728932 1.408729	H 1.804330 -2.853638 1.233944
H 2.04361 1.44581 2.66875	H 1.612911 -3.122786 0.231859	H 3.246438 -3.987305 -0.492163
H 4.51127 0.86818 3.14315	H 3.235114 -3.572482 -1.621516	H 4.479797 -4.241981 0.749742
H 4.84115 2.58658 2.91479	H 4.293679 -4.275134 -0.392586	H 4.912363 -3.471716 -0.770931

H 5.77211 1.39670 2.01953	H 4.922623 -3.063661 -1.499058	H 5.868833 -1.494694 0.622642
H 5.08699 2.47151 -0.23467	H 5.666414 -1.643416 0.553121	H 5.356612 -2.298863 2.097836
H 4.29575 3.72610 0.71739	H 5.023525 -2.889891 1.610471	H 4.818083 -0.653422 1.769674
H 3.38695 2.86413 -0.52709	H 4.476643 -1.225574 1.793657	
H 0.17284 -1.08411 1.86579		

Table S27: Cartesian coordinates (x y z) of the refined geometry of **[AH]**$^{+}$, NHP **A** and its corresponding phosphide (**P(R)$_2$$^{-}$**) at the B3LYP/6-311++G(2df,2p) level of theory using the SMD solvent model for DMSO.

**[AH]**$^{+}$ ([H_2PR_2]$^{+}$)	**A** (HPR_2)	**P(R)$_2$**$^{-}$
P -0.00000 0.87164 -0.05803	P 0.996348 -0.078536 0.000000	P 0.000000 -1.115122 -0.981265
N -1.22611 -0.23352 -0.13720	N -0.199796 0.199795 1.221716	N 1.319686 0.127532 -0.729103
N 1.22611 -0.23351 -0.13719	N -0.199796 0.199795 -1.221716	N -1.319685 0.127532 -0.729102
C -2.68871 0.05796 0.01739	C 0.056355 0.051274 2.677915	C 2.431185 -0.036022 0.260010
C 2.68871 0.05797 0.01739	C 0.056355 0.051274 -2.677915	C -2.431185 -0.036022 0.260010
C -0.66628 -1.53916 -0.05897	C -1.467465 -0.080372 0.670685	C 0.672306 1.377296 -0.781482
C 0.66628 -1.53915 -0.05896	C -1.467465 -0.080372 -0.670685	C -0.672305 1.377296 -0.781482
C -2.91447 1.53416 -0.31845	C 1.486665 0.509230 2.981063	C 3.105443 -1.384284 -0.017014
C -3.10853 -0.23403 1.46162	C -0.118924 -1.413120 3.113951	C 1.940024 0.006881 1.716546
C -3.47048 -0.81401 -0.96863	C -0.922286 0.948489 3.448127	C 3.468688 1.077140 0.04378 0
C 2.91452 1.53410 -0.31871	C 1.486665 0.50923 0 -2.981063	C -3.105443 -1.384284 -0.017014
C 3.47049 -0.81421 -0.96845	C -0.922286 0.948489 -3.448127	C -3.468689 1.077139 0.043779
C 3.10847 -0.23379 1.46168	C -0.118924 -1.41312 0 -3.113951	C -1.940024 0.006881 1.716546
H -0.00001 1.66425 1.10880	H 0.981316 -1.550899 0.000000	H -1.226421 -0.795213 1.908937
H 2.55418 0.39232 2.16221	H 0.568717 -2.067615 -2.576415	H -1.453904 0.959106 1.935699
H 2.93099 -1.27845 1.71831	H -1.135474 -1.760761 -2.926083	H -2.774004 -0.110570 2.411833
H 4.17196 -0.03035 1.58880	H 0.08022 0 -1.517413 -4.181403	H -3.931965 -1.550034 0.676075
H 3.97070 1.77218 -0.20274	H 1.659999 0.470288 -4.056402	H -3.500181 -1.417662 -1.034374
H 2.62996 1.75620 -1.34757	H 1.652927 1.533516 -2.645316	H -2.396725 -2.205595 0.100787
H 2.36635 2.19740 0.35625	H 2.230401 -0.133189 -2.506181	H -3.089501 2.058114 0.330302
H 3.36271 -1.87577 -0.74919	H -1.958746 0.649066 -3.294697	H -3.780181 1.120509 -1.001587
H 3.14071 -0.63471 -1.99207	H -0.816144 1.989408 -3.139286	H -4.353125 0.880918 0.652042
H 4.53022 -0.56899 -0.90081	H -0.717213 0.885177 -4.517280	H -1.258206 2.285055 -0.847545
H 1.31706 -2.39422 -0.03664	H -2.335449 -0.175578 -1.300812	H 1.258206 2.285054 -0.847546
H -1.31706 -2.39423 -0.03664	H -2.335449 -0.175578 1.300812	H 1.226421 -0.795214 1.908936
H -2.55424 0.39194 2.16227	H 0.568717 -2.067615 2.576415	H 2.774004 -0.11057 0 2.411833
H -4.17201 -0.03060 1.58874	H 0.080220 -1.517413 4.181403	H 1.453903 0.959106 1.935699
H -2.93106 -1.27875 1.71806	H -1.135474 -1.760761 2.926083	H 3.780181 1.120509 -1.001586
H -3.14066 -0.63436 -1.99221	H -0.816144 1.989408 3.139286	H 3.0895 00 2.058114 0.330303
H -3.36276 -1.87562 -0.74955	H -1.958746 0.649066 3.294697	H 4.353124 0.880918 0.652043
H -4.53020 -0.56876 -0.90099	H -0.717213 0.885177 4.517280	H 3.500182 -1.417662 -1.034374
H -2.62986 1.75644 -1.34726	H 1.652927 1.533516 2.645316	H 3.931965 -1.550034 0.676076
H -3.97065 1.77225 -0.20248	H 1.659999 0.470288 4.056402	H 2.396726 -2.205595 0.100787
H -2.36630 2.19732 0.35665	H 2.230401 -0.133189 2.506181	
H -0.00001 1.80002 -1.10607		

Table S28: Cartesian coordinates (x y z) of the refined geometry **[BH]**$^{+}$, NHP **B** and its corresponding phosphide (**P(R)$_2$$^{-}$**) at the B3LYP/6-311++G(2df,2p) level of theory using the SMD solvent model for DMSO.

**[BH]**$^{+}$ ([H_2PR_2]$^{+}$)	**B** (HPR_2)	**P(R)$_2$**$^{-}$
P -0.00000 0.82254 0.00000	P -0.005950 -0.956482 0.207789	P -0. 003000 -0.994817 0.375531
N -1.23576 -0.24891 0.16391	N 1.222272 0.169439 -0.188389	N 1.225707 0.169057 -0.252112
N 1.23576 -0.24891 -0.16390	N -1.219819 0.198982 -0.194552	N -1.225704 0.169085 -0.252072
C -2.68050 0.11735 0.00806	C 2.670356 -0.117652 -0.074250	C 2.671255 -0.111021 -0.100365
C 2.68050 0.11735 -0.00806	C -2.671185 -0.110741 -0.091903	C -2.671259 -0.111014 -0.100377
C -0.73974 -1.60217 -0.18380	C 0.755905 1.560718 -0.042062	C 0.774497 1.502956 0.155504
C 0.73974 -1.60217 0.18380	C -0.742278 1.502634 0.287803	C -0.774481 1.502898 0.155773
C -2.88009 1.52485 0.57963	C 2.941134 -1.560877 -0.518008	C 2.977325 -1.479370 -0.726398
C -3.52255 -0.87621 0.81366	C 3.172087 0.065887 1.371331	C 3.153276 -0.122046 1.367782
C -3.08371 0.08714 -1.47181	C 3.440888 0.829316 -1.007406	C 3.476547 0.951434 -0.870163
C 2.88014 1.52479 -0.57978	C -2.927763 -1.495551 -0.699655	C -2.977295 -1.47933 0 -0.726499
C 3.52255 -0.87631 -0.81353	C -3.456358 0.925761 -0.910511	C -3.476543 0.951472 -0.870141
C 3.08367 0.08729 1.47182	C -3.170397 -0.095880 1.364960	C -3.153322 -0.122124 1.367753
H 2.48928 0.79255 2.05498	H -2.645668 -0.836893 1.969880	H -2.646072 -0.897915 1.942331
H 2.95735 -0.90702 1.90000	H -3.032557 0.881635 1.828043	H -2.971109 0.833289 1.861478
H 4.13337 0.36357 1.57569	H -4.235845 -0.328805 1.400226	H -4.227227 -0.314571 1.413328
H 3.93229 1.79714 -0.50830	H -4. 030000 -1.691296 -0.724760	H -4.051304 -1.67185 0 -0.703187
H 2.58725 1.57197 -1.62885	H -2.546129 -1.551677 -1.720208	H -2.646118 -1.512305 -1.765930
H 2.32152 2.28111 -0.02157	H -2.46084 0 -2.290844 -0.117700	H -2.479420 -2.288048 -0.191280
H 3.46177 -1.88588 -0.40884	H -3.376931 1.928288 -0.491268	H -3.381729 1.941919 -0.425126
H 3.20633 -0.89949 -1.85696	H -3.099939 0.954947 -1.941419	H -3.145861 1.011667 -1.908913
H 4.56801 -0.57038 -0.77930	H -4.514018 0.660345 -0.922484	H -4.536237 0.691076 -0.865159
H 1.27710 -2.34814 -0.39488	H -1.273440 2.318849 -0.198563	H -1.156679 2.260839 -0.531939
H -0.87511 -1.81017 -1.24705	H 0.924563 2.104657 -0.974278	H 1.156406 2.260705 -0.532583
H -3.20629 -0.89930 1.85708	H 2.664121 -0.621151 2.049931	H 2.646039 -0.897835 1.942376
H -4.56800 -0.57025 0.77943	H 4.243773 -0.131585 1.432253	H 4.227188 -0.314445 1.413401
H -3.46183 -1.88582 0.40905	H 3.003815 1.082762 1.727990	H 2.971004 0.833379 1.861463
H -2.48933 0.79233 -2.05506	H 3.092968 0.728171 -2.036801	H 3.145907 1.011557 -1.908952
H -2.95740 -0.90722 -1.89989	H 3.332654 1.872366 -0.710448	H 3.381689 1.941906 -0.425212
H -4.13340 0.36341 -1.57568	H 4.504294 0.589042 -0.981580	H 4.536247 0.691063 -0.865122
H -2.32148 2.28110 0.02132	H 2.572828 -1.736566 -1.529702	H 2.646188 -1.512406 -1.765840

H -3.93225 1.79722 0.50815	H 4.014638 -1.751693 -0.506965	H 4.051335 -1.671883 -0.703034
H -2.58717 1.57213 1.62870	H 2.471751 -2.287858 0.14643 0	H 2.479436 -2.288061 -0.191153
H -1.27709 -2.34815 0.39489	H 1.28668 0 2.092777 0.75176 0	H 1.124317 1.772233 1.163837
H 0.87511 -1.81016 1.24706	H -0.887843 1.603485 1.370683	H -1.123974 1.771816 1.164317
H 0.06438 1.68236 1.10518	H -0.041085 -0.842354 1.660945	
H -0.06439 1.68236 -1.10518		

Table S29: Cartesian coordinates (x y z) of the refined geometry of **dimethylphosphine** and its corresponding phosphide (**PMe$_2$**$^-$) at the B3LYP/6-311++G(2df,2p) level of theory using the SMD solvent model for DMSO.

**HPMe$_2$** (HPR$_2$)	**PMe$_2$**$^-$ (PR$_2$$^-$)
C 1.430780 0.522624 0.024159	C -1.425620 -0.516968 0.000000
P 0. 004000 -0.645368 -0.114711	P 0.000000 0.720413 0.000000
H 2.354135 -0.047444 0.130683	H -2.372444 0.029115 0. 056
H 1.333763 1.204885 0.868640	H -1.424735 -1.165238 0.882724
H 1.499499 1.104684 -0.895541	H -1.424797 -1.165163 -0.882779
C -1.430774 0.522633 0.024145	C 1.425620 -0.516968 0.000000
H -1.333522 1.205184 0.868368	H 1.424818 -1.165137 0.882798
H -2.35404 0 -0.047482 0.131209	H 2.372444 0.029115 -0. 094000
H -1.499872 1.104368 -0.895726	H 1.424714 -1.165264 -0.882705
H -0. 057000 -1.115226 1.223211	

Table S30: Cartesian coordinates (x y z) of the refined geometry of **diphenylphosphine** and its corresponding phosphide (**PPh$_2$**$^-$) at the B3LYP/6-311++G(2df,2p) level of theory using the SMD solvent model for DMSO.

**HPPh$_2$** (HPR$_2$)	**PPh$_2$**$^-$ (PR$_2$$^-$)
C -1.438148 -0.142827 -0.537735	C -1.467932 0.474687 -0.015725
P -0. 728000 -0.435026 -1.657637	P 0. 001000 1.566583 -0. 007000
C 1.441731 -0.176195 -0.535191	C 1.467932 0.474684 0.015722
C -1.894971 1.167083 -0.353859	C -2.680828 1.011569 0.469256
C -2.114219 -1.185557 0.102362	C -1.539277 -0.821052 -0.56896 0
C 1.949051 -1.190743 0.283536	C 1.539273 -0.821058 0.56895 0
C 2.064565 1.075472 -0.519504	C 2.680833 1.01157 0 -0.469246
C -2.987155 1.429575 0.465108	C -3.877349 0.31004 0 0.40313 0
C -3.65289 0 0.383597 1.098248	C -3.916988 -0.975375 -0.135932
C -3.217115 -0.923921 0.910312	C -2.733219 -1.530597 -0.619507
H -1.397386 1.988139 -0.855191	H -2.678302 2.002244 0.910332
H -1.778923 -2.206369 -0.027075	H -0.64999 0 -1.277561 -0.981991
H -3.731025 -1.742762 1.397142	H -2.740679 -2.524345 -1.051921
H -4.507958 0.586829 1.729472	H -4.845253 -1.529817 -0.177552
H -3.323989 2.449155 0.600996	H -4.782921 0.764522 0.787102
C 3.155587 1.315043 0.310271	C 3.877352 0.310041 -0.403115
C 3.048242 -0.955311 1.10256 0	C 2.733215 -1.530602 0.619502
C 3.650326 0.299703 1.122273	C 3.916987 -0.975377 0.135939
H 4.503561 0.482379 1.762361	H 4.845253 -1.529819 0.177563
H 3.623091 2.291134 0.314691	H 4.782927 0.764525 -0.787077
H 3.432498 -1.751276 1.727426	H 2.740671 -2.524353 1.051909
H 1.484545 -2.168253 0.282369	H 0.649983 -1.277571 0.981970
H 1.700165 1.867656 -1.161674	H 2.678310 2.002249 -0.910314
H -0.023685 -1.846749 -1.596242	

Table S31: Cartesian coordinates (x y z) of the refined geometry of **bis(dimethylamino)phosphine** and its corresponding phosphide (**P(NMe$_2$)$_2$**$^-$) at the B3LYP/6-311++G(2df,2p) level of theory using the SMD solvent model for DMSO.

**HP(NMe$_2$)$_2$** (HPR$_2$)	**P(NMe$_2$)$_2$**$^-$ (PR$_2$$^-$)
N 1.437164 -0.007764 0.156589	N -1.393027 0. 003000 -0.209333
P 0.000000 -0.356025 1.018367	P 0. 156000 -0. 016000 0.891795
N -1.437168 -0.007763 0.156592	N 1.392438 0. 004000 -0.212186
H 0.000001 0.854282 1.750848	C -2.219062 -1.199342 -0.281308
C 1.766976 1.318265 -0.349951	C -2.219055 1.199355 -0.281272
C 2.054399 -1.084841 -0.607761	C 2.219110 1.199287 -0.280287
C -2.054399 -1.084840 -0.607762	C 2.219107 -1.199279 -0.280339
C -1.766973 1.318266 -0.349952	H -1.593067 -2.088044 -0.331644
H 1.360278 2.084872 0.307334	H -2.852131 -1.179267 -1.176856
H 1.379725 1.490652 -1.361826	H -2.894542 -1.314739 0.583657
H 2.853218 1.441750 -0.387169	H -1.593055 2.088055 -0.331586
H 1.809545 -2.048773 -0.162653	H -2.894531 1.314732 0.583698
H 3.142128 -0.972543 -0.60405 0	H -2.852128 1.179309 -1.176819
H 1.725418 -1.099120 -1.654655	H 1.593499 2.088059 -0.334541
H -1.809551 -2.048771 -0.162651	H 2.856944 1.178527 -1.172387
H -1.725411 -1.099120 -1.654654	H 2.889884 1.315195 0.588271
H -3.142128 -0.972540 -0.604059	H 1.593493 -2.088048 -0.334612
H -1.360280 2.084872 0.307336	H 2.889893 -1.315217 0.588207
H -2.853215 1.441752 -0.387180	H 2.856928 -1.178491 -1.172447
H -1.379714 1.49065 0 -1.361825	

3.3 Determination of Hydride Ion Affinities

Similar to Riedel and coworkers the hydride anion affinity (HIA) was calculated.[45] All structures were optimized using the B3LYP functional[18,19] and the def2-TZVPP basis set[25–27,46] along with Grimme's dispersion correction with Becke-Johnson damping[47]. A frequency calculation at the same level of theory was performed of the final structure to obtain ΔH values and confirm the calculated structure is a local minimum.

To determine the HIA the negative reaction enthalpy of the hydride exchange with silane and the chosen compound is calculated:

ΔH: $X^+ + Me_3Si\text{-}H \rightarrow X\text{-}H + Me_3Si^+$

$\Delta H = H(X\text{-}H) + H(Me_3Si^+) - H(X^+) - H(Me_3Si\text{-}H)$

$HIA = -\Delta H + HIA(Me_3SiH)$ with $HIA(Me_3SiH) = 959\ kJ\ mol^{-1}$[48]

Table S32: Calculated ΔH and HIA values at the B3LYP/def2-TZVPP level of theory, **A**: 1,3-di-*tert*-butyl-2-hydrido-1,3,2-diazaphosphole, **B**: 1,3-di-*tert*-butyl-2-hydrido-1,3,2-diazaphospholidine.

Compound	ΔH / kJ mol^{-1}	**HIA** / kJ mol^{-1}
**1**	351.63040	607
**3**	363.24038	596
**A**	287.94854	671
**B**	228.36445	731

Table S33: Cartesian coordinates (x y z) of the refined geometry of **Me$_3$SiH** and **its corresponding base** at the B3LYP/def2-TZVPP level of theory.

Me$_3$Si**H** (XH)	Me$_3$Si$^+$ (X$^+$)
Si -1.463474000 0.435989000 -0.095773000	Si 0.000005000 0.000009000 -0.000020000
C 0.414461000 0.446372000 -0.118964000	C -0.492321000 -1.759190000 -0.000052000
H -1.959633000 1.010672000 -1.375998000	C -1.277359000 1.305946000 0.000174000
C -2.101421000 1.487655000 1.323467000	C 1.769676000 0.453236000 -0.000109000
C -2.101444000 -1.323389000 0.061587000	H -2.291285000 0.910653000 0.005835000
H -1.752725000 2.518387000 1.240030000	H -1.143113000 1.946399000 -0.878286000
H -1.757007000 1.095991000 2.282931000	H -1.135812000 1.954866000 0.871084000
H -3.192401000 1.505469000 1.343364000	H 2.257786000 0.014695000 0.876986000
H -1.752658000 -1.946033000 -0.764011000	H 1.934348000 1.528968000 -0.003530000
H -3.192428000 -1.350123000 0.061421000	H 2.260268000 0.008177000 -0.872402000
H -1.757048000 -1.779997000 0.991905000	H -1.121839000 -1.961633000 -0.873230000
H 0.803171000 1.460546000 -0.225242000	H -1.117370000 -1.962542000 0.876167000
H 0.803217000 -0.148115000 -0.947464000	H 0.356975000 -2.439649000 -0.002426000
H 0.816971000 0.030608000 0.807177000	

Table S34: Cartesian coordinates (x y z) of the refined geometry of **1** and its corresponding base (**P(R^1)$_2$$^+$**) at the B3LYP/def2-TZVPP level of theory.

**1** (XH)	**P(R^1)$_2$$^+$** (X$^+$)
N 3.199520000 1.151484000 -0.559430000	N 3.358396000 1.145866000 0.114465000
C 2.563385000 0.010886000 -0.061540000	C 2.574611000 0.030366000 0.019431000
C 2.496241000 2.403453000 -0.927256000	C 2.876644000 2.545172000 0.362560000
C 4.568602000 0.939984000 -0.611588000	C 4.688139000 0.760622000 0.005782000
N 3.605472000 -0.896181000 0.173029000	N 3.411475000 -1.039914000 -0.129918000
N 1.307390000 -0.075923000 0.170297000	N 1.259657000 -0.003224000 0.161788000
C 1.801579000 2.968811000 0.317156000	C 2.050142000 2.571226000 1.652104000

C 3.512116000 3.427264000 -1.436734000	C 4.088849000 3.461875000 0.532002000
C 1.490272000 2.111242000 -2.047972000	C 2.064141000 3.025701000 -0.844262000
C 4.816972000 -0.304269000 -0.171034000	C 4.720941000 -0.575379000 -0.142526000
H 5.258651000 1.676753000 -0.968550000	H 5.497836000 1.459478000 0.039710000
C 3.465715000 -2.216098000 0.835041000	C 3.005072000 -2.480588000 -0.229200000
P 0.032946000 -1.080481000 -0.184185000	P -0.000114000 -0.000373000 -0.800290000
H 1.093572000 2.250407000 0.716572000	H 1.188701000 1.913300000 1.595881000
H 1.271094000 3.885746000 0.060676000	H 1.701758000 3.587535000 1.831458000
H 2.540767000 3.201152000 1.085021000	H 2.661475000 2.266621000 2.501267000
H 4.245896000 3.686780000 -0.673631000	H 4.714482000 3.157375000 1.369890000
H 2.976995000 4.336822000 -1.705034000	H 3.727817000 4.467175000 0.738726000
H 4.034436000 3.073648000 -2.325879000	H 4.696287000 3.510665000 -0.371124000
H 2.007578000 1.716003000 -2.922925000	H 2.646547000 2.938718000 -1.760944000
H 0.981677000 3.031996000 -2.334985000	H 1.799784000 4.073336000 -0.706132000
H 0.748390000 1.388548000 -1.726074000	H 1.143084000 2.464350000 -0.966785000
H 5.755898000 -0.811205000 -0.087671000	H 5.563770000 -1.224768000 -0.256294000
C 2.730246000 -2.040610000 2.170053000	C 2.126067000 -2.847150000 0.970780000
C 4.855183000 -2.789005000 1.131200000	C 4.261882000 -3.352138000 -0.198128000
C 2.740613000 -3.190790000 -0.101378000	C 2.282399000 -2.709179000 -1.560406000
N -1.276657000 -0.088087000 0.085956000	N -1.259629000 0.003687000 0.162194000
H -0.051068000 -1.855915000 1.031056000	H 2.670733000 -2.694292000 1.902148000
H 3.308360000 -1.393031000 2.830337000	H 1.856963000 -3.900382000 0.903578000
H 2.616461000 -3.011158000 2.653257000	H 1.215273000 -2.259600000 1.008260000
H 1.747242000 -1.602578000 2.036305000	H 4.907403000 -3.174029000 -1.057234000
H 5.424252000 -2.972849000 0.220069000	H 3.952750000 -4.394698000 -0.233306000
H 4.729349000 -3.743985000 1.638889000	H 4.833838000 -3.206829000 0.717381000
H 5.432049000 -2.135361000 1.784846000	H 1.353503000 -2.149717000 -1.625750000
H 1.753279000 -2.836041000 -0.386204000	H 2.044626000 -3.766866000 -1.667307000
H 2.630814000 -4.161181000 0.384404000	H 2.916304000 -2.415683000 -2.396609000
H 3.319876000 -3.326843000 -1.015401000	C -2.574593000 -0.030269000 0.019634000
C -2.549476000 -0.031304000 0.120981000	N -3.358272000 -1.145845000 0.114532000
N -3.239054000 1.117600000 0.535850000	N -3.411541000 1.039940000 -0.129723000
N -3.567123000 -0.953415000 -0.185344000	C -2.876466000 -2.545139000 0.362608000
C -2.582051000 2.384765000 0.933554000	C -4.688047000 -0.760715000 0.005736000
C -4.605523000 0.894715000 0.476935000	C -3.005239000 2.480639000 -0.229198000
C -3.391011000 -2.289215000 -0.805492000	C -4.720960000 0.575282000 -0.142551000
C -4.807478000 -0.358175000 0.043337000	C -2.049719000 -2.571139000 1.651993000
C -1.826675000 2.950874000 -0.274161000	C -4.088624000 -3.461857000 0.532312000
C -3.645804000 3.393961000 1.370026000	C -2.064195000 -3.025701000 -0.844355000
C -1.634682000 2.130663000 2.113964000	H -5.497685000 -1.459643000 0.039560000
H -5.329028000 1.631277000 0.760456000	C -2.126215000 2.847442000 0.970694000
C -2.637059000 -2.146381000 -2.133586000	C -4.262123000 3.352073000 -0.198226000
C -4.764732000 -2.898647000 -1.106778000	C -2.282569000 2.709109000 -1.560432000
C -2.670697000 -3.226976000 0.171322000	H -5.563844000 1.224589000 -0.256378000
H -5.733709000 -0.872013000 -0.106968000	H -1.188286000 -1.913220000 1.595594000
H -1.079816000 2.243520000 -0.616514000	H -1.701299000 -3.587442000 1.831314000
H -1.331310000 3.882876000 -0.001369000	H -2.660881000 -2.266500000 2.501263000
H -2.521765000 3.154999000 -1.089756000	H -4.714153000 -3.157279000 1.370248000
H -4.333683000 3.637957000 0.560390000	H -3.727531000 -4.467123000 0.739100000
H -3.144287000 4.313764000 1.666958000	H -4.696178000 -3.510770000 -0.370727000
H -4.218137000 3.035018000 2.225602000	H -2.646793000 -2.938801000 -1.760920000
H -2.193632000 1.741078000 2.965866000	H -1.799735000 -4.073312000 -0.706222000
H -1.163876000 3.068098000 2.411913000	H -1.143194000 -2.464303000 -0.967128000
H -0.864149000 1.416103000 1.843764000	H -2.670807000 2.694678000 1.902119000
H -3.172268000 -1.461670000 -2.792543000	H -1.857225000 3.900694000 0.903305000
H -2.575061000 -3.118404000 -2.624479000	H -1.215366000 2.259978000 1.008190000
H -1.624036000 -1.780168000 -1.991045000	H -4.907620000 3.173835000 -1.057321000
H -5.356742000 -3.039721000 -0.202677000	H -3.953090000 4.394660000 -0.233488000
H -4.609922000 -3.878018000 -1.556406000	H -4.834070000 3.206784000 0.717293000
H -5.332116000 -2.293431000 -1.813033000	H -1.353644000 2.149686000 -1.625680000
H -1.692576000 -2.857366000 0.456181000	H -2.044831000 3.766796000 -1.667432000
H -2.539926000 -4.208440000 -0.285455000	H -2.916433000 2.415508000 -2.396625000
H -3.264964000 -3.345167000 1.078011000	

Table S35: Cartesian coordinates (x y z) of the refined geometry of **3** and its corresponding base (**$P(R^3)_2^+$**) at the B3LYP/def2-TZVPP level of theory.

**3** (XH)	**$P(R^3)_2^+$** (X^+)
N -1.198232000 0.063941000 -0.475114000	N -1.263511000 -0.013997000 0.013138000
N 1.339623000 -0.144270000 0.027694000	N 1.263613000 0.014613000 0.012665000
C -2.346094000 -0.120655000 0.034114000	C -2.572190000 0.006805000 -0.020814000
P 0.099326000 -1.034055000 -0.639578000	P -0.000096000 0.000118000 -0.935624000
C 2.584638000 0.013572000 -0.186282000	C 2.572247000 -0.006659000 -0.020907000
N -3.265062000 0.896777000 0.276460000	N -3.361936000 -1.100981000 -0.100610000
N -2.890616000 -1.349858000 0.528634000	N -3.326811000 1.130274000 0.101030000
H -0.241823000 -1.902934000 0.443899000	N 3.362376000 1.100849000 -0.100451000
N 3.170591000 1.268205000 -0.345266000	N 3.326480000 -1.130403000 0.101061000
N 3.596856000 -0.949543000 -0.324194000	C -2.902158000 -2.502075000 0.169039000
C -2.986523000 2.333131000 0.048942000	C -4.744936000 -0.703446000 0.191308000
C -4.230121000 0.478427000 1.288738000	C -2.834432000 2.534671000 -0.019241000
C -3.508684000 -2.299271000 -0.455023000	C -4.731024000 0.780669000 -0.132660000
C -3.767354000 -0.942693000 1.634376000	C 2.902930000 2.502105000 0.168925000
C 2.450454000 2.540624000 -0.097118000	C 4.745193000 0.702881000 0.191680000
C 4.613005000 1.166872000 -0.213015000	C 2.833636000 -2.534629000 -0.019399000
C 3.706603000 -2.230182000 0.419455000	C 4.730871000 -0.781249000 -0.132261000

C 4.850737000 -0.266532000 -0.656334000	C -2.308102000 -2.589896000 1.579273000
C -2.574462000 2.558023000 -1.411551000	C -4.116874000 -3.430914000 0.065842000
C -4.274925000 3.130525000 0.288542000	C -1.889072000 -2.954321000 -0.890441000
C -1.891474000 2.811060000 1.011621000	H -4.977055000 -0.872615000 1.246942000
H -5.252514000 0.511388000 0.905717000	H -5.455926000 -1.251086000 -0.417041000
H -4.183341000 1.118484000 2.170610000	C -4.003259000 3.481028000 0.275887000
C -3.665767000 -3.649234000 0.251043000	C -1.746377000 2.804626000 1.026624000
C -4.874315000 -1.796065000 -0.959247000	C -2.334739000 2.797234000 -1.445830000
C -2.600113000 -2.495206000 -1.666722000	H -5.004128000 0.964368000 -1.176156000
H -3.185860000 -0.935255000 2.557653000	H -5.398890000 1.344819000 0.508199000
H -4.595640000 -1.629094000 1.771952000	C 4.117898000 3.430596000 0.065732000
C 3.408441000 3.710122000 -0.351071000	C 1.890049000 2.954425000 -0.890727000
C 1.276865000 2.678952000 -1.073724000	C 2.308736000 2.590260000 1.579076000
C 1.967273000 2.598497000 1.359605000	H 4.977243000 0.872020000 1.247330000
H 4.944453000 1.328427000 0.822519000	H 5.456432000 1.250282000 -0.416601000
H 5.132806000 1.875710000 -0.851064000	C 2.334232000 -2.796922000 -1.446135000
C 2.362858000 -2.929038000 0.595960000	C 4.002068000 -3.481400000 0.275966000
C 4.611695000 -3.168525000 -0.391473000	C 1.745303000 -2.804267000 1.026273000
C 4.304184000 -1.971596000 1.814213000	H 5.004206000 -0.965062000 -1.175674000
H 5.031853000 -0.315857000 -1.733396000	H 5.398374000 -1.345601000 0.508796000
H 5.703318000 -0.708000000 -0.150439000	H -1.451057000 -1.929746000 1.688997000
H -1.669255000 2.014860000 -1.653923000	H -1.983435000 -3.610013000 1.782267000
H -2.409792000 3.622674000 -1.581418000	H -3.051187000 -2.320511000 2.330587000
H -3.370776000 2.224056000 -2.078286000	H -4.876316000 -3.209894000 0.813236000
H -5.084582000 2.764809000 -0.343768000	H -3.784181000 -4.452947000 0.234637000
H -4.094820000 4.174407000 0.034128000	H -4.570567000 -3.387417000 -0.924177000
H -4.602298000 3.097885000 1.326523000	H -2.243698000 -2.713392000 -1.891742000
H -2.214145000 2.693417000 2.048048000	H -1.762953000 -4.034316000 -0.824271000
H -1.668740000 3.866271000 0.847020000	H -0.912398000 -2.506787000 -0.748425000
H -0.983072000 2.235896000 0.862672000	H -4.802368000 3.396898000 -0.458844000
H -2.695454000 -3.999713000 0.600771000	H -3.635774000 4.504681000 0.241680000
H -4.076991000 -4.386887000 -0.438826000	H -4.415147000 3.308689000 1.269750000
H -4.336518000 -3.591698000 1.107949000	H -2.135127000 2.625517000 2.028617000
H -5.604640000 -1.711040000 -0.155070000	H -1.442963000 3.848866000 0.960563000
H -5.279665000 -2.492229000 -1.693880000	H -0.868945000 2.184646000 0.891564000
H -4.770949000 -0.821661000 -1.436298000	H -1.476083000 2.180119000 -1.699297000
H -2.443286000 -1.568402000 -2.214172000	H -2.038693000 3.840590000 -1.550493000
H -3.066988000 -3.212452000 -2.342011000	H -3.121476000 2.598337000 -2.174020000
H -1.628230000 -2.888882000 -1.379310000	H 4.877184000 3.209492000 0.813262000
H 4.253236000 3.713972000 0.337362000	H 3.785471000 4.452750000 0.234312000
H 2.863167000 4.641964000 -0.207911000	H 4.571713000 3.386802000 -0.924219000
H 3.788042000 3.698554000 -1.373278000	H 2.244683000 2.713185000 -1.891951000
H 1.637083000 2.606722000 -2.100684000	H 1.764267000 4.034474000 -0.824792000
H 0.808096000 3.655326000 -0.942259000	H 0.913240000 2.507194000 -0.748672000
H 0.531005000 1.910873000 -0.912042000	H 1.451548000 1.930296000 1.688799000
H 1.291454000 1.773010000 1.564765000	H 1.984245000 3.610472000 1.781883000
H 1.445476000 3.537309000 1.548702000	H 3.051677000 2.320840000 2.330518000
H 2.814626000 2.538948000 2.045760000	H 1.475963000 -2.179385000 -1.699914000
H 1.703117000 -2.378364000 1.257663000	H 2.037749000 -3.840138000 -1.550959000
H 2.541665000 -3.911601000 1.033983000	H 3.121305000 -2.598359000 -2.174058000
H 1.862335000 -3.068125000 -0.361251000	H 4.801418000 -3.397471000 -0.458525000
H 4.184383000 -3.337231000 -1.379804000	H 3.634267000 -4.504933000 0.241568000
H 4.693025000 -4.128551000 0.117916000	H 4.413729000 -3.309267000 1.269958000
H 5.620177000 -2.777647000 -0.516722000	H 2.133985000 -2.625364000 2.028327000
H 5.293395000 -1.516840000 1.758139000	H 1.441543000 -3.848399000 0.960099000
H 4.402233000 -2.906116000 2.367870000	H 0.868092000 -2.183998000 0.891184000
H 3.650898000 -1.307163000 2.380079000	

Table S36: Cartesian coordinates (x y z) of the refined geometry of NHP **A** and its corresponding base at the B3LYP/def2-TZVPP level of theory.

**A** (XH)	(X^+)
P 0.000000000 0.996666000 0.036267000	P 0.000000000 0.932725000 -0.254714000
N -1.217755000 -0.205724000 -0.223436000	N -1.187565000 -0.210652000 0.072684000
N 1.217755000 -0.205723000 -0.223436000	N 1.187564000 -0.210652000 0.072685000
C -2.654077000 0.056631000 -0.012753000	C -2.671989000 0.056191000 -0.003720000
C 2.654077000 0.056631000 -0.012752000	C 2.671988000 0.056191000 -0.003718000
C -0.668913000 -1.478822000 0.037604000	C -0.681210000 -1.428089000 0.421470000
C 0.668913000 -1.478822000 0.037604000	C 0.681209000 -1.428089000 0.421471000
C -2.965228000 1.488682000 -0.454255000	C -2.886183000 1.508706000 -0.419795000
C -3.024629000 -0.119884000 1.467999000	C -3.268128000 -0.190505000 1.383370000
C -3.468691000 -0.910533000 -0.879565000	C -3.267998000 -0.887594000 -1.049915000
C 2.965229000 1.488682000 -0.454255000	C 2.886182000 1.508706000 -0.419793000
C 3.468691000 -0.910534000 -0.879564000	C 3.267998000 -0.887595000 -1.049911000
C 3.024629000 -0.119884000 1.468000000	C 3.268125000 -0.190505000 1.383374000
H 0.000000000 1.002873000 1.508926000	H 2.809857000 0.460895000 2.126650000
H 2.449483000 0.561824000 2.094677000	H 3.144014000 -1.224328000 1.701893000
H 2.814927000 -1.136021000 1.801760000	H 4.335758000 0.020153000 1.356025000
H 4.085005000 0.079983000 1.626346000	H 3.955144000 1.703515000 -0.475510000
H 4.035735000 1.673670000 -0.369544000	H 2.465595000 1.717018000 -1.404498000
H 2.664058000 1.649025000 -1.488944000	H 2.465586000 2.206721000 0.305358000
H 2.451891000 2.224185000 0.166568000	H 3.144039000 -1.933206000 -0.772436000
H 3.318265000 -1.947600000 -0.582458000	H 2.809292000 -0.728747000 -2.025182000
H 3.184452000 -0.810186000 -1.926678000	H 4.335569000 -0.694376000 -1.138808000
H 4.532480000 -0.692429000 -0.782453000	H 1.324120000 -2.256981000 0.658676000
H 1.300206000 -2.346046000 0.115900000	H -1.324122000 -2.256981000 0.658675000

H -1.300206000 -2.346045000 0.115900000	H -2.809860000 0.460894000 2.126648000
H -2.449484000 0.561823000 2.094676000	H -4.335760000 0.020153000 1.356020000
H -4.085006000 0.079983000 1.626344000	H -3.144018000 -1.224329000 1.701888000
H -2.814929000 -1.136021000 1.801759000	H -2.809291000 -0.728745000 -2.025186000
H -3.184451000 -0.810186000 -1.926679000	H -3.144038000 -1.933205000 -0.772441000
H -3.318268000 -1.947599000 -0.582460000	H -4.335569000 -0.694376000 -1.138813000
H -4.532480000 -0.692426000 -0.782455000	H -2.465594000 1.717019000 -1.404500000
H -2.664056000 1.649025000 -1.488944000	H -3.955145000 1.703516000 -0.475514000
H -4.035733000 1.673671000 -0.369545000	H -2.465587000 2.206721000 0.305357000
H -2.451890000 2.224185000 0.166568000	

Table S37: Cartesian coordinates (x y z) of the refined geometry of NHP **B** and its corresponding base at the B3LYP/def2-TZVPP level of theory.

**B** (XH)	(X^+)
P -0.007540000 -0.970066000 0.126927000	P 0.004714000 -0.814747000 -0.402303000
N 1.215100000 0.187817000 -0.153175000	N 1.209395000 0.197540000 0.014180000
N -1.211508000 0.220325000 -0.163869000	N -1.205381000 0.180414000 0.039032000
C 2.652271000 -0.107639000 -0.068117000	C 2.673169000 -0.095112000 -0.098643000
C -2.651415000 -0.099364000 -0.090552000	C -2.667542000 -0.110068000 -0.097336000
C 0.753004000 1.558000000 0.082761000	C 0.737127000 1.546669000 0.395284000
C -0.737915000 1.470299000 0.425984000	C -0.740038000 1.390512000 0.751451000
C 2.912499000 -1.514207000 -0.615347000	C 2.857120000 -1.494521000 -0.681889000
C 3.151985000 -0.030354000 1.385721000	C 3.287431000 -0.033108000 1.303524000
C 3.420288000 0.900287000 -0.933074000	C 3.311397000 0.945690000 -1.023162000
C -2.902249000 -1.400372000 -0.859076000	C -2.843960000 -1.499741000 -0.705680000
C -3.442190000 1.023508000 -0.774004000	C -3.284844000 0.943151000 -1.023033000
C -3.135642000 -0.259566000 1.360575000	C -3.308737000 -0.061556000 1.292589000
H -2.604326000 -1.068808000 1.861785000	H -2.840684000 -0.779997000 1.965497000
H -2.979933000 0.653968000 1.934510000	H -3.244547000 0.930256000 1.737429000
H -4.201865000 -0.487931000 1.384731000	H -4.364388000 -0.313325000 1.209094000
H -3.971491000 -1.609033000 -0.893160000	H -3.906516000 -1.703900000 -0.819689000
H -2.528379000 -1.317800000 -1.879260000	H -2.391010000 -1.571814000 -1.696020000
H -2.408205000 -2.249611000 -0.387972000	H -2.425574000 -2.279537000 -0.067666000
H -3.380991000 1.960894000 -0.222401000	H -3.197264000 1.948696000 -0.614614000
H -3.066231000 1.190387000 -1.783357000	H -2.814234000 0.921354000 -2.005397000
H -4.495545000 0.750124000 -0.835871000	H -4.346158000 0.735247000 -1.148665000
H -1.276541000 2.325472000 0.021004000	H -1.316768000 2.257884000 0.443812000
H 0.906247000 2.160740000 -0.815956000	H 0.878508000 2.219177000 -0.451223000
H 2.632156000 -0.758521000 2.008649000	H 2.819006000 -0.760337000 1.965871000
H 4.222020000 -0.235952000 1.439724000	H 4.349994000 -0.262474000 1.242250000
H 2.983245000 0.959255000 1.810867000	H 3.194179000 0.956650000 1.747689000
H 3.057853000 0.871812000 -1.960800000	H 2.845320000 0.933150000 -2.008377000
H 3.313242000 1.918129000 -0.559288000	H 3.241793000 1.952437000 -0.614126000
H 4.483264000 0.658728000 -0.932743000	H 4.368351000 0.716155000 -1.145319000
H 2.538179000 -1.605662000 -1.634453000	H 2.441067000 -1.574463000 -1.687225000
H 3.983584000 -1.716407000 -0.615636000	H 3.920819000 -1.713193000 -0.748508000
H 2.428113000 -2.279875000 -0.008982000	H 2.406424000 -2.262003000 -0.050248000
H 1.291598000 2.042413000 0.903253000	H 1.310556000 1.926578000 1.235781000
H -0.866877000 1.473733000 1.517241000	H -0.882924000 1.243047000 1.822271000
H -0.045860000 -0.975276000 1.585500000	

3.4 Determination of Bond Dissociation Free Energies (BDFEs)

All structures were optimized using the B3LYP[18,19] functional and the 6-311+g(d,p)[20–27] basis set including a PCM solvent model for toluene. A frequency calculation at the same level of theory was performed of the final structure to obtain ΔG values and confirm the calculated structure is a local minimum.

To determine the BDFE the free reaction energy of the hydrogen radical exchange with TEMPO and the chosen compound is calculated. To obtain the BDFE of the chosen compound the BDFE of the TEMPO-H is added. The BDFE value for TEMPO-H in benzene of 65.2 kcal mol^{-1} is employed for this method.[49] It is assumed that the BDFE value in benzene is equal to BDFE value in toluene.[50]

ΔG: X-H + TEMPO· → X· + TEMPO-H

ΔG = G(X·) + G(TEMPO-H) - G(X-H) - G(TEMPO·)

BDFE(TEMPO-H) = G(TEMPO·) + G(H·) – G(TEMPO-H)

BDFE(X-H) = ΔG + BDFE(TEMPO-H) with BDFE(TEMPO-H) = 65.2 kcal mol^{-1}

= [G(X·) + G(TEMPO-H) - G(X-H) - G(TEMPO·)] + [G(TEMPO·) + G(H·) – G(TEMPO-H)]

= G(X·) - G(X-H) + G(H·)

= G(X·) + G(H·) - G(X-H)

Table S 38: Calculated ΔG and BDFE values in toluene.

Compound	ΔG / kcal mol^{-1}	**BDFE** / kcal mol^{-1}
**1**	-0.3	64.9
**3**	3.2	68.4

Table S39: Cartesian coordinates (x y z) of the refined geometry of **1** and its corresponding radical (**P(R^1)$_2$·, X·**) at the B3LYP/6-311++G(d,p) level of theory using the PCM solvent model for toluene.

**1** (X-H)				**P(R^1)$_2$·** (X·)			
N	3.235260000	1.108275000	-0.710216000	N	3.318804000	-1.136392000	0.486996000
C	2.585943000	0.044064000	-0.066991000	C	2.584720000	-0.010502000	0.066648000
C	2.567635000	2.360302000	-1.180688000	C	2.720615000	-2.436467000	0.915477000
C	4.599150000	0.851706000	-0.773910000	C	4.675365000	-0.858035000	0.428091000
N	3.616209000	-0.862133000	0.247212000	N	3.569963000	0.953799000	-0.249309000
N	1.325823000	0.021404000	0.211087000	N	1.295142000	-0.011641000	0.017517000
C	1.959046000	3.087285000	0.031491000	C	1.954356000	-3.052614000	-0.268848000
C	3.608918000	3.278096000	-1.840263000	C	3.841348000	-3.401791000	1.333294000
C	1.491877000	2.012991000	-2.225923000	C	1.797167000	-2.206248000	2.126473000
C	4.831243000	-0.344031000	-0.200968000	C	4.829677000	0.403742000	-0.014919000
H	5.300239000	1.521224000	-1.234487000	H	5.432323000	-1.565280000	0.708695000
C	3.490598000	-2.093614000	1.088959000	C	3.362174000	2.319232000	-0.827548000
P	0.045883000	-1.028094000	-0.084519000	P	-0.000001000	1.048923000	-0.000027000
H	1.245527000	2.443313000	0.542586000	H	1.165964000	-2.382639000	-0.605117000
H	1.446873000	3.994850000	-0.299113000	H	1.507596000	-4.003474000	0.034272000
H	2.745963000	3.374329000	0.735029000	H	2.636963000	-3.245034000	-1.101679000
H	4.391605000	3.582731000	-1.141284000	H	4.521807000	-3.625619000	0.507979000
H	3.100597000	4.182804000	-2.179614000	H	3.382997000	-4.342054000	1.646246000
H	4.073078000	2.811506000	-2.712914000	H	4.420008000	-3.018608000	2.177576000
H	1.939815000	1.481926000	-3.070392000	H	2.364487000	-1.783409000	2.960569000
H	1.038175000	2.933808000	-2.602798000	H	1.373639000	-3.160847000	2.450759000
H	0.711485000	1.391233000	-1.793503000	H	0.986879000	-1.526707000	1.871790000
H	5.763566000	-0.863686000	-0.092666000	H	5.741256000	0.945258000	-0.175962000
C	2.765459000	-1.748969000	2.403292000	C	2.541646000	2.214461000	-2.126264000
C	4.893451000	-2.614935000	1.448073000	C	4.724861000	2.939322000	-1.186662000
C	2.765341000	-3.197531000	0.297366000	C	2.693030000	3.230242000	0.218440000
N	-1.287701000	-0.004787000	0.122162000	N	-1.295154000	-0.011631000	-0.017524000
H	-0.063755000	-1.717917000	1.189172000	H	3.062676000	1.580573000	-2.849017000
H	3.342175000	-1.011509000	2.968303000	H	2.425407000	3.209599000	-2.563890000
H	2.672920000	-2.651424000	3.013085000	H	1.546440000	1.805738000	-1.956729000
H	1.771119000	-1.344431000	2.229049000	H	5.357543000	3.087408000	-0.308438000
H	5.456536000	-2.929424000	0.566216000	H	4.542708000	3.921280000	-1.627416000
H	4.776865000	-3.490005000	2.090384000	H	5.267947000	2.340872000	-1.921740000
H	5.477027000	-1.872767000	1.997422000	H	1.716493000	2.859001000	0.527379000
H	1.764269000	-2.898627000	-0.012494000	H	2.555125000	4.232046000	-0.198144000
H	2.680179000	-4.099420000	0.910061000	H	3.328468000	3.311941000	1.104592000
H	3.332352000	-3.447327000	-0.603499000	C	-2.584732000	-0.010450000	-0.066633000
C	-2.572468000	-0.039713000	0.112418000	N	-3.318880000	-1.136303000	-0.486969000
N	-3.343898000	1.034010000	0.598374000	N	-3.569916000	0.953904000	0.249332000
N	-3.528610000	-0.993025000	-0.311391000	C	-2.720769000	-2.436406000	-0.915474000
C	-2.785855000	2.290168000	1.182365000	C	-4.675425000	-0.857866000	-0.428060000
C	-4.694824000	0.735711000	0.476268000	C	-3.362018000	2.319331000	0.827548000
C	-3.281407000	-2.265506000	-1.059992000	C	-4.829664000	0.403917000	0.014961000
C	-4.809965000	-0.486449000	-0.070260000	C	-1.954633000	-3.052682000	0.268864000
C	-1.967164000	3.027799000	0.108220000	C	-3.841556000	-3.401616000	-1.333408000
C	-3.939137000	3.201344000	1.632109000	C	-1.797230000	-2.206196000	-2.126402000
C	-1.921443000	1.950980000	2.411649000	H	-5.432426000	-1.565066000	-0.708663000
H	-5.473401000	1.402776000	0.793648000	C	-2.541420000	2.214513000	2.126215000
C	-2.484590000	-1.966071000	-2.342984000	C	-4.724640000	2.939526000	1.186730000
C	-4.626526000	-2.886683000	-1.479844000	C	-2.692856000	3.230278000	-0.218483000
C	-2.569601000	-3.285160000	-0.151064000	H	-5.741211000	0.945483000	0.176011000
H	-5.704312000	-1.033139000	-0.296258000	H	-1.166190000	-2.382804000	0.605202000
H	-1.152958000	2.400479000	-0.247536000	H	-1.507956000	-4.003575000	-0.034277000
H	-1.550616000	3.948037000	0.527252000	H	-2.637303000	-3.245070000	1.101650000
H	-2.606752000	3.295259000	-0.738076000	H	-4.522104000	-3.625402000	-0.508155000
H	-4.583111000	3.492018000	0.798275000	H	-3.383265000	-4.341913000	-1.646346000
H	-3.510098000	4.113834000	2.050996000	H	-4.420112000	-3.018361000	-2.177729000
H	-4.551591000	2.736786000	2.408931000	H	-2.364470000	-1.783282000	-2.960516000
H	-2.525720000	1.448712000	3.172628000	H	-1.373745000	-3.160808000	-2.450703000
H	-1.523104000	2.872450000	2.845497000	H	-0.986914000	-1.526719000	-1.871639000
H	-1.093027000	1.302726000	2.133375000	H	-3.062454000	1.580674000	2.849007000
H	-3.036178000	-1.260587000	-2.970464000	H	-2.425077000	3.209648000	2.563821000
H	-2.342595000	-2.890524000	-2.909546000	H	-1.546256000	1.805709000	1.956622000
H	-1.500806000	-1.553401000	-2.127017000	H	-5.357361000	3.087646000	0.308539000
H	-5.245903000	-3.152575000	-0.619972000	H	-4.542391000	3.921477000	1.627459000
H	-4.416617000	-3.806089000	-2.029669000	H	-5.267728000	2.341127000	1.921847000
H	-5.195329000	-2.228157000	-2.139855000	H	-1.716359000	2.858960000	-0.527459000
H	-1.593957000	-2.940816000	0.183544000	H	-2.554861000	4.232079000	0.198078000
H	-2.420373000	-4.222613000	-0.693499000	H	-3.328326000	3.312008000	-1.104610000
H	-3.182102000	-3.491868000	0.730480000				

Table S40: Cartesian coordinates (x y z) of the refined geometry of **3** and its corresponding radical (**$P(R^3)_2\cdot$, X·**) at the B3LYP/6-311++G(d,p) level of theory using the PCM solvent model for toluene.

**3** (X-H)				**$P(R^3)_2\cdot$** (X·)			
N	-1.261282000	0.085150000	-0.449582000	N	1.277650000	-0.158686000	0.121659000
N	1.260748000	0.057398000	0.210337000	N	-1.277613000	-0.158697000	-0.121605000
C	-2.440898000	-0.035956000	0.022156000	C	2.531844000	0.128122000	0.171376000
P	0.049349000	-1.022880000	-0.371970000	P	0.000015000	0.943983000	0.000018000
C	2.502530000	-0.001017000	-0.107039000	C	-2.531811000	0.128094000	-0.171331000
N	-3.380622000	0.998414000	0.036688000	N	3.490517000	-0.744608000	0.676073000
N	-3.003461000	-1.177734000	0.695258000	N	3.160879000	1.376023000	-0.161971000
H	-0.278476000	-1.674325000	0.864260000	N	-3.490457000	-0.744634000	-0.676085000
N	3.367745000	1.081201000	0.017713000	N	-3.160877000	1.375979000	0.162017000
N	3.216413000	-1.103091000	-0.684495000	C	3.195852000	-2.085435000	1.261626000
C	-3.117047000	2.391784000	-0.423968000	C	4.712864000	-0.012642000	1.027435000
C	-4.391597000	0.742863000	1.069591000	C	3.448256000	1.688656000	-1.613773000
C	-3.586247000	-2.302917000	-0.129619000	C	4.327421000	1.447165000	0.738714000
C	-3.925246000	-0.587327000	1.684256000	C	-3.195760000	-2.085446000	-1.261657000
C	2.958821000	2.457027000	0.428450000	C	-4.712789000	-0.012668000	-1.027496000
C	4.594741000	0.854734000	-0.752424000	C	-3.448376000	1.688567000	1.613806000
C	3.624209000	-2.271475000	0.182114000	C	-4.327361000	1.447136000	-0.738742000
C	4.311072000	-0.480477000	-1.453464000	C	2.414428000	-2.944697000	0.249422000
C	-2.582097000	2.382468000	-1.868208000	C	4.527708000	-2.805789000	1.547397000
C	-4.451008000	3.164212000	-0.434898000	C	2.398621000	-1.930189000	2.571691000
C	-2.120339000	3.088857000	0.522508000	H	5.569052000	-0.353783000	0.436239000
H	-5.397215000	0.685895000	0.640837000	H	4.961560000	-0.139509000	2.083826000
H	-4.394109000	1.531219000	1.827157000	C	3.726363000	3.199238000	-1.705256000
C	-3.737412000	-3.517482000	0.803106000	C	4.657702000	0.890253000	-2.154625000
C	-4.956063000	-1.935233000	-0.746974000	C	2.236859000	1.367318000	-2.498466000
C	-2.642235000	-2.691057000	-1.274365000	H	4.026579000	1.946976000	1.664067000
H	-3.373476000	-0.401864000	2.610273000	H	5.146065000	2.017839000	0.306968000
H	-4.752676000	-1.251767000	1.921502000	C	-4.527605000	-2.805757000	-1.547585000
C	4.222461000	3.321279000	0.606568000	C	-2.398401000	-1.930165000	-2.571640000
C	2.053379000	3.081998000	-0.650100000	C	-2.414454000	-2.944767000	-0.249412000
C	2.239757000	2.413151000	1.790264000	H	-5.569003000	-0.353817000	-0.436342000
H	5.476459000	0.817348000	-0.103488000	H	-4.961435000	-0.139524000	-2.083900000
H	4.753046000	1.645336000	-1.489130000	C	-2.237042000	1.367273000	2.498602000
C	2.450380000	-2.763852000	1.035954000	C	-3.726568000	3.199133000	1.705294000
C	4.038233000	-3.419714000	-0.755913000	C	-4.657829000	0.890096000	2.154540000
C	4.791091000	-1.906130000	1.128431000	H	-4.026462000	1.946958000	-1.664070000
H	3.967525000	-0.306185000	-2.477305000	H	-5.146031000	2.017805000	-0.307038000
H	5.190889000	-1.116753000	-1.504640000	H	1.449821000	-2.500326000	0.017034000
H	-1.626471000	1.868973000	-1.932130000	H	2.258347000	-3.942446000	0.669558000
H	-2.461495000	3.413158000	-2.213749000	H	2.985539000	-3.051529000	-0.677478000
H	-3.292943000	1.881996000	-2.532175000	H	5.137600000	-2.885084000	0.643583000
H	-5.191936000	2.661130000	-1.062106000	H	4.311099000	-3.818540000	1.894475000
H	-4.279550000	4.159885000	-0.850428000	H	5.117071000	-2.313844000	2.323587000
H	-4.872277000	3.294891000	0.563840000	H	2.956601000	-1.330997000	3.297965000
H	-2.514305000	3.127402000	1.542957000	H	2.210238000	-2.910686000	3.019031000
H	-1.939423000	4.117143000	0.195492000	H	1.441501000	-1.445755000	2.377730000
H	-1.170258000	2.554936000	0.535650000	H	2.863670000	3.763101000	-1.343747000
H	-2.767774000	-3.792625000	1.223988000	H	3.917580000	3.482581000	-2.743926000
H	-4.130001000	-4.371904000	0.244956000	H	4.598678000	3.496962000	-1.118317000
H	-4.424285000	-3.322584000	1.630094000	H	5.583916000	1.126495000	-1.625605000
H	-5.712125000	-1.725186000	0.012993000	H	4.817543000	1.130547000	-3.209280000
H	-5.325997000	-2.770195000	-1.348296000	H	4.479380000	-0.185416000	-2.079277000
H	-4.868604000	-1.063430000	-1.399813000	H	1.994703000	0.303228000	-2.488564000
H	-2.489101000	-1.871579000	-1.978328000	H	2.471095000	1.651494000	-3.527817000
H	-3.082513000	-3.527461000	-1.823394000	H	1.352218000	1.920049000	-2.181724000
H	-1.667961000	-3.011723000	-0.904482000	H	-5.137582000	-2.885081000	-0.643831000
H	4.912308000	2.873077000	1.326561000	H	-4.310986000	-3.818496000	-1.894690000
H	3.925684000	4.298986000	0.992752000	H	-5.116886000	-2.313762000	-2.323806000
H	4.756919000	3.489753000	-0.330323000	H	-2.956298000	-1.330932000	-3.297943000
H	2.576020000	3.135385000	-1.610327000	H	-2.209996000	-2.910647000	-3.019001000
H	1.768818000	4.099402000	-0.365553000	H	-1.441288000	-1.445759000	-2.377569000
H	1.148253000	2.488452000	-0.777117000	H	-1.449855000	-2.500433000	-0.016918000
H	1.332765000	1.816676000	1.735320000	H	-2.258363000	-3.942505000	-0.669571000
H	1.985582000	3.431770000	2.097148000	H	-2.985653000	-3.051618000	0.677431000
H	2.897453000	1.982680000	2.551236000	H	-1.994823000	0.303198000	2.488692000
H	2.093838000	-2.000088000	1.727875000	H	-2.471393000	1.651406000	3.527939000
H	2.781426000	-3.624090000	1.623536000	H	-1.352405000	1.920068000	2.181962000
H	1.618797000	-3.086479000	0.407939000	H	-2.863872000	3.763048000	1.343875000
H	3.213642000	-3.673172000	-1.425764000	H	-3.917894000	3.482445000	2.743953000
H	4.293999000	-4.305222000	-0.167784000	H	-4.598846000	3.496823000	1.118283000
H	4.909503000	-3.170575000	-1.365675000	H	-5.584009000	1.126300000	1.625443000
H	5.690571000	-1.611781000	0.582513000	H	-4.817773000	1.130365000	3.209185000
H	5.054903000	-2.768842000	1.746367000	H	-4.479446000	-0.185564000	2.079190000
H	4.508660000	-1.088249000	1.796314000				

Table S41: Cartesian coordinates (x y z) of the refined geometry of **TEMPO-H** and its corresponding radical (**TEMPO·**) at the B3LYP/6-311++G(d,p) level of theory using the PCM solvent model for toluene.

**TEMPO-H**				**TEMPO·**			
C	1.300667000	-0.055939000	-0.043064000	C	1.334255000	-0.072445000	-0.025260000
C	1.248849000	1.434566000	-0.444262000	C	1.244719000	1.392991000	-0.494282000
C	0.000058000	2.158170000	0.059107000	C	0.000005000	2.124008000	0.004972000
H	1.272592000	1.493862000	-1.537965000	H	1.243505000	1.411245000	-1.590287000
H	2.159114000	1.920429000	-0.079032000	H	2.157361000	1.904363000	-0.173317000
C	-1.248710000	1.434625000	-0.444365000	C	-1.244733000	1.393005000	-0.494237000
H	0.000098000	3.191890000	-0.300850000	H	-0.000001000	3.152044000	-0.369554000
H	0.000009000	2.217949000	1.152455000	H	0.000026000	2.196074000	1.097378000
C	-1.300679000	-0.055845000	-0.043048000	C	-1.334250000	-0.072450000	-0.025262000
H	-1.272318000	1.493819000	-1.538074000	H	-1.243596000	1.411313000	-1.590239000
H	-2.158984000	1.920578000	-0.079275000	H	-2.157354000	1.904362000	-0.173185000
N	-0.000045000	-0.665768000	-0.448427000	N	0.000002000	-0.752899000	-0.195757000
C	2.396689000	-0.748408000	-0.873160000	C	2.349847000	-0.825260000	-0.897570000
H	2.512122000	-1.794483000	-0.586515000	H	2.474584000	-1.852895000	-0.558205000
H	3.354169000	-0.241553000	-0.722306000	H	3.316243000	-0.316829000	-0.844295000
H	2.143977000	-0.711827000	-1.934661000	H	2.026262000	-0.842651000	-1.941087000
C	-2.396740000	-0.748294000	-0.873101000	C	-2.349827000	-0.825259000	-0.897601000
H	-2.512060000	-1.794398000	-0.586505000	H	-2.474590000	-1.852884000	-0.558217000
H	-2.144151000	-0.711629000	-1.934631000	H	-2.026194000	-0.842675000	-1.941102000
H	-3.354234000	-0.241505000	-0.722132000	H	-3.316215000	-0.316810000	-0.844376000
C	1.661487000	-0.202989000	1.455756000	C	1.763556000	-0.166029000	1.453518000
H	2.697877000	0.109665000	1.607937000	H	2.793686000	0.184880000	1.558967000
H	1.598183000	-1.243269000	1.788900000	H	1.715600000	-1.203287000	1.789647000
H	1.036637000	0.403654000	2.110370000	H	1.134078000	0.439001000	2.107963000
C	-1.661484000	-0.202891000	1.455766000	C	-1.763571000	-0.166059000	1.453509000
H	-1.036422000	0.403490000	2.110416000	H	-1.134142000	0.439030000	2.107953000
H	-1.598436000	-1.243249000	1.788702000	H	-1.715554000	-1.203310000	1.789650000
H	-2.697774000	0.110034000	1.608056000	H	-2.793725000	0.184784000	1.558938000
O	-0.000122000	-2.066160000	-0.163188000	O	0.000002000	-2.027560000	-0.065382000
H	0.000069000	-2.171770000	0.801835000				

4 References

[1] P. Löwe, F. Dielmann, *Chem. Commun.* **2022**, *58*, 11831.

[2] M. D. Böhme, T. Eder, M. B. Röthel, P. D. Dutschke, L. F. B. Wilm, E. Hahn, F. Dielmann, *Angew. Chem. Int. Ed.* **2022**, *61*, e202202190.

[3] P. Löwe, M. A. Wünsche, F. R. S. Purtscher, J. Gamper, T. S. Hofer, L. F. B. Wilm, M. B. Röthel, F. Dielmann, *Chem. Sci.* **2023**, *14*, 7928.

[4] H. M. J. Wang, C. S. Vasam, T. Y. R. Tsai, S.-H. Chen, A. H. H. Chang, I. J. B. Lin, *Organometallics* **2005**, *24*, 486.

[5] H. Chen, M. Yang, G. Wang, L. Gao, Z. Ni, J. Zou, S. Li, *Org. Lett.* **2021**, *23*, 5533.

[6] D. J. Nelson, J. D. Egbert, S. P. Nolan, *Dalton Trans.* **2013**, *42*, 4105.

[7] O. Back, B. Donnadieu, M. von Hopffgarten, S. Klein, R. Tonner, G. Frenking, G. Bertrand, *Chem. Sci.* **2011**, *2*, 858.

[8] J. J. Dunsford, E. R. Clark, M. J. Ingleson, *Angew. Chem. Int. Ed.* **2015**, *54*, 5688.

[9] M. Duncan, M. J. Gallagher, *Org. Magn. Reson.* **1981**, *15*, 37.

[10] A. M. Aguiar, T. G. Archibald, *Tetrahedron Lett.* **1966**, *7*, 5471.

[11] L. Horner, I. Ertel, H.-D. Ruprecht, O. Bêlovský, *Chem. Ber.* **1970**, *103*, 1582.

[12] M.-P. Simonnin, R.-M. Lequan, F. W. Wehrli, *Tetrahedron Lett.* **1972**, *13*, 1559.

[13] W. J. Stec, N. Goddard, J. R. van Wazer, *J. Phys. Chem.* **1971**, *75*, 3547.

[14] G. M. Sheldrick, *Acta Cryst.* **2015**, *A71*, 3.

[15] O. V. Dolomanov, L. J. Bourhis, R. J. Gildea, J. A. K. Howard, H. Puschmann, *J. Appl. Crystallogr.* **2009**, *42*, 339.

[16] G. M. Sheldrick, *Acta Cryst.* **2015**, *C71*, 3.

[17] M. J. Frisch, G. W. Trucks, H. B. Schlegel, G. E. Scuseria, M. A. Robb, J. R. Cheeseman, G. Scalmani, V. Barone, G. A. Petersson, H. Nakatsuji, X. Li, M. Caricato, A. V. Marenich, J. Bloino, B. G. Janesko, R. Gomperts, B. Mennucci, H. P. Hratchian, J. V. Ortiz, A. F. Izmaylov, J. L. Sonnenberg, D. Williams-Young, F. Ding, F. Lipparini, F. Egidi, J. Goings, B. Peng, A. Petrone, T. Henderson, D. Ranasinghe, V. G. Zakrzewski, J. Gao, N. Rega, G. Zheng, W. Liang, M. Hada, M. Ehara, K. Toyota, R. Fukuda, J. Hasegawa, M. Ishida, T. Nakajima, Y. Honda, O. Kitao, H. Nakai, T. Vreven, K. Throssell, J. A. Montgomery, Jr., J. E. Peralta, F. Ogliaro, M. J. Bearpark, J. J.

Heyd, E. N. Brothers, K. N. Kudin, V. N. Staroverov, T. A. Keith, R. Kobayashi, J. Normand, K. Raghavachari, A. P. Rendell, J. C. Burant, S. S. Iyengar, J. Tomasi, M. Cossi, J. M. Millam, M. Klene, C. Adamo, R. Cammi, J. W. Ochterski, R. L. Martin, K. Morokuma, O. Farkas, J. B. Foresman and D. J. Fox, "Gaussian 16, Revision C.01. Inc., Wallingford CT 2016", **2016**.

[18] A. D. Becke, *J. Chem. Phys.* **1993**, *98*, 5648.

[19] C. Lee, W. Yang, R. G. Parr, *Phys. Rev. B Condens. Matter* **1988**, *37*, 785.

[20] A. D. McLean, G. S. Chandler, *J. Chem. Phys.* **1980**, *72*, 5639.

[21] R. Krishnan, J. S. Binkley, R. Seeger, J. A. Pople, *J. Chem. Phys.* **1980**, *72*, 650.

[22] M. M. Francl, W. J. Pietro, W. J. Hehre, J. S. Binkley, M. S. Gordon, D. J. DeFrees, J. A. Pople, *J. Chem. Phys.* **1982**, *77*, 3654.

[23] T. Clark, J. Chandrasekhar, G. W. Spitznagel, P. V. R. Schleyer, *J. Comput. Chem.* **1983**, *4*, 294.

[24] G. W. Spitznagel, T. Clark, P. von Ragué Schleyer, W. J. Hehre, *J. Comput. Chem.* **1987**, *8*, 1109.

[25] D. Feller, *J. Comput. Chem.* **1996**, *17*, 1571.

[26] K. L. Schuchardt, B. T. Didier, T. Elsethagen, L. Sun, V. Gurumoorthi, J. Chase, J. Li, T. L. Windus, *J. Chem. Inf. Model.* **2007**, *47*, 1045.

[27] B. P. Pritchard, D. Altarawy, B. Didier, T. D. Gibson, T. L. Windus, *J. Chem. Inf. Model.* **2019**, *59*, 4814.

[28] Zhurko G. A., "Chemcraft - graphical program for visualization of quantum chemistry computations", can be found under https://chemcraftprog.com, **2005**.

[29] T. Yanai, D. P. Tew, N. C. Handy, *Chem. Phys. Lett.* **2004**, *393*, 51.

[30] J. Heyd, G. E. Scuseria, *J. Chem. Phys.* **2004**, *120*, 7274.

[31] J. Heyd, G. E. Scuseria, *J. Chem. Phys.* **2004**, *121*, 1187.

[32] J. Heyd, J. E. Peralta, G. E. Scuseria, R. L. Martin, *J. Chem. Phys.* **2005**, *123*, 174101.

[33] J. Heyd, G. E. Scuseria, M. Ernzerhof, *J. Chem. Phys.* **2006**, *124*.

[34] A. F. Izmaylov, G. E. Scuseria, M. J. Frisch, *J. Chem. Phys.* **2006**, *125*, 104103.

[35] A. V. Krukau, O. A. Vydrov, A. F. Izmaylov, G. E. Scuseria, *J. Chem. Phys.* **2006**, *125*, 224106.

[36] T. M. Henderson, A. F. Izmaylov, G. Scalmani, G. E. Scuseria, *J. Chem. Phys.* **2009**, *131*, 44108.

[37] J.-D. Chai, M. Head-Gordon, *Phys. Chem. Chem. Phys.* **2008**, *10*, 6615.

[38] R. Ditchfield, W. J. Hehre, J. A. Pople, *J. Chem. Phys.* **1971**, *54*, 724.

[39] P. C. Hariharan, J. A. Pople, *Theoret. Chim. Acta* **1973**, *28*, 213.

[40] W. J. Hehre, R. Ditchfield, J. A. Pople, *J. Chem. Phys.* **1972**, *56*, 2257.

[41] M. S. Gordon, J. S. Binkley, J. A. Pople, W. J. Pietro, W. J. Hehre, *J. Am. Chem. Soc.* **1982**, *104*, 2797.

[42] J.-N. Li, L. Liu, Y. Fu, Q.-X. Guo, *Tetrahedron* **2006**, *62*, 4453.

[43] A. V. Marenich, C. J. Cramer, D. G. Truhlar, *J. Phys. Chem. B* **2009**, *113*, 6378.

[44] F. G. Bordwell, *Acc. Chem. Res.* **1988**, *21*, 456.

[45] J. Schlögl, A. L. Brosius, A. N. Toraman, A. Wiesner, S. Steinhauer, C. Müller, S. Riedel, *Angew. Chem. Int. Ed.* **2025**, e202423857.

[46] F. Weigend, R. Ahlrichs, *Phys. Chem. Chem. Phys.* **2005**, *7*, 3297.

[47] S. Grimme, S. Ehrlich, L. Goerigk, *J. Comput. Chem.* **2011**, *32*, 1456.

[48] H. Böhrer, N. Trapp, D. Himmel, M. Schleep, I. Krossing, *Dalton Trans.* **2015**, *44*, 7489.

[49] J. J. Warren, T. A. Tronic, J. M. Mayer, *Chem. Rev.* **2010**, *110*, 6961.

[50] T. R. Porter, J. M. Mayer, *Chem. Sci.* **2014**, *5*, 372.
